# Supplementary material for: Japanese Encephalitis Virus Genotype III Strains Detection and Genome Sequencing from Indian Pig and Mosquito Vector
Source: Vaccines (Basel). 2023 Jan 10;11(1):150. doi: 10.3390/vaccines11010150 (PMC9862938; doi:10.3390/vaccines11010150)

Supplementary Fig. 1: Agarose gel electrophoresis of JEV partial E gene amplified products isolated from pig and mosquito using genotype III specific primers. Here, L: 100bp ladder, 1 to 11: Test samples of JEV isolated from pig sources, 12 and 13: Test samples of JEV isolated from mosquito, P: Positive control of JEV, N: Negative control


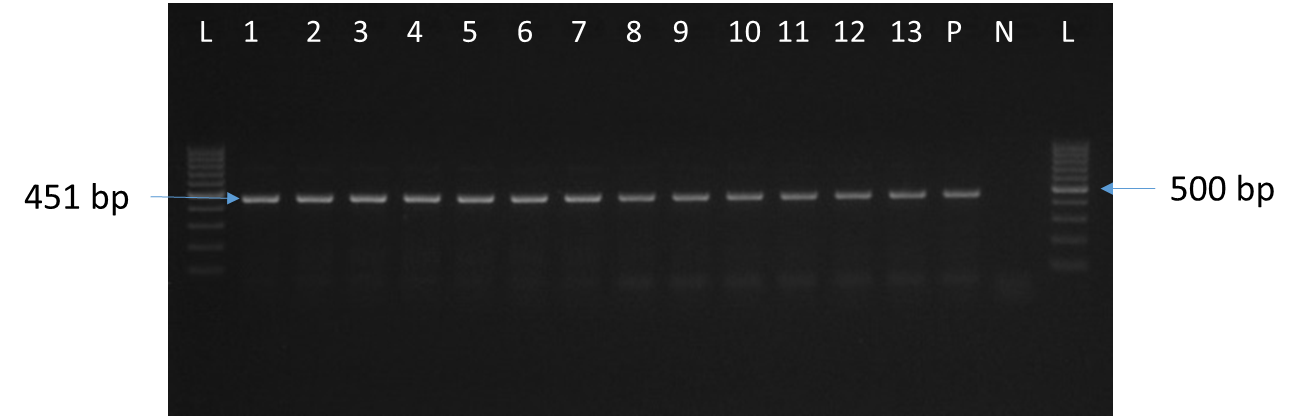

Supplement: Supplementary file 1 [file vaccines-11-00150-s001.zip › vaccines-2082975-supplementary/Supplementary fig 1.docx]
