# Supplementary material for: Japanese Encephalitis Virus Genotype III Strains Detection and Genome Sequencing from Indian Pig and Mosquito Vector
Source: Vaccines (Basel). 2023 Jan 10;11(1):150. doi: 10.3390/vaccines11010150 (PMC9862938; doi:10.3390/vaccines11010150)
Supplement: Supplementary file 1 [file vaccines-11-00150-s001.zip › vaccines-2082975-supplementary/Supplementary File 1.docx]

**Supplementary File 1: Pair Distances by ClustalW (Weighted) of JEV isolated from pig (1a), vector mosquito (1b) and both vector and Host (1c)**

**1a: Pair Distances by ClustalW (Weighted) of JEV isolated from Pig**

|  | **PERCENT IDENTITY** | | | | | | | | | | | | | | | | | | | | | | | |
| --- | --- | --- | --- | --- | --- | --- | --- | --- | --- | --- | --- | --- | --- | --- | --- | --- | --- | --- | --- | --- | --- | --- | --- | --- |
| D  **I**  **V**  **E**  **R**  **G**  **E**  **N**  **C**  **E** |  | 1 | 2 | 3 | 4 | 5 | 6 | 7 | 8 | 9 | 10 | 11 | 12 | 13 | 14 | 15 | 16 | 17 | 18 | 19 | 20 | 21 | 22 |  |
|  | 1 |  | 88.3 | 97.8 | 97.8 | 88.7 | 88.7 | 98.6 | 97.4 | 97.4 | 99.1 | 98.7 | 97.9 | 97.8 | 98.3 | 88.0 | 96.6 | 88.4 | 88.3 | 88.6 | 89.0 | 88.6 | 98.5 | AB551990.1_Japan |
|  | 2 | 13.0 |  | 88.5 | 88.5 | 95.3 | 95.3 | 88.5 | 88.2 | 88.2 | 88.2 | 88.1 | 88.4 | 88.5 | 88.2 | 95.8 | 87.7 | 97.6 | 97.5 | 97.1 | 89.2 | 97.4 | 88.4 | AY316157.1_Korea |
|  | 3 | 2.3 | 12.9 |  | 99.7 | 88.7 | 88.8 | 98.0 | 97.1 | 97.1 | 97.6 | 97.3 | 98.0 | 99.7 | 98.0 | 88.0 | 95.5 | 88.6 | 88.4 | 88.6 | 89.0 | 88.7 | 98.2 | AY849939.1_China |
|  | 4 | 2.2 | 12.9 | 0.3 |  | 88.7 | 88.8 | 98.0 | 97.1 | 97.1 | 97.6 | 97.4 | 98.0 | 99.8 | 98.0 | 88.0 | 95.5 | 88.6 | 88.4 | 88.5 | 89.0 | 88.6 | 98.2 | EF107523.1_China |
|  | 5 | 12.6 | 4.9 | 12.5 | 12.5 |  | 98.2 | 88.7 | 88.4 | 88.4 | 88.5 | 88.4 | 88.6 | 88.8 | 88.6 | 94.7 | 87.8 | 95.5 | 95.4 | 95.2 | 89.6 | 95.5 | 88.7 | GQ902058.1_Thailand |
|  | 6 | 12.6 | 4.9 | 12.5 | 12.5 | 1.9 |  | 88.7 | 88.4 | 88.4 | 88.6 | 88.4 | 88.6 | 88.8 | 88.6 | 94.5 | 87.9 | 95.6 | 95.4 | 95.3 | 89.8 | 95.6 | 88.7 | GQ902061.1_Thailand |
|  | 7 | 1.4 | 12.8 | 2.1 | 2.0 | 12.6 | 12.6 |  | 98.4 | 98.4 | 98.4 | 98.1 | 98.1 | 98.0 | 98.5 | 88.0 | 96.1 | 88.5 | 88.4 | 88.6 | 89.0 | 88.6 | 98.7 | JN381872.1_China |
|  | 8 | 2.7 | 13.2 | 3.0 | 3.0 | 13.0 | 13.0 | 1.7 |  | 99.9 | 97.1 | 96.8 | 97.1 | 97.0 | 97.5 | 87.7 | 95.2 | 88.3 | 88.1 | 88.4 | 88.7 | 88.4 | 97.7 | KC915016.1_China |
|  | 9 | 2.7 | 13.2 | 3.0 | 3.0 | 12.9 | 12.9 | 1.6 | 0.1 |  | 97.2 | 96.8 | 97.2 | 97.1 | 97.5 | 87.7 | 95.2 | 88.3 | 88.1 | 88.4 | 88.7 | 88.4 | 97.7 | KF297916.1_China |
|  | 10 | 0.9 | 13.2 | 2.5 | 2.4 | 12.8 | 12.7 | 1.6 | 2.9 | 2.9 |  | 98.5 | 97.7 | 97.6 | 98.2 | 87.8 | 96.4 | 88.3 | 88.1 | 88.4 | 88.9 | 88.4 | 98.3 | KF711994.1_South Korea |
|  | 11 | 1.3 | 13.4 | 2.7 | 2.7 | 13.0 | 13.0 | 2.0 | 3.3 | 3.2 | 1.5 |  | 97.5 | 97.3 | 97.9 | 87.7 | 96.3 | 88.2 | 88.0 | 88.3 | 88.7 | 88.2 | 98.0 | KP164498.2_India |
|  | 12 | 2.1 | 12.9 | 2.0 | 2.0 | 12.7 | 12.7 | 2.0 | 2.9 | 2.9 | 2.3 | 2.6 |  | 98.0 | 98.1 | 87.9 | 95.6 | 88.4 | 88.4 | 88.5 | 88.9 | 88.6 | 98.3 | KT447437.1_South Korea |
|  | 13 | 2.3 | 12.8 | 0.3 | 0.2 | 12.5 | 12.5 | 2.1 | 3.0 | 3.0 | 2.5 | 2.7 | 2.0 |  | 98.0 | 88.1 | 95.4 | 88.6 | 88.4 | 88.6 | 88.9 | 88.7 | 98.2 | KU363309.1_China |
|  | 14 | 1.7 | 13.2 | 2.1 | 2.0 | 12.8 | 12.8 | 1.5 | 2.6 | 2.6 | 1.9 | 2.2 | 1.9 | 2.1 |  | 87.7 | 95.9 | 88.3 | 88.2 | 88.3 | 88.7 | 88.4 | 99.4 | KX965684.1_China |
|  | 15 | 13.5 | 4.3 | 13.4 | 13.4 | 5.6 | 5.8 | 13.5 | 13.9 | 13.9 | 13.7 | 13.9 | 13.6 | 13.4 | 13.8 |  | 87.3 | 96.0 | 96.0 | 95.7 | 89.0 | 96.0 | 87.9 | KY927818.1_Cambodia |
|  | 16 | 3.5 | 13.9 | 4.7 | 4.7 | 13.7 | 13.6 | 4.1 | 5.1 | 5.0 | 3.7 | 3.9 | 4.6 | 4.8 | 4.2 | 14.4 |  | 87.8 | 87.7 | 87.9 | 88.3 | 87.8 | 96.1 | LC461960.1_Japan |
|  | 17 | 12.9 | 2.5 | 12.7 | 12.7 | 4.6 | 4.6 | 12.8 | 13.1 | 13.1 | 13.1 | 13.2 | 12.9 | 12.7 | 13.1 | 4.1 | 13.7 |  | 97.7 | 97.3 | 89.4 | 97.7 | 88.5 | LC708276._Japan |
|  | 18 | 13.1 | 2.6 | 13.0 | 13.0 | 4.8 | 4.8 | 13.0 | 13.3 | 13.3 | 13.3 | 13.4 | 13.0 | 12.9 | 13.3 | 4.1 | 13.8 | 2.4 |  | 97.9 | 89.4 | 98.3 | 88.3 | MN544780.1_China |
|  | 19 | 12.7 | 2.9 | 12.8 | 12.8 | 5.0 | 4.9 | 12.7 | 13.0 | 13.0 | 12.9 | 13.1 | 12.8 | 12.7 | 13.1 | 4.4 | 13.6 | 2.7 | 2.1 |  | 89.5 | 99.2 | 88.5 | MT232844.1_India |
|  | 20 | 12.2 | 12.0 | 12.2 | 12.2 | 11.4 | 11.2 | 12.1 | 12.6 | 12.5 | 12.4 | 12.5 | 12.3 | 12.3 | 12.5 | 12.2 | 13.0 | 11.8 | 11.7 | 11.6 |  | 89.5 | 88.9 | MT253737.1_Australia |
|  | 21 | 12.8 | 2.6 | 12.6 | 12.7 | 4.7 | 4.6 | 12.7 | 13.0 | 12.9 | 13.0 | 13.2 | 12.7 | 12.6 | 13.0 | 4.1 | 13.7 | 2.3 | 1.8 | 0.8 | 11.6 |  | 88.5 | MZ702743.1_India (NRCP_Assam_2021) |
|  | 22 | 1.5 | 12.9 | 1.9 | 1.8 | 12.6 | 12.6 | 1.3 | 2.4 | 2.4 | 1.7 | 2.0 | 1.7 | 1.9 | 0.6 | 13.6 | 4.0 | 12.9 | 13.0 | 12.9 | 12.4 | 12.8 |  | U15763.1_USA |
|  |  | 1 | 2 | 3 | 4 | 5 | 6 | 7 | 8 | 9 | 10 | 11 | 12 | 13 | 14 | 15 | 16 | 17 | 18 | 19 | 20 | 21 | 22 |  |

**1b: Pair Distances by ClustalW (Weighted) of JEV isolated from vector mosquito**

|  |  | **PERCENT IDENTITY** | | | | | | | | | | | | | | | | | | |  |
| --- | --- | --- | --- | --- | --- | --- | --- | --- | --- | --- | --- | --- | --- | --- | --- | --- | --- | --- | --- | --- | --- |
| D  **I**  **V**  **E**  **R**  **G**  **E**  **N**  **C**  **E** |  | **1** | **2** | **3** | **4** | **5** | **6** | **7** | **8** | **9** | **10** | **11** | **12** | **13** | **14** | **15** | **16** | **17** | **18** | **19** |  |
|  | **1** |  | 88.7 | 96.7 | 88.4 | 88.5 | 97.9 | 88.4 | 97.9 | 88.6 | 88.6 | 88.6 | 88.5 | 84.4 | 88.5 | 89.1 | 88.5 | 88.5 | 88.5 | 88.4 | **AF098735.1_Taiwan** |
|  | **2** | 12.6 |  | 88.4 | 95.5 | 95.7 | 88.4 | 95.7 | 88.4 | 95.8 | 95.8 | 95.8 | 95.9 | 84.0 | 95.5 | 95.2 | 95.7 | 95.5 | 95.8 | 95.5 | **GQ902060.1_Thailand** |
|  | **3** | 3.4 | 13.0 |  | 88.0 | 88.2 | 97.0 | 88.2 | 97.0 | 88.4 | 88.4 | 88.2 | 88.2 | 84.4 | 88.2 | 88.7 | 88.3 | 88.2 | 88.2 | 88.1 | **GQ902063.1_Thailand** |
|  | **4** | 13.0 | 4.7 | 13.4 |  | 97.3 | 88.1 | 97.3 | 88.1 | 97.4 | 97.4 | 97.3 | 97.3 | 83.7 | 97.0 | 96.7 | 97.2 | 97.0 | 97.2 | 97.0 | **HQ652538.1_China** |
|  | **5** | 12.9 | 4.5 | 13.2 | 2.7 |  | 88.2 | 99.6 | 88.2 | 98.3 | 98.3 | 97.7 | 97.8 | 83.7 | 97.5 | 97.9 | 97.6 | 98.1 | 97.7 | 98.1 | **JF499790.1_Taiwan** |
|  | **6** | 2.1 | 12.9 | 3.1 | 13.3 | 13.2 |  | 88.2 | 100.0 | 88.4 | 88.4 | 88.3 | 88.3 | 84.2 | 88.3 | 88.8 | 88.3 | 88.3 | 88.3 | 88.1 | **JN864064.1_China** |
|  | **7** | 12.9 | 4.5 | 13.2 | 2.8 | 0.4 | 13.2 |  | 88.2 | 98.3 | 98.3 | 97.7 | 97.8 | 83.6 | 97.5 | 97.9 | 97.6 | 98.1 | 97.7 | 98.0 | **JQ031753.1_Taiwan** |
|  | **8** | 2.1 | 12.9 | 3.1 | 13.3 | 13.2 | 0.0 | 13.2 |  | 88.4 | 88.4 | 88.3 | 88.3 | 84.3 | 88.3 | 88.8 | 88.3 | 88.3 | 88.3 | 88.1 | **JQ086762.1_China** |
|  | **9** | 12.6 | 4.4 | 13.0 | 2.6 | 1.7 | 13.0 | 1.7 | 13.0 |  | 100.0 | 97.8 | 97.8 | 83.6 | 97.6 | 97.8 | 97.6 | 98.0 | 97.8 | 98.0 | **KT229574.1_China** |
|  | **10** | 12.6 | 4.4 | 13.0 | 2.7 | 1.7 | 13.0 | 1.7 | 13.0 | 0.0 |  | 97.8 | 97.8 | 83.6 | 97.5 | 97.8 | 97.6 | 98.0 | 97.8 | 98.0 | **KT229575.1_China** |
|  | **11** | 12.8 | 4.4 | 13.2 | 2.7 | 2.3 | 13.1 | 2.3 | 13.1 | 2.3 | 2.3 |  | 99.5 | 83.4 | 98.5 | 97.2 | 98.5 | 97.5 | 98.7 | 97.4 | **LC461957.1_Japan** |
|  | **12** | 12.8 | 4.3 | 13.2 | 2.7 | 2.2 | 13.1 | 2.2 | 13.1 | 2.2 | 2.2 | 0.5 |  | 83.5 | 98.5 | 97.3 | 98.6 | 97.6 | 98.7 | 97.5 | **LC513838.1_Japan** |
|  | **13** | 18.0 | 18.5 | 18.1 | 19.0 | 19.0 | 18.2 | 19.0 | 18.2 | 19.1 | 19.1 | 19.4 | 19.3 |  | 83.6 | 83.8 | 83.6 | 83.7 | 83.6 | 83.7 | **LC579814.1_Indonesia** |
|  | **14** | 12.8 | 4.7 | 13.2 | 3.1 | 2.6 | 13.1 | 2.6 | 13.1 | 2.5 | 2.5 | 1.6 | 1.5 | 19.1 |  | 97.0 | 98.6 | 97.2 | 98.8 | 97.1 | **LC623822.1_Japan** |
|  | **15** | 12.1 | 5.0 | 12.6 | 3.4 | 2.2 | 12.4 | 2.2 | 12.4 | 2.3 | 2.3 | 2.9 | 2.8 | 18.9 | 3.1 |  | 97.1 | 98.2 | 97.3 | 98.5 | **MH385014.1_China** |
|  | **16** | 12.5 | 4.3 | 12.8 | 2.7 | 2.2 | 12.8 | 2.2 | 12.8 | 2.1 | 2.2 | 1.3 | 1.2 | 18.9 | 1.1 | 2.7 |  | 97.4 | 98.9 | 97.3 | **MK558811.1_China** |
|  | **17** | 12.8 | 4.7 | 13.2 | 3.1 | 1.9 | 13.1 | 2.0 | 13.1 | 2.0 | 2.0 | 2.6 | 2.5 | 19.0 | 2.9 | 1.8 | 2.4 |  | 97.5 | 98.5 | **MT254426.1_China** |
|  | **18** | 12.8 | 4.3 | 13.2 | 2.8 | 2.4 | 13.1 | 2.4 | 13.1 | 2.3 | 2.3 | 1.3 | 1.3 | 19.1 | 1.2 | 2.8 | 0.8 | 2.6 |  | 97.4 | **MT560941.1_China** |
|  | **19** | 12.9 | 4.7 | 13.4 | 3.1 | 2.0 | 13.3 | 2.0 | 13.3 | 2.0 | 2.0 | 2.7 | 2.6 | 19.0 | 3.0 | 1.5 | 2.5 | 1.6 | 2.7 |  | **ON875960_India** |
|  |  | **1** | **2** | **3** | **4** | **5** | **6** | **7** | **8** | **9** | **10** | **11** | **12** | **13** | **14** | **15** | **16** | **17** | **18** | **19** |  |

**1c: Pair Distances by ClustalW of JEV isolated from Vector vs. Host**

|  | **PERCENT IDENTITY** | | | | | | | | | | | | | | | | | | | | | | | | | | | | | | | | | | | | | | | | | |  |
| --- | --- | --- | --- | --- | --- | --- | --- | --- | --- | --- | --- | --- | --- | --- | --- | --- | --- | --- | --- | --- | --- | --- | --- | --- | --- | --- | --- | --- | --- | --- | --- | --- | --- | --- | --- | --- | --- | --- | --- | --- | --- | --- | --- |
| D  **I**  **V**  **E**  **R**  **G**  **E**  **N**  **C**  **E** |  | 1 | 2 | 3 | 4 | 5 | 6 | 7 | 8 | 9 | 10 | 11 | 12 | 13 | 14 | 15 | 16 | 17 | 18 | 19 | 20 | 21 | 22 | 23 | 24 | 25 | 26 | 27 | 28 | 29 | 30 | 31 | 32 | 33 | 34 | 35 | 36 | 37 | 38 | 39 | 40 | 41 |  |
|  | 1 |  | 97.8 | 88.3 | 97.8 | 97.8 | 88.7 | 88.7 | 88.7 | 97.1 | 88.4 | 88.5 | 98.6 | 98.2 | 88.5 | 98.2 | 97.4 | 97.4 | 99.1 | 98.7 | 88.8 | 88.8 | 97.9 | 97.8 | 98.3 | 88.0 | 88.6 | 96.6 | 88.6 | 84.6 | 88.6 | 88.4 | 89.2 | 88.6 | 88.3 | 88.6 | 89.0 | 88.6 | 88.6 | 88.6 | 88.5 | 98.5 | AB551990.1_Japan_Pig |
|  | 2 | 2.2 |  | 88.3 | 98.0 | 98.0 | 88.6 | 88.7 | 88.6 | 96.7 | 88.4 | 88.5 | 98.0 | 97.9 | 88.4 | 97.9 | 97.1 | 97.2 | 97.7 | 97.4 | 88.6 | 88.6 | 98.9 | 98.0 | 98.1 | 87.9 | 88.6 | 95.5 | 88.5 | 84.4 | 88.5 | 88.4 | 89.1 | 88.5 | 88.3 | 88.5 | 89.0 | 88.5 | 88.5 | 88.5 | 88.4 | 98.3 | AF098735.1_Taiwan_Mosquito |
|  | 3 | 13.0 | 13.0 |  | 88.5 | 88.5 | 95.3 | 95.4 | 95.3 | 88.1 | 97.1 | 97.5 | 88.5 | 88.1 | 97.4 | 88.1 | 88.2 | 88.2 | 88.2 | 88.1 | 97.6 | 97.6 | 88.4 | 88.5 | 88.2 | 95.8 | 97.6 | 87.7 | 97.7 | 83.3 | 97.3 | 97.6 | 97.0 | 97.4 | 97.5 | 97.1 | 89.2 | 97.2 | 97.6 | 97.4 | 97.2 | 88.4 | AY316157.1_Korea_Pig |
|  | 4 | 2.3 | 2.0 | 12.9 |  | 99.7 | 88.7 | 88.8 | 88.8 | 96.7 | 88.5 | 88.6 | 98.0 | 97.8 | 88.6 | 97.8 | 97.1 | 97.1 | 97.6 | 97.3 | 88.9 | 88.9 | 98.0 | 99.7 | 98.0 | 88.0 | 88.7 | 95.5 | 88.7 | 84.4 | 88.6 | 88.6 | 89.1 | 88.7 | 88.4 | 88.6 | 89.0 | 88.7 | 88.7 | 88.7 | 88.5 | 98.2 | AY849939.1_China_Pig |
|  | 5 | 2.2 | 2.0 | 12.9 | 0.3 |  | 88.7 | 88.8 | 88.8 | 96.7 | 88.5 | 88.6 | 98.0 | 97.9 | 88.6 | 97.9 | 97.1 | 97.1 | 97.6 | 97.4 | 88.9 | 88.8 | 98.0 | 99.8 | 98.0 | 88.0 | 88.7 | 95.5 | 88.7 | 84.5 | 88.6 | 88.6 | 89.1 | 88.7 | 88.4 | 88.5 | 89.0 | 88.7 | 88.7 | 88.6 | 88.5 | 98.2 | EF107523.1_China_Pig |
|  | 6 | 12.6 | 12.7 | 4.9 | 12.5 | 12.5 |  | 98.7 | 98.2 | 88.3 | 95.3 | 95.5 | 88.7 | 88.4 | 95.4 | 88.4 | 88.4 | 88.4 | 88.5 | 88.4 | 95.5 | 95.5 | 88.6 | 88.8 | 88.6 | 94.7 | 95.6 | 87.8 | 95.7 | 84.0 | 95.3 | 95.5 | 95.0 | 95.4 | 95.4 | 95.2 | 89.6 | 95.3 | 95.6 | 95.5 | 95.3 | 88.7 | GQ902058.1_Thailand_Pig |
|  | 7 | 12.6 | 12.6 | 4.8 | 12.5 | 12.5 | 1.3 |  | 98.4 | 88.4 | 95.5 | 95.7 | 88.7 | 88.4 | 95.7 | 88.4 | 88.3 | 88.3 | 88.6 | 88.4 | 95.8 | 95.8 | 88.7 | 88.8 | 88.6 | 94.8 | 95.8 | 87.9 | 95.9 | 84.0 | 95.5 | 95.7 | 95.2 | 95.7 | 95.7 | 95.5 | 89.7 | 95.5 | 95.8 | 95.7 | 95.5 | 88.7 | GQ902060.1_Thailand_Mosquito |
|  | 8 | 12.6 | 12.7 | 4.9 | 12.5 | 12.5 | 1.9 | 1.6 |  | 88.3 | 95.4 | 95.5 | 88.7 | 88.4 | 95.5 | 88.4 | 88.4 | 88.4 | 88.6 | 88.4 | 95.6 | 95.6 | 88.6 | 88.8 | 88.6 | 94.5 | 95.6 | 87.9 | 95.7 | 83.9 | 95.4 | 95.6 | 95.1 | 95.5 | 95.4 | 95.3 | 89.8 | 95.3 | 95.7 | 95.6 | 95.4 | 88.7 | GQ902061.1_Thailand_Pig |
|  | 9 | 3.0 | 3.4 | 13.4 | 3.4 | 3.4 | 13.0 | 13.0 | 13.1 |  | 88.0 | 88.2 | 97.1 | 97.0 | 88.2 | 97.0 | 96.2 | 96.2 | 96.8 | 96.6 | 88.4 | 88.4 | 96.7 | 96.7 | 97.1 | 87.6 | 88.2 | 94.9 | 88.2 | 84.4 | 88.2 | 88.1 | 88.7 | 88.3 | 88.0 | 88.2 | 88.3 | 88.2 | 88.2 | 88.2 | 88.1 | 97.4 | GQ902063.1_Thailand_Mosquito |
|  | 10 | 13.0 | 13.0 | 3.0 | 12.9 | 12.8 | 4.9 | 4.7 | 4.8 | 13.4 |  | 97.3 | 88.5 | 88.1 | 97.3 | 88.1 | 88.2 | 88.2 | 88.2 | 88.0 | 97.4 | 97.4 | 88.4 | 88.5 | 88.2 | 96.1 | 97.3 | 87.7 | 97.3 | 83.7 | 97.0 | 97.3 | 96.7 | 97.2 | 97.3 | 97.0 | 89.5 | 97.0 | 97.2 | 97.2 | 97.0 | 88.4 | HQ652538.1_China_Mosquito |
|  | 11 | 12.8 | 12.9 | 2.6 | 12.6 | 12.7 | 4.7 | 4.5 | 4.6 | 13.2 | 2.7 |  | 88.7 | 88.2 | 99.6 | 88.2 | 88.4 | 88.4 | 88.3 | 88.2 | 98.3 | 98.3 | 88.5 | 88.7 | 88.3 | 96.0 | 97.7 | 87.9 | 97.8 | 83.7 | 97.5 | 97.7 | 97.9 | 97.6 | 98.3 | 98.0 | 89.6 | 98.1 | 97.7 | 98.4 | 98.1 | 88.5 | JF499790.1_Taiwan_Mosquito |
|  | 12 | 1.4 | 2.0 | 12.8 | 2.1 | 2.0 | 12.6 | 12.6 | 12.6 | 2.9 | 12.8 | 12.6 |  | 98.3 | 88.6 | 98.3 | 98.4 | 98.4 | 98.4 | 98.1 | 88.8 | 88.7 | 98.1 | 98.0 | 98.5 | 88.0 | 88.6 | 96.1 | 88.6 | 84.6 | 88.6 | 88.5 | 89.2 | 88.6 | 88.4 | 88.6 | 89.0 | 88.6 | 88.6 | 88.6 | 88.5 | 98.7 | JN381872.1_China_Pig |
|  | 13 | 1.8 | 2.1 | 13.3 | 2.2 | 2.2 | 12.9 | 12.9 | 12.9 | 3.1 | 13.3 | 13.2 | 1.7 |  | 88.2 | 100.0 | 97.3 | 97.4 | 98.0 | 97.7 | 88.4 | 88.4 | 97.9 | 97.8 | 99.6 | 87.6 | 88.3 | 95.8 | 88.3 | 84.2 | 88.3 | 88.2 | 88.8 | 88.3 | 88.0 | 88.2 | 88.6 | 88.3 | 88.3 | 88.3 | 88.1 | 99.3 | JN864064.1_China_Mosquito |
|  | 14 | 12.8 | 12.9 | 2.6 | 12.7 | 12.7 | 4.8 | 4.5 | 4.7 | 13.2 | 2.8 | 0.4 | 12.7 | 13.2 |  | 88.2 | 88.3 | 88.3 | 88.3 | 88.2 | 98.3 | 98.3 | 88.5 | 88.7 | 88.3 | 96.0 | 97.7 | 87.9 | 97.8 | 83.6 | 97.5 | 97.7 | 97.9 | 97.6 | 98.2 | 98.0 | 89.6 | 98.1 | 97.7 | 98.4 | 98.0 | 88.5 | JQ031753.1_Taiwan_Mosquito |
|  | 15 | 1.8 | 2.1 | 13.3 | 2.2 | 2.2 | 12.9 | 12.9 | 12.9 | 3.1 | 13.3 | 13.2 | 1.7 | 0.0 | 13.2 |  | 97.3 | 97.4 | 98.0 | 97.7 | 88.4 | 88.4 | 98.0 | 97.8 | 99.6 | 87.6 | 88.3 | 95.8 | 88.3 | 84.3 | 88.3 | 88.2 | 88.8 | 88.3 | 88.0 | 88.2 | 88.6 | 88.3 | 88.3 | 88.3 | 88.1 | 99.3 | JQ086762.1_China_Mosquito |
|  | 16 | 2.7 | 2.9 | 13.2 | 3.0 | 3.0 | 13.0 | 13.1 | 13.0 | 4.0 | 13.3 | 13.0 | 1.7 | 2.7 | 13.0 | 2.7 |  | 99.9 | 97.1 | 96.8 | 88.5 | 88.5 | 97.1 | 97.0 | 97.5 | 87.7 | 88.4 | 95.2 | 88.4 | 84.2 | 88.3 | 88.3 | 88.9 | 88.3 | 88.1 | 88.4 | 88.7 | 88.4 | 88.3 | 88.4 | 88.3 | 97.7 | KC915016.1_China_Pig |
|  | 17 | 2.7 | 2.9 | 13.2 | 3.0 | 3.0 | 12.9 | 13.0 | 12.9 | 3.9 | 13.2 | 13.0 | 1.6 | 2.7 | 13.0 | 2.7 | 0.1 |  | 97.2 | 96.8 | 88.5 | 88.5 | 97.2 | 97.1 | 97.5 | 87.7 | 88.4 | 95.2 | 88.4 | 84.2 | 88.3 | 88.3 | 88.9 | 88.3 | 88.1 | 88.4 | 88.7 | 88.4 | 88.3 | 88.4 | 88.3 | 97.7 | KF297916.1_China_Pig |
|  | 18 | 0.9 | 2.4 | 13.2 | 2.5 | 2.4 | 12.8 | 12.8 | 12.7 | 3.3 | 13.2 | 13.0 | 1.6 | 2.0 | 13.0 | 2.0 | 2.9 | 2.9 |  | 98.5 | 88.6 | 88.6 | 97.7 | 97.6 | 98.2 | 87.8 | 88.4 | 96.4 | 88.4 | 84.3 | 88.4 | 88.3 | 89.0 | 88.4 | 88.1 | 88.4 | 88.9 | 88.4 | 88.5 | 88.4 | 88.3 | 98.3 | KF711994.1_South Korea_Pig |
|  | 19 | 1.3 | 2.6 | 13.4 | 2.7 | 2.7 | 13.0 | 12.9 | 13.0 | 3.5 | 13.4 | 13.2 | 2.0 | 2.3 | 13.2 | 2.3 | 3.3 | 3.2 | 1.5 |  | 88.4 | 88.4 | 97.5 | 97.3 | 97.9 | 87.7 | 88.3 | 96.3 | 88.3 | 84.4 | 88.3 | 88.2 | 88.9 | 88.3 | 88.0 | 88.3 | 88.7 | 88.3 | 88.4 | 88.2 | 88.1 | 98.0 | KP164498.2_India_Pig_Pig |
|  | 20 | 12.5 | 12.6 | 2.5 | 12.4 | 12.4 | 4.6 | 4.4 | 4.6 | 13.0 | 2.6 | 1.7 | 12.5 | 13.0 | 1.7 | 13.0 | 12.8 | 12.8 | 12.7 | 12.9 |  | 100.0 | 88.7 | 88.9 | 88.5 | 96.1 | 97.8 | 88.0 | 97.8 | 83.6 | 97.6 | 97.8 | 97.8 | 97.6 | 98.4 | 97.9 | 89.6 | 98.0 | 97.8 | 98.3 | 98.0 | 88.7 | KT229574.1_China_Mosquito |
|  | 21 | 12.5 | 12.6 | 2.5 | 12.4 | 12.4 | 4.7 | 4.4 | 4.6 | 13.0 | 2.7 | 1.7 | 12.5 | 13.0 | 1.7 | 13.0 | 12.8 | 12.8 | 12.8 | 13.0 | 0.0 |  | 88.7 | 88.9 | 88.5 | 96.1 | 97.8 | 88.0 | 97.8 | 83.6 | 97.5 | 97.7 | 97.8 | 97.6 | 98.3 | 97.9 | 89.6 | 98.0 | 97.8 | 98.3 | 98.0 | 88.7 | KT229575.1_China_Mosquito |
|  | 22 | 2.1 | 1.2 | 12.9 | 2.0 | 2.0 | 12.7 | 12.6 | 12.7 | 3.4 | 12.9 | 12.8 | 2.0 | 2.1 | 12.8 | 2.1 | 2.9 | 2.9 | 2.3 | 2.6 | 12.6 | 12.6 |  | 98.0 | 98.1 | 87.9 | 88.6 | 95.6 | 88.5 | 84.6 | 88.5 | 88.4 | 89.1 | 88.6 | 88.4 | 88.5 | 88.9 | 88.6 | 88.6 | 88.6 | 88.4 | 98.3 | KT447437.1_South Korea_Pig |
|  | 23 | 2.3 | 2.0 | 12.8 | 0.3 | 0.2 | 12.5 | 12.4 | 12.5 | 3.4 | 12.8 | 12.6 | 2.1 | 2.2 | 12.6 | 2.2 | 3.0 | 3.0 | 2.5 | 2.7 | 12.4 | 12.4 | 2.0 |  | 98.0 | 88.1 | 88.8 | 95.4 | 88.7 | 84.5 | 88.7 | 88.6 | 89.1 | 88.7 | 88.4 | 88.6 | 88.9 | 88.7 | 88.8 | 88.7 | 88.6 | 98.2 | KU363309.1_China_Pig |
|  | 24 | 1.7 | 2.0 | 13.2 | 2.1 | 2.0 | 12.8 | 12.8 | 12.8 | 3.0 | 13.2 | 13.0 | 1.5 | 0.4 | 13.1 | 0.4 | 2.6 | 2.6 | 1.9 | 2.2 | 12.8 | 12.8 | 1.9 | 2.1 |  | 87.7 | 88.4 | 95.9 | 88.4 | 84.3 | 88.3 | 88.3 | 89.0 | 88.4 | 88.2 | 88.3 | 88.7 | 88.4 | 88.4 | 88.4 | 88.2 | 99.4 | KX965684.1_China_Pig |
|  | 25 | 13.5 | 13.6 | 4.3 | 13.4 | 13.4 | 5.6 | 5.5 | 5.8 | 13.9 | 4.1 | 4.1 | 13.5 | 14.0 | 4.1 | 14.0 | 13.9 | 13.9 | 13.7 | 13.9 | 4.0 | 4.1 | 13.6 | 13.4 | 13.8 |  | 96.1 | 87.3 | 96.1 | 83.4 | 95.8 | 96.0 | 95.5 | 96.0 | 96.0 | 95.7 | 89.0 | 95.8 | 96.0 | 96.0 | 95.8 | 87.9 | KY927818.1_Cambodia_Pig |
|  | 26 | 12.7 | 12.8 | 2.5 | 12.6 | 12.6 | 4.6 | 4.4 | 4.6 | 13.2 | 2.7 | 2.3 | 12.6 | 13.1 | 2.3 | 13.1 | 13.0 | 13.0 | 13.0 | 13.0 | 2.3 | 2.3 | 12.7 | 12.5 | 12.9 | 4.0 |  | 87.9 | 99.5 | 83.4 | 98.5 | 99.4 | 97.2 | 98.5 | 97.7 | 97.3 | 89.4 | 97.5 | 98.7 | 97.7 | 97.4 | 88.6 | LC461957.1_Japan_Mosquito |
|  | 27 | 3.5 | 4.7 | 13.9 | 4.7 | 4.7 | 13.7 | 13.6 | 13.6 | 5.4 | 13.8 | 13.6 | 4.1 | 4.4 | 13.6 | 4.4 | 5.1 | 5.0 | 3.7 | 3.9 | 13.4 | 13.5 | 4.6 | 4.8 | 4.2 | 14.4 | 13.6 |  | 88.0 | 84.0 | 87.8 | 87.8 | 88.3 | 87.8 | 87.7 | 87.9 | 88.3 | 87.8 | 87.8 | 87.8 | 87.8 | 96.1 | LC461960.1_Japan_Pig |
|  | 28 | 12.8 | 12.8 | 2.4 | 12.6 | 12.6 | 4.5 | 4.3 | 4.4 | 13.2 | 2.7 | 2.2 | 12.7 | 13.1 | 2.2 | 13.1 | 13.0 | 13.0 | 13.0 | 13.1 | 2.2 | 2.2 | 12.8 | 12.5 | 13.0 | 4.0 | 0.5 | 13.5 |  | 83.5 | 98.5 | 99.5 | 97.3 | 98.6 | 97.8 | 97.4 | 89.4 | 97.6 | 98.7 | 97.8 | 97.5 | 88.6 | LC513838.1_Japan_Mosquito |
|  | 29 | 17.8 | 18.0 | 19.6 | 18.0 | 17.9 | 18.6 | 18.5 | 18.7 | 18.1 | 19.0 | 19.0 | 17.8 | 18.2 | 19.0 | 18.2 | 18.3 | 18.3 | 18.2 | 18.1 | 19.1 | 19.1 | 17.8 | 17.9 | 18.1 | 19.4 | 19.4 | 18.6 | 19.3 |  | 83.6 | 83.4 | 83.8 | 83.6 | 83.6 | 83.7 | 83.8 | 83.7 | 83.6 | 83.7 | 83.7 | 84.4 | LC579814.1_Indonesia_Mosquito |
|  | 30 | 12.8 | 12.8 | 2.7 | 12.6 | 12.7 | 4.9 | 4.7 | 4.8 | 13.2 | 3.1 | 2.6 | 12.7 | 13.1 | 2.6 | 13.1 | 13.1 | 13.1 | 13.0 | 13.1 | 2.5 | 2.5 | 12.8 | 12.6 | 13.0 | 4.3 | 1.6 | 13.7 | 1.5 | 19.1 |  | 98.4 | 97.0 | 98.6 | 97.4 | 97.1 | 89.3 | 97.2 | 98.8 | 97.5 | 97.1 | 88.5 | LC623822.1_Japan_Mosquito |
|  | 31 | 12.9 | 12.9 | 2.5 | 12.7 | 12.7 | 4.6 | 4.4 | 4.6 | 13.3 | 2.8 | 2.3 | 12.8 | 13.2 | 2.3 | 13.2 | 13.1 | 13.1 | 13.1 | 13.2 | 2.3 | 2.3 | 12.9 | 12.7 | 13.1 | 4.1 | 0.6 | 13.7 | 0.5 | 19.4 | 1.6 |  | 97.2 | 98.5 | 97.7 | 97.3 | 89.4 | 97.5 | 98.7 | 97.7 | 97.4 | 88.5 | LC708276._Japan_Pig |
|  | 32 | 12.0 | 12.1 | 3.1 | 12.1 | 12.2 | 5.3 | 5.0 | 5.1 | 12.6 | 3.4 | 2.2 | 12.0 | 12.4 | 2.2 | 12.4 | 12.4 | 12.4 | 12.2 | 12.3 | 2.3 | 2.3 | 12.1 | 12.1 | 12.3 | 4.7 | 2.9 | 13.1 | 2.8 | 18.9 | 3.1 | 2.9 |  | 97.1 | 97.8 | 98.4 | 89.4 | 98.2 | 97.3 | 98.8 | 98.5 | 89.1 | MH385014.1_China_Mosquito |
|  | 33 | 12.4 | 12.5 | 2.3 | 12.3 | 12.3 | 4.5 | 4.3 | 4.5 | 12.8 | 2.7 | 2.2 | 12.4 | 12.8 | 2.2 | 12.8 | 12.8 | 12.7 | 12.6 | 12.8 | 2.1 | 2.2 | 12.5 | 12.2 | 12.7 | 3.9 | 1.3 | 13.4 | 1.2 | 18.9 | 1.1 | 1.3 | 2.7 |  | 97.5 | 97.2 | 89.4 | 97.4 | 98.9 | 97.6 | 97.3 | 88.6 | MK558811.1_China_Mosquito |
|  | 34 | 13.1 | 13.1 | 2.6 | 13.0 | 13.0 | 4.8 | 4.5 | 4.8 | 13.4 | 2.8 | 1.7 | 13.0 | 13.4 | 1.8 | 13.4 | 13.3 | 13.3 | 13.3 | 13.4 | 1.7 | 1.7 | 13.0 | 12.9 | 13.3 | 4.1 | 2.4 | 13.8 | 2.3 | 19.2 | 2.6 | 2.4 | 2.3 | 2.3 |  | 97.9 | 89.4 | 98.0 | 97.6 | 98.3 | 98.0 | 88.3 | MN544780.1_China_Pig |
|  | 35 | 12.7 | 12.8 | 2.9 | 12.8 | 12.8 | 5.0 | 4.7 | 4.9 | 13.2 | 3.1 | 2.0 | 12.7 | 13.2 | 2.1 | 13.2 | 13.0 | 13.0 | 12.9 | 13.1 | 2.1 | 2.1 | 12.8 | 12.7 | 13.1 | 4.4 | 2.7 | 13.6 | 2.6 | 19.0 | 3.0 | 2.7 | 1.6 | 2.6 | 2.1 |  | 89.5 | 98.4 | 97.3 | 99.2 | 99.9 | 88.5 | MT232844.1_India_Pig |
|  | 36 | 12.2 | 12.2 | 12.0 | 12.2 | 12.2 | 11.4 | 11.3 | 11.2 | 13.1 | 11.6 | 11.5 | 12.1 | 12.6 | 11.5 | 12.6 | 12.6 | 12.5 | 12.4 | 12.5 | 11.5 | 11.5 | 12.3 | 12.3 | 12.5 | 12.2 | 11.7 | 13.0 | 11.7 | 18.9 | 11.9 | 11.8 | 11.7 | 11.4 | 11.7 | 11.6 |  | 89.5 | 89.5 | 89.5 | 89.5 | 88.9 | MT253737.1_Australia_Pig |
|  | 37 | 12.7 | 12.8 | 2.8 | 12.6 | 12.6 | 4.9 | 4.7 | 4.9 | 13.2 | 3.1 | 1.9 | 12.7 | 13.1 | 2.0 | 13.1 | 12.9 | 12.9 | 13.0 | 13.0 | 2.0 | 2.0 | 12.7 | 12.5 | 13.0 | 4.3 | 2.6 | 13.7 | 2.5 | 19.0 | 2.9 | 2.6 | 1.8 | 2.4 | 2.0 | 1.7 | 11.7 |  | 97.5 | 98.7 | 98.5 | 88.6 | MT254426.1_China_Mosquito |
|  | 38 | 12.7 | 12.8 | 2.5 | 12.6 | 12.6 | 4.6 | 4.3 | 4.5 | 13.2 | 2.8 | 2.4 | 12.7 | 13.1 | 2.4 | 13.1 | 13.0 | 13.0 | 12.9 | 13.0 | 2.3 | 2.3 | 12.7 | 12.5 | 13.0 | 4.1 | 1.3 | 13.7 | 1.3 | 19.1 | 1.2 | 1.3 | 2.8 | 0.8 | 2.4 | 2.8 | 11.6 | 2.6 |  | 97.7 | 97.4 | 88.6 | MT560941.1_China_Mosquito |
|  | 39 | 12.8 | 12.8 | 2.6 | 12.6 | 12.7 | 4.7 | 4.5 | 4.6 | 13.2 | 2.8 | 1.6 | 12.7 | 13.1 | 1.7 | 13.1 | 13.0 | 12.9 | 13.0 | 13.2 | 1.7 | 1.7 | 12.7 | 12.6 | 13.0 | 4.1 | 2.3 | 13.7 | 2.3 | 19.0 | 2.6 | 2.3 | 1.2 | 2.2 | 1.8 | 0.8 | 11.6 | 1.3 | 2.3 |  | 99.3 | 88.5 | MZ702743.1_India_Assam__Pig |
|  | 40 | 12.9 | 12.9 | 2.9 | 12.8 | 12.9 | 4.9 | 4.7 | 4.8 | 13.4 | 3.1 | 2.0 | 12.9 | 13.3 | 2.0 | 13.3 | 13.1 | 13.1 | 13.1 | 13.3 | 2.0 | 2.0 | 12.9 | 12.8 | 13.2 | 4.4 | 2.7 | 13.7 | 2.6 | 19.0 | 3.0 | 2.7 | 1.5 | 2.5 | 2.0 | 0.1 | 11.7 | 1.6 | 2.7 | 0.7 |  | 88.4 | ON875960_India_Assam_Mosquito |
|  | 41 | 1.5 | 1.8 | 12.9 | 1.9 | 1.8 | 12.6 | 12.6 | 12.6 | 2.7 | 12.9 | 12.8 | 1.3 | 0.7 | 12.9 | 0.7 | 2.4 | 2.4 | 1.7 | 2.0 | 12.6 | 12.6 | 1.7 | 1.9 | 0.6 | 13.6 | 12.7 | 4.0 | 12.8 | 18.0 | 12.8 | 12.9 | 12.1 | 12.5 | 13.0 | 12.9 | 12.4 | 12.7 | 12.7 | 12.8 | 13.0 |  | U15763.1_USA_Pig |
|  | 1 | 2 | 3 | 4 | 5 | 6 | 7 | 8 | 9 | 10 | 11 | 12 | 13 | 14 | 15 | 16 | 17 | 18 | 19 | 20 | 21 | 22 | 23 | 24 | 25 | 26 | 27 | 28 | 29 | 30 | 31 | 32 | 33 | 34 | 35 | 36 | 37 | 38 | 39 | 40 | 41 |  |  |
