# Supplementary material for: Japanese Encephalitis Virus Genotype III Strains Detection and Genome Sequencing from Indian Pig and Mosquito Vector
Source: Vaccines (Basel). 2023 Jan 10;11(1):150. doi: 10.3390/vaccines11010150 (PMC9862938; doi:10.3390/vaccines11010150)
Supplement: Supplementary file 1 [file vaccines-11-00150-s001.zip › vaccines-2082975-supplementary/Supplementary file 2.docx]

**Supplementary file 2: Alignments of amino acid sequences of JEV polyprotein gene (Isolated from Pig)**

**M T K K P G G P G K N R A I N M L K R G L P R V F P L V G V K R V V M S L L D G R G P V R F V L A L I T F F K F T A L A P T K A L L G R W K A V E K S V A M K H L T S F K R E L G T L I D A V N K R G R Majority**

**------------------+-------------------+-------------------+-------------------+-------------------+-------------------+-------------------+-------------------+-------------------+-------------------+-**

**10 20 30 40 50 60 70 80 90 100**

**------------------+-------------------+-------------------+-------------------+-------------------+-------------------+-------------------+-------------------+-------------------+-------------------+-**

**1 . . . . . . . . . . . . . . . . . . . . . . . . . . . . . . . . . . . . . . . . . . . . . . . . . . . . . . . . . . . . . . . . . . . . . . . . . . . . . . . . . . . . . . . . . . . . . . . . . . . . AB551990.1_Japan_Pig**

**1 . . . . . . . . . . . . . . . . . . . . . . . . . . . . . . . K . . . . . . . . . . . . . . . . . . . . . . . . . . . . . . . . . . . . . R . . . . . . . . . . . . . . . . . . . . . . . V . . . . . K AY316157.1_Korea_Pig**

**1 . . . . . . . . . . . . . . . . . . . . . . . . . . . . . . . . . . . . . . . . . . . . . . . . . . . . . . . . . . . . . . . . . . . . . . . . . . . . . . . . . . . . . . . . . . . . . . . . . . . . AY849939.1_China_Pig**

**1 . . . . . . . . . . . . . . . . . . . . . . . . . . . . . . . . . . . . . . . . . . . . . . . . . . . . . . . . . . . . . . . . . . . . . . . . . . . . . . . . . . . . . . . . . . . . . . . . . . . . EF107523.1_China_Pig**

**1 . . . . . . . . . . . . . . . . . . . . . . . . . . . . . . . . . . . . . . . . . . . . . . . . . . . . . . . . . . . . . . . . . . . . . R . . . . . . . . . . . . . . . . . . . . . . . . . . . . . K GQ902058.1_Thailand_Pig**

**1 . . . . . . . . . . . . . . . . . . . . . . . . . . . . . . . . . . . . . . . . . . . . . . . . . . V A . . . . . . . . . . . . . . . . . R . . . . . . . . . . . . . . . . . . . . . . . . . . . . . K GQ902061.1_Thailand_Pig**

**1 . . . . . . . . . . . . . . . . . . . . . . . . . . . . . . . . . . . . . . . . . . . . . . . . . . . . . . . . . . . . . . . . . . . . . . . . . . . . . . . . . . . . . . . . . . . . . . . . . . . . JN381872.1_China_Pig**

**1 . . . . . . . . . . . . . . . . . . . . . . . . . . . . . . . . . . . . . . . . . . . . . . . . . . . . . . . . . . . . . . . . . . . . . . . . . . . . . . . . . . . . . . . . . . . . . . . . . . . . KC915016.1_China_Pig**

**1 . . . . . . . . . . . . . . . . . . . . . . . . . . . . . . . . . . . . . . . . . . . . . . . . . . . . . . . . . . . . . . . . . . . . . . . . . . . . . . . . . . . . . . . . . . . . . . . . . . . . KF297916.1_China_Pig**

**1 . . . . . . . . . . . . . . . . . . . . . . . . L . . . . . . . . . . . . . . . . . . . . . . . . . . . . . . . . . . . . . . . . . . . . . . . Q . . . . . . . . . . . . . . . . . . . . . . . . . . . KF711994.1_South Korea_Pig**

**1 . . . . . . . . . . . . . . . . . . . . . . . . . . . . . . . . . . . . . . . . . . . . . . . . . . . . . . . . . . . . . . . . . . . . . . . . . . . . . . . . . . . . . . . . . . . . . . . . . . . . KP164498.2_India_Pig**

**1 . . . . . . . . . . . . . . . . . . . . . . . . . . . . . . . . . . . . . . . . . . . . . . . . . . . . . . . . . . . . . . . . . . . . . . . . . . . . . . . . . . . . . . . . . . . . . . . . . . . . KT447437.1_South Korea_Pig**

**1 . . . . . . . . . . . . . . . . . . . . . . . . . . . . . . . . . . . . . . . . . . . . . . . . . . . . . . . . . . . . . . . . . . . . . . . . . . . . . . . . . . . . . . . . . . . . . . . . . . . . KU363309.1_China_Pig**

**1 . . . . . . . . . . . . . . . . . . . . . . . . . . . . . . . . . . . . . . . . . . . . . . . . . . . . . . . . . . . . . . . . . S . . . . . . . . . . . . . . . . . . . . . . . . . . . . . . . . . . KX965684.1_China_Pig**

**1 . . . . . . . . . . . . . . . . . . . . . . . . . . . . . . . . . . . . . . . . . . . . . . . . . . . . . . . . . . . . . . . . . . . . . R . . . . . . . . . . . . . . . . . . . . . . . . . . . . . K KY927818.1_Cambodia_Pig**

**1 . . . . . . . . . . . . . . . . . . . . . . . . . . . . . . . . . . . . . . . . . . . . . . . . . . . . . . . . . . . . . . . . . . . . . . . . . . . . . . . . . . . . . . . . . . . . . . . . . . . . LC461960.1_Japan_Pig**

**1 . . . . . . . . . . . . . . . . . . . . . . . . . . . . . . . . . . . . . . . . . . . . . . . . . . . . . . . . . . . . . . . . . . . . . R . . . . . . . . . . . . . . . . . . . . . . . . . . . . . K LC708276._Japan_Pig**

**1 . . . . . . . . . . . . . . . . . . . . . . . . . . . . . . . . . . . . . . . . . . . . . . . . . . . . . . . . . . . . . . . . . . . . . R . . . . . . . . . . . . . . . . . . . . . . . . . . . . . K MN544780.1_China_Pig**

**1 . . . . . . . . . . . . . . . . . . . . . . . . . . . . . . . . . . . . . . . . . . . . . . . . . . . . . . . . . . . . . . . . . . . . . R . . . . . . . . . . . . . . . . . . . . . . . . . . . . . K MT232844.1_India_Pig**

**1 . . . . . . . . . . . . . . . . . . . . . . . . . . . . . . . . . . . . . . . . . . . A . . . . . . V . . . . . . . . . . . . . . . . . . R . . . . . . . . . . . . . . . . . . . I . . . . . . . . . K MT253737.1_Australia_Pig**

**1 . . . . . . . . . . . . . . . . . . . . . . . . . . . . . . . . . . . . . . . . . . . . . . . . . . . . . . . . . . . . . . . . . . . . . R . . . . . . . . . . . . . . . . . . . . . . . . . . . . . K MZ702743.1_India_Assam_Pig**

**1 . . . . . . . . . . . . . . . . . . . . . . . . . . . . . . . . . . . . . . . . . . . . . . . . . . . . . . . . . . . . . . . . . . . . . . . . . . . . . . . . . . . . . . . . . . . . . . . . . . . . U15763.1_USA_Pig**

**K Q N K R G G N E G S I M W L A S L A V V I A C A G A M K L S N F Q G K L L M T I N N T D I A D V I V I P T S K G E N R C W V R A I D V G Y M C E D T I T Y E C P K L T M G N D P E D V D C W C D N Q E Majority**

**------------------+-------------------+-------------------+-------------------+-------------------+-------------------+-------------------+-------------------+-------------------+-------------------+-**

**110 120 130 140 150 160 170 180 190 200**

**------------------+-------------------+-------------------+-------------------+-------------------+-------------------+-------------------+-------------------+-------------------+-------------------+-**

**301 . . . . . . . . . S . . . . . . . V . . . . . . . . . . . . . . . . . . . . . . . . . . . . . . . . . . . . . . . . . . . . . . . . . . . . . . . . . . . . . . . . . . . . . . . . . . . . . . . . . . AB551990.1_Japan_Pig**

**301 . . . . . . . . . S T . . . . . . . . I . T . . . . . . . . . . . . . . . . . . . . . . . . . . . . . . . . . . . . . . . . . . . . . . . . . . . . . . . . . . . . . A V . . . . . . . . . . . . . . . AY316157.1_Korea_Pig**

**301 . . . . . . . . . . . . . . . . . . . A . . . . . . . . R . . . . . . . . . . . . . . . . . . . . . . . . . . . . . . . . . . . . . . . . . . . . . . . . . . . . . . . . . . . . . . . . . . . . . . . AY849939.1_China_Pig**

**301 . . . . . . . . . . . . . . . . . . . A . . . . . . . . R . . . . . . . . . . . . . . . . . . . . . . . . . . . . . . . . . . . . . . . . . . . . . . . . . . . . . . . . . . . . . . . . . . . . . . . EF107523.1_China_Pig**

**301 . . . . . . . . . S . . . . . . . . . I . A . . . . . . . . . . . . . . . . . . . . . . . . . . . . . . . . . . . . . . . . . . . . . . . . . . . . . . . . . . . . . A V . . . . . . . . . . . . . . . GQ902058.1_Thailand_Pig**

**301 . . . . . . . . . S . . V . . . . . . I . T . . . . . . . . . . . . . . . . . . . . . . . . . . . . . . . . . . . . . . . . . . . . . . . . . . . . . . . . . . . . . A V . . . . . . . . . . . . . . . GQ902061.1_Thailand_Pig**

**301 . . . . . . . . . . . . . . . . . . . . . . . . V . . . . . . . . . . . . . . . . . . . . . . . . . . . . . . . . . . . . . . . . . . . . . . . . . . . . . . . . . . . . . . . . . . . . . . . . . . . JN381872.1_China_Pig**

**301 . . . . . . . . . . . . . . . . . . . . . V . . . . . . . . . . . . . . . . . . . . . . . . . . . . . . . . . . . . . . . . . . . . . . . . . . K . . . A . . . . . . . . . . . . . . . . . . . . . . . KC915016.1_China_Pig**

**301 . . . . . . . . . . . . . . . T . . . . . V . . . . . . . . . . . . . . . . . . . . . . . . . . . . . . . . . . . . . . . . . . . . . . . . . . K . . . . . . . . . . . . . . . . . . . . . . . . . . . KF297916.1_China_Pig**

**301 . . . . . . . . . . . . . . . . . . . . . . . . . . . . R . . . . . . . . . . . . . . . . V . . . . . . . . . . . . . . . . . . . . . . . . . . . . . . . . . . . . . . . . . . . . . . . . . . . . . . KF711994.1_South Korea_Pig**

**301 . . . . . . . . . . . . . . . . . . . . . . . Y . . . . . . . . . . . . . . . . . . . . . . . . . . . . . . . . . . . . . . . . . . . . . . . . . . . . . . . . . . . . . . . . . . . . . . . . . . . . KP164498.2_India_Pig**

**301 . . . . . . . . . . . . . . . . . . . . . . . . . . . . . . . . . . . . . . . . . . . . . . . . . . . . . . . . . . . . . . . . . . . . . . . . . . . . . . . . . . . . . . . . . . . . . . . . . . . . KT447437.1_South Korea_Pig**

**301 . . . . . . . . . . . . . . . . . . . A . . . . . . . . R . . . . . . . . . . . . . . . . . . . . . . . . . . . . . . . . . . . . . . . . . . . . . . . . . . . . . . . . . . . . . . . . . . . . . . . KU363309.1_China_Pig**

**301 . . . . . . . . . . . . . . . . . . . . . . . . . . . . . . . . . . . . . . . . . . . . . . . . . . . . . . . . . . . . . . . . . . . . . . . . . . . . . . . . . . . . . . . . . . . . . . . . . . . . KX965684.1_China_Pig**

**301 . . . . . . . . . S . . . . . . . . . I . T . . . . . . . . . . . . . . . . . . . . . . . . . . . . . . . . . . . . . . . . . . . . . . . . . . . . . . . . . . . . . A V . . . . . . . . . . . . . . . KY927818.1_Cambodia_Pig**

**301 . . . . . . . . . . . . . . . . . . . . . . . . . . . . . . . . . . . . . . . . . . . . . . . . . . . . . . . . . . . . . . . . . . . . . H . . . . . . . . . . . . . . . . . . . . . . . . . . . . . . LC461960.1_Japan_Pig**

**301 . . . . . . . . . S . . . . . . . . . I . T . . . . . . . . . . . . . . . . . A . . . . . . . . . . . . . . . . . . . . . . . . . . . . . . . . . . . . . . . . . . . A V . . . . . . . . . . . . . . . LC708276._Japan_Pig**

**301 . . . . . R . . . S L . . . . G . . . I . T . W . . T . . . . . . . . . . . . . . . . . . . . . . L L . . . . . . . . . . . . P T . H . . . . . . . . . . . . W . . . G V . . . . . . . . . . . . . . . MN544780.1_China_Pig**

**301 . . . . . . . . . S . . . . . . . . . I . . . . . . . . . . . . . . . . . . . . . . . . . . . . . . . . . . . . . . . . . . . . . . . . . . . . . . . . . . . . . . . A V . . . . . . . . . . . . . . . MT232844.1_India_Pig**

**301 . . . . . . . . . . . . . . . . . . . . I M . . . . . . . . . . . . . . . . . . V . . . . . . . . . . . . . . . . . . . . . . . . . . . . . . . . . . . . . . . . . . . T . . . . . . . . . . . . . . . MT253737.1_Australia_Pig**

**301 . . . . . . . . . S . . . . . . . . . I . . . . . . . . . . . . . . . . . . . . . . . . . . . . . . . . . . . . . . . . . . . . . . . . . . . . . . . . . . . . . . . A V . . . . . . . . . . . . . . . MZ702743.1_India_Assam_Pig**

**301 . . . . . . . . . . . . . . . . . . . . . . . . . . . . . . . . . . . . . . . . . . . . . . . . . . . . . . . . . . . . . . . . . . . . . . . . . . . . . . . . . . . . . . . . . . . . . . . . . . . . U15763.1_USA_Pig**

**V Y V Q Y G R C T R T R H S K R S R R S V S V Q T H G E S S L V N K K E A W L D S T K A T R Y L M K T E N W I I R N P G Y A F L A A A L G W M L G S N N G Q R V V F T I L L L L V A P A Y S F N C L G M Majority**

**------------------+-------------------+-------------------+-------------------+-------------------+-------------------+-------------------+-------------------+-------------------+-------------------+-**

**210 220 230 240 250 260 270 280 290 300**

**------------------+-------------------+-------------------+-------------------+-------------------+-------------------+-------------------+-------------------+-------------------+-------------------+-**

**601 . . . . . . . . . . . . . . . . . . . . . . . . . . . . . . . . . . . . . . . . . . . . . . . . . . . . . . . . . . . . . . . . . . . . . . . . . . . . . . . . . . . . . . . . . . . . . . . . . . . . AB551990.1_Japan_Pig**

**601 . . . . . . . . . . . . . . . . . . . . . . . . . . . . . . . . . . . K . . . . . . R . . . . . . . . . . . . . . . . . . . . . . . . . . . . . . . . S . . . . . . . . . . . . . . . . . . . . . . . . AY316157.1_Korea_Pig**

**601 . . . . . . . . . . . . . . . . . . . . . . . . . . . . . . . . . . . . . . . . . . . . . . . . . . . . . . . . . . . . . . . . . . V . . . . . . . . . . . . . . . . . . . . . . . . . . . . . . . . . AY849939.1_China_Pig**

**601 . . . . . . . . . . . . . . . . . . . . . . . . . . . . . . . . . . . . . . . . . . . . . . . . . . . . . . . . . . . . . . . . . . V . . . . . . . . . . . . . . . . . . . . . . . . . . . . . . . . . EF107523.1_China_Pig**

**601 . F . . . . . . . . . . . . . . . . . . . . . . . . . . . . . . . . . . . . . . . . . . . . . . . . . . . . . . . . . . . . . . . . . . . . . . . . . S . . . . . . . . . . . . . . . . . . . . . . . . GQ902058.1_Thailand_Pig**

**601 . F . . . . . . . . . . . . . . . . . . . . . . . . . . . . . . . . . . . . . . . . . . . . . . . . . . . . . . . . . . . . . . . . . . . . . . . . . S . . . . . . . . . . . . . . . . . . . . . . . . GQ902061.1_Thailand_Pig**

**601 . . . . . . . . . . . . . . . . G . . . . . . . . . . . . . . . . . . . . . . . . . . . . . . . . . . . . . . . . . . . . . . . . . V . . . . . . . . . . . . . . . . . . . . . . . . . . . L . . . . . JN381872.1_China_Pig**

**601 . . . . . . . . . . . . . . . . . . . . . . . . . . . . . . . . . . . . . . . . . . . . . . . . . . . . . . . . . . . . . . . . . . V . . . . . . . . . . . . . . . . . . . . . . . . . . . . . . . . . KC915016.1_China_Pig**

**601 . . . . . . . . . . . . . . . . . . . . . . . . . . . . . . . . . . . . . . . . . . . . . . . . . . . . . . . . . . . . . . . . . . V . . . . . . . . . . . . . . . . . . . . . . . . . . . . . . . . . KF297916.1_China_Pig**

**601 . . . . . . . . . . . . . . . . . . . . . . . . . . . . . . . . . . . . . . . . . . . . . . . . . . . . . . . . . . . . . . . . . . . . . . . . . . . . . . . . . . . . . . . . . . . . . . . . . . . . KF711994.1_South Korea_Pig**

**601 . . . . . . . . . . . . . . . . . . . . . . . . . . . . . . . . . . . . . . . . . . . . . . . . . . . . . . . . . . . . . . . . . . T . E . . . . R . . . . . . . . . . . . . . . . . . . . . . . . . . KP164498.2_India_Pig**

**601 . . . . . . . . . . . . . . . . . . . . . . . . . . . . . . . . . . . . . . . . . . . . . . . . . . . . . . . . . . . . . . . . . . V . . . . . . . . . . . . . . . . . . . . . . . . . . . . . . . . . KT447437.1_South Korea_Pig**

**601 . . . . . . . . . . . . . . . . . . . . . . . . . . . . . . . . . . . . . . . . . . . . . . . . . . . . . . . . . . . . . . . . . . V . . . . . . . . . . . . . . . . . . . . . . . . . . . . . . . . . KU363309.1_China_Pig**

**601 . . . . . . . . . . . . . . . . . . . . . . . . . . . . . . . . . . . . . . . . . . . . . . . . V . . . . . . . . . . . . . . . . . V . . . . . . . . . . . . . . . . . . . . . . . . . . . . . . . . . KX965684.1_China_Pig**

**601 . . . . . . . . . . . . . . . . . . . . I . . . . . . . . . . . . . . . . . . . . . . . . . . . . . . . . . . . . . . . . . . . . . . . . . . . . . . . . . . . . . . . . . . . . . . . . . . . . . . . KY927818.1_Cambodia_Pig**

**601 . . . . . . . . . . . . . . . . . . . . . . . . . . . . . . . . . . . . . . . . . . . . . . . . . . . . . . . . . . . . . . . . . . . . . . . . . . . . . . . . . . . . . . . . . . . . . . . . . . . . LC461960.1_Japan_Pig**

**601 . . . . . . . . . . . . . . . . . . . . . . . . . . . . . . . . . . . . . . . . . . . . . . . . . . . . . . . . . . . . . . . . . . . . . . . . . . . S . . . . . . . . . . . . . . . . . . . . . . . . LC708276._Japan_Pig**

**601 . . . . . . . . . . . . . . . . . . . . . . . . . . . . . . . . . . . . . . . . . . . . . . . . . . . . . . . . . . . . . . . . . V . . . . . . . . . . . . . . . . . . . . . . . . . . . . . . . . . . MN544780.1_China_Pig**

**601 . . . . . . . . . . . . . . . . . . . . . . . . . . . . . . . . . . . . . . . . . . . . . . . . . . . . . . . . . . . . . . . . . . . . . . . . . . . . . . . . . . . . . . . . . . . . . . . . . . . . MT232844.1_India_Pig**

**601 . . I . . . . . . . . . . . . . . . . . . . . . . . . . . . . . . . . . . . . . . . . . . . . . . . . . . . . . . . . . . . . . . . . . . . . . . . . . . . . . . . . . . . . . . . . . . . . . . . . . MT253737.1_Australia_Pig**

**601 . . . . . . . . . . . . . . . . . . . . . . . . . . . . . . . . . . . . . . . . . . . . . . . . . . . . . . . . . . . . . . . . . . . . . . . . . . . . . . . . . . . . . . . . . . . . . . . . . . . . MZ702743.1_India_Assam_Pig**

**601 . . . . . . . . . . . . . . . . . . . . . . . . . . . . . . . . . . . . . . . . . . . . . . . . . . . . . . . . . . . . . . . . . . V . . . . . . . . . . . . . . . . . . . . . . . . . . . . . . . . . U15763.1_USA_Pig**

**G N R D F I E G A S G A T W V D L V L E G D S C L T I M A N D K P T L D V R M I N I E A S Q L A E V R S Y C Y H A S V T D I S T V A R C P T T G E A H N E K R A D S S Y V C K Q G F T D R G W G N G C G Majority**

**------------------+-------------------+-------------------+-------------------+-------------------+-------------------+-------------------+-------------------+-------------------+-------------------+-**

**310 320 330 340 350 360 370 380 390 400**

**------------------+-------------------+-------------------+-------------------+-------------------+-------------------+-------------------+-------------------+-------------------+-------------------+-**

**901 . . . . . . . . . . . . . . . . . . . . . . . . . . . . . . . . . . . . . . . . . . . . . . . . . . . . . . . . . . . . . . . . . . . . . . . . . . . . . . . . . . . . . . . . . . . . . . . . . . . . AB551990.1_Japan_Pig**

**901 . . . . . . . . . . . . . . . . . . . . . . . . . . . . . . . . . . . . . . . . . . . . . . . . . . . . . . . . . . . . . . . . . . . . . . . . . . . . . . . . . . . . . . . . . . . . . . . . . . . . AY316157.1_Korea_Pig**

**901 . . . . . . . . . . . . . . . . . . . . . . . . . . . . . . . . . . . . . . . . . . . . . . . . . . . . . . . . . . . . . . . . . . . . . M . . . . . . . . . . . . . . . . . . . . . . . . . . . . . . AY849939.1_China_Pig**

**901 . . . . . . . . . . . . . . . . . . . . . . . . . . . . . . . . . . . . . . . . . . . . . . . . . . . . . . . . . . . . . . . . . . . . . M . . . . . . . . . . . . . . . . . . . . . . . . . . . . . . EF107523.1_China_Pig**

**901 . . . . . . . . . . . . . . . . . . . . . . . . . . . . . . . . . . . . . . . . . . . . . . . . . . . . . . . . . . . . . . . . . . . . . . . . . . . . . . . . . . N . . . . . . . . . . . . . . . . . GQ902058.1_Thailand_Pig**

**901 . . . . . . . . . . . . . . . . . . . . . . . . . . . . . . . . . . . . . . . . . . . . . . . . . . . . . . . . . . . . . . . . . . . . . . . . . . . . . . . . . . . . . . . . . . . . . . . . . . . . GQ902061.1_Thailand_Pig**

**901 . . . . . . . . . . . . . . . . . . . . . . . . . . . . . . . . . . . . . . . . . . . . . . . . . . . . . . . . . . . . . . . . . . . . . . . . . . . . . . . . . . . . . . . . . . . . . . . . . . . . JN381872.1_China_Pig**

**901 . . . . . . . . . . . . . . . . . . . . . . . . . . . . . . . . . . . . . . . . . . . . . . . . . . . . . . . . . . . . . . . . . . . . . . . . . . . . . . . . . . . . . . . . . . . . . . . . . . . . KC915016.1_China_Pig**

**901 . . . . . . . . . . . . . . . . . . . . . . . . . . . . . . E . . . . . . . . . . . . . . . . . . . . . . . . . . . . . . . . . . . . . . . . . . . . . . . . . . . . . . . . . . . . . . . . . . . . . KF297916.1_China_Pig**

**901 . . . . . . . . . . . . . . . . . . . . . . . . . . . . . . . . . . . . . . . . . . K . . . . . . . . . . . . . . . . . . . . . . . . . . . . . . . . . . . . . . . . . . . . . . . . . . . . . . . . . KF711994.1_South Korea_Pig**

**901 . . . . . . . . . . . . . . . . . . . . . . . . . . . . . . . . . . . . . . . . . . . . . . . . . . . . . . . . . . . . . . . . . . . . . . . . . . . . . . . . . . . . . . . . . . . . . . . . . . . . KP164498.2_India_Pig**

**901 . . . . . . . . . . . . . . . . . . . . . . . . . . . . . . . . . . . . . . . . . . . . . . . . . . . . . . . . . . . . . . . . . . . . . . . . . . . . . . . . . . . . . . . . . . . . . . . . . . . . KT447437.1_South Korea_Pig**

**901 . . . . . . . . . . . . . . . . . . . . . . . . . . . . . . . . . . . . . . . . . . . . . . . . . . . . . . . . . . . . . . . . . . . . . M . . . . . . . . . . . . . . . . . . . . . . . . . . . . . . KU363309.1_China_Pig**

**901 . . . . . . . . . . . . . . . . . . . . . . . . . . . . . . . . . . . . . . . . . . . . . . . . . . . . . . . . . . . . . . . . . . . . . . . . . . . . . . . . . . . . . . . . . . . . . . . . . . . . KX965684.1_China_Pig**

**901 . . . . . . . . . . . . . . . . . . . . . . . . . . . . . . . . . . . . . . . . . . . . . . . . . . . . . . . . . . . . . . . . . . . . . . . . . . . . D . . . . . N . . . . . . . . . . . . . . . . . KY927818.1_Cambodia_Pig**

**901 . . . . . . . . . . . . . . . . . . . . . . . . . . . . . . . . . . . . . . . . . . . . . . . . . . . . . . . . . . . . . . . . . . . . . . . . . . . . . . . . . . . . . . . . . . . . . . . . . . . . LC461960.1_Japan_Pig**

**901 . . . . . . . . T . . . . . . . . . . . . . . . . . . . . . . . . . . . . . . . . . . . . . . . . . . . . . . . . . . . . . . . . . . . . . . . . . . . . . . . . . . . . . . . . . . . . . . . . . . . LC708276._Japan_Pig**

**901 . . . . . . . . . . . . . . . . . . . . . . . . . . . . . . . . . . . . . . . . . . . . . . . . . . . . . . . . . . . . . . . . . . . . . . . . . . . . . . . . . . . . . . . . . . . . . . . . . . . . MN544780.1_China_Pig**

**901 . . . . . . . . . . . . . . . . . . . . . . . . . . . . . . . . . . . . . . . . . . . . . . . . . . . . . . . . . . . . . . . . . . . . . . . . . . . . . . . . . . . . . . . . . . . . . . . . . . . . MT232844.1_India_Pig**

**901 . . . . . . . . . . . . . . . . . . . . . . . . . . . . . . . . . . . . . . . . . . . . . . . . . . . . . . . . . . . . . . . . . . . . . . . . . . . . . . . . . . . . . . . . . . . . . . . . . . . . MT253737.1_Australia_Pig**

**901 . . . . . . . . . . . . . . . . . . . . . . . . . . . . . . . . . . . . . . . . . . . . . . . . . . . . . . . . . . . . . . . . . . . . . . . . . . . . . . . . . . . . . . . . . . . . . . . . . . . . MZ702743.1_India_Assam_Pig**

**901 . . . . . . . . . . . . . . . . . . . . . . . . . . . . . . . . . . . . . . . . . . . . . . . . . . . . . . . . . . . . . . . . . . . . . . . . . . . . . . . . . . . . . . . . . . . . . . . . . . . . U15763.1_USA_Pig**

**L F G K G S I D T C A K F S C T S K A I G R T I Q P E N I K Y E V G I F V H G T T T S E N H G N Y S A Q V G A S Q A A K F T V T P N A P S I T L K L G D Y G E V T L D C E P R S G L N T E A F Y V M T V Majority**

**------------------+-------------------+-------------------+-------------------+-------------------+-------------------+-------------------+-------------------+-------------------+-------------------+-**

**410 420 430 440 450 460 470 480 490 500**

**------------------+-------------------+-------------------+-------------------+-------------------+-------------------+-------------------+-------------------+-------------------+-------------------+-**

**1201 . . . . . . . . . . . . . . . . . . . . . . . . . . . . . . . . . . . . . . . . . . . . . . . . . . . . . . V . . . . . . . . . . . . . . . . . . . . . . . . . . . . . . . . . . . . . . . . . . . . . AB551990.1_Japan_Pig**

**1201 . . . . . . . . . . . . . . . . . . . . . . M . . S . . . . . . . . . . . . . . . . . . . . . . . . . . . . . . . . . . . . . . . . . . . . . . . . . . . . . . . . . . . . . . . . . . . . . . . . . . AY316157.1_Korea_Pig**

**1201 . . . . . . . . . . . . . . . . . . . . . . . . . . . . . . . . . . . . . . . . . . . . . . . . . . . . . . . . . . . . . . . . . . . . . . . . . . . . . . . . . . . . . . . . . . . . . . . . . . . . AY849939.1_China_Pig**

**1201 . . . . . . . . . . . . . . . . . . . . . . . . . . . . . . . . . . . . . . . . . . . . . . . . . . . . . . . . . . . . . . . . . . . . . . . . . . . . . . . . . . . . . . . . . . . . . . . . . . . . EF107523.1_China_Pig**

**1201 . . . . . . . . . . . . . . . . . . . . . . . . . . . . . . . . . . V . . . . . . . . . . . . . . . . . . . . . . . . . . . . . . . . . . . . . . . . . . . . . . . . . . . . . . . . . . . . . . . . . GQ902058.1_Thailand_Pig**

**1201 . . . . . . . . . . . . . . . . . . . . . . . . . . . . . . . . . . V . . . . . . . . . . . . . . . . . . . . . . . . . . . . . . . . . . . . . . . . . . . . . . . . . . . . . . . . . . . . . . . . . GQ902061.1_Thailand_Pig**

**1201 . . . . . . . . . . . . . . . . . . . . . . . . . . . . . . . . . . . . . . . . . . . . . . . . . . . . . . . . . . . . . . . . . . . . . . . . . . . . . . . . . . . . . . . . . . . . . . . . . . . . JN381872.1_China_Pig**

**1201 . . . . . . . . . . . . . . . . . . . . . . . . . . . . T . . . . . . . . . . . . . . . . . . . . . . . . . . . . . . . . . . . . . . . . . . . . . . . . . . . . . . . . . . . . . . . . . . . . . . . KC915016.1_China_Pig**

**1201 . . . . . . . . . . . . . . . . . . . . . . . . . . . . . . . . . . . . . . . . . . . . . . . . . . . . . . . . . . . . . . . . . . . . . . . . . . . . . . . . . . . . . . . . . . . . . . . . . . . . KF297916.1_China_Pig**

**1201 . . . . . . . . . . . . . . . . . . . . . . . . . . . . . . . . . . . . . . . . . . . . . . . . . . . . . . . . . . . . . . . . . . . . . M . . . . . . . . . . . . . . . . . . . . . . . . . . . . . . KF711994.1_South Korea_Pig**

**1201 . . . . . . . . . . . . . . . . . . . . . . . . . . . . . . . . . . . . . . . . . . . . . . . . . . . . . . . . . . . . . . I . . . . . . . . . . . . . . . . . . . . . . . . . . . . . . . . . . . . . KP164498.2_India_Pig**

**1201 . . . . . . . . . . . . . . . . . . . . . . . . . . . . . . . K . . . . . . . . . . . . . . . . . . . . . . . . . . . . . . . . . . . . . T . . . . . . . . . . . . . . . . . . . . . . . . . . . . . . KT447437.1_South Korea_Pig**

**1201 . . . . . . . . . . . . . . . . . . . . . . . . . . . . . . . . . . . . . . . . . . . . . . . . . . . . . . . . . . . . . . . . . . . . . . . . . . . . . . . . . . . . . . . . . . . . . . . . . . . . KU363309.1_China_Pig**

**1201 F . . . . . . . . . . . . . . . . . . . . . . . . . . . . . . K . . . . . . . . . . . . . . . . . . . . . . . . . . . . . . . . . . . . . V A . . . . . . . . . . . . . . . . . . . . . . . . . . . . . KX965684.1_China_Pig**

**1201 . . . . . . . . . . . . . . . . N . . . . . M . . . . . . . . . . . . . . . . . . . . . . . . . . . V . . . . . . . . . . . . . . . . . . . . . . . . . . . . . . . . . . . . . . . . . . . . . . . . . KY927818.1_Cambodia_Pig**

**1201 . . . . . . . . . . . . . . . . . . . . . . . . . . . . . . . . . . . . . . . . . . . . . . . . . . . . . . . . . . . . . . . . . . . . . . . . . . . . . . . . . . . . . . . . . . . . . . . . . . . . LC461960.1_Japan_Pig**

**1201 . . . . . . . . . . . . . . . . N . . . . . M . . . . . . . . . . . . . . . . . . . . . . . . . . . . . . . . . . . . . . . . . . . . . . . . . . . . . . . . . . . . . . . . . . . . . . . . . . . . . LC708276._Japan_Pig**

**1201 . . . . . . . . . . . . . . . . . . . . . . M . . . . . . . . R . . . . . . . . . . . . . . . . . . . . . . . . . . . . . . . . . . . . . T . . . . . . . . . . . . . . . . . . . . . . . . . . . . . . MN544780.1_China_Pig**

**1201 . . . . . . . . . . . . . . . . N . . . . . M . . . . . . . . . . . . . . . . . . . . . . . . . . . . . . . . . . . . . . . . . . . . . . . . . . . . . . . . . . . . . . . . . . . . . . . . . . . . . MT232844.1_India_Pig**

**1201 . . . . . . . . . . . . . . . . . . . . . . . . . . . . . . . . . . . . . . . . . . . . . . . . . . . . . . . . . . . . . . . . . . . . . . . . . . . . . . . . . . . . . . . . . . . . . . . . . . . . MT253737.1_Australia_Pig**

**1201 . . . . . . . . . . . . . . . . N . . . . . M . . . . . . . . . . . . . . . . . . . . . . . . . . . . . . . . . . . . . . . . . . . . . . . . . . . . . . . . . . . . . . . . . . . . . . . . . . . . . MZ702743.1_India_Assam_Pig**

**1201 . . . . . . . . . . . . . . . . . . . T . K . . . . . . . . . K . . . . . . . . . . . . . . . . . . . . . . . . . . . . . . . . . . . . . V . . E . . . . . . . . . . . . . . . . . . . . . . . . . . . U15763.1_USA_Pig**

**G S K S F L V H R E W F H D L A L P W T S P S S T A W R N R E L L M E F E E A H A T K Q S V V A L G S Q E G G L H Q A L A G A I V V E Y S S S V K L T S G H L K C R L K M D K L A L K G T T Y G M C T E Majority**

**------------------+-------------------+-------------------+-------------------+-------------------+-------------------+-------------------+-------------------+-------------------+-------------------+-**

**510 520 530 540 550 560 570 580 590 600**

**------------------+-------------------+-------------------+-------------------+-------------------+-------------------+-------------------+-------------------+-------------------+-------------------+-**

**1501 . . . . . . . . . . . . . . . . . . . . . . . . . . . . . . . . . . . . . . . . . . . . . . . . . . . . . . . . . . . . . . . . . . . . . . . . . . . . . . . . . . . . . . . . . . . . . . . . . . . . AB551990.1_Japan_Pig**

**1501 . . . . . . . . . . . . . . . S . . . . . . . . . . . . . . . . . . . . . . . . . . . . . . . . . . . . . . . . . . . . . . . . . . . . . . . . . . . . . . . . . . . . . . . . . . . . . . . . . . . . AY316157.1_Korea_Pig**

**1501 . . . . . . . . . . . . . . . . . . . . P . . . . . . . . . . . . . . . . . . . . . . . . . . . . . . . . . . . . . . . . . . . . . . . . . . . . . . . . . . . . . . . . . . . . . . . . . . . . . . G AY849939.1_China_Pig**

**1501 . . . . . . . . . . . . . . . . . . . . P . . . . . . . . . . . . . . . . . . . . . . . . . . . . . . . . . . . . . . . . . . . . . . . . . . . . . . . . . . . . . . . . . . . . . . . . . . . . . . G EF107523.1_China_Pig**

**1501 . . . . . . . . . . . . . . . S . . . . . . . . . . . . . . . . . . . . . . . . . . . . . . . . . . . . . . . . . . . . . . . . . . . . . . . . . . . . . . . . . . . . . . . . . . . . . . . . . . . G GQ902058.1_Thailand_Pig**

**1501 . . . . . . . . . . . . . . . S . . . . . . . . . . . . . . . . . . . . . . . . . . . . . . . . . . . . . . . . . . . . . . . . . . . . . . . . . . . . . . . . . . . . . . . . . . . . . . . . . . . . GQ902061.1_Thailand_Pig**

**1501 . . . . . . . . . . . . . . . . . . . . . . . . . . . . . . . . . . . . . . . . . . . . . . . . . . . . . . . . . . . . . . . . . . . . . . . . . . . . . . . . . . . . . . . . . . . . . . . . . . . . JN381872.1_China_Pig**

**1501 . . . . . . . . . . . . . . . . . . . . . . . . . T . . . . . . . . . . . . . . . . . . . . . . . . . . . . . . . . . . . . . . . . . . . . . . . . . . . . . . . . . . . . . . . . . . . . . . . . . . KC915016.1_China_Pig**

**1501 . . . . . . . . . . . . . . . . . . . . . . . . . T . . . . . . . . . . . . . . . . . . . . . . . . . . . . . . . . . . . . . . . . . . . . . . . . . . . . . . . . . . . . . . . . . . . . . . . . . . KF297916.1_China_Pig**

**1501 . . R . . . . . . . . . . . . . . . . . . . . . . . . . . . . . . . . . . . . . . . . . . . . . . . . . . . . . . H . . . . . . . . D . . . . . . . . . . . . . . . . . . . . . . . . . . . . . . . . . KF711994.1_South Korea_Pig**

**1501 . . . . . . . . . . . . . . . . . . . . . . . . . . . . . . . . . . . . . . . . . . . . . . . . . . . . . . . . . . . . . . . . . . . . . . . . . . . . . . . . . . . . . . . . . . . . . . . . . . . . KP164498.2_India_Pig**

**1501 . . R . . . . . . . . . . . . . . . . . . . . . . . . . . . . . . . . . . . . . . . . . . . . . . . . . . . . . . . . . . . . . . . . . . . . . . . . . . . . . . . . . . . . . . . . . . . . . . . . . KT447437.1_South Korea_Pig**

**1501 . . R . . . . . . . . . . . . . . . . . P . . . . . . . . . . . . . . . . . . . . . . . . . . . . . . . . . . . . . . . . . . . . . . . . . . . . . . . . . . . . . . . . . . . . . . . . . . . . . . G KU363309.1_China_Pig**

**1501 . . . . . . . . . . . . . . . . . . . . . . . . . . . . . . . . . . . . . G . . . . . . . . . . . . . . . . . . . H . . . . . . . . . . . . . . . . . . . . . . . . . . . . . . . . . . . . . . . . . . KX965684.1_China_Pig**

**1501 . . . . . . . . . . . . . . . S . . . . . . . N . . . . . . . . . . . . . . . . . . . . . . . . . . . . . . . . . . . . . . . . . . . . . . . . . . . . . . . . . . . . . . . . . . . . . . . . . . . . KY927818.1_Cambodia_Pig**

**1501 . . . . . . . . . . . . . . . . . . . . . . . . . . . . . . . . . . . . . . . . . . . . . . . . . . . . . . . . . . . . . . . . . . . . . . . . . . . . . . . . . . . . . . . . . . . . . . . . . . . . LC461960.1_Japan_Pig**

**1501 . . . . . . . . . . . . . . . S . . . . . . . . . . . . . . . . . . . . . . . . . . . . . . . . . . . . . . . . . . . . . . . . . . . . . . . . . . . . . . . . . . . . . . . . . . . . . . . . . . . . LC708276._Japan_Pig**

**1501 . . . . . . . . . . . . . . . S . . . . . . . . . . . . . . . . . . . . . . . . . . . . . . . . . . . . . . . . . . . . . . . . . . . . . . . . . . . . . . . . . . . . . . . . . . . . . . . . . . . . MN544780.1_China_Pig**

**1501 . . . . . . . . . . . . . . . S . . . . . . . . . . . . . . . . . . . . . . . . . . . . . . . . . . . . . . . . . . . . . . . . . . . . . . . . . . . . . . . . . . . . . . . . . . . . . . . . . . . . MT232844.1_India_Pig**

**1501 . P . . . . . . . . . . . . . S . . . . . . . . . . . . . . . . . . . . . . . . . . . . . . . . . . . . . . . . . . . . . . . . . . . . . . . . . . . . . . . . . . . . . . . . . . . . . . . . . . . . MT253737.1_Australia_Pig**

**1501 . . . . . . . . . . . . . . . S . . . . . . . . . . . . . . . . . . . . . . . . . . . . . . . . . . . . . . . . . . . . . . . . . . . . . . . . . . . . . . . . . . . . . . . . . . . . . . . . . . . . MZ702743.1_India_Assam_Pig**

**1501 . . . . . . . . . . . . . . . . . . . . . . . . . . . . . . . . . . . . . G . . . . . . . . . . . . . . . . . . . . . . . . . . . . . . . . . . . . . . . . . . . . . . . . . . . . . . . . . . . . . . U15763.1_USA_Pig**

**K F S F A K N P A D T G H G T V V I E L S Y S G S D G P C K I P I V S V A S L N D M T P V G R L V T V N P F V A T S S A N S K V L V E M E P P F G D S Y I V V G R G D K Q I N H H W H K A G S T L G K A Majority**

**------------------+-------------------+-------------------+-------------------+-------------------+-------------------+-------------------+-------------------+-------------------+-------------------+-**

**610 620 630 640 650 660 670 680 690 700**

**------------------+-------------------+-------------------+-------------------+-------------------+-------------------+-------------------+-------------------+-------------------+-------------------+-**

**1801 . . . . . . . . . . . . . . . . . . . . . . . . . . . . . . . . . . . . . N . . . . . . . . . . . . . . . . . . . . . . . . . . . . . . . . . . . . . . . . . . . . . . . . . . . . . . . . . . . . . . AB551990.1_Japan_Pig**

**1801 . . . . . . . . . . . . . . . . . . . . T . . . . . . . . . . . . . . . . . . . . . . . . . . . . . . . . . . . . . . S . . . . . . . . . . . . . . . . . . . . . . . . . . . . . . . . . . . . . . . . AY316157.1_Korea_Pig**

**1801 . . . . . . . . . . . . . . . . . . . . . . . . . . . . . . . . . . . . . . . . . . . . . . . . . . . . . . . . . . . . . . . . . . . . . . . . . . . . . . . . . . . . . . . . . . . . . . . . . . . . AY849939.1_China_Pig**

**1801 . . . . . . . . . . . . . . . . . . . . . . . . . . . . . . . . . . . . . . . . . . . . . . . . . . . . . . . . . . . . . . . . . . . . . . . . . . . . . . . . . . . . . . . . . . . . . . . . . . . . EF107523.1_China_Pig**

**1801 . . . . . . . . . . . . . . . . . . . . T . . . . . . . . . . . . . . . . . . . . . . . . . . . . . . . . . . . . . . S . . . . . . . . . . . . . . . . . . . . . . . . . . . . . . . . . . . . . . . . GQ902058.1_Thailand_Pig**

**1801 . . . . . . . . . . . . . . . . . . . . T . . . . . . . . . . . . . . . . . . . . . . . . . . . . . . . . . . . . . . S . . . . . . . . . . . . . . . . . . . . . . . . . . . . . . . . . . . . . . . . GQ902061.1_Thailand_Pig**

**1801 . . . . . . . . . . . . . . . . . . . . . . . . . . . . . . . . . . . . . . . . . . . . . . . . . . . . . . . . . . . . . . . . . . . . . . . . . . . . . . . . . . . . . . . . . . . . . . . . . . . . JN381872.1_China_Pig**

**1801 . . . . . . . . . . . . . . . . . . . . . . . . . . . . . . . . . . . . . . . . . . . . . . . . . . . . . . . . . . . . . . . . . . . . . . . . . . . . . . . . . . . . . . . . . . . . . . . . . . . . KC915016.1_China_Pig**

**1801 . . . . . . . . . . . . . . . . . . . . . . . . . . . . . . . . . . . . . . . . . . . . . . . . . . . . . . . . . . . . . . . . . . . . . . . . . . . . . . . . . . . . . . . . . . . . . . . . . . . . KF297916.1_China_Pig**

**1801 . . . . . . . . . . . . . . . . . . . . . . . . . . . . . . . . . . . . . . . . . . . . . . . . . . . . . . . . . . . . . . . . . . . . . . . . . . . . . . . . . . . . . . . . . . . . . . . . . . . . KF711994.1_South Korea_Pig**

**1801 . . . . . . . . . . . . . . . . . . . . . . . . . . . . . . . . . . . . . . . . . . . . . . . . . . . . . . . . . . . . . . . . . . . . . . . . . . . . . . . . . . . . . . . . . . . . . . . . . . . . KP164498.2_India_Pig**

**1801 . . . . . . . . . . . . . . . . . . . . . . . . . . . . . . . . . . . . . . . . . . . . . . . . . . . . . . . . . . . . . . . . . . . . . . . . . . . . . . . . . . . . . . . . . . . . . . . . . . . . KT447437.1_South Korea_Pig**

**1801 . . . . . . . . . . . . . . . . . . . . . . . . . . . . . . . . . . . . . . . . . . . . . . . . . . . . . . . . . . . . . . . . . . . . . . . . . . . . . . . . . . . . . . . . . . . . . . . . . . . . KU363309.1_China_Pig**

**1801 . . . . . . . . V . . . . . . . . . . . . . . . . . . . . . . . . . . . . . . . . . . . . . . . . . . . . . . . . . . . . . . . . . . . . . . . . . . . . . . . . . . . . . . . . . . . . . . . . . . . KX965684.1_China_Pig**

**1801 . . . . . . . . . . . . . . . . . . . . T . . . . . . . . . . . . . . . . . . . . . . . . . . . . . . . . . . . . . . S . . . . . . . . . . . . . . . . . . . . . . . . . . . . . . . . . . . . . . . . KY927818.1_Cambodia_Pig**

**1801 . . . . . . . . . . . . . . . . . . . . . . . . . . . . . . . . . . . . . . . . . . . . . . . . . . . . . . . . . . . . . . . . . . . . . . . . . . . . . . . . . . . . . . . . . . . . . . . . . . . . LC461960.1_Japan_Pig**

**1801 . . . . . . . . . . . . . . . . . . . . T . . . . . . . . . . . . . . . . . . . . . . . . . . . . . . . . . . . . . . S . . . . . . . . . . . . . . . . . . . . . . . . . . . . . . . . . . . . . . . . LC708276._Japan_Pig**

**1801 . . . . . . . . . . . . . . . . . . . . T . . . . . . . . . . . . . . . . . . . . . . . . . . . . . . . . . . . . . . S . . . . . . . . . . . . . . . . . . . . . . . . . . . . . . . . . . . . . . . . MN544780.1_China_Pig**

**1801 . . . . . . . . . . . . . . . . . . . . T . . . . . . . . . . . . . . . . . . . . . . . . . . . . . . . . . . . . . . S . . . . . . . . . . . . . . . . . . . . . . . . . . . . . . . . . . . . . . . . MT232844.1_India_Pig**

**1801 . . . . . . . . . . . . . . . . . . . . T . . . . . . . . . . . . . . . . . . . . . . . . . . . . . . . . . . . . . . . . . . . . . . . . . . . . . . . . . . . . . . . . . . . . . . . . . . . . . . . MT253737.1_Australia_Pig**

**1801 . . . . . . . . . . . . . . . . . . . P T . . . . . . . . . . . . A . . . . . . . . . . . . . . . . . . . . . . . . . S . . . . . . . . . . . . . . . . . . . . . . . . . . . . . . . . . . . . . . . . MZ702743.1_India_Assam_Pig**

**1801 . . . . . . . . V . . . . . . . . . . . . . . . . . . . . . . . . . . . . . . . . . . . . . . . . . . . . . . . . . . . . . . . . . . . . . . . . . . . . . . . . . . . . . . . . . . . . . . . . . . . U15763.1_USA_Pig**

**F S T T L K G A Q R L A A L G D T A W D F G S I G G V F N S I G K A V H Q V F G G A F R T L F G G M S W I T Q G L M G A L L L W M G V N A R D R S I A L A F L A T G G V L V F L A T N V H A D T G C A I Majority**

**------------------+-------------------+-------------------+-------------------+-------------------+-------------------+-------------------+-------------------+-------------------+-------------------+-**

**710 720 730 740 750 760 770 780 790 800**

**------------------+-------------------+-------------------+-------------------+-------------------+-------------------+-------------------+-------------------+-------------------+-------------------+-**

**2101 . . . . . . . . . . . . . . . . . . . . . . . . . . . . . . . . . . . . . . . . . . . . . . . . . . . . . . . . . . . . . . . . . . . . . . . . . . . . . . . . . . . . . . . . . . . . . . . . . . . . AB551990.1_Japan_Pig**

**2101 . . . . . . . . . . . . . . . . . . . . . . . . . . . . . . . . . . . . . . . . . . . . . . . . . . . . . . . . . . . . . . . . . . . . . . . . . . . . . . . . . . . . . . . . . . . . . . . . . . . . AY316157.1_Korea_Pig**

**2101 . L . . . . . . . . . . . . . . . . . . . . . . . . . . . . . . . . . . . . . . . . . . . . . . . . . . . . . . . . . . . . . . . . . . . . . . . . . . . . . . . . . . . . . . . . . . . . . . . . . . AY849939.1_China_Pig**

**2101 . L . . . . . . . . . . . . . . . . . . . . . . . . . . . . . . . . . . . . . . . . . . . . . . . . . . . . . . . . . . . . . . . . . . . . . . . . . . . . . . . . . . . . . . . . . . . . . . . . . . EF107523.1_China_Pig**

**2101 . . . . . . . . . . . . . . . . . . . . . . . . . . . . . . . . . . . . . . . . . . . . . . . . . . . . . . . . . . . . . . . . . . . . . . . . . . . . . . . . . . . . . . . . . . . . . . . . . . . . GQ902058.1_Thailand_Pig**

**2101 . . . . . . . . . . . . . . . . . . . . . . . . . . . . . . . . . . . . . . . . . . . . . . . . . . . . . . . . . . . . . . . . . . . . . . . . . . . . . . . . . . . . . . . . . . . . . . . . . . . . GQ902061.1_Thailand_Pig**

**2101 . . . . . . . . . . . . . . . . . . . . . . . . . . . . . . . . . . . . . . . . . . . . . . . . . . . . . . . . . . . . . . . . . . . . . . . . . . . . . . . . . . . . . . . . . . . . . . . . . . . . JN381872.1_China_Pig**

**2101 . . . . . . . . . . . . . . . . . . . . . . . . . . . . . . . . . . . . . . . . . . . . . . . . . . . . . . . . . . . . . . . . . . . . . . . . . . . . . . . . . . . . . . . . . . . . . . . . . . . . KC915016.1_China_Pig**

**2101 . . . . . . . . . . . . . . . . . . . . . . . . . . . . . . . . . . . . . . . . . . . . . . . . . . . . . . . . . . . . . . . . . . . . . . . . . . . . . . . . . . . . . . . . . . . . . . . . . . . . KF297916.1_China_Pig**

**2101 . . . . . . . . . . . . . . . . . . . . . . . . . . . . . . . . . . . . . . . . . . . . . . . . . . . . . . . . . . . . . . . . . . . . . . . . . . . . . . . . . . . . . . . . . . . . . . . . . . . . KF711994.1_South Korea_Pig**

**2101 . . . . . . . . . . . . . . . . . . . . . . . . . . . . . . . . . . . . . . . . . . . . . . . . . . . . . . . . . . . . . . . . . . . . . . . . . . . . . . . . . . . . . . . . . . . . . . . . . . . . KP164498.2_India_Pig**

**2101 . . . . . . . . . . . . . . . . . . . . . . . . . . . . . . . . . . . . . . . . . . . . . . . . . . . . . . . . . . . . . . . . . . . . . . . . . . . . . . . . . . . . . . . . . . . . . . . . . . . . KT447437.1_South Korea_Pig**

**2101 . L . . . . . . . . . . . . . . . . . . . . . . . . . . . . . . . . . . . . . . . . . . . . . . . . . . . . . . . . . . . . . . . . . . . . . . . . . . . . . . . . . . . . . . . . . . . . . . . . . . KU363309.1_China_Pig**

**2101 . . . . . . . . . . . . . . . . . . . . . . . . . . . . . . . . R . . . . . . . . . . . . . . . . . . . . . . . . . . . . . . . . . . . . . . . . . . . . . . . . . . . . . . . . . . . . . . . . . . . KX965684.1_China_Pig**

**2101 . . . . . . . . . . . . . . . . . . . . . . . V . . . . . . . . . . . . . . . . . . . . . . . . . . . . . . . . . . . . . . . . . . I . . . . . . . . . . . . . . . . . . . . . . . . . . . . . . . . . KY927818.1_Cambodia_Pig**

**2101 . . . . . . . . . . . . . . . . . . . . . . . . . . . . . . . . . . . . . . . . . . . . . . . . . . . . . . . . . . . . . . . . . . . . . . . . . . . . . . . . . . . . . . . . . . . . . . . . . . . . LC461960.1_Japan_Pig**

**2101 . . . . . . . . . . . . . . . . . . . . . . . . . . . . . . . . . . . . . . . . . . . . . . . . . . . . . . . . . . . . . . . . . . . . . . . . . . . . . . . . . . . . . . . . . . . . . . . . . . . . LC708276._Japan_Pig**

**2101 . . . . . . . . . . . . . . . . . . . . . . . . . . . . . . . . . . . . . . . . . . . . . . . . . . . . . . . . . . . . . . . . . . . . . . . . . . . . . . . . . . . . . . . . . . . . . . . . . . . . MN544780.1_China_Pig**

**2101 . . . . . . . . . . . . . . . . . . . . . . . . . . . . . . . . . . . . . . . . . . . . . . . . . . . . . . . . . . . . . . . . . . . . . . . . . . . . . . . . . . . . . . . . . . . . . . . . . . . . MT232844.1_India_Pig**

**2101 . . . . . . . . . . . . . . . . . . . . . . . . . . . . . . . . . . . . . . . . . . . . . . . . . . . . . . . . . . . . . . . . . . . . . . . . . . . . . . . . . . . . . . . . . . . . . . . . . . . . MT253737.1_Australia_Pig**

**2101 . . . . . . . . . . . . . . . . . . . . . . . . . . . . . . . . . . . . . . . . . . . . . . . . . . . . . . . . . . . . . . . . . . . . . . . . . . . . . . . . . . . . . . . . . . . . . . . . . . . . MZ702743.1_India_Assam_Pig**

**2101 . . . . . . . . . . . . . . . . . . . . . . . . . . . . . . . . R . . . . . . . . . . . . . . . . . . . . . . . . . . . . . . . . . . . . . . . . . . . . . . . . . . . . . . . . . . . . . . . . . . . U15763.1_USA_Pig**

**D I T R K E M R C G S G I F V H N D V E A W V D R Y K Y L P E T P R S L A K I V H K A H K E G V C G V R S V T R L E H Q M W E A V R D E L N V L L K E N A V D L S V V V N K P V G R Y R S A P K R L S M Majority**

**------------------+-------------------+-------------------+-------------------+-------------------+-------------------+-------------------+-------------------+-------------------+-------------------+-**

**810 820 830 840 850 860 870 880 890 900**

**------------------+-------------------+-------------------+-------------------+-------------------+-------------------+-------------------+-------------------+-------------------+-------------------+-**

**2401 . . . . . . . . . . . . . . . . . . . . . . . . . . . . . . . . . . . . . . . . . . . . . . . . . . . . . . . . . . . . . . . . . . . . . . . . . . . . . . . . . . . . . . . . . . . . . . . . . . . . AB551990.1_Japan_Pig**

**2401 . . . . . . . . . . . . . . . . . . . . . . . . . . . . . . . . . . . . . . . . . . . . . . . . . . . . . . . . . . . . . . . S . . . . . . . . . . . . . . . . . . . . . . . . . . . . . . . . . . . . AY316157.1_Korea_Pig**

**2401 . . . . . . . . . . . . . . . . . . . . . . . . . . . . . . . . . . . . . . . . . . . . . . . . . . . . . . . . . . . . . . . . . . . . . . . P . . . . . . . . . . . . . . . . . . . . . . . . . . . . AY849939.1_China_Pig**

**2401 . . . . . . . . . . . . . . . . . . . . . . . . . . . . . . . . . . . . . . . . . . . . . . . . . . . . . . . . . . . . . . . . . . . . . . . . . . . . . . . . . . . . . . . . . . . . . . . . . . . . EF107523.1_China_Pig**

**2401 . . . . . . . . . . . . . . . . . . . . . . . . . . . . . . . . . . . . . . . . . . . . Q . . . . . . . . . . . . . . . . . . S . . . . . . . . . . . . . . . . . . . . . . . . . . . . . . . . . . . . GQ902058.1_Thailand_Pig**

**2401 . . . . . . . . . . . . . . . . . . . . . . . . . . . . . . . . . . . . . . . . . . . . Q . . . . . . . . . . . . . . . . . . S . . . . . . . . . . . . . . . . . . . . . . . . . . . . . . . . . . . . GQ902061.1_Thailand_Pig**

**2401 . . . . . . . . . . . . . . . . . . . . . . . . . . . . . . . . . . . . . . . . . . . . . . . . . . . . . . . . . . . . . . . . . . . . . . . . . . . . . . . . . . . . . . . . . . . . . . . . . . . . JN381872.1_China_Pig**

**2401 . . . . . . . . . . . . . . . . . . . . . . . . . . . . . . . . . . . . . . . . . . . . . . . . . . . . . . . . . . . . . . . . . . . . . . . . . . . . . . . . . . . . . . . . . . . . . . . . . . . . KC915016.1_China_Pig**

**2401 . . . . . . . . . . . . . . . . . . . . . . . . . . . . . . . . . . . . . . . . . . . . . . . . . . . . . . . . . . . . . . . . . . . . . . . . . . . . . . . . . . . . . . . . . . . . . . . . . . . . KF297916.1_China_Pig**

**2401 . . . . . . . . . . . . . . . . . . . . . . . . . . . . . . . . . . . . . . . . . . . . . . . . . . . . . . . . . . . . . . . . . . . . . . . . . . . . . . . . . . . . . . . . . . . . . . . . . . . . KF711994.1_South Korea_Pig**

**2401 . . . . . . . . . . . . . . . . . . . . . . . . . . . . . . . . . S . . . . . . . . . . . . . . . . . . . . . . . . . . . . . . . . . . . . . . . . . . . . . . . . . . . . . . . . . . . . . . . . . . KP164498.2_India_Pig**

**2401 . . . . . . . . . . . . . . . . . . . . . . . . . . . . . . . . . . . . . . . . . . . . . . . . . . . . . . . . . . . . . . . . . . . . . . . . . . . . . . . . . . . . . . . . . . . . . . . . . . . . KT447437.1_South Korea_Pig**

**2401 . . . . . . . . . . . . . . . . . . . . . . . . . . . . . . . . . . . . . . . . . . . . . . . . . . . . . . . . . . . . . . . . . . . . . . . . . . . . . . . . . . . . . . . . . . . . . . . . . . . . KU363309.1_China_Pig**

**2401 . . . . . . . . . . . . . . . . . . . . . . . . . . . . . . . . . . . . . . . . . . . . . . . . . . . . . . . . . . . . . . . . . . . . . . . . . . . . . . . . . . . . . . . . . . . . . . . . . . . . KX965684.1_China_Pig**

**2401 . . . . . . . . . . . . . . . . . . . . . . . . . . . . . . . . . . . . . . . . . . . . Q . . . . . . . . . . . . . . . . . . S . . . . . . . . . . . . . . . . . . . . . . . . . . . . . . . . . . . . KY927818.1_Cambodia_Pig**

**2401 . . . . . . . . . . . . . . . . . . . . . . . . . . . . . . . . . . . . . . . . . . . . . . . . . . . . . . . . . . . . . . . . . . . . . . . . . . . . . . . . . . . . . . . . . . . . . . . . . . . . LC461960.1_Japan_Pig**

**2401 . . . . . . . . . . . . . . . . . . . . . . . . . . . . . . . . . . . . . . . . . . . . Q . . . . . . . . . . . . . . . . . . S . . . . . . . . . . . . T . . . . . . . . . . . . . . . . . . . . . . . LC708276._Japan_Pig**

**2401 . . . . . . . . . . . . . . . . . . . . . . . . . . . . . . . . . . . . . . . . . . . . Q . . . . . . . . . . . . . . . . . . S . . . . . . . . . . . . . . . . . . . . . . . . . . . . . . . . . . . . MN544780.1_China_Pig**

**2401 . . . . . . . . . . . . . . . . . . . . . . . . . . . . . . . . . . . . . . . . . . . . Q . . . . . . . . . . . . . . . . . . S . . . . . . . . . . . . . . . . . . . . . . . . . . . . . . . . . . . . MT232844.1_India_Pig**

**2401 . . . . . . . . . . . . . . . . . . . . . . . . . . . . . . . . . K . . . . . . . . . Y . . . . . . I . . . . . . . . . . . . S . . . . . . . . . . . . . . . . . . . . . . . . . . . . . . . . . . . . MT253737.1_Australia_Pig**

**2401 . . . . . . . . . . . . . . . . . . . . . . . . . . . . . . . . . . . . . . . . . . . . Q . . . . . . . . . . . . . . . . . . S . . . . . . . . . . . . . . . . . . . . . . . . . . . . . . . . . . . . MZ702743.1_India_Assam_Pig**

**2401 . . . . . . . . . . . . . . . . . . . . . . . . . . . . . . . . . . . . . . . . . . . . . . . . . . . . . . . . . . . . . . . . . . . . . . . . . . . . . . . . . . . . . . . . . . . . . . . . . . . . U15763.1_USA_Pig**

**T Q E K F E M G W K A W G K S I L F A P E L A N S T F V V D G P E T K E C P D E H R A W N S M Q I E D F G F G I T S T R V W L K I R E E S T D E C D G A I I G T A V K G H V A V H S D L S Y W I E S R Y Majority**

**------------------+-------------------+-------------------+-------------------+-------------------+-------------------+-------------------+-------------------+-------------------+-------------------+-**

**910 920 930 940 950 960 970 980 990 1000**

**------------------+-------------------+-------------------+-------------------+-------------------+-------------------+-------------------+-------------------+-------------------+-------------------+-**

**2701 . . . . . . . . . . . . . . . . . . . . . . . . . . . . . . . . . . . . . . . . . . . . . . . . . . . . . . . . . . . . . . . . . . . . . . . . . . . . . . . . . . . . . . . . . . . . . . . . . . . . AB551990.1_Japan_Pig**

**2701 . . . . . . . . . . . . . . . . . . . . . . . . . . . . . . . . . . . . . . . . . . . . . . . . . . . . . . . . . . . . . . . . . . . . T . . . . . . . . . . . . . . . . . T . . . . V . . . . . . . . AY316157.1_Korea_Pig**

**2701 . . . . . . . . . . . . . . . . . . . . . . . . . . . . . . . . . . . . . . . . . . . . . . . . . . . . . . . . . . . . . . . . . . . . . . . . . . . . . . . . . . . . . . . . . . . . . . . . . . . . AY849939.1_China_Pig**

**2701 . . . . . . . . . . . . . . . . . . . . . . . . . . . . . . . . . . . . . . . . . . . . . . . . . . . . . . . . . . . . . . . . . . . . . . . . . . . . . . . . . . . . . . . . . . . . . . . . . . . . EF107523.1_China_Pig**

**2701 . . . . . . . . . . . . . . . . . . . . . . . . . . . . . . . . . . . . . . . . R . . . . . . . . . . . . . . . . . . . . . . . . . . . D . . . . . . . . . . . . . . . . . . . . . . . . . . . . . . L GQ902058.1_Thailand_Pig**

**2701 . . . . . . . . . . . . . . . . . . . . . . . . . . . . . . . . . . . . . . . . R . . . . . . . . . . . . . . . . . . . . . . . . . . . D . . . . . . . . . . . . . . . . . . . . . . . . . . . . . . F GQ902061.1_Thailand_Pig**

**2701 . . . . . . . . . . . . . . . . . . . . . . . . . . . . . . . . . . . . . . . . . . . . . . . . . . . . . . . . . . . . . . . . . . . . . . . . . . . . . . . . . . . . . . . . . . . . . . . . . . . . JN381872.1_China_Pig**

**2701 . . . . . . . . . . . . . . . . . . . . . . . . . . . . . . . . . . . . . . . . . . . . . . . . . . . . . . . . . . . . . . . . . . . . . . . . . . . . . . . . . . . . . . . . . . . . . . . . . . . . KC915016.1_China_Pig**

**2701 . . . . . . . . . . . . . . . . . . . . . . . . . . . . . . . . . . . . . . . . . . . . . . . . . . . . . . . . . . . . . . . . . . . . . . . . . . . . . . . . . . . . . . . . . . . . . . . . . . . . KF297916.1_China_Pig**

**2701 . . . . . . . . . . . . . . . . . . . . . . . . . . . . . . . . . . . . . . . . . . . . . . . . . . . . . . . . . . . . . . . . . . . . . . . . . . . . . . . . . . . . . . . . . . . . . . . . . . . . KF711994.1_South Korea_Pig**

**2701 . . . . . . . . . . . . . . . . . . . . . . . . . . . . . . . . . . . . . . . . . . . . . . . . . . . . . . . . . . . . . . . . . . . . . . . . . . . . . . . . . . . . . . . . . . . . . . . . . . . . KP164498.2_India_Pig**

**2701 . . . . . . . . . . . . . . . . . . . . . M . . . . . . . . . . . . . . . . . . . . . . . . . . . . . . . . . . . . . . . . . . . . . . . . . . . . . . . . . . . . . . . . . . . . . . . . . . . . . F KT447437.1_South Korea_Pig**

**2701 . . . . . . . . . . . . . . . . . . . . . . . . . . . . . . . . . . . . . . . . . . . . . . . . . . . . . . . . . . . . . . . . . . . . . . . . . . . . . . . . . . . . . . . . . . . . . . . . . . . . KU363309.1_China_Pig**

**2701 . . . . . . . . . . . . . . . . . . . . . . . . . . . . . . . . . . . . . . . . . . . . . . . . . . . . . . . . . . . . . . . . . . . . . . . . . . . . . . . . . . . . . . . . . . . . . . . . . . . . KX965684.1_China_Pig**

**2701 . . . . . . . . . . . . . . . . . . . . . . . . . . . . . . . . . . . . . . . . R . . . . . . . . . . . . . . . . . . . . . . . . . . . D . . . . . . . . . . . . . . . . . . . . . . . . . . . . . . L KY927818.1_Cambodia_Pig**

**2701 . . . . . . . . . . . . . . . . . . . . . . . . . . . . . . . . . . . . . . . . . . . . . . . . . . . . . . . . . . . . . . . . . . . . . . . . . . . . . . . . . . . . . . . . . . . . . . . . . . . . LC461960.1_Japan_Pig**

**2701 . . . . . . . . . . . . . . . . . . . . . . . . . . . . . . . . . . . . . . . . R . . . . . . . . . . . . . . . . . . . . . . . . . . . N . . . . . . . . . . . . . . . . . . . . . . . . . . . . . . L LC708276._Japan_Pig**

**2701 . . . . . . . . . . . . . . . . . . . . . . . . . . . . . . . . . . . . . . . . R . . . . . . . . . . . . . . . . . . . . . . . . . . . N . . . . . . . . . . . . . . . . . . . . . . . . . . . . . . L MN544780.1_China_Pig**

**2701 . . . . . . . . . . . . . . . . . . S . . . . . . . . . . . . . . . . . . . . . R . . . . . . . . . . . . . . . . . . . . . . . . . . . N . . . . . . . . . . . . . . . . . . . . . . . . . . . . . . L MT232844.1_India_Pig**

**2701 . . . . . . . . . . . . . . . . . . . . . . . . . . . . . . . . . . . . . . . . . . . . . . . . . . . . . . . . . . . . . . . . . . . . . . . . . . . . . . . . . . . . . . . . . . . . . . . . . . . F MT253737.1_Australia_Pig**

**2701 . . . . . . . . . . . . . . . . . . S . . . . . . . . . . . . . . . . . . . . . R . . . . . . . . . . . . . . . . . . . . . . . . . . . N . . . . . . . . . . . . . . . . . . . . . . . . . . . . . . L MZ702743.1_India_Assam_Pig**

**2701 . . . . . . . . . . . . . . . . . . . . . . . . . . . . . . . . . . . . . . . . . . . . . . . . . . . . . . . . . . . . . . . . . . . . . . . . . . . . . . . . . . . . . . . . . . . . . . . . G . . . U15763.1_USA_Pig**

**N D T W K L E R A V F G E V K S C T W P E T H T L W G D G V E E S E L I I P H T I A G P K S K H N R R E G Y K T Q N Q G P W D E N G I V L D F D Y C P G T K V T I T E D C G K R G P S V R T T T D S G K Majority**

**------------------+-------------------+-------------------+-------------------+-------------------+-------------------+-------------------+-------------------+-------------------+-------------------+-**

**1010 1020 1030 1040 1050 1060 1070 1080 1090 1100**

**------------------+-------------------+-------------------+-------------------+-------------------+-------------------+-------------------+-------------------+-------------------+-------------------+-**

**3001 . . . . . . . . . . . . . . . . . . . . . . . . . . . . . . . . . . . . . . . . . . . . . . . . . . . . . . . . . . . . . . . . . . . . . . . . . . . . . . . . . . . . . . . . . . . . . . . . . . . . AB551990.1_Japan_Pig**

**3001 . . . . . R . . . . . E . . . . . . . . . . . . . . . . . . . . . . . . . . . . . . . . R . . . . . . . . . . . . . . . . . . . . . . . . . . . . . . . . . . . . . . . . . . . . . . L . . . . . . . . AY316157.1_Korea_Pig**

**3001 . . . . . . . . . . . . . . . . . . . . . . . . . . . . . . . . . . . . . . . . . . . . . . . . . . . . . . . . . . . . . . . . . . . . . . . . . . . . . . . . . . . . . . . . . . . . . . . . . . . . AY849939.1_China_Pig**

**3001 . . . . . . . . . . . . . . . . . . . . . . . . . . . . . . . . . . . . . . . . . . . . . . . . . . . . . . . . . . . . . . . . . . . . . . . . . . . . . . . . . . . . . . . . . . . . . . . . . . . . EF107523.1_China_Pig**

**3001 . . . . . . . . . . . . . . . . . . . . . . . . . . . . . . . . . . . . . . . . . . . . . . . . . . . . . . . . . . . . . . . . . . . . . . . . . . . . . . . . . . . . . . . . . . . I . . . . . . . . GQ902058.1_Thailand_Pig**

**3001 . . . . . . . . . . . . . . . . . . . . . . . . . . . . . . . . . . . . . . . . . . . . R . . . . . . . . . . . . . . . . . . . . . . . . . . . . . . . . . . . . . . . . . . . . . . I . . . . . . . . GQ902061.1_Thailand_Pig**

**3001 . . . . . . . . . . . . . . . . . . . . . . . . . . . . . . . . . . . . . . . . . . . . . . . . . . . . . . . . . . . . . . . . . . . . . . . . . . . . . . . . . . . . . . . . . . . . . . . . . . . . JN381872.1_China_Pig**

**3001 . . . . . . . . . . . . . . . . . . . . . . . . . . . . . . . . . . . . . . . . . . . . . . . . . . . . . . . . . . . . . . . . . . . . . . . . . . . . . . . . . . . . . . . . . . . . . . . . . . . . KC915016.1_China_Pig**

**3001 . . . . . . . . . . . . . . . . . . . . . . . . . . . . . . . . . . . . . . . . . . . . . . . . . . . . . . . . . . . . . . . . . . . . . . . . . . . . . . . . . . . . . . . . . . . . . . . . . . . . KF297916.1_China_Pig**

**3001 . . . . . . . . . . . . . . . . . . . . . . . . . . . . . . . . . . . . . . . . . . . . . . . . . . . . . . . . . . . . . . . . . . . . . . . . . . . . . . . . . . . . . . . . . . . . . . . . . . . . KF711994.1_South Korea_Pig**

**3001 . . . . . . . . . . . . . . . . . . . . . . . . . . . . . . . . . . . . . . . . . . . . . . . . . . . . . . . . . . . . . . . . . . . . . . . . . . . . . . . . . . . . . . . . . . . . . . . . . . . . KP164498.2_India_Pig**

**3001 . . . . . . . . . . . . . . . . . . . . . . . . . . . . . . . . . . . . . . . . . . . . . . . . . . . . . . . . . . . . . . . . . . . . . . . . . . . . . . . . . . . . . . . . . . . . . . . . . . . . KT447437.1_South Korea_Pig**

**3001 . . . . . . . . . . . . . . . . . . . . . . . . . . . . . . . . . . . . . . . . . . . . . . . . . . . . . . . . . . . . . . . . . . . . . . . . . . . . . . . . . . . . . . . . . . . . . . . . . . . . KU363309.1_China_Pig**

**3001 . . . . . . . . . . . . . . . . . . . . . . . . . . . . D . . . . . . . . . . . . . . . . . . . . . . . . . . . . . . . . . . . . . . . . . . . . . . . . . . . . . . . . S . . . . . . . . . . . . . . KX965684.1_China_Pig**

**3001 . . . . . . . . . . . . . . . . . . . . . . . . . . . . . . . . . . . . . . . . . . . . R . . . . . . . . . . . . . . . . . . . . . . . . . . . . . . . . . . . . . . . . . . . . . . I . . . . . . . . KY927818.1_Cambodia_Pig**

**3001 . . . . . . . . . . . . . . . . . . . . . . . . . . . . . . . . . . . . . . . . . . . . . . . . . . . . . . . . . . . . . . . . . . . . . . . . . . . . . . . . . . . . . . . . . . . . . . . . . . . . LC461960.1_Japan_Pig**

**3001 . . . . . . . . . . . . . . . . . . . . . . . . . . . . . . . . . . . . . . . . . . . . R . . . . . . . . . . . . . . . . . . . . . . . . . . . . . . . . . . . . . . . . . . . . . . I . . . . . . . . LC708276._Japan_Pig**

**3001 . . . . . . . . . . . . . . . . . . . . . . . . . . . . . . . . . . . . . . . . . . . . R . . . . . . . . . . . . . . . . . . . . . . . . . . . . . . . . . . . . . . . . . . . . . . I . . . . . . . . MN544780.1_China_Pig**

**3001 . . . . . . . . . . . . . . . . . . . . . . . . . . . . . . . . . . . . . . . . . . . . R . . . . . . . . . . . . . . . . . . . . . . . . . . . . . . . . . . . . . . . . . . . . . . I . . . . . . . . MT232844.1_India_Pig**

**3001 . . . . . . . . . . . . . . . . . . . . . . . . . . . . . . . . . D . V . . . . . . . . R . . . . . . . . . . . . . . . . . . . . . . . . . . . . . . . . T . . . . . . . . . . . . . . . . . . . . . . MT253737.1_Australia_Pig**

**3001 . . . . . . . . . . . . . . . . . . . . . . . . . . . . . . . . . . . . . . . . . . . . R . . . . . . . . . . . . . . . . . . . . . . . . . . . . . . . . . . . . . . . . . . . . . . I . . . . . . . . MZ702743.1_India_Assam_Pig**

**3001 . . . . . . . . . . . . . . . . . . . . . . . . . . . . D A . . . . . . . . . . . . . . . . . . . . . . . . . . . . . . . . . . . . . . . . . . . . . . . . . . . . . . . . . . . . . . . . . . . . . . U15763.1_USA_Pig**

**L I T D W C C R S C S L P P L R F R T E N G C W Y G M E I R P V R H D E T T L V R S Q V D A F N G E M V D P F Q L G L L V M F L A T Q E V L R K R W T A R L T I P A V L G A L L V L M L G G I T Y T D L Majority**

**------------------+-------------------+-------------------+-------------------+-------------------+-------------------+-------------------+-------------------+-------------------+-------------------+-**

**1110 1120 1130 1140 1150 1160 1170 1180 1190 1200**

**------------------+-------------------+-------------------+-------------------+-------------------+-------------------+-------------------+-------------------+-------------------+-------------------+-**

**3301 . . . . . . . . . . . . . . . . . . . . . . . . . . . . . . . . . . . . . . . . . . . . . . . . . . . . . . . . . . . . . . . . . . . . . . . . . . . . . . . . . . . . . . . . . . . . . . . . . . . . AB551990.1_Japan_Pig**

**3301 . . . . . . . . N . . . . . . . . . . . . . . . . . . . . . . . . . . . . . . . . . . . . . . . . . . . . . . . . . . . . . . . . . . . . . . . . . . . . . . . . . . . . . . . . . . . . . . . . . . . AY316157.1_Korea_Pig**

**3301 . . . . . . . . . . . . . . . . . . . . . . . . . . . . . . . . . . . . . . . . . . . . . . . . . . . . . . . . . . . . . . . . . . . . . F . . . . . . . . . . . . . . . . . . . . . . . . . . . . . . AY849939.1_China_Pig**

**3301 . . . . . . . . . . . . . . . . . . . . . . . . . . . . . . . . . . . . . . . . . . . . . . . . . . . . . . . . . . . . . . . . . . . . . . . . . . . . . . . . . . . . . . . . . . . F . . . . . . . . EF107523.1_China_Pig**

**3301 . . . . . . . . . . . . . . . . . . . . . . . . . . . . . . . . . . . . . . . . . . . . . . . . . . . I . . . . . . . . . . . . . . . . . . . . . . . . . . . . . . . . . . . . . . . . . . . . . . . . GQ902058.1_Thailand_Pig**

**3301 . . . . . . . . . . . . . . . . . . . . . . . . . . . . . . . . . . . . . . . . . . . . . . . . . . . I . . . . . . . . . . . . . . . . . . . . . . . . . . . . . . . . . . . . . . . . . . . . . . . . GQ902061.1_Thailand_Pig**

**3301 . . . . . . . . . . . . . . . . . . . . . . . . . . . . . . . . . . . . . . . . . . . . . . . . . . . . . . . . . . . . . . . . . . . . . . . . . . . . . . . . . . . . . . . . . . . . . . . . . . . . JN381872.1_China_Pig**

**3301 . . . . . . . . . . . . . . . . . . . . . . . . . . . . . . . . K . . . . . . . . . . . . . . . . . . . . . . . . . . . . . . . . . . . . . . . . . . . . . . . . . . . . . . . . . . . . . . . . . . . KC915016.1_China_Pig**

**3301 . . . . . . . . . . . . . . . . . . . . . . . . . . . . . . . . K . . . . . . . . . . . . . . . . . . . . . . . . . . . . . . . . . . . . . . . . . . . . . . . . . . . . . . . . . . . . . . . . . . . KF297916.1_China_Pig**

**3301 . . . . . . . . . . . . . . . . . . . . . . . . . . . . . . . . . . . . . . . . . . . A . . . . . . . . . . . . . . . . . . . . . . . . . . . . . . . . . . . . . . . . . . . . . . . . . . . . . . . . KF711994.1_South Korea_Pig**

**3301 . . . . . . . . . . . . . . . . . . . . . . . . . . . . . . . . . . . . . . . . . . . . . . . . . . . . . . . . . . . . . . . . . . . . I . . . . . . . . . . . . . . . . . . . . . . . . . . . . . . . KP164498.2_India_Pig**

**3301 . . . . . . . . . . . . . . . . . . . . . . . . . . . . . . . . . . . . . . . . . . . . . . . . . . . . . . . . . . . . . V . . . . . . . . . . . . . . . . . . . . . . . . . . . . . . . . . . . . . . KT447437.1_South Korea_Pig**

**3301 . . . . . . . . . . . . . . . . . . . . . . . . . . . . . . . . . . . . . . . . . . . . . . . . . . . . . . . . . . . . . . . . T . . . . . . . . . . . . . . . . . . . . . . . . . . . . . . . . . . . KU363309.1_China_Pig**

**3301 . . . . . . . . . . . . . . . . . . . . . . . . . . . . . . . . M . . . . . . . . . . . H . . K . . . . . . . . . . . . . . . . . . . . . . . . . . . . . . . . . . . . . V . . . . . . . . . . . . . . KX965684.1_China_Pig**

**3301 . . . . . . . . . . . . . . . . . . . . . . . . . . . . . . . . . . . . . . . . . . . . . . . . . . . I . . . . . . . . . . . . . . . . . . . . . . . . . . . . . . . . . . . . . . . . . . . . . . . . KY927818.1_Cambodia_Pig**

**3301 . . . . . . . . . . . . . . . . . . . . . . . . . . . . . . . . . . . . . . . . . . . . . . . . . . . . . . . . . . . . . . . . . . . . . . . . . . . . . . . . . . . . . . . . A . . . . . . . . . . . LC461960.1_Japan_Pig**

**3301 . . . . . . . . . . . . . . . . . . . . . . . . . . . . . . . . . . . . . . . . . . . . . . . . . . . I . . . . . . . . . . . . . . . . . . . . . . . . . . . . . . . . . . . . . . . . . . . . . . . M LC708276._Japan_Pig**

**3301 . . . . . . . . . . . . . . . . . . . . . . . . . . . . . . . . . . . . . . . . . . . . . . . . . . . I . . . . . . . . . . . . . . . . . . . . . . . . . . . . . . . . . . . . . . . . . . . . . . . . MN544780.1_China_Pig**

**3301 . . . . . . . . . . . . . . . . . . . . . . . . . . . . . . . . . . . . . . . . . . . . . . . . . . . I . . . . . . . . . . . . . . . . . . . . . . . . . . . . . . . . . . . . . . . . . . . . . . . . MT232844.1_India_Pig**

**3301 . . . . . . . . . . . . . . . . . . . D . . . . . . . . . . . A . . . . . . . . . . . . . . . . . . . I . . . . . . . . . . . . . . . . . . . . . . . . . . . V . . . . . . . . . . . . . . . . . . . . MT253737.1_Australia_Pig**

**3301 . . . . . . . . . . . . . . . . . . . . . . . . . . . . . . . . . . . . . . . . . . . . . . . . . . . I . . . . . . . . . . . . . . . . . . . . . . . . . . . . . . . . . . . . . . . . . . . . . . . . MZ702743.1_India_Assam_Pig**

**3301 . . . . . . . . . . . . . . . . . . . . . . . . . . . . . . . . . . . . . . . . . . . . . . S . . . . . . . . . . . . . . . . . . . . . . . . . . . . . . . . . . . . . . . . . . . . . . . . . . . . . U15763.1_USA_Pig**

**A R Y V V L V A A A F A E A N S G G D V L H L A L I A V F K I Q P A F L V M N M L S T R W T N Q E N V V L V L G A A F F Q L A S V D L Q I G V H G I L N A A A I A W M I V R A I T F P T T S S V T M P V Majority**

**------------------+-------------------+-------------------+-------------------+-------------------+-------------------+-------------------+-------------------+-------------------+-------------------+-**

**1210 1220 1230 1240 1250 1260 1270 1280 1290 1300**

**------------------+-------------------+-------------------+-------------------+-------------------+-------------------+-------------------+-------------------+-------------------+-------------------+-**

**3601 . . . . . . . . . . . . . . . . . . . . . . . . . . . . . . . . . . . . . . . . . . . . . . . . . . . . . . . . . . . . . . . . . . . . . . . . . . . . . . . . . . . . . . . . . . . . . . . . . . . . AB551990.1_Japan_Pig**

**3601 . . . . . . . . . . . . . . . . . . . . . . . . . . . . . . . . . . . . . . . . . . A . . . . . . . . . . . . . . . . . . . . . . . . . . . . . . . . . . . . . . . . . . . . . . . . . . . T . A . . . AY316157.1_Korea_Pig**

**3601 . . . . . . . . . . . . . . . . . . . . . . . . . . . . . . . . . . . . . . . . . . . . . . . . . . . . . . . . . . . . . . . . . . . . . . . . . . . . . . . . . . . . . . . . . . . . . . . . . . . . AY849939.1_China_Pig**

**3601 . . . . . . . . . . . . . . . . . . . . . . . . . . . . . . . . . . . . A . K . . . . . . . . . . . . . . . . . . . . . . . . . . . . . . . . . . . . . . . . . . . . . . . . . . . . . . . . . . . . . EF107523.1_China_Pig**

**3601 . . . . . . . . . . . . . . . . . . . . . . . . . . . . . . . . . . . . . . . . . . A . . . . . . . M . . . . . . . . . . . . . . . . . . . . . . . . . . . . . . . . . . . . . . . . . . . T . . . . . GQ902058.1_Thailand_Pig**

**3601 . . . . . . . . . . . . . . . . . . . . . . . . . . . . . . . . . . . . . . . . . . A . . . . . . . M . . . . . . . . . . . . . . . . . . . . . . . . . . . . . . . . . . . . . . . . . . . T . . . . . GQ902061.1_Thailand_Pig**

**3601 . . . . . . . . . . . . . . . N . . . . . . . . . . . . . . . . . . . . . . . . . . . . . . . . . . . . . . . . . . . . . . . . . . . . . . . . . . . . . . . . . . . . . . . . . . . . . . . . . . . . JN381872.1_China_Pig**

**3601 . . . . . . . . . . . . . . . . . . . . . . . . . . . . . . . . . . . . . . . . . . . . . . . . . . . . . . . . . . . . . . . . . . . . . . . . . . . . . . . . . . . . . . . . . . . . . . . . . . . . KC915016.1_China_Pig**

**3601 . . . . . . . . . . . . . . . . . . . . . . . . . . . . . . . . . . . . . . . . . . . . . . . . . . . . . . . . . . . . . . . . . . . . . . . . . . . . . . . . . . . . . . . . . . . . . . . . . . . . KF297916.1_China_Pig**

**3601 . . . . . . . . . . . . . . . G . . . . . . . . . . . . . . . . . . . . . . . . . . . . . . . . . . . . . . . . . . . . . . . . . . . . . . . . . . . . . . . . . . . . . . . . . . . . . . . . . . . . KF711994.1_South Korea_Pig**

**3601 . . . . . . . . . . . . . . . . . . . . . . . . . . . . . . . . . . . . . . . . . . . . . . . . . . . . . . . . . . L . . . . . . . . . . . . . . . . . . . . . . . . . . . . . . . . . . . . . . . . . KP164498.2_India_Pig**

**3601 . . . . . . . . . . . . . . . . . . . . . . . . . . . . . . . . . . . . . . . . . . . . . . . . . . . . . . . . . . . . . . . . . . . . . . . . . . . . . . . . . . . . . . . . . . . . . . . . . . . . KT447437.1_South Korea_Pig**

**3601 . . . . . . . . . . . . . . . . . . . . . . . . . . . . . . . . . . . . A . K . . . . . . . . . . . . . . . . . . . . . . . . L . . . . . . . . . . . . . . . . . . . . . . . . . . . . . . . . . . . . KU363309.1_China_Pig**

**3601 . . . . . . . . . . . . . . . . . . . . . . . . . . . . . . . . . . . . . . . . . . . . . . . . . . . . . . . . . . . . . . . . . . . . . . . . . . . . . . . . . . . . . . . . . . . . . . . . . . . . KX965684.1_China_Pig**

**3601 . . . . . . . . . . . . . . . N . . . . . . . . . . . . . . . . . . . . . . . . . . A . . . . . . . . . . . . . . . . . . . . . . . . . . . . . . . . . . . . . . . . . . . . . . . . . . . T . . . . . KY927818.1_Cambodia_Pig**

**3601 . . . . . . . . . . . . . . . . . . . . . . . . . V . A . . . . . . . . . . . . . . . . . . . . . . . . . . . . . . . . . . . . A . . . . . . . . . . . . . . . . . . . . . . . . . . . . . . . . . . . LC461960.1_Japan_Pig**

**3601 . . . . . . . . . . . V . . . . . . . . . . . . . . . . . . . . . S . . . . . . . . A . . . . . . . . . . . . . . . . . . . . . . . . . . . . . . . . . . . . . . . . . . . . . . . . . . . T . A . . . LC708276._Japan_Pig**

**3601 . . . . . . . . . . . . . . . . . . . . . . . . . . . . . . . . . . . . . . . . . . A . . . . . . . . . . . . . . . . . . . . . . . . . . . . . . . . . . . . . . . . . . . . . . . . . . . T . A . . I MN544780.1_China_Pig**

**3601 . . . . . . . . . . . . . . . . . . . . . . . . . . . . . . . . . . . . . . . . . . A . . . . . . . . . . . . . . . L . . . . . . . . . . . . . . . . . . . . . . . . . . . . . . . . . . . T . A . . I MT232844.1_India_Pig**

**3601 . . . . . . . . . . . . . . . N . . . . . . . . . . . . . . . . . . . . . . . . . . A K . . . . . . M . . . . . . . . . . . . . . . . . . . . . . . . . . . . . . . . . . . . . . . . . . . T . . . . . MT253737.1_Australia_Pig**

**3601 . . . . . . . . . . . . . . . . . . . . . . . . . . . . . . . . . . . . . . . . . . A . . . . . . . . . . . . . . . . . . . . . . . . . . . . . . . . . . . . . . . . . . . . . . . . . . . T . A . . I MZ702743.1_India_Assam_Pig**

**3601 . . . . . . . . . . . . . . . . . . . . . . . . . . . . . . . . . . . . . . . . . . . . . . . . . . . . . . . . . . . . . . . . . . . . . . . . . . . . . . . . . . . . . . . . . . . . . . . . . . . . U15763.1_USA_Pig**

**L A L L T P G M R A L Y L D T Y R I I L L V I G I C S L L Q E R K K T M A K K K G A V L L G L A L T S T G W F S P T T I A A G L M V C N P N K K R G W P A T E F L S A V G L M F A I V G G L A E L D I E Majority**

**------------------+-------------------+-------------------+-------------------+-------------------+-------------------+-------------------+-------------------+-------------------+-------------------+-**

**1310 1320 1330 1340 1350 1360 1370 1380 1390 1400**

**------------------+-------------------+-------------------+-------------------+-------------------+-------------------+-------------------+-------------------+-------------------+-------------------+-**

**3901 . . . . . . . . . . . . . . . . . . . . . . . . . . . . Q . . . . . . . . . . . . . . . . . . . . . . . . . . . . . . . . . . . . . . . . . . . . . . . . . . . . . . . . . . . . . . . . . . . . . . . AB551990.1_Japan_Pig**

**3901 . . . . . . . . . . F D . . . . . . . . . . . . . . . . . . . . R . . . . . . . . . . . . . . . . . . . . . . . . . . . . . . . . . . . . . . . . . . . . . . . . . . . . . . . . . . . . . . . . . . . AY316157.1_Korea_Pig**

**3901 . . . . . . . . . . . . . . . . . . . . . . . . . . . . . . . . . . . . . . . . . . . . . . . . . . . . . . . . . . . . . . . . . . . . . . . . . . . . . . . . . . . . . . . . . . . . . . . . . . . . AY849939.1_China_Pig**

**3901 . . . . . . . . . . . . . . . . . . . . . . . . . . . . . . . . . . . . . . . . . . . . . . . . . . . . . . . . . . . . . . . . . . . . . . . . . . . . . . . . . . . . . . . . . . . . . . . . . . . . EF107523.1_China_Pig**

**3901 . . . . . . . . . . . . . . . . . . . . . . . . . . . . . . . . R . . . . . . . . . . . . . . . . . . . . . . . . . . . . . . . . . . . . . . . . . . . . . . . . . . . . . . . . . . . . . . . . . . . GQ902058.1_Thailand_Pig**

**3901 . . . . . . . . . . . . . . . . . . . . . . . . . . . . . . . . R . . . . . . . . . . . . . . . . . . . . . . . . . . . . . . . . . . . . . . . . . . . . . . . . . . . . . . . . . . . . . . . . . . . GQ902061.1_Thailand_Pig**

**3901 . . . . . . . . . . . . . . . . . . . . . . . . . . . . . . . . . . . . . . . . . . . . . . . . . . . . . . . . . . . . . . . . . . . . . . . . . . . . . . . . . . . . . . . . . . . . . . . . . . . . JN381872.1_China_Pig**

**3901 . . . . . . . . . . . . . . . . . . . . . . . . . . . . . . . . . . . . . . . . . . . . . . . . . . . . . . . . . . . . . . . . . . . . . . . . . . . . . . . . . . . . . . . . . . . . . . . . . . . . KC915016.1_China_Pig**

**3901 . . . . . . . . . . . . . . . . . . . . . . . . . . . . . . . . . . . . . . . . . . . . . . . . . . . . . . . . . . . . . . . . . . . . . . . . . . . . . . . . . . . . . . . . . . . . . . . . . . . . KF297916.1_China_Pig**

**3901 . . . . . . . . . . . . . . . . . . . . . . . . . . . . . . . . . . . . . . . . . . . . . . . . . . . . . . . . . . . . . . . . . . . . . . . . . . . . . . . V . . . . . . . . . . . . . . . . . . . . KF711994.1_South Korea_Pig**

**3901 . . . . . . . . . . . . . . . . . . . . . . . . . . . . . . . . . . . . . . . . . . . . . . . . . . . . . . . . . . . . . . . . . . . . . . . . . . . . . . . . . . . . . . . . . . . . . . . . . . . . KP164498.2_India_Pig**

**3901 . . . . . . . . . . . . . . . . . . . . . . . . . . . . . . . . . . . . . . . . . . . . . . . . . . . . . . . . . . . . . . . . . . . . . . . . . . . . . . . . . . . . . . . . . . . . . . . . . . . . KT447437.1_South Korea_Pig**

**3901 . . . . . . . . . . . . . . . . . . . . . . . . . . . . . . . . . . . . . . . . . . . . . . . . . . . . . . . . . . . . . . . . . . . . . . . . . . . . . . . . . . . . . . . . . . . . . . . . . . . . KU363309.1_China_Pig**

**3901 . . . . . . . . . . . . . . . . . . . . . . . . . . . . . H . . . . . . . . . . . . . . . . . . . . . . . . . . . . . . . . . . . . . . . . . . . . . . . . . . . . . . . . . . . . . . . . . . . . . . KX965684.1_China_Pig**

**3901 . . . . . . . . . . . . . . . . . . . . . . . . . . . . . . . . R . . . . . . . . . . . . . . . . . . . . . . . . . . . . . . . . . . . . . . . . . . . . . . . . . . . . . . . . . . . . . . . . . . . KY927818.1_Cambodia_Pig**

**3901 . . . . . . . . . . . . . . . . . . . . . I . . . . . . . . . . . . . . . . . . . . . . . . . . . . . . . . . . . . . . . . . . . . . . . . . . . . . . . . . . . . . . . . . . . . . . . . . . . . . . LC461960.1_Japan_Pig**

**3901 . . . . . . . . . . . . . . . . . . . . . . . . . . . . . . . . R . . . . . . . . . . . . . . . . . . . . . . . . . . . . . . . . . . . . . . . . . . . . . . . . . . . . . . . . . . . . . . . . . . . LC708276._Japan_Pig**

**3901 . . . . . . . . . . . . . . . . . . . . . . . . . . . . . . . . R . . . . . . . . . . . . . . . . . . . . . . . . . . . . . . . . . . . . . . . . . . . . . . . . . . . . . . . . . . . . . . . . . . . MN544780.1_China_Pig**

**3901 . . . . . . . . . . . . . . . . . . . . . . . . . . . . . . . . R . . . . . . . . . . . . . . . . . . . . . . . . . . . . . . . . . . . . . . . . . . . . . . . . . . . . . . . . . . . . . . . . . . . MT232844.1_India_Pig**

**3901 . . . . . . . . . . . . . . . . . . . . . . . . . . . . . . . . R . . . . . . . . . . . . . . . . . . . . . . . . . . . . . . . . A . . . . . . . . . . . . . . . . . . . . . . . . . . . . . . . . . . MT253737.1_Australia_Pig**

**3901 . . . . . . . . . . . . . . . . . . . . . . . . . . . . . . . . R . . . . . . . . . . . . . . . . . . . . . . . . . . . . . . . . . . . . . . . . . . . . . . . . . . . . . . . . . . . . . . . . . . . MZ702743.1_India_Assam_Pig**

**3901 . . . . . . . . . . . . . . . . . . . . . . . . . . . . . H . . . . . . . . . . . . . . . . . . . . . . . . . . . . . . . . . . . . . . . . . . . . . . . . . . . . . . . . . . . . . . . . . . . . . . U15763.1_USA_Pig**

**S M S I P F M L A G L M A V S Y V V S G K A T D M W L E R A A D I S W E M D A A I T G S S R R L D V K L D D D G D F H L I D D P G V P W K V W V L R M S C I G L A A L T P W A I V P A A F G Y W L T L K Majority**

**------------------+-------------------+-------------------+-------------------+-------------------+-------------------+-------------------+-------------------+-------------------+-------------------+-**

**1410 1420 1430 1440 1450 1460 1470 1480 1490 1500**

**------------------+-------------------+-------------------+-------------------+-------------------+-------------------+-------------------+-------------------+-------------------+-------------------+-**

**4201 . . . . . . . . . . . . . . . . . . . . . . . . . . . . . . . . . . . . . . . . . . . . . . . . . . . . . . . . . . . . . . . . . . . . . . . . . . . . . . . . . . . . . . . . . . . . . . . . . . . . AB551990.1_Japan_Pig**

**4201 . . . . . . . . . . . . . . . . . . . . . . . . . . . D . . . . . . . . . E . . . . . . . . . . . . . . . . . . . . . . . . . . . . . . . . . L . . . . . . . . . . . . . . . . . . . . . . . . . . . . AY316157.1_Korea_Pig**

**4201 . . . . . . . . . . . . . . . . . . . . . . . . . . . . . . . . . . . . . . . . . . . . . . . . . . . . . . . . . . . . . . . . . . . . . . . . . . . . . S . . . . . . . . . . . . . . . . . . . . . . AY849939.1_China_Pig**

**4201 . . . . . . . . . . . . . . . . . . . . . . . . . . . . . . . . . . . . . . . . . . . . . . . . . . . . . . . . . . . . . . . . . . . . . . . . . . . . . . . . . . . . . . . . . . . . . . . . . . . . EF107523.1_China_Pig**

**4201 . . . . . . . . . . . . . . . . . . . . . . . . . . . D . . . . . . . . . E . . . . . . . . . . . . . . . . . . . . . . . . . . . . . . . . . L . . . . . . . . . . . . . . . . . . . . . . . . . . . . GQ902058.1_Thailand_Pig**

**4201 . . . . . . . . . . . . . . . . . . . . . . . . . . . D . . . . . . . . . E . . . . . . . . . . . . . . . . . . . . . . . . . . . . . . . . . L . . . . . . . . . . . . . . . . . . . . . . . . . . . . GQ902061.1_Thailand_Pig**

**4201 . . . . . . . . . . . . . . . . . . . . . . . . . . . . . . . . . . . . . . . . . . . . . . . . . . . . . . . . . . . . . . . . . . . . . . . . . . . . . . . . . . . . . . . . . . . . . . . . . . . . JN381872.1_China_Pig**

**4201 . . . . . . . . . . . . . . . . . . . . . . . . . . . . . . . . . . . . . . . . . . . . . . . . . . . . . . . . . . . . . . . . . . . . . . . . . . . . . . . . . . . . . . . . . . . . . . . . . . . . KC915016.1_China_Pig**

**4201 . . . . . . . . . . . . . . . . . . . . . . . . . . . . . . . . . . . . . . . . . . . . . . . . . . . . . . . . . . . . . . . . . . . . . . . . . . . . . . . . . . . . . . . . . . . . . . . . . . . . KF297916.1_China_Pig**

**4201 . . . . . . . . . . . . . . . . . . . . . . . . . . . . . . . . . . . . . . . . . . . . . . . . . . . . . . . . . . . . . . . . . . . . . . . . . . . . . . . . . . . . . . . . . . . . . . . . . . . . KF711994.1_South Korea_Pig**

**4201 . . . . . . . . . . . . . . . . . . . . . . . . . . . . . . . . . . . . . . . . . . . . . . . . . . . . . . . . . . . . . . . . . . . . . . . . . . . . . . . . . . . . . . . . . . . . . . . . . . . . KP164498.2_India_Pig**

**4201 . . . . . . . . . . . . . . . . . . . . . . . . . . . . . . . . . . . . . . . . . . . . . . . . . . . . . E . . . . . . . . . . . . . . . . . . . . . . . . . . . . . . . . . . . . . . . . . . . . . . KT447437.1_South Korea_Pig**

**4201 . . . . . . . . . . . . . . . . . . . . . . . . . . . . . . . . . . . . . . . . . . . . . . . . . . . . . . . . . . . . . . . . . . . . . . . . . . . . . . . . . . . . . . . . . . . . . . . . . . . . KU363309.1_China_Pig**

**4201 . . . . . . . . . . . . . . . . . . . . . . . . . . . . . . . . . . . D . G . . . . . . . . . . . . . . . . . . . . . . . . . . . . . . . . . . . . . . . . . . . . . . . . . . . . . . . . . . . . . . KX965684.1_China_Pig**

**4201 . . . . . . . . . . . . . . . . . . . . . . . . . . . D . . . . . . . . . E . . . . . . . . . . . . . . . . . . . . . . . . . . . . . . . . . L . . . . . . . . . . . . . . . . . . . . . . . . . . . . KY927818.1_Cambodia_Pig**

**4201 . . . . . . . . . . . . . . . . . . . . . . . . . . . . . . . . . . . . . . . . . . . . . . . . . . . . . . . . . . . . . . . . . . . . . . . . . . . . . . . . . . . . . . . . . . . . . . . . . . . . LC461960.1_Japan_Pig**

**4201 . . . . . . . . . . . . . . . . . . . . . . . . . . . D . . . . . . . . . E . . . . . . . . . . . . . . . . . . . . . . . . . . . . . . . . . L . . . . . . . . . . . . . . . . . . . . . . . . . . . . LC708276._Japan_Pig**

**4201 . . . . . . . . . . . . . . . . . . . . . . . . . . . D . . . . . . . . . E . . . . . . . . . . . . . . . . . . . . . . . . . . . . . . . . . L . . . . . . . . . . . . . . . . . . . . . . . . . . . . MN544780.1_China_Pig**

**4201 . . . . . . . . . . . . . . . . . . . . . . . . . . . D . . . . . . . . . E . . . . . . . . . . . . . . . . . . . . . . . . . . . . . . . . . L . . . . . . . . . . . . . . . . . . . . . . . . . . . . MT232844.1_India_Pig**

**4201 . . . . . . . . . . . . . . . . . . . . . . . . . . . . . . . . . . . . . . . . . . . . . . . . . . . . . . . . . . . . . . . . . . . . . I . L . . . . . . . . . . . . . . . . . . . . . . . . . . . . MT253737.1_Australia_Pig**

**4201 . . . . . . . . . . . . . . . . . I . . . . . . . . . D . . . . . . . . . E . . . . . . . . . . . . . . . . . . . . . . . . . . . . . . . . . L . . . . . . . . . . . . . . . . . . . . . . . . . . . . MZ702743.1_India_Assam_Pig**

**4201 . . . . . . . . . . . . . . . . . . . . . . . . . . . . . . . . . . . G . . . . . . . . . . . . . . . . . . . . . . . . . . . . . . . . . . . . . . . . . . . . . . . . . . . . . . . . . . . . . . . . U15763.1_USA_Pig**

**T T K R G G V F W D T P S P K P C S K G D T T T G V Y R I M A R G I L G T Y Q A G V G V M Y E N V F H T L W H T T R G A A I M S G E G K L T P Y W G S V K E D R I A Y G G P W R F D R K W N G T D D V Q Majority**

**------------------+-------------------+-------------------+-------------------+-------------------+-------------------+-------------------+-------------------+-------------------+-------------------+-**

**1510 1520 1530 1540 1550 1560 1570 1580 1590 1600**

**------------------+-------------------+-------------------+-------------------+-------------------+-------------------+-------------------+-------------------+-------------------+-------------------+-**

**4501 . . . . . . . . . . . . . . . . . . . . . . . . . . . . . . . . . . . . . . . . . . . . . . . . . . . . . . . . . . . . . . . . . . . . . . . . . . . . . . . . . . . . . . . . . . . . . . . . . . . . AB551990.1_Japan_Pig**

**4501 . . . . . . . . . . . . . . . . . . . . . . . . . . . . . . . . . . . . . . . . . . . . . . Q . . . . . . . . . . . . . . . . . . K . . . . . . . . . . . . . . . S . . . . . . . . . . . . . . N . . . AY316157.1_Korea_Pig**

**4501 . . . . . . . . . . . . . . . . . . . . . . . . . . . . . . . . . . . . . . . . . . . . . . . . . . . . . . . . . . . . . . . . . . . . . . . . . . . . . . . . . . . . . . . . . . . . . . . . . . . . AY849939.1_China_Pig**

**4501 . . . . . . . . . . . . . . . . . . . . . . . . . . . . . . . . . . . . . . . . . . . . . . . . . . . . . . . . . . . . . . . . . . . . . . . . . . . . . . . . . . . . . . . . . . . . . . . . . . . . EF107523.1_China_Pig**

**4501 . . . . . . . . . . . . . . . . . L . . . . . . . . . . . . . . . . . . . . . . . . . . . . . . . . . . . . . . . . . . . . . . . . . . . . . . . . . . . . . . . S . . . . . . . . . . . . . . . . . . GQ902058.1_Thailand_Pig**

**4501 . . . . . . . . . . . . . . . . . L . . . . . . . . . . . . . . . . . . . . . . . . . . . . . . . . . . . . . . . . . . . . . . . . . . . . . . . . . . . . . . . S . . . . . . . . . . . . . . . . . . GQ902061.1_Thailand_Pig**

**4501 . . . . . . . . . . . . . . . . . . . . . . . . . . . . . . . . . . . . . . . . . . . . . . . . . . . . . . . . . . . . . . . . . . . . . . . . . . . . . . . . . . . . . . . . . . . . . . . . . . . . JN381872.1_China_Pig**

**4501 . . . . . . . . . . . . . . . . . . . . . . . . . . . . . . . . . . . . . . . . . . . . . . . . . . . . . . . . . . . . . . . . . . . . . . . . . . . . . . . . . . . . . . . . . . . . . . . . . . . . KC915016.1_China_Pig**

**4501 . . . . . . . . . . . . . . . . . . . . . . . . . . . . . . . . . . . . . . . . . . R . . . . . . . . . . . . . . . . . . . . . . . . . . . . . . . . . . . . . . . . . . . . . . . . . . . . . . . . . KF297916.1_China_Pig**

**4501 . . . . . . . . . . . . . . . . . . . . . . . . . . . . . . . . . . . . . . . . . . . . . . . . . . . . . . . . . . . . . . . . . . . . . M . . . . . . . . . . . . . . . . . . . . . . . . . . . . . . KF711994.1_South Korea_Pig**

**4501 . . . . . . . . . . . . . . . . . . . . . . . . . . . . . . . . . . . . . . . . . . . . . . . . . . . . . . . . . . . . . . . . . . . . . . . . . . . . . . . . . . . . . . . . . . . . . . . . . . . . KP164498.2_India_Pig**

**4501 . . . . . . . . . . . . . . . . . . . . . . . . . . . . . . . . . . . . . . . . . . . . . . . . . . . . . . . . . . . . . . . . . . . . . . . . . . . . . . . . . . . . . . . . . . . . . . . . . . . . KT447437.1_South Korea_Pig**

**4501 . . . . . . . . . . . . . . . . . . . . . . . . . . . . . . . . . . . . . . . . . . . . . . . . . . . . . . . . . . . . . . . . . . . . . . . . . . . . . . . . . . . . . . . . . . . . . . . . . . . . KU363309.1_China_Pig**

**4501 . . . . . . . . . . . . . . . . . . . . . . . . . . . . . . . . . . F . . . . . . . . . . . . . . . . . . . . . . . . . . . V . . . . . . . . . . . . . . . . . . . . . . . . . . . . . . . . . . . . . KX965684.1_China_Pig**

**4501 . . . . . . . . . . . . . . . . . L . . . . . . . . . . . . . . . . . . . . . . . . . . . . . . . . . . . . . . . . . . . . . . . . . . . . . . . . . . . . . . . S . . . . . . . . . . . . . . . . . . KY927818.1_Cambodia_Pig**

**4501 . . . . . . . . . . . . . . . . . . . . . . . . . . . . . . . . . . . . . . . . . . . . . . . . . . . . . . . . . . . . . . . . . . . R . . . . . . . . . . . . . . . . . . . . . . . . . . . . . . . . LC461960.1_Japan_Pig**

**4501 . . . . . . . . . . . . . . . . V L . . . . . . . . . . . . . . . . . . . . . . . . . . . . . . . . . . . . . . . . . . . . . . . . . . . . . . . . . . . . . . . S . . . . . . . . . . . . . . . . . . LC708276._Japan_Pig**

**4501 . . . . . . . . . . . . . . . . . L . . . . . . . . . . . . . . . . . . . . . . . . . . . . . . . . . . . . . . . . . . . . . . . . . . . . . . . . . . . . . . . S . . . . . . . . . . . . . . . . . . MN544780.1_China_Pig**

**4501 . . . . . . . . . . . . . . . . . L . . . . . . . . . . . . . . . . . . . . . . . . . . . . . . . . . . . . . . . . . . . . . . . . . . . . . . . . . . . . . . . S . . . . . . . . . . . . . . . . . . MT232844.1_India_Pig**

**4501 . . . . . . . . . . . . . . . . . L . . . . . . . . . . . . . . . . . . . . . . . . . . . . . . . . . . . . . . . . . . . . . . . G . . . . . . . . . . . . . . . S . . . . . . . . . . . . . . . . . . MT253737.1_Australia_Pig**

**4501 . . . . . . . . . . . . . . . . . L . . . . . . . . . . . . . . . . . . . . . . . . . . . . . . . . . . . . . . . . . . . . . . . . . . . . . . . . . . . . . . . S . . . . . . . . . . . . . . . . . . MZ702743.1_India_Assam_Pig**

**4501 . . . . . . . . . . . . . . . . . . . . . . . . . . . . . . . . . . . . . . . . . . . . . . . . . . . . . . . . . . . . . . . . . . . . . . . . . . . . R . . . . . . . . . . . . . . . . . . . . . . . U15763.1_USA_Pig**

**V I V V E P G K A A V N I Q T K P G V F R T P F G E V G A V S L D Y P R G T S G S P I L D S N G D I I G L Y G N G V E L G D G S Y V S A I V Q G D R Q E E P V P E A Y T P N M L R K R Q M T V L D L H P Majority**

**------------------+-------------------+-------------------+-------------------+-------------------+-------------------+-------------------+-------------------+-------------------+-------------------+-**

**1610 1620 1630 1640 1650 1660 1670 1680 1690 1700**

**------------------+-------------------+-------------------+-------------------+-------------------+-------------------+-------------------+-------------------+-------------------+-------------------+-**

**4801 . . . . . . . . . . . . . . . . . . . . . . . . . . . . . . . . . . . . . . . . . . . . . . . . . . . . . . . . . . . . . . . . . . . . . . . . . . . . . . . . . . . . . . . . . . . . . . . . . . . . AB551990.1_Japan_Pig**

**4801 . . . . . . . . P . . . . . . . . . I . P . . . . K . . T . . . . . . . . . . . . . . . . . . . . . . . . . . . . . . . . . . . . . . . . . . . . . . . . . . . D . . . . S . . K . . . . . . . . . . . AY316157.1_Korea_Pig**

**4801 . . . . . . . . . . . . . . . . . . . . . . . . . . . . . . . . . . . . . . . . . . . . . . . . . . . . . . . . . . . . . . . . . . . . . . . . . . . . . . . . . . . . . . . . . . . . . . . . . . . . AY849939.1_China_Pig**

**4801 . . . . . . . . . . . . . . . . . . . . . . . . . . . . . . . . . . . . . . . . . . . . . . . . . . . . . . . . . . . . . . . . . . . . . . . . . . . . . . . . . . . . . . . . . . . . . . . . . . . . EF107523.1_China_Pig**

**4801 . . . . . . . . P . . . . . . . . . . . . . . . . . I . . . . . . . . . . . . . . . . . . . . . . . . . . . . . . . . . . . . . . . . . . . . . . . . . . . . . D . . . . S . . K . . . . . . . . . . . GQ902058.1_Thailand_Pig**

**4801 . . . . . . . . P . . . . . . . . . . . . . . . . . I . . . . . . . . . . . . . . . . . . . . . . . . . . . . . . . . . . . . . . . . . . . . . . . . . . . . . D . . . . S . . K . . . . . . . . . . . GQ902061.1_Thailand_Pig**

**4801 . . . . . . . . . . . . . . . . . . . . . . . . . . . . . . . . . . . . . . . . . . . . . . . . . . . . . . . . . . . . . . . . . . . . . . . . . . . . . . . . . . . . . . . . . . . . . . . . . . . . JN381872.1_China_Pig**

**4801 . . . . . . . . . . . . . . . . . . . . . . . . . . . . . . . . . . . . . . . . . . . . . . . . . . . . . . . . . . . . . . . . . . . . . . . . . . . . . . . . . . . . . . . . . . . . . . . . . . . . KC915016.1_China_Pig**

**4801 . . . . . . . . . . . . . . . . . . . . . . . . . . . . . . . . . . . . . . . . . . . . . . . . . . . . . . . . . . . . . . . . . . . . . . . . . . . . . . . . . . . . . . . . . . . . . . . . . . . . KF297916.1_China_Pig**

**4801 . . . . . . . . . . . . . . . . . . . . . . . . . . . . . . . . . . . . . . . . . . . . . . . . . . . . . . . . . . . . . . . . . . . . . . . . . . . . . . . . . . . . . . . . . . . . . . . . . . . . KF711994.1_South Korea_Pig**

**4801 . . . . . . . . . . . . . . . . . . . . . . . . . . . . . . . . . . . . . . . . . . . . . . . . . . . . . . . . . . . . . . . . . . . . . . . . . . . . . . . . . . . . . . . . . . . . . . . . . . . . KP164498.2_India_Pig**

**4801 . . . . . . . . . . . . . . . . . . . . . . . . . . . . . . . . . . . . . . . . . . . . . . . . . . . . . . . . . . . . . . . . . . . . . . . . . . . . . . . . . . . . . . . . . . . . . . . . . . . . KT447437.1_South Korea_Pig**

**4801 . . . . . . . . . . . . . . . . . . . . . . . . . . . . . . . . . . . . . . . . . . . . . . . . . . . . . . . . . . . . . . . . . . . . . . . . . . . . . . . . . . . . . . . . . . . . . . . . . . . . KU363309.1_China_Pig**

**4801 . . . . . . . . G . . . . . . . . . . . . . . . . . . . . . . . . . . . . . . . . . . . . . . . . . . . . . . . . . . . . . . . . . . . . . . . . . . . . . . . . . . . . . . . . . . . . . . . . . . . KX965684.1_China_Pig**

**4801 . . . . . . . . P . . . . . . . . . . . . . . . . . . . . . . . . . . . . . . . . . . . . . . . . V . . . . . . . . . . . . . . . . . . . . . . . . . . . . . . D . . . . S . . K . . . . . . . . . . . KY927818.1_Cambodia_Pig**

**4801 . . . . . . . . . . . . . . . . . . . . . . . . . . . . . . . . . . . . . . . . . . . . . . . . . . . . . . . . . . . . . . . . . . . . . . . . . . . . . . . . . . . . . . . . . . . . . . . . . . . . LC461960.1_Japan_Pig**

**4801 . . . . . . . . P . . . . . . . . . . . . . . . . . I . . . . . . . . . . . . . . . . . . . . . . . . . . . . . . . . . . . . . . . . . . . . . . . . . . . . . D . . . . G . . K . . . . . . . . . . . LC708276._Japan_Pig**

**4801 . . . . . . . . P . . . . . . . . . . . . . . . . . . . . . . . . . . . . . . . . . . . . . . . . . . . . . . . . . . . . . . . . . . . . . . . . . . . . . . . D . . . . S . . K . . . . . . . . . . . MN544780.1_China_Pig**

**4801 . . . . . . . . P . . . . . . . . . . . . . . . . . . . . . . . . . . . . . . . . . . . . . . . . V . . . . . . . . . . . . . . . . . . . . . . . . . . . . . . D . . . . S . . K . . . . . . . . . . . MT232844.1_India_Pig**

**4801 . . . . . . . . . . . . . . . . . . . . . . . . . . . . . . . . . . . . . . . . . . . . . . . . . . . . . . . . . . . . . . . . . . . . . . . . . . . . . . . . . . . . . S . . K . . . . . . . . . . . MT253737.1_Australia_Pig**

**4801 . . . . . . . . P . . . . . . . . . . . . . . . . . . . . . . . . . . . . . . . . . . . . . . . . . . . . . . . . . . . . . . . . . . . . . . . . . . . . . . . D . . . . S . . K . . . . . . . . . . . MZ702743.1_India_Assam_Pig**

**4801 . . . . . . . . V . . . . . . . . . . . . . . . . . . . . . . . . . . . . . . . . . . . . . . . . . . . . . . . . . . . . . . . . . . . . . . . . . . . . . . . . . . . . . . . . . . . . . . . . . . . U15763.1_USA_Pig**

**G S G K T R K I L P Q I I K D A I Q Q R L R T A V L A P T R V V A A E M A E A L R G L P V R Y Q T S A V Q R E H Q G N E I V D V M C H A T L T H R L M S P N R V P N Y N L F V M D E A H F T D P A S I A Majority**

**------------------+-------------------+-------------------+-------------------+-------------------+-------------------+-------------------+-------------------+-------------------+-------------------+-**

**1710 1720 1730 1740 1750 1760 1770 1780 1790 1800**

**------------------+-------------------+-------------------+-------------------+-------------------+-------------------+-------------------+-------------------+-------------------+-------------------+-**

**5101 . . . . . . . . . . . . . . . . . . . . . . . . . . . . . . . . . . . . . . . . . . . . . . . . . . . . . . . . . . . . . . . . . . . . . . . . . . . . . . . . . . . . . . . . . . . . . . . . . . . . AB551990.1_Japan_Pig**

**5101 . . . . . . . . . . . . . . . . . . . . . . . . . . . . . . . . . . . . . . . . . . . . . . . . . . . . . . . . . . . . . . . . . . . . . . . . . . . . . . . . . . . . . . . . . . . . . . . . . . . . AY316157.1_Korea_Pig**

**5101 . . . . . . . . . . . . . . . . . . . . . . . . . . . . . . . . . . . . . . . . . . . . . . . . . . . . . . . . . . . . . . . . . . . . . . . . . . . . . . . . . . . . . . . . . . . . . . . . . . . . AY849939.1_China_Pig**

**5101 . . . . . . . . . . . . . . . . . . . . . . . . . . . . . . . . . . . . . . . . . . . . . . . . . . . . . . . . . . . . . . . . . . . . . . . . . . . . . . . . . . . . . . . . . . . . . . . . . . . . EF107523.1_China_Pig**

**5101 . . . . . . . . . . . . . . . . . . . . . . . . . . . . . . . . . . . . . . . . . . . . . . . . . . . . . . . . . . . . . . . . . . . . . . . . . . . . . . . . . . . . . . . . . . . . . . . . . . . . GQ902058.1_Thailand_Pig**

**5101 . . . . . . . . . . . . . . . . . . . . . . . . . . . . . . . . . . . . . . . . . . . . . . . . . . . . . . . . . . . . . . . . . . . . . . . . . . . . . . . . . . . . . . . . . . . . . . . . . . . . GQ902061.1_Thailand_Pig**

**5101 . . . . . . . . . . . . . . . . . . . . . . . . . . . . . . . . . . . . . . . . . . . . . . . . . . . . . . . . . . . . . . . . . . . . . . . . . . . . . . . . . . . . . . . . . . . . . . . . . . . . JN381872.1_China_Pig**

**5101 . . . . . . . . . . . . . . . . . . . . . . . . . . . . . . . . . . . . . . . . . . . . . . . . . . . . . . . . . . . . . . . . . . . . . . . . . . . . . . . . . . . . . . . . . . . . . . . . . . . . KC915016.1_China_Pig**

**5101 . . . . . . . . . . . . . . . . . . . . . . . . . . . . . . . . . . . . . . . . . . . . . . . . . . . . . . . . . . . . . . . . . . . . . . . . . . . . . . . . . . . . . . . . . . . . . . . . . . . . KF297916.1_China_Pig**

**5101 . . . . . . . . . . . . . . . . . . . . . . . . . . . . . . . . . . . . . . . . . . . . . . . . . . . . . . . . . . . . . . . . . . . . . . . . . . . . . . . . . . . . . . . . . . . . . . . . . . . . KF711994.1_South Korea_Pig**

**5101 . . . . . . . . . . . . . . . . . . . . . . . . . . . . . . . . . . . . . . . . . . . . . . . . . . . . . . . . . . . . . . . . . . . . . . . . . . . . . . . . . . . . . . . . . . . . . . . . . . . . KP164498.2_India_Pig**

**5101 . . . . . . . . . . . . . . . . . . . . . . . . . . . . . . . . . . . . . . . . . . . . . . . . . . . . . . . . . . . . . . . . . . . . . . . . . . . . . . . . . . . . . . . . . . . . . . . . . . . . KT447437.1_South Korea_Pig**

**5101 . . . . . . . . . . . . . . . . . . . . . . . . . . . . . . . . . . . . . . . . . . . . . . . . . . . . . . . . . . . . . . . . . . . . . . . . . . . . . . . . . . . . . . . . . . . . . . . . . . . . KU363309.1_China_Pig**

**5101 . . . . . . . . . . . . . . . . . . . . . . . . . . . . . . . . . . . . . . . . . . . . . . . . . . . . . . . . . . . . . . . . . . . . . . . . . . . . . . . . . . . . . . . . . . . . . . . . . . . . KX965684.1_China_Pig**

**5101 . . . . . . . . . . . . . . . . . . . . . . . . . . . . . . . . . . . . . . . . . . . . . . . . . . . . . . . . . . . . . . . . . . . . . . . . . . . . . . . . . . . . . . . . . . . . . . . . . . . . KY927818.1_Cambodia_Pig**

**5101 . . . . . . . . . . . . . . . . . . . . . . . . . . . . . . . . . . . . . . . . . . . . . . . . . . . . . . . . . . . . . . . . . . . . . . . . . . . . . . . . . . . . . . . . . . . . . . . . . . . . LC461960.1_Japan_Pig**

**5101 . . . . . . . . . . . . . . . . . . . . . . . . . . . . . . . . . . . . . . . . K . . . . . . . . . . . . . . . . . . . . . . . . . . . . . . . . . . . . . . . . . . . . . . . . . . . . . . . . . . . LC708276._Japan_Pig**

**5101 . . . . . . . . . . . . . . . . . . . . . . . . . . . . . . . . . . . . . . . . . . . . . . . . . . . . . . . . . . . . . . . . . . . . . . . . . . . . . . . . . . . . . . . . . . . . . . . . . . . . MN544780.1_China_Pig**

**5101 . . . . . . . . . . . . . . . . . . . . . . . . . . . . . . . . . . . . . . . . . . . . . . . . . . . . . . . . . . . . . . . . . . . . . . . . . . . . . . . . . . . . . . . . . . . . . . . . . . . . MT232844.1_India_Pig**

**5101 . . . . . . . . . . . . . . . . . . . . . . . . . . . . . . . . . . . . . . . . . . . . . . . . . . . . . . . . . . . . . . . . . . . . . . . . . . . . . . . . . . . . . . . . . . . . . . . . . . . . MT253737.1_Australia_Pig**

**5101 . . . . . . . . . . . . . . . . . . . . . . . . . . . . . . . . . . . . . . . . . . . . . . . . . . . . . . . . . . . . . . . . . . . . . . . . . . . . . . . . . . . . . . . . . . . . . . . . . . . . MZ702743.1_India_Assam_Pig**

**5101 . . . . . . . . . . . . . . . . . . . . . . . . . . . . . . . . . . . . . . . . . . . . . . . . . . . . . . . . . . . . . . . . . . . . . . . . . . . . . . . . . . . . . . . . . . . . . . . . . . . . U15763.1_USA_Pig**

**A R G Y I A T K V E L G E A A A I F M T A T P P G T T D P F P D S N A P I H D L Q D E I P D R A W S S G Y E W I T E Y A G K T V W F V A S V K M G N E I A M C L Q R A G K K V I Q L N R K S Y D T E Y P Majority**

**------------------+-------------------+-------------------+-------------------+-------------------+-------------------+-------------------+-------------------+-------------------+-------------------+-**

**1810 1820 1830 1840 1850 1860 1870 1880 1890 1900**

**------------------+-------------------+-------------------+-------------------+-------------------+-------------------+-------------------+-------------------+-------------------+-------------------+-**

**5401 . . . . . . . . . . . . . . . . . . . . . . . . . . . . . . . . . . . . . . . . . . . . . . . . . . . . . . . . . . . . . . . . . . . . . . . . . . . . . . . . . . . . . . . . . . . . . . . . . . . . AB551990.1_Japan_Pig**

**5401 . . . . . . . . . . . . . . . . . . . . . . . . . . . . . . . . . . . . . . . . . . . . . . . . . . . . . . . . . D . . . . . . . . . . . . . . . . . . . . . . . . . . . . . . . . . . . . . . . . . . AY316157.1_Korea_Pig**

**5401 . . . . . . . . . . . . . . . . . . . . . . . . . . . . . . . . . . . . . . . . . . . T . . . . . . . . . . . . . . . . . . . . . . . . . . R . . . . . . . . . . . . . . . . . . . . . . . . . . . . . AY849939.1_China_Pig**

**5401 . . . . . . . . . . . . . . . . . . . . . . . . . . . . . . . . . . . . . . . . . . . . . . . . . . . . . . . . . . . . . . . . . . . . . . R . . . . . . . . . . . . . . . . . . . . . . . . . . . . . EF107523.1_China_Pig**

**5401 . . . . . . . . . . . . . . . . . . . . . . . . . . . . . . . . . . . . . . . . . . . . . . . . . . . . . . . . . G . . . . . . . . . . . . . . . . . . . . . . . . . . . . . . . . . . . . . . . . . . GQ902058.1_Thailand_Pig**

**5401 . . . . . . . . . . . . . . . . . . . . . . . . . . . . . . . . . . . . . . . . . . . . . . . . . . . . . . . . . . . . . . . . . . . . . . . . . . . . . . . . . . . . . . . . . . . . . . . . . . . . GQ902061.1_Thailand_Pig**

**5401 . . . . . . . . . . . . . . . . . . . . . . . . . . . . . . . . . . . . . . . . . . . . . . . . . . . . . . . . . . . . . . . . . . . . . . . . . . . . . . . . . . . . . . . . . . . . . . . . . . . . JN381872.1_China_Pig**

**5401 . . . . . . . . . . . . . . . . . . . . . . . . . . . . . . . . . . . . . . . . . . . . . . . . . . . . . . . . . . . . . . . . . . . . . . . . . . . . . . . . . . . . . . . . . . . . . . . . . . . . KC915016.1_China_Pig**

**5401 . . . . . . . . . . . . . . . . . . . . . . . . . . . . . . . . . . . . . . . . . . . . . . . . . . . . . . . . . . . . . . . . . . . . . . . . . . . . . . . . . . . . . . . . . . . . . . . . . . . . KF297916.1_China_Pig**

**5401 . . . . . . . . . . . . . . . . . . . . . . . . . . . . . . . . . . . . . . . . . . . . . . . . . . . . . . . . . . . . . . . . . . . . . . . . . . . . . . . . . . . . . . . . . . . . . . . . . . . . KF711994.1_South Korea_Pig**

**5401 . . . . . . . . . . . V . . . . . . K . V . . S . S . . . . . . . . . . . . . W P . . . . . . . . . . . . . . . . . . . . . . . . . . . . . . . . . . . . . . . . . . . . . . . . . . . . . . . . . . . KP164498.2_India_Pig**

**5401 . . . . . . . . . . . . . . . . . . . . . . . . . . . . . . . . . . . . . . . . . . . . . . . . . . . . . . . . . . . . . . . . . . . . . . . . . . . . . . . . . . . . . . . . . . . . . . . . . . . . KT447437.1_South Korea_Pig**

**5401 . . . . . . . . . . . . . . . . . . . . . . . . . . . . . . . . . . . . . . . . . . . . . . . . . . . . . . . . . . . . . . . . . . . . . . R . . . . . . . . . . . . . . . . . . . . . . . . . . . . . KU363309.1_China_Pig**

**5401 . . . . . . . . . . . . . . . . . . . . . . . . . . . . . . . . . . . . . . . . . . . . . . . . . . . . . . . . . . . . . . . . . . . . . . . . . . . . . . . . . . . . . . . . . . . . . . . . . . . . KX965684.1_China_Pig**

**5401 . . . . . . . . . . . . . . . . . . . . . . . . . . . . . . . . . . . . . . . . . . . . . . . . . . . . . . . . . D . . . . . . . . . . . . . . . . . . . . . . . . . . . R . . . . . . . . . . . . . . KY927818.1_Cambodia_Pig**

**5401 . . . . . . . . . . . . . . . . . . . . . . . . . . . . . . . . . . . . . . . . . . . . . . . . . . . . . . . . . . . . . . . . . . . . . . . . . . . . . . . . . . . . . . . . . . . . . . . . . . . . LC461960.1_Japan_Pig**

**5401 . . . . . . . . . . . . . . . . . . . . . . . . . . . . . . . . . . . . . . . . . . . . . . . . . . . . . . . . . D . . . . . . . . . . . . . . . . . . . . . . . . . . . . . . . . . . . . . . . . . . LC708276._Japan_Pig**

**5401 . . . . . . . . . . . . . . . . . . . . . . . . . . . . . . . . . . . . . . . . . . . . . . . . . . . . . . . . . D . . . . . . . . . . . . . . . . . . . . . . . . . . . . . . . . . . . . . . . . . . MN544780.1_China_Pig**

**5401 . . . . . . . . . . . . . . . . . . . . . . . . . . . . . . . . . . . . . . . . . . . . . . . . . . . . . . . . . D . . . . . . . . . . . . . . . . . . . . . . . . . . . . . . . . . . . . . . . . . . MT232844.1_India_Pig**

**5401 . . . . . . . . . . . . . . . . . . . . . . . . . . . . . . . . . . . . . . . . . . . . . . . . . . . . . . . . . . . S . . . . . . . . . . . . . . . . . . . . . . . . . . . . . . . . . . . . . . . . MT253737.1_Australia_Pig**

**5401 . . . . . . . . . . . . . . . . . . . . . . . . . . . . . . . . . . . . . . . . . . . . . . . . . . . . . . . . . D . . . . . . . . . . . . . . . . . . . . . . . . . . . . . . . . . . . . . . . . . . MZ702743.1_India_Assam_Pig**

**5401 . . . . . . . . . . . . . . . . . . . . . . . . . . . . . . . . . . . . . . . . . . . . . . . . . . . . . . . . . . . . . . . . . . . . . . . . . . . . . . . . . . . . . . . . . . . . . . . . . . . . U15763.1_USA_Pig**

**K C K N G D W D F V I T T D I S E M G A N F G A S R V I D C R K S V K P T I L E E G E G R V I L G N P S P I T S A S A A Q R R G R V G R N P N Q V G D E Y H Y G G A T S E D D S N L A H W T E A K I M L Majority**

**------------------+-------------------+-------------------+-------------------+-------------------+-------------------+-------------------+-------------------+-------------------+-------------------+-**

**1910 1920 1930 1940 1950 1960 1970 1980 1990 2000**

**------------------+-------------------+-------------------+-------------------+-------------------+-------------------+-------------------+-------------------+-------------------+-------------------+-**

**5701 . . . . . . . . . . . . . . . . . . . . . . . . . . . . . . . . . . . . . . . . . . . . . . . . . . . . . . . . . . . . . . . . . . . . . . . . . . . . . . . . . . . . . . . . . . . . . . . . . . . . AB551990.1_Japan_Pig**

**5701 . . . . . . . . . . . . . . . . . . . . . . . . . . . . . . . . . . . . . . . . K E . . . . . . . . . . . . . . . . . . . . . . . . . . . . . . . . . . . . . . . . . . . . . . . . . . . . . . . . . . AY316157.1_Korea_Pig**

**5701 . . . . . . . . . . . . . . . . . . . . . . . . . . . . . . . . . . . . . . . . . . . . . . . . . . . . . . . . . . . . . . . . . . . . . . . . . . . . . . . . . . . . . . . . . . . . . . . . . . . . AY849939.1_China_Pig**

**5701 . . . . . . . . . . . . . . . . . . . . . . . . . . . . . . . . . . . . . . . . . . . . . . . . . . . . . . . . . . . . . . . . . . . . . . . . . . . . . . . . . . . . . . . . . . . . . . . . . . . . EF107523.1_China_Pig**

**5701 . . . . . . . . . . . . . . . . . . . . . . . . . . . . . . . . . . . . . . . . . . . . . . . . . . . . . . . . . . . . . . . . . . . . . . . . . . . . . . . . . . . . . . . . . . . . . . . . . . . . GQ902058.1_Thailand_Pig**

**5701 . . . . . . . . . . . . . . . . . . . . . . . . . . . . . . . . . . . . . . . . . . . . . . . . . . . . . . . . . . . . . . . . . . . . . . . . . . . . . . . . . . . . . . . . . . . . . . . . . . . . GQ902061.1_Thailand_Pig**

**5701 . . . . . . . . . . . . . . . . . . . . . . . . . . . . . . . . . . . . . . . . . . . . . . . . . . . . . . . . . . . . . . . . . . . . . . . . . . . . . . . . . . . . . . . . . . . . . . . . . . . . JN381872.1_China_Pig**

**5701 . . . . . . . . . . . . . . . . . . . . . . . . . . . . . . . . . . . . . . . . . . . . . . . . . . . . . . . . . . . . . . . . . . . . . . . . . . . . . . . . . . . . . . . . . . . . . . . . . . . . KC915016.1_China_Pig**

**5701 . . . . . . . . . . . . . . . . . . . . . . . . . . . . . . . . . . . . . . . . . . . . . . . . . . . . . . . . . . . . . . . . . . . . . . . . . . . . . . . . . . . . . . . . . . . . . . . . . . . . KF297916.1_China_Pig**

**5701 . . . . . . . . . . . . . . . . . . . . . . . . . . . . . . . . . . . . . . . . . . . . . . . . . . . . . . . . . . . . . . . . . . . . . . . . . . . . . . . . . . . . . . . . . . . . . . . . . . . . KF711994.1_South Korea_Pig**

**5701 . . . . . . . . . . . . . . . . . . . . . . . . . . . . . . . . . . . . . . . . . . . . . . . . . . . . . . . . . . . . . . . . . . . . . . . . . . . . . . . . . . . . . . . . . . . . . . . . . . . . KP164498.2_India_Pig**

**5701 . . . . . . . . . . . . . . . . . . . . . . . . . . . . . . . . . . . . . . . . . . . . . . . . . . . . . . . . . . . . . . . . . . . . . . . . . . . . . . . . . . . . . G . . . . . . . . . . . . . . KT447437.1_South Korea_Pig**

**5701 . . . . . . . . . . . . . . . . . . . . . . . . . . . . . . . . . . . . . . . . . . . . . . . . . . . . . . . . . . . . . . . . . . . . . . . . . . . . . . . . . . . . . . . . . . . . . . . . . . . . KU363309.1_China_Pig**

**5701 . . . . . . . . . . . . . . . . . . . . . . . . . . . . . . . . . . . . . . . . . . . . . . . . . . . . . . . . . . . . . . . . . . . . . . . . . . . . . . . . . . . . . . . . . . . . . . . . . . . . KX965684.1_China_Pig**

**5701 . . . . . . . . . . . . . . . . . . . . . . . . . . . . . . . . . . . . . . . . . . . . . . . . . . . . . . . . . . . . . . . . . . . . . . . . . . . . . . . . . . . . . . . . . . . . . . . . . . . . KY927818.1_Cambodia_Pig**

**5701 . . . . . . . . . . . . . . . . . . . . . . . . . . . . . . . . . . . . . . . . . . . . . . . . . . . . . . . . . . . . . . . . . . . . . . . . . . . . . . . . . . . . . . . . . . . . . . . . . . . . LC461960.1_Japan_Pig**

**5701 . . . . . . . . . . . . . . . . . . . . . . . . . . . . . . . . . . . . . . . . . . . . . . . . . . . . . . . . . . . . . . . . . . . . . . . . . . . . . . . . . . . . . . . . . . . . . . . . . . . . LC708276._Japan_Pig**

**5701 . . . . . . . . . . . . . . . . . . . . . . . . . . . . . . . . . . . . . . . . . . . . . . . . . . . . . . . . . . . . . . . . . . . . . . . . . . . . . . . . . . . . . . . . . . . . . . . . . . . . MN544780.1_China_Pig**

**5701 . . . . . . . . . . . . . . . . . . . . . . . . . . . . . . . . . . . . . . . . . . . . . . . . . . . . . . . . . . . . . . . . . . . . . . . . . . . . . . . . . . . . . . . . . . . . . . . . . . . . MT232844.1_India_Pig**

**5701 . . . . . . . . . . . . . . . . . . . . . . . . . . . . . . . . . . . . . . . . . . . . . . . . . . . . . . . . . . . . . . . . . . . . . . . . . . . . . . . . . . . . . . . . . . . . . . . . . . . . MT253737.1_Australia_Pig**

**5701 . . . . . . . . . . . . . . . . . . . . . . . . . . . . . . . . . . . . . . . . . . . . . . . . . . . . . . . . . . . . . . . . . . . . . . . . . . . . . . . . . . . . . . . . . . . . . . . . . . . . MZ702743.1_India_Assam_Pig**

**5701 . . . . . . . . . . . . . . . . . . . . . . . . . . . . . . . . . . . . . . . . . . . . . . . . . . . . . . . . . . . . . . . . . . . . . . . . . . . . . . . . . . . . . . . . . . . . . . . . . . . . U15763.1_USA_Pig**

**D N I H M P N G L V A Q L Y G P E R E K A F T M D G E Y R L R G E E K K N F L E L L R T A D L P V W L A Y K V A S N G I Q Y T D R K W C F D G P R T N A I L E D N T E V E I V T R M G E R K I L K P R W Majority**

**------------------+-------------------+-------------------+-------------------+-------------------+-------------------+-------------------+-------------------+-------------------+-------------------+-**

**2010 2020 2030 2040 2050 2060 2070 2080 2090 2100**

**------------------+-------------------+-------------------+-------------------+-------------------+-------------------+-------------------+-------------------+-------------------+-------------------+-**

**6001 . . . . . . . . . . . . . . . . . . . . . . . . . . . . . . . . . . . . . . . . . . . . . . . . . . . . . . . . . . . . . . . . . . . . . . . . . . . . . . . . . . . . . . . . . . . . . . . . . . . . AB551990.1_Japan_Pig**

**6001 . . . . . . . . . . . . . . . . . . . . . . . . . . . . . . . . . . . . . . . . . . . . . . . . . . . . . . . . . . . . . . . . . . . . . . . . . . . . . . . . . . . . . . . . . . . . . . . . . . . . AY316157.1_Korea_Pig**

**6001 . . . . . . . . . . . . . . . . . . . . . S . . . . . . . . . . . . . . . . . . . . . . . . . . . . . . . . . . . . . . . . . . . . . . . . . . . . . . . . . . . . . . . . . . . . . . . . . . . . . . AY849939.1_China_Pig**

**6001 . . . . . . . . . . . . . . . . . . . . . S . . . . . . . . . . . . . . . . . . . . . . . . . . . . . . . . . . . . . . . . . . . . . . . . . . . . . . . . . . . . . . . . . . . . . . . . . . . . . . EF107523.1_China_Pig**

**6001 . . . . . . . . . . . . . . . . . . . . . . . . . . . . . . . . . . . . . . . . . . . . . . . . . . . . . . . . . . . . . . . . . . . . . . . . . . . . . . . . . . . . . . . . . . . . . . . . . . . . GQ902058.1_Thailand_Pig**

**6001 . . . . . . . . . . . . . . . . . . . . . . . . . . . . . . . . . . . . . . . . . . . . . . . . . . . . . . . . . . . . . . . . . . . . . . . . . . . . . . . . . . . . . . . . . . . . . . . . . . . . GQ902061.1_Thailand_Pig**

**6001 . . . . . . . . . . . . . . . . . . . . . . . . . . . . . . . . . . . . . . . . . . . . . . . . . . . . . . . . . . . . . . . . . . . . . . . . . . . . . . . . . . . . . . . . . . . . . . . . . . . . JN381872.1_China_Pig**

**6001 . . . . . . . . . . . . . . . . . . . . . . . . . . . . . . . . . . . . . . . . . . . . . . . . . . . . . . . . . . . . . . . . . . . . . . . . . . . . . . . . . . . . . . . . . . . . . . . . . . . . KC915016.1_China_Pig**

**6001 . . . . . . . . . . . . . . . . . . . . . . . . . . . . . . . . . . . . . . . . . . . . . . . . . . . . . . . . . . . . . . . . . . . . . . . . . . . . . . . . . . . . . . . . . . . . . . . . . . . . KF297916.1_China_Pig**

**6001 . . . . . . . . . . . . . . . . . . G . . . . . . . . . . . . . . . . . . L . . . . . . . . . . . . . . . . . . . . . . . . . . . . . . . . . . . . . . . . . . . . . . . . . . . . . . . . . . . . . . KF711994.1_South Korea_Pig**

**6001 . . . . . . . . . . . . . . . . . . . . . . . . . . . . . . . . . . . . . . . . . . . . . . . . . . . . . . . . . . . . . . . . . R . . . . . . . . . . . . . . . . . . . . . . . . . . . . . . . . . . KP164498.2_India_Pig**

**6001 . . . . . . . . . . . . . . . . . . . . . . . . . . . . . . . . . . . . . . . . . . . . . . . . . . . . . . . . . . . . . . . . . . . . . . . . . . . . . . . . . . . . . . . . . . . . . . . . . . . . KT447437.1_South Korea_Pig**

**6001 . . . . . . . . . . . . . . . . . . . . . S . . . . . . . . . . . . . . . . . . . . . . . . . . . . . . . . . . . . . . . . . . . . . . . . . . . . . . . . . . . . . . . . . . . . . . . . . . . . . . KU363309.1_China_Pig**

**6001 . . . . . . . . . . . . . . . . . . . . . . . . . . . . . . . . . . . . . . . . . . . . . . . . . . . . . . . . . . . . . . . . . . . . . . . . . . . . . . . . . . . . . . . . . . . . . . . . . . . . KX965684.1_China_Pig**

**6001 . . . . . . . . . . . . . . . . . . . . . . . . . . . . . . . . . . . . . . . . . . . . . . . . . . . . . . . . . . . . . . . . . . . . . . . . . . . . . . . . . . . . . . . . . . . . . . . . . . . . KY927818.1_Cambodia_Pig**

**6001 . . . . . . . . . . . . . . . . . . . . . Y . . . . . . . . . . . . . . . . . . . . . . . . . . . . . . . . . . . . . . . . . . . . . . . . . . . . . . . . . . . . . . . . . . . . . . . . . . . . . . LC461960.1_Japan_Pig**

**6001 . . . . . . . . . . . . . . . . . . . . . . . . . . . . . . . . . . . . . . . . . . . . . . . . . . . . . . . . . . . . . . . . . . . . . . . . . . . . . . . . . . . . . . . . . . . . . . . . . . . . LC708276._Japan_Pig**

**6001 . . . . . . . . . . . . . . . . . . . . . . . . . . . . . . . . . . . . . . . . . . . . . . . . . . . . . . . . . . . . . . . . . . . . . . . . . . . . . . . . . . . . . . . . . . . . . . . . . . . . MN544780.1_China_Pig**

**6001 . . . . . . . . . . . . . . . . . . . . . . . . . . . . . . . . . . . . . . . . . . . . . . . . . . . . . . . . . . . . . . . . . . . . . . . . . . . . . . . . . . . . . . . . . . . . . . . . . . . . MT232844.1_India_Pig**

**6001 . . . . . . . . . . . . . . . . . . . . . . . . . . . . . . . . . . . . . . . . . . . . . . . . . . . . . . . . . . . . . . . . . . . . . . . . . . . . . . . . . . . . . . . . . . . . . . . . . . . . MT253737.1_Australia_Pig**

**6001 . . . . . . . . . . . . . . . . . . . . . . . . . . . . . . . . . . . . . . . . . . . . . . . . . . . . . . . . . . . . . . . . . . . . . . . . . . . . . . . . . . . . . . . . . . . . . . . . . . . . MZ702743.1_India_Assam_Pig**

**6001 . . . . . . . . . . . . . . . . . . . . . . . . . . . . . . . . . . . . . . . . . . . . . . . . . . . . . . . . . . . . . . . . . . . . . . . . . . . . . . . . . . . . . . . . . . . . . . . . . . . . U15763.1_USA_Pig**

**L D A R V Y A D H Q A L K W F K D F A A G K R S A V S F I E V L G R M P E H F M G K T R E A L D T M Y L V A T A E K G G K A H R M A L E E L P D A L E T I T L I V A I T V M T G G F F L L M M Q R K G I Majority**

**------------------+-------------------+-------------------+-------------------+-------------------+-------------------+-------------------+-------------------+-------------------+-------------------+-**

**2110 2120 2130 2140 2150 2160 2170 2180 2190 2200**

**------------------+-------------------+-------------------+-------------------+-------------------+-------------------+-------------------+-------------------+-------------------+-------------------+-**

**6301 . . . . . . . . . . . . . . . . . . . . . . . . . I . . . . . . . . . . . . . . . . . . . . . . . . . . . . . . . . . . . . . . . . . . . . . . . . . . . . . . . . . . . . . . . . . . . . . . . . . . AB551990.1_Japan_Pig**

**6301 . . . . . . . . . . . . . . . . . . . . . . . . . . . . . . . . . . . . . . . . . . . . . . . . . . . . . . . . . . S . . . . . . . . . . . . . . . . . . . . . . . . . . . . R . . . . . . . . . . . . AY316157.1_Korea_Pig**

**6301 . . . . . . . . . . . . . . . . . . . . . . . . . . . . . . . . . . . . . . . . . . . . . . . . . . . . . . . . . . . . . . . . . . . . . . . . . . . . . . . . A . . . . . . . . . . . . . . . . . . . AY849939.1_China_Pig**

**6301 . . . . . . . . . . . . . . . . . . . . . . . . . . . . . . . . . . . . . . . . . . . . . . . . . . . . . . . . . . . . . . . . . . . . . . . . . . . . . . . . A . . . . . . . . . . . . . . . . . . . EF107523.1_China_Pig**

**6301 . . . . . . . . . . . . . . . . . . . . . . . . . . . . . . . . . . . . . . . . . . . . . . . . . . . . . . . . . . . . . . . . . . . . . . . . . . . . . . . . . . . . . . . . . . . . . . . . . . . . GQ902058.1_Thailand_Pig**

**6301 . . . . . . . . . . . . . . . . . . . . . . . . . . . . . . . . . . . . . . . . . . . . . . . . . . . . . . . . . . . . . . . . . . . . . . . . . . . . . . . . . . . . . . . . . . . . . . . . . . . . GQ902061.1_Thailand_Pig**

**6301 . . . . . . . . . . . . . . . . . . . . . . . . . I . . . . . . . . . . . . . . . . . . . . . . . . . . . . . . . . . . . . . . . . . . . . . . . . . . . . . . . . . . . . . . . . . . . . . . . . . . JN381872.1_China_Pig**

**6301 . . . . . . . . . . . . . . . . . . . . . . . . . . . . . . . . . . . . . . . . . . . . . . . . . . . . . . . . . . . . . . . . . . . . . . . . . . . . . . . . . . . . . . . . . . . . . . . . . . . . KC915016.1_China_Pig**

**6301 . . . . . . . . . . . . . . . . . . . . . . . . . . . . . . . . . . . . . . . . . . . . . . . . . . . . . . . . . . . . . . . . . . . . . . . . . . . . . . . . . . . . . . . . . . . . . . . . . . . . KF297916.1_China_Pig**

**6301 . . . . . . . . . . . . . . . . . . . . . . . . . I . . . . . . . . . . . . . . . . . . . . . . . . . . . . . . . . . . . . . . . . . . . . . . . . . . . . . . . . . . A . . . . . . . . . . . . . . . KF711994.1_South Korea_Pig**

**6301 . . . . . . . . . . . . . . . . . . . . . . . . . I . . . . . . . . . . . . . . . . . . . . . . . . . . . . . . . . . . . . . . . . . . . . . . . . . . . . . . . . . . . . . . . . . . . . . . . . . . KP164498.2_India_Pig**

**6301 . . . . . . . . . . . . . . . . . . . . . . . . . . . . . . . . . . . . . . . . . . . . . . . . . . . . . . . . . . . . . . . . . . . . . . . . . . . . . . . . . . . . . . . . . . . . . . . . . . . . KT447437.1_South Korea_Pig**

**6301 . . . . . . . . . . . . . . . . . . . . . . . . . . . . . . . . . . . . . . . . . . . . . . . . . . . . . . . . . . . . . . . . . . . . . . . . . . . . . . . . A . . . . . . . . . . . . . . . . . . . KU363309.1_China_Pig**

**6301 . . . . . . . . . . . . . . . . . . . . . . . . . . . . . . . . . . . . . . . . . . . . . . . . . . . . . . . . . . . . . . . . . . . . . . . . . . . . . . . . . . . . . . . . . . . . . . . . . . . . KX965684.1_China_Pig**

**6301 . . . . . . . . . . . . . . . . . . . . . . . . . . . . . . . . . . . . . . . . . . . . . . . . . . . . . . . . . . . . . . . . . . . . . . . . . . . . . . . . . . . . . . . . . . . . . . . . . . . . KY927818.1_Cambodia_Pig**

**6301 . . . . . . . . . . . . . . . . . . . . . . . . . . . . . . . . . . . . . . . . . . . . . . . . . . . . . . . . . . . . . . . . . . . . . . . . . . . . . . . . A . . . . . . . . . . . . . . . . . . . LC461960.1_Japan_Pig**

**6301 . . . . . . . . . . . . . . . . . . . . . . . . . . . . . . . . . . . . . . . . . . . . . . . . . . . . . . . . . . . . . . . . . . . . . . . . . . . . . . . . . . . . . . . . . . . . . . . . . . . . LC708276._Japan_Pig**

**6301 . . . . . . . . . . . . . . . . . . . . . . . . . . . . . . . . . . . . . . . . . . . . . . . . . . . . . . . . . . . . . . . . . . . . . . . . . . . . . . . . . . . . . . . . . . . . . . . . . . . . MN544780.1_China_Pig**

**6301 . . . . . . . . . . . . . . . . . . . . . . . . . . . . . . . . . . . . . . . . . . . . . . . . . . . . . . . . . . . . . . . . . . . . . . . . . . . . . . . . . . . . . . . . . . . . . . . . . . . . MT232844.1_India_Pig**

**6301 . . . . . . . . . . . . . . . . . . . . . . . . . . . . . . . . . . . . . . . . . . . . . . . . . . . . . . . . . . . . . . . . . . . . . . . . . . . . . . . . I . . . . . . . . . . . . . . . . . . . MT253737.1_Australia_Pig**

**6301 . . . . . . . . . . . . . . . . . . . . . . . . . . . . . . . . . . . . . . . . . . . . . . . . . . . . . . . . . . . . . . . . . . . . . . . . . . . . . . . . . . . . . . . . . . . . . . . . . . . . MZ702743.1_India_Assam_Pig**

**6301 . . . . . . . . . . . . . . . . . . . . . . . . . . . . . . . . . . . . . . . . . . . . . . . . . . . . . . . . . . . . . . . . . . . . . . . . . . . . . . . . . . . . . . . . . . . . . . . . . . . . U15763.1_USA_Pig**

**G K M G L G A L V L T L A T F F L W A A E V P G T K I A G T L L I A L L L M V V L I P E P E K Q R S Q T D N Q L A V F L I C V L T V V G V V A A N E Y G M L E K T K A D L K S M F G G K T Q A S G L T G Majority**

**------------------+-------------------+-------------------+-------------------+-------------------+-------------------+-------------------+-------------------+-------------------+-------------------+-**

**2210 2220 2230 2240 2250 2260 2270 2280 2290 2300**

**------------------+-------------------+-------------------+-------------------+-------------------+-------------------+-------------------+-------------------+-------------------+-------------------+-**

**6601 . . . . . . . . . . . . . . . . . . . . . . . . . . . . . . . . . . . . . . . . . . . . . . . . . . . . . . . . . . . . . . . . . . . . . . . . . . . . . . . . . . . . . . . . . V . . . . . . . . . . AB551990.1_Japan_Pig**

**6601 E . . . . . . . . . . . . . . . . . . . . . . . . . . . . . . . V . . . . . . . . . . . . . . . . . . . . . . . . . . . . . . . . . . . . . . . . . . . . . . . . . . . . . . . . . . R . . . . . . . . AY316157.1_Korea_Pig**

**6601 . . . . . . . . . . . . . . . . . . . . . . . . . . . . . . . . . . . . . . . . . . . . . . . . . . . . . . . . . . . . . . . . . . . . . . . . . . . . . . . . . . . . . . . . . . . . . . . . . . . . AY849939.1_China_Pig**

**6601 . . . . . . . . . . . . . . . . . . . . . . . . . . . . . . . . . . . . . . . . . . . . . . . . . L . . . . . . . . . . . . . . . . . . . . . . . . . . . . . . . . . . . . . . . . . . . . . . . . . . EF107523.1_China_Pig**

**6601 . . . . . . . . . . . . . . . . . . . . . . . . . . . . . . . . V . . . . . . . . . . . . . . . . . . . . . . . . . . . . . . . . . . . . . . . . . . . . . . . . . . . . . . . . . . R . . . P . . . . GQ902058.1_Thailand_Pig**

**6601 . . . . . . . . . . . . . . . . . . . . . . . . . . . . . . . . V . . . . . . . . . . . . . . . . . . . . . . . . . . . . . . . . . . . . . . . . . . . . . . . . . . . . . . . . . . R . . . P . . . . GQ902061.1_Thailand_Pig**

**6601 . . . . . . . . . . . . . . . . . . . . . . . . . . . . . . . . . . . . . . . . . . . . . . . . . . . . . . . . . . . . . . . . . . . . . . . . . . . . . . . . . . . . . . . . . . . . . . . . . . . . JN381872.1_China_Pig**

**6601 . . . . . . . . . . . . . . . . . . . . . . . . . . . . . . . . . . . . . . . . . . . . . . . . . . . . . . . . . . . . . . . . . . . . . . . . . . . . . . . . . . . . . . . . . . . . . . . . . . . . KC915016.1_China_Pig**

**6601 . . . . . . . . . . . . . . . . . . . . . . . . . . . . . . . . . . . . . . . . . . . . . . . . . . . . . . . . . . . . . . . . . . . . . . . . . . . . . . . . . . . . . . . . . . . . . . . . . . . . KF297916.1_China_Pig**

**6601 . . . . . . V . . . . . . . . . . . . . . . . . . . . . . . . . . . . . . . . . . . . . . . . . . . . . . . . . . . . . . . . . . . . . . . . . . . . . . . . . . . . . . . . . . V . . . . . . . . . . KF711994.1_South Korea_Pig**

**6601 . . . . . . . . . . . . . . . . . . . . . . . . . . . . . . . . . . . . . . . . . . . . . . . . . . . . N . . . . . . . . . . . . . . . . . . . . . . . . . . . . . . . . . . . . V . . . . . . . . . . KP164498.2_India_Pig**

**6601 . . . . . . . . . . . . . . . . . . . . . . . . . . . . . . . . . . . . . . . . . . . . . . . . . . . . . . . . . . . . . . . . . . . . . . . . . . . . . . . . . . . . . . . . . . . . . . . . . . . . KT447437.1_South Korea_Pig**

**6601 . . . . . . . . . . . . . . . . . . . . . . . . . . . . . . . . . . . . . . . . . . . . . . . . . . . . . . . . . . . . . . . . . . . . . . . . . . . . . . . . . . . . . . . . . . . . . . . . . . . . KU363309.1_China_Pig**

**6601 . . . . . . . . . . . . . . . . . . . . . . . . . . . . . . . . . . . . . . . . . . . . . . . . . . . . . . . . . . . . . . . . . . . . . . . . . . . . . . . . . . . . . . . . . . . . . . . . . . . . KX965684.1_China_Pig**

**6601 . . . . . . . . . . . . . . . . . . . . . . . . . . . . . . . . V . . . . . . . . . . . . . . . . . . . . . . . . . . . . . . . . . . . . . . . . . . . . . . . . . . . . . . . . . . R S . . P . . . . KY927818.1_Cambodia_Pig**

**6601 . . . . . . . . . . . . . . . . . . . . . . S . . . . . . . . . . . . . . . . . . . . . . . . . . . . . . . . . . . . . . . . . . . . . . . . . . . . . . . . . . . . . . . . . . A . . . . . . . . P . LC461960.1_Japan_Pig**

**6601 . . . . . . . . . . . . . . . . . . . . . . . . . . . . . . . . V . . . . . . . . . . . . . . . . . . . . . . . . . . . . . . . . . . . . . . . . . . . . . . . . . . . . . . . . . . R . . . . . . . . LC708276._Japan_Pig**

**6601 . . . . . . . . . . . . . . . . . . . . . . . . . . . . . . . . V . . . . . . . . . . . . . . . . . . . . . . . . . . . . . . . . . . . . . . . . . . . . . . . . . . . . . . . . . . R . . . P . . . . MN544780.1_China_Pig**

**6601 . . . . . . . . . . . . . . . . . . . . . . . . . . . . . . . . V . . . . . . . . . . . . . . . . . . . . . . . . . . . . . . . . . . . . . . . . . . . . . . R . . . . . . . . . . . R . . . P . . . . MT232844.1_India_Pig**

**6601 . . . . . . . . . . . . . . . . . . . . . . . . . . . . . . . . . . . . . . . . . . . . . . . . . . . . . . . . . . . . . . . . . . . . . . . . . . . . . . . R . . . . . . . . . . . . . . . P . . . . MT253737.1_Australia_Pig**

**6601 . . . . . . . . . . . . . . . . . . . . . . . . . . . . . . . . V . . . . . . . . . . . . . . . . . . . . . . . . . . . . . . . . . . . . . . . . . . . . . . . . . . . . . . . . . . R . . . P . . . . MZ702743.1_India_Assam_Pig**

**6601 . R . . . . . . . . . . . . . . . . . . . . . . . . . . . . . . . . . . . . . . . . . . . . . . . . . . . . . . . . . . . . . . . . . . . . . . . . . . . . . . . . . . . . . . . . . . . . . . . . . . U15763.1_USA_Pig**

**L P S M A L D L R P A T A W A L Y G G S T V V L T P L L K H L I T S E Y V T T S L A S I N S Q A G S L F V L P R G V P F T D L D L T V G L V F L G C W G Q I T L T T F L T A M V L A T L H Y G Y M L P G Majority**

**------------------+-------------------+-------------------+-------------------+-------------------+-------------------+-------------------+-------------------+-------------------+-------------------+-**

**2310 2320 2330 2340 2350 2360 2370 2380 2390 2400**

**------------------+-------------------+-------------------+-------------------+-------------------+-------------------+-------------------+-------------------+-------------------+-------------------+-**

**6901 . . . . . . . . . . . . . . . . . . . . . . . . . . . . . . . . . . . . . . . . . . . . . . . . . . . . . . . . . . . . . . . . . . . . . . . . . . . . . . . . . . . . . . . . . . . . . . . . . . . . AB551990.1_Japan_Pig**

**6901 . . . . . . . . . . . . . . . . . . . . . . . . . . . . . . . . . . . . . . P . . . . . S . . . . . . . . . . . . . . . . . . . . . . . . . . . . . . . . . . . . . . . . . . . . V . . . . . . . . . . AY316157.1_Korea_Pig**

**6901 . . . . . . . . . . . . . . . . . . . . . . . . . . . . . . . . . . . . . . . . . . . . . . . . . . . . . . . . . . . . . . . . . . . . . . . . . . . . . . . . . . . . . . . . . . . . . . . . . . . . AY849939.1_China_Pig**

**6901 . . . . . . . . . . . . . . . . . . . . . . . . . . . . . . . . . . . . . . . . . . . . . . . . . . . . . . . . . . . . . . . . . . . . . . . . . . . . . . . . . . . . . . . . . . . . . . . . . . . . EF107523.1_China_Pig**

**6901 . . . . . . . . . . . . . . . . . . . . . . . . . . . . . . . . . . . . . . . . . . . . S . . . . . . . . . . . . . . . . . . . . . . . . . . . . . . . . . . . . . . . . . . . . V . . . . . . . . . . GQ902058.1_Thailand_Pig**

**6901 . . . . . . . . . . . . . . . . . . . . . . . . . . . . . . . . . . . . . . . . . . . . S . . . . . . . . . . . . . . . . . . . . . . . . . . . . . . . . . . . . . . . . . . . . V . . . . . . . . . . GQ902061.1_Thailand_Pig**

**6901 . . . . . . . . . . . . . . . . . . . . . . . . . . . . . . . . . . . . . . . . . . . . . . . . . . . . . . . . . . . . . . . . . . . . . . . . . . . . . . . . . . . . . . . . . . . . . . . . . . . . JN381872.1_China_Pig**

**6901 . . . . . . . . . . . . . . . . . . . . . . . . . . . . . . . . . . . . . . . . . . . . . . . . . . . . . . . . . . . . . . . . . . . . . . . . . . . . . . . . . . . . . . . . . . . . . . . . . . . . KC915016.1_China_Pig**

**6901 . . . . . . . . . . . . . . . . . . . . . . . . . . . . . . . . . . . . . . . . . . . . . . . . . . . . . . . . . . . . . . . . . . . . . . . . . . . . . . . . . . . . . . . . . . . . . . . . . . . . KF297916.1_China_Pig**

**6901 . . . . . . . . . . . . . . . . . . . . . . . . . . . . . . . . . . . . . . . . . . . . . . . . . . . . . . . . . . . . . . . . . . . . . . . . . . . . . . . . . . . . . . . F . . . . . . . . . . . . KF711994.1_South Korea_Pig**

**6901 . . . . . . . . . . . . . . . . . . . . . . . . . . . . . . . . . . . . . . . . . . . . . . . . . . . . . . . . . . . . . . . . . . . . . . . . . . . . . . . . . . . . . . . . . . . . . . . . . . . . KP164498.2_India_Pig**

**6901 . . . . . . . . . . . . . . . . . . . . . . . . . . . . . . . . . . . . . . . . . . . . . . . . . . . . . . . . . . . . . . . . . . . . . . . . . . . . . . . . . . . . . . . . . . . . . . . . . . . . KT447437.1_South Korea_Pig**

**6901 . . . . . . . . . . . . . . . . . . . . . . . . . . . . . . . . . . . . . . . . . . . . . . . . . . . . . . . . . . . . . . . . . . . . . . . . . . . . . . . . . . . . . . . . . . . . . . . . . . . . KU363309.1_China_Pig**

**6901 . . . . . . . . . . . . . . . . . . . . . . . . . . . . . . . . . . . . . . . . . . . . . . . . . . . . . . . . . . . . . . . . . . . . . . . . . . . . . V . . . . . . . . . . . . . . . . . . . . . . KX965684.1_China_Pig**

**6901 . . . . . . . . . . . . . . . . . . . . . . . . . . . . . . . . . . . . . . . . . . . . S . . . . . . . . . . . . . . . . . . . . . . . . . . . . . . . . . . . . . . . . . . . . V . . . . . . . . . . KY927818.1_Cambodia_Pig**

**6901 . . G . . . . . . . . . . . . . . . . . . . . . . . . . . . . . . . . . . . . . . . . . . . . . . . . . . . . . . . . . . . . . . . . . . . . . . . . . . . . . . . . . . . . . . . . . . . . . . . . . LC461960.1_Japan_Pig**

**6901 . . . . . . . . . . . . . . . . . . . . . . . . . . . . . . . . . . . . . . . . . . . . S . . . . . . . . . . . . . . . . . . . . . . . . . . . . . . . . . . . . . . . . . . . . V . . . . . . . . . . LC708276._Japan_Pig**

**6901 . . . . . . . . . . . . . . . . . . . . . . . . . . . . . . . . . . . . . . . . . . . . S . . . . . . . . . . . . . . . . . . . . . . . . . . . . . . . . . . . . . . . . . . . . V . . . . . . . . . . MN544780.1_China_Pig**

**6901 . . . . . . . . . . . . . . . . . . . . . . . . . . . . . . . . . . . . . . . . . . . . S . . . . . . . . . . . . . . . . . . . . . . . . . . . . . . . . . . . . . . . . . . . . V . . . . . . . . . . MT232844.1_India_Pig**

**6901 . . . . . . . . . . . . . . . . . . . . . . . . . . . . . . . . . . . . . . . . . . . . S . . . . . . . . . . . . . . . . . . . . . . . . . . . . . . . . . . . . . . . . . . . . V . . . . . . . . . . MT253737.1_Australia_Pig**

**6901 . . . . . . . . . . . . . . . . . . . . . . . . . . . . . . . . . . . . . . . . . . . . S . . . . . . . . . . . . . . . . . . . . . . . . . . . . . . . . . . . . . C . . . . . . V . . . . . . . . . . MZ702743.1_India_Assam_Pig**

**6901 . . . . . . . . . . . . . . . . . . . . . . . . . . . . . . . . . . . . . . . . . . . . . . . . . . . . . . . . . . . . . . . . . . . . . . . . . . . . . V . . . . . . . . . . . . . . . . . . . . . . U15763.1_USA_Pig**

**W Q A E A L R A A Q R R T A A G I M K N A V V D G M V A T D V P E L E R T T P L M Q K K V G Q V L L I G V S V A A F L V N P N V T T V R E A G V L V T A A T L T L W D N G A S A V W N S T T A T G L C H Majority**

**------------------+-------------------+-------------------+-------------------+-------------------+-------------------+-------------------+-------------------+-------------------+-------------------+-**

**2410 2420 2430 2440 2450 2460 2470 2480 2490 2500**

**------------------+-------------------+-------------------+-------------------+-------------------+-------------------+-------------------+-------------------+-------------------+-------------------+-**

**7201 . . . . . . . . . . . . . . . . . . . . . . . . . . . . . . . . . . . . . . . . . . . . . . . . . . . . . . . . . . . . . . . . . . . . . . . . . . . . . . . . . . . . . . . . . . . . . . . . . . . . AB551990.1_Japan_Pig**

**7201 . . . . . . . . . . . . . . . . . . . . . . . . . . . . . . . . . . . . . . . . . . . . . . . . . . . . . . . . . . . . . . . . S . . . K . . . . . . . . . . . . . . . . . . . . . . . . . . . . . . . AY316157.1_Korea_Pig**

**7201 . . . . . . . . . . . . . . . . . . . . . . . . . . . . . . . . . . . . . . . . . . . . . . . . . . . . . . . . . . . . . . . . . . . . . . . . . . . . . . . . . . . . . . . . . . . . . . . . . . . . AY849939.1_China_Pig**

**7201 . . . . . . . . . . . . . . . . . . . . . . . . . . . . . . . . . . . . . . . . . . . . . . . . . . . . . . . . . . . . . . . . . . . . . . . . . . . . . . . . . . . . . . . . . . . . . . . . . . . . EF107523.1_China_Pig**

**7201 . . . . . . . . . . . . . . . . . . . . . . . . . . . . . . . . . . . . . . . . . . . . . . . . . . . . . . . . . . . . . . . . . . . . . . . . . . . . . . . . . . . . . . . . . . . . . . . . . . . . GQ902058.1_Thailand_Pig**

**7201 . . . . . . . . . . . . . . . . . . . . . . . . . . . . . . . . . . . . . . . . . . . . . . . . . . . . . . . . . . . . . . . . . . . . . . . . . . . . . . . . . . . . . . . . . . . . . . . . . . . . GQ902061.1_Thailand_Pig**

**7201 . . . . . . . . . . . . . . . . . . . . . . . . . . . . . . . . . . . . . . . . . . . . . . . . . . . . . . . . . . . . . . . . . . . . . . . . . . . . . . . . . . . . . . . . . . . . . . . . . . . . JN381872.1_China_Pig**

**7201 . . . . . . . . . . . . . . . . . . . . . . . . . . . . . . . . . . . . . . . . . . . . . . . . . . . . . . . . . . . . . . . . . . . . . . . . . . . . . . . . . . . . . . . . . . . . . . . . . . . . KC915016.1_China_Pig**

**7201 . . . . . . . . . . . . . . . . . . . . . . . . . . . . . . . . . . . . . . . . . . . . . . . . . . . . . . . . . . . . . . . . . . . . . . . . . . . . . . . . . . . . . . . . . . . . . . . . . . . . KF297916.1_China_Pig**

**7201 . . . . . . . . . . . . . . . . . . . . . . . . . . . . . . . . . . . . . . . . . . . . . . . . . . . . . . . . . . . . . . . . . . . . . . . . . . . . . . . . . . . . . . . . . . . . . . . . . . . . KF711994.1_South Korea_Pig**

**7201 . . . . . . . . . . . . . . . . . . . . . . . . . . . . . . . . . . . . . . . . . . . . . . . . . . . . . . . . . . . . . . . . . . . . . . . . . . . . . . . . . . . . . . . . . . . . . . . . . . . . KP164498.2_India_Pig**

**7201 . . . . . . . . . . . . . . . . . . . . . . . . . . . . . . . . . . . . . . . . . . . . . . . . . . . . . . . . . L . . . . . . . . . . . . . . . . . . . . . . . . . . . . . . . . . . . . . . . . . . KT447437.1_South Korea_Pig**

**7201 . . . . . . . . . . . . . . . . . . . . . . . . . . . . . . . . . . . . . . . . . . . . . . . . . . . . . . . . . . . . . . . . . . . . . . . . . . . . . . . . . . . . . . . . . . . . . . . . . . . . KU363309.1_China_Pig**

**7201 . . . . . . . V . . . . . . . . . . . . . . . . . . . . . . . . . . . . . . . . . . . . . . . . . . . . . . . . . . . . . . . . . . . . . . . . . . . . . . . . . . . . . . . . . . . . . . . . . . . . KX965684.1_China_Pig**

**7201 . . . . . . . . . . . . . . . . . . . . . . . . . . . . . . . . . . . . . . . . . . . . . . . . . . . . . . . . . . . . . . . . . . . . . . . . . . . . . . . . . . . . . . . . . . . . . . . . . . . . KY927818.1_Cambodia_Pig**

**7201 . . . . . . . . . . . . . . . . . . . . . . . . . . . . . . . . . . . . . . . . . . . . . . . . . . . . . . I . . . . . . . S . . . . . . . . . . . . . . . . . . . . . . . . . . . . . . . . . . . . . LC461960.1_Japan_Pig**

**7201 . . . . . . . . . . . . . . . . . . . . . . . . . . . . . . . . . . . . . . . . . . . . . . . . . . . . . . . . . . . . . . . . . . . . . . . . . . . . . . . . . . . . . . . . . . . . . . . . . . . . LC708276._Japan_Pig**

**7201 . . . . . . . . . . . . . . . . . . . . . . . . . . . . . . . . . . . . . . . . . . . . . . . . . . . . . . . . . . . . . . . . . . . . . . . . . . . . . . . . . . . . . . . . . . . . . . . . . . . . MN544780.1_China_Pig**

**7201 . . . . . . . . . . . . . . . . . . . . . . . . . . . . . . . . . . . . . . . . . . . . . . . . . . . . . . . . . . . . . . . . . . . . . . . . . . . . . . . . . . . . . . . . . . . . . . . . . . . . MT232844.1_India_Pig**

**7201 . . . . . . . . . . . . . . . . . . . . . . . . . . . . . . . . . . . . . . . . . . . . . . . . . . . . . . . . . . . . . . . . . . . . . . . . . . . . . . . . . . . . . . . . . . . . . . . . . . . . MT253737.1_Australia_Pig**

**7201 . . . . . . . . . . . . . . . . . . . . . . . . . . . . . . . . . . . . . . . . . . . . . . . . . . . . . . . . . . . . . . . . . . . . . . . . . . . . . . . . . . . . . . . . . . . . . . . . . . . . MZ702743.1_India_Assam_Pig**

**7201 . . . . . . . . . . . . . . . . . . . . . . . . . . . . . . . . . . . . . . . . . . . . . . . . . . . . . . . . . . . . . . . . . . . . . . . . . . . . . . . . . . . . . . . . . . . . . . . . . . . . U15763.1_USA_Pig**

**V M R G S Y L A G G S I A W T L I K N A D K P S L K R G R P G G R T L G E Q W K E K L N A M S R E E F F K Y R R E A I I E V D R T E A R R A R R E N N I V G G H P V S R G S A K L R W L V E K G F V S P Majority**

**------------------+-------------------+-------------------+-------------------+-------------------+-------------------+-------------------+-------------------+-------------------+-------------------+-**

**2510 2520 2530 2540 2550 2560 2570 2580 2590 2600**

**------------------+-------------------+-------------------+-------------------+-------------------+-------------------+-------------------+-------------------+-------------------+-------------------+-**

**7501 . . . . . . . . . . . . . . . . . . . . . . . . . . . . . . . . . . . . . . . . . . . . . . . . . . . . . . . . . . . . . . . . . . . . . . . . . . . . . . . . . . . . . . . . . . . . . . . . . . . . AB551990.1_Japan_Pig**

**7501 . . . . . . . . . . . . . . . . . . . . . . . . . . . . . . . . . . . . . . . . . . . . . . . . D . . . . . . . . . . . . . . . . . . . . . . . . . . . . . . . . . . . . . . . . . . . . . . . . . . . AY316157.1_Korea_Pig**

**7501 . . . . . . . . . . . . . . . . . . . . . . . . . . . . . . . . . . . . . . . . . . . . . . . . . . . . . . . . . . . . . . . . . . . . . . . . . . . . . . . . . . . . . . . . . . . . . . . . . . . . AY849939.1_China_Pig**

**7501 . . . . . . . . . . . . . . . . . . . . . . . . . . . . . . . . . . . . . . . . . . . . . . . . . . . . . . . . . . . . . . . . . . . . . . . . . . . . . . . . . . . . . . . . . . . . . . . . . . . . EF107523.1_China_Pig**

**7501 . . . . . . . . . . . . . . . . . . . . . . . . . . . . . . . . . . . . . . . . . . . . . . . . D . . . . . . . . . . . . . . . . . . . . . . . . . . . . . . . . . . . . . . . . . . . . . . . . . . . GQ902058.1_Thailand_Pig**

**7501 . . . . . . . . . . . . . . . . . . . . . . . . . . . . . . . . . . . . . . . . . . . . . . . . D . . . . . . . . . . . . . . . . . . . . . . . . . . . . . . . . . . . . . . . . . . . . . . . . . . . GQ902061.1_Thailand_Pig**

**7501 . . . . . . . . . . . . . . . . . . . . . . . . . . . . . . . . . . . . . . . . . . . . . . . . . . . . . . . . . . . . . . . . . . . . . . . . . . . . . . . . . . . . . . . . . . . . . . . . . . . . JN381872.1_China_Pig**

**7501 . . . . . . . . . . . . . . . . . . . . . . . . . . . . . . . . . . . . . . . . . . . . . . . . . . . . . . . . . . . . . . . . . . . . . . . . . . . . . . . . . . . . . . . . . . . . . . . . . . . . KC915016.1_China_Pig**

**7501 . . . . . . . . . . . . . . . . . . . . . . . . . . . . . . . . . . . . . . . . . . . . . . . . . . . . . . . . . . . . . . . . . . . . . . . . . . . . . . . . . . . . . . . . . . . . . . . . . . . . KF297916.1_China_Pig**

**7501 . . . . . . . . . . . . . . . . . . . . . . . . . . . . . . . . . . . . . . . . . . . . . . . . . . . . . . . . . . . . . . . . . . . . . . . . . . . . . . . . . . . . . . . . . . . . . . . . . . . . KF711994.1_South Korea_Pig**

**7501 . . . . . . . . . . . . V . . . . . . . . . . . . . . . . . . . . . . . . . . . . . . . . . . . . . . . . . . . . V . . . . . . . . . . . . . . . . . . . . . . . . . . . . . . . . . . . . . . . . . . KP164498.2_India_Pig**

**7501 . . . . . . . . . . . . . . . . . . . . . . . . . . . . . . . . . . . . . . . . . . . . . . . . . . . . Q . . . . . . . . . . . . . . . . . . . . . . . . . . . . . . . . . . . . . . . . . . . . . . . KT447437.1_South Korea_Pig**

**7501 . . . . . . . . . . . . . . . . . . . . . . . . . . . . . . . . . . . . . . . . . . . . . . . . . . . . . . . . . . . . . . . . . . . . . . . . . . . . . . . . . . . . . . . . . . . . . . . . . . . . KU363309.1_China_Pig**

**7501 . . . . . . . . . . . . . . . . . . . . . . . . . . . . . . . . . . . . . . . . . . . . . . . . . . . . . . . . . . . . . . . . . . . . . . . . . . . . . . . . . . . . . . . . . . . . . . . . . . . . KX965684.1_China_Pig**

**7501 . . . . . . . . . . . . . . . . . . . . . . . . . . . . . . . . . . . . . . . . . . . . . . . . D . . . . . . . . . . T . . . . . . . . . . . . . . . . . . . . . . . . . . . . . . . . . . . . . . . . KY927818.1_Cambodia_Pig**

**7501 . . . . . . . . . . . . . . . . . . . . . . . . . . . . . . . . . . . . . . . . . . . . . . . . . . . . . . . . . . . T . . . . . . . . . . . . . . . . . . . . . . . . . . . . . . . . . . . . . . . . LC461960.1_Japan_Pig**

**7501 . . . . . . . . . . . . . . . . . . . . . . . . . . . . . . . . . . . . . . . . . . . . . . . . D . . . . . . . . . . . . . . . . . . . . . . . . . . . . . . . . . . . . . . . . . . . . . . . . . . . LC708276._Japan_Pig**

**7501 . . . . . . . . . . . . . . . . . . . . . . . . . . . . . . . . . . . . . . . . . . . . . . . . D . . . . . . . . . . . . . . . . . . . . . . . . . . . . . . . . . . . . . . . . . . . . . . . . . . . MN544780.1_China_Pig**

**7501 . . . . . . . . . . . . . . . . . . . . . . . . . . . . . . . . . . . . . . . . . . . . . . . . D . . . . . . . . . . . . . . . . . . . . . . . . . . . . . . . . . . . . . . . . . . . . . . . . . . . MT232844.1_India_Pig**

**7501 . . . . . . . . . . . . . . . . . . . . . . . . . . . . . . . . . . . . . . . . . . . . . . . . D . . . . . . . . . . . . . . . . . . . . . . . . . . . . . . . . . . . . . . . . . . . . . . . . . . . MT253737.1_Australia_Pig**

**7501 . . . . . . . . . . . . . . . . . . . . . . . . . . . . . . . . . . . . . . . . . . . . . . . . D . . . . . . . . . . . . . . . . . . . . . . . . . . . . . . . . . . . . . . . . . . . . . . . . . . . MZ702743.1_India_Assam_Pig**

**7501 . . . . . . . . . . . . . . . . . . . . . . . . . . . . . . . . . . . . D . . . . . . . . . . . . . . . . . . . . . . . . . . . . . . . . . . . . . . . . . . . . . . . . . . . . . . . . . . . . . . . U15763.1_USA_Pig**

**I G K V I D L G C G R G G W S Y Y A A T L K K V Q E V R G Y T K G G A G H E E P M L M Q S Y G W N L V S L K S G V D V F Y K P S E P S D T L F C D I G E S S P S P E V E E Q R T L R V L E M T S D W L H Majority**

**------------------+-------------------+-------------------+-------------------+-------------------+-------------------+-------------------+-------------------+-------------------+-------------------+-**

**2610 2620 2630 2640 2650 2660 2670 2680 2690 2700**

**------------------+-------------------+-------------------+-------------------+-------------------+-------------------+-------------------+-------------------+-------------------+-------------------+-**

**7801 . . . . . . . . . . . . . . . . . . . . . . . . . . . . . . . . . . . . . . . . . . . . . . . . . . . . . . . . . . . . . . . . . . . . . . . . . . . . . . . . . . . . . . . . . . . . . . . . . . . . AB551990.1_Japan_Pig**

**7801 . . . . . . . . . . . . S . N . . . . . . . . . . . . K . . . . . . . . . . . . . . . . . . . R . . . . . . . . . . . . . . . . . . . . . . . . . . . . . . . . . . . . . . . . . . . . . . . . . . . . AY316157.1_Korea_Pig**

**7801 . . . . . . . . . . . . . . . . C . . . . . . . . . . . . . . . . . . . . . . . . . . . . . . . . . . . . . . . . . . . . . . . . . . . . . . . . . . . . . . . . . . . . . . . . . . . . . . . . . . . AY849939.1_China_Pig**

**7801 . . . . . . . . . . . . . . . . . . . . . . . . . . . . . . . . . . . . . . . . . . . . . . . . . . . . . . . . . . . . . . . . . . . . . . . . . . . . . . . . . . . . . . . . . . . . . . . . . . . . EF107523.1_China_Pig**

**7801 . . . . . . . . . . . . . . . . . . . . . . . . . . . K . . . . . . . . . . . . . . . . . . . . . . . . . . . . . . . . . . . . . . . . . . . . . . . . . . . . . . . . . . . . . . . . . . . . . . . . GQ902058.1_Thailand_Pig**

**7801 . . . . . . . . . . . . . . . . . . . . . . . . . . . K . . . . . . . . . . . . . . . . . . . . . . . . . . . . . . . . . . . . . . . . . . . . . . . . . . . . . . . . . . . . . . . . . . . . . . . . GQ902061.1_Thailand_Pig**

**7801 . . . . . . . . . . . . . . . . . . . . . . . . . . . . . . . . . . . . . . . . . . . . . . . . . . . . . . . . . . . . . . . . . . . . . . . . . . . . . . . . . . . . . . . . . . . . . . . . . . . . JN381872.1_China_Pig**

**7801 . . . . . . . . . . . . . . . . . . . . . . . . . . . . . . . . . . . . . . . . . . . . . . . . . . . . . . . . . . . . F . . . . . . . . . . . . . . . . . . . . . . . . . . . . . . . . . . . . . . . KC915016.1_China_Pig**

**7801 . . . . . . . . . . . . . . . . . . . . . . . . . . . . . . . . . . . . . . . . . . . . . . . . . . . . . . . . . . . . F . . . . . . . . . . . . . . . . . . . . . . . . . . . . . . . . . . . . . . . KF297916.1_China_Pig**

**7801 . . . . . . . . . . . . . . . . . . . . . . . . . . . . . . . . . . . . . . . . . . . . . . . . . . . . . . . . . . . . . . . . . . . . . . . . . . . . . . . . . . . . . . . . . . . . . . . . . . . . KF711994.1_South Korea_Pig**

**7801 . . . . . . . . . . . . . . . . . . . . . . . . . . . . . . . . . . . . . . . . . . . . . . . . . . . . . . . . . . . . . . . . . . . . . . . . . . . . . . . . . . . . . . . . . . . . . . . . . . . . KP164498.2_India_Pig**

**7801 . . . . . . . . . . . . . . . . . . . . . . . . . . . . . . . . . . . . . . . . . . . . . . . . . . . . . . . . . . . . . . . . . . . . . . . . . . . . . . . . . . . . . . . . . . . . . . . . . . . . KT447437.1_South Korea_Pig**

**7801 . . . . . . . . . . . . . . . . . . . . . . . . . . . . . . . . . . . . . . . . . . . . . . . . . . . . . . . . . . . . . . . . . . . . . . . . . . . . . . . . . . . . . . . . . . . . . . . . . . . . KU363309.1_China_Pig**

**7801 . . . . . . . . . . . . . . . . . . . . . . . . . . . . . . . . . . . . . . . . . . . . . . . . . . . . . . . . . . . . . . . . . . . . . . . . . . . . . . . . . . . . . . . . . . . . . . . . . . . . KX965684.1_China_Pig**

**7801 . . . . . . . . . . . . . . . . . . . . . . . . . . . K . . . . . . . . . . . . . . . . . . . . . . . . . . . . . . . . . . . . . . . . . . . . . . . . . . . . . . . . . . . . . . . . . . . . . . . . KY927818.1_Cambodia_Pig**

**7801 . . . . . . . . . . . . . . . . . . . . . . . . . . . . . . . . . . . . . . . . . . . . . . . . . . . . . . . . . . . . . . . . . . . . . . . . . . . . . . . . . . . . . . . . . . . . . . . . . . . . LC461960.1_Japan_Pig**

**7801 . . . . . . . . . . . . . . . . . . . . . . . . . . . K . . . . . . . . . . . . . . . . . . . . . . . . . . . . . . . . . . . . . . . . . . . . . . . . . . . . . . . . . . . . . . . . . . . . . . . . LC708276._Japan_Pig**

**7801 . . . . . . . . . . . . . . . . . . . . . . . . . . . K . . . . . . . . . . . . . . . . . . . . . . . . . . . . . . . . . . . . . . . . . . . . . . . . . . . . . . . . . . . . . . . . . . . . . . . . MN544780.1_China_Pig**

**7801 . . . . . . . . . . . . . . . . . . . . . . . . . . . . . . . . . . . . . . . . . . . . . . . . . . . . . . . . . . . . . . . . . . . . . . . . . . . . . . . . . . . . . . . . . . . . . . . . . . . . MT232844.1_India_Pig**

**7801 . . . . . . . . . . . . . . . . . . . . . . . . . . . K . . . . . . . . . . . . . . . . . . . . . . . . . . . . . . . . . . . . . . . . . . . . . . . . . . . . . D . . . . . . . . . . . . . . . . . . MT253737.1_Australia_Pig**

**7801 . . . . . . . . . . . . . . . . . . . . . . . . . . . K . . . . . . . . . . . . . . . . . . . . . . . . . . . . . . . . . . . . . . . . . . . . . . . . . . . . . . . . . . . . . . . . . . . . . . . . MZ702743.1_India_Assam_Pig**

**7801 . . . . . . . . . . . . . . . . . . . . . . . . . . . . . . . . . . . . . . . . . . . . . . . . . . . . . . . . . . . . . . . . . . . . . . . . . . . . . . . . . . . . . . . . . . . . . . . . . . . . U15763.1_USA_Pig**

**R G P R E F C I K V L C P Y M P K V I E K M E V L Q R R F G G G L V R L P L S R N S N H E M Y W V S G A A G N V V H A V N M T S Q V L L G R M D R T V W R G P K Y E E D V N L G S G T R A V G K G E V H Majority**

**------------------+-------------------+-------------------+-------------------+-------------------+-------------------+-------------------+-------------------+-------------------+-------------------+-**

**2710 2720 2730 2740 2750 2760 2770 2780 2790 2800**

**------------------+-------------------+-------------------+-------------------+-------------------+-------------------+-------------------+-------------------+-------------------+-------------------+-**

**8101 . . . . . . . . . . . . . . . . . . . . . . . . . . . . . . . . . . . . . . . . . . . . . . . . . . . . . . . . . . . . . . . . . . . . . . . . . . . . . . . . . . . . . . . . . . . . . . . . . . . . AB551990.1_Japan_Pig**

**8101 . . . . . . . . . . . . . . . . . . . . . N . . . . . . . . . . . . . . . . . . . . . . . . . . . . . . . . . . . . . . . . . . . . . . . . . . . . . . . . . . . K . . . . . . . . . . . . . . . . . . AY316157.1_Korea_Pig**

**8101 . . . . . . . . . . . . . . . . . . . . . . . . . . . . . . . . . . . . . . . . . . . . . . . . . . . . . . . . . . . . . . . . . . . . . . . . . . . . . . . . . . . . . . . . . . . . . . . . . . . . AY849939.1_China_Pig**

**8101 . . . . . . . . . . . . . . . . . . . . . . . . . . . . . . . . . . . . . . . . . . . . . . . . . . . . . . . . . . . . . . . . . . . . . . . . . . . . . . . . . . . . . . . . . . . . . . . . . . . . EF107523.1_China_Pig**

**8101 . . . . . . . . . . . . . . . . . . . . . . . . . . . . . . . . . . . . . . . . . . . . . . . . . . . . . . . . . . . . . . . . . . . . . . . . . . . . . . . . . . . . . . . . . . . . . . . . . . . . GQ902058.1_Thailand_Pig**

**8101 . . . . . . . . . . . . . . . . . . . . . . . . . . . . . . . . . . . . . . . . . . . . . . . . . . . . . . . . . . . . . . . . . . . . . . . . . . . . . . . . . . . . . . . . . . . . . . . . . . . . GQ902061.1_Thailand_Pig**

**8101 . . . . . . . . . . . . . . . . . . . . . . . . . . . . . . . . . . . . . . . . . . . . . . . G . . . . . . . . . . . . . . . . . . . . . . . . . . . . . . . . . . . . . . . . . . . . . . . . . . . . JN381872.1_China_Pig**

**8101 . . . . . . . . . . . . . . . . . . . . . . . . . . . . . . . . . . . . . . . . . . . . . . . . . . . . . . . . . . . . . . . . . . . . . . . . . . . . . . . . . . . . . . . . . . . . . . . . . . . . KC915016.1_China_Pig**

**8101 . . . . . . . . . . . . . . . . . . . . . . . . . . . . . . . . . . . . . . . . . . . . . . . . . . . . . . . . . . . . . . . . . . . . . . . . . . . . . . . . . . . . . . . . . . . . . . . . . . . . KF297916.1_China_Pig**

**8101 . . . . . . . . . . . . . . . . . . . . . . . . . . . . . . . . . L . . . . . . . . . . . . . . . . . . . . . . . . . . . . . . . . . . . . . . . . . . . . . . . . . . . . . . . . . . . . . . . . . . KF711994.1_South Korea_Pig**

**8101 . . . . . . . . . . . . . . . . . . . . . . . . . . . . . . . . . . . . . . . . . . . . . . . . . . . . . . . . . . . . . . . . . . . . . . . . . . . . . . . . . . . . . . . . . . . . . . . . . . . . KP164498.2_India_Pig**

**8101 . . . . . . . . . . . . . . . . . . . . . . . . . . . . . . . . . . . . . . . . . . . . . . . . . . . . . . . . . . . . . . . . . . . . . . . . . . . . . . . . . . . . . . . . . . . . . . . . . . . . KT447437.1_South Korea_Pig**

**8101 . . . . . . . . . . . . . . . . . . . . . . . . . . . . . . . . . . . . . . . . . . . . . . . . . . . . . . . . . . . . . . . . . . . . . . . . . . . . . . . . . . . . . . . . . . . . . . . . . . . . KU363309.1_China_Pig**

**8101 . . . . . . . . . . . . . . . . . . . . . . . . . . . . . . . . . . . . . . . . . . . . . . . . . . . . . . . . . . . . . . . . . . . . . . . . . . . . . . . . . . . . . . . . . . . . . . . . . . . . KX965684.1_China_Pig**

**8101 . . . . . . . . . . . . . . . . . . . . . . . . . . . . . . . . . . . . . . . . . . . . . . . . . . . . . . . . . . . . . . . . . . . . . . . . . . . . . . . . . . . . . . . . . . . . . . . . . . . . KY927818.1_Cambodia_Pig**

**8101 . . . . . . . . . . . . . . . . . . . . . . . . . . . . . . . . . . . . . . . . . . . . . . . . . . . . . . . . . . . . . . . . . . . . . . . . . . . . . . . . . . . . . . . . . . . . . . . . . . . . LC461960.1_Japan_Pig**

**8101 . . . . . . . . . . . . . . . . . . . . . . . . . . . . . . . . . . . . . . . . . . . . . . . . . . . . . . . . . . . . . . . . . . . . . . . . . . . . . . . . . . . . . . . . . . . . . . . . . . . . LC708276._Japan_Pig**

**8101 . . . . . . . . . . . . . . . . . . . . . . . . . . . . . . . . . . . . . . . . . . . . . . . . . . . . . . . . . . . . . . . . . . . . . . . . . . . . . . . . . . . . . . . . . . . . . . . . . . . . MN544780.1_China_Pig**

**8101 . . . . . . . . . . . . . . . . . . . . . . . . . . . . . . . . . . . . . . . . . . . . . . . . . . . . . . . . . . . . . . . . . . . . . . . . . . . . . . . . . . . . . . . . . . . . . . . . . . . . MT232844.1_India_Pig**

**8101 . . . . . . . . . . . . . . . . . . . . . . . . . . . . . . . . . . . . . . . . . . . . . . . . . . . . . . . . . . . . . . . . . . . . . . . . . . . . . . . R . . . . . . . . . . . . . . . . . . . . MT253737.1_Australia_Pig**

**8101 . . . . . . . . . . . . . . . . . . . . . . . . . . . . . . . . . . . . . . . . . . . . . . . . . . . . . . . . . . . . . . . . . . . . . . . . . . . . . . . . . . . . . . . . . . . . . . . . . . . . MZ702743.1_India_Assam_Pig**

**8101 . . . . . . . . . . . . . . . . . . . . . . . . . . . . . . . . . . . . . . . . . . . . . . . . . . . . . . . . . . . . . . . . . . . . . . . . . . . . . . . . . . . . . . . . . . . . . . . . . . . . U15763.1_USA_Pig**

**S N Q E K I K K R I Q K L K E E F A T T W H K D P E H P Y R T W T Y H G S Y E V K A T G S A S S L V N G V V K L M S K P W D A I A N V T T M A M T D T T P F G Q Q R V F K E K V D T K A P E P P A G A K Majority**

**------------------+-------------------+-------------------+-------------------+-------------------+-------------------+-------------------+-------------------+-------------------+-------------------+-**

**2810 2820 2830 2840 2850 2860 2870 2880 2890 2900**

**------------------+-------------------+-------------------+-------------------+-------------------+-------------------+-------------------+-------------------+-------------------+-------------------+-**

**8401 . . . . . . . . . . . . . . . . . . . . . . . . . . . . . . . . . . . . . . . . . . . . . . . . . . . . . . . . . . . . . . . . . . . . . . . . . . . . . . . . . . . . . . . . . . . . . . . . . . . . AB551990.1_Japan_Pig**

**8401 . T . . . . R . . . . . . R . G . . . . . . . . . . . . . . . . . . . . . . . . . . . . . . . . . . . . . . . . . . . . . . . . . . . . . . . . . . . . . . . . . . . . . . . . . . . . . . . . . . V . AY316157.1_Korea_Pig**

**8401 . . . . . . . . . . . . . . . . . . . . . . . . . . . . . . . . . . . . . . . . . . . . . . . . . . . . . . . . . . . . . . . . . . . . . . . . . . . . . . . . . . . . . . . . . . . . . . . . . . . . AY849939.1_China_Pig**

**8401 . . . . . . . . . . . . . . . . . . . . . . . . . . . . . . . . . . . . . . . . . . . . . . . . . . . . . . . . . . . . . . . . . . . . . . . . . . . . . . . . . . . . . . . . . . . . . . . . . . . . EF107523.1_China_Pig**

**8401 . . . . . . R . . . . . . R . . . . . . . . . . . . . . . . . . . . . . . . . . . . . . . . . . . . . . . . . . . . . . . . . . . . . . . . . . . . . . . . . . . . . . . . . . . . . . . . . . . . V . GQ902058.1_Thailand_Pig**

**8401 . . . . . . R . . . . . . R . . . . . . . . . . . . . . . . . . . . . . . . . . . . . . . . . . . . . . . . . . . . . . . . . . . . . . . . . . . . . . . . . . . . . . . . . . . . . . . . . . . . V . GQ902061.1_Thailand_Pig**

**8401 . . . . . . . . . . . . . . . . . . . . . . . . . . . . . . . . . . . . . . . . . . . . . . . . . . . . . . . . . . . . . . . . . . . . . . . . . . . . . . . . . . . . . . . . . . . . . . . . . . . . JN381872.1_China_Pig**

**8401 . . . . . . . . . . . . . . . . . . . . . . . . . . . . . . . . . . . . . . K . . . . . . . . . . . . . . . . . . . . . . . . . . . . . . . . . . . . . . . . . . . . . . . . . . . . . . . . . . . . . KC915016.1_China_Pig**

**8401 . . . . . . . . . . . . . . . . . . . . . . . . . . . . . . . . . . . . . . . . . . . . . . . . . . . . . . . . . . . . . . . . . . . . . . . . . . . . . . . . . . . . . . . . . . . . . . . . . . . . KF297916.1_China_Pig**

**8401 . . . . . . . . . . . . . . . . . . . . . . . . . . . . . . . . . . . . . . . . . . . . . . . . . . . . . . . . . . . . . . . . . . . . . . . . . . . . . . . . . . . . . . . . . . . . . . . . . . . . KF711994.1_South Korea_Pig**

**8401 . . . . . . . . . . . . . . . . . . . . . . . . . . . . . . . . . . . . . . . . . . . . . . . . . . . . . . . . . . . . . . . . . . . . . . . . . . . . . . . . . . . . . . . . . . . . . . . . . . . . KP164498.2_India_Pig**

**8401 . . . . . . . . . . . . . . . . . . . . . . . . . . . . . . . . . . . . . . . . . . . . . . . . . . . . . . . . . . . . . . . . . . . . . . . . . . . . . . . . . . . . . . . . . . . . . . . . . . . . KT447437.1_South Korea_Pig**

**8401 . . . . . . . . . . . . . . . . . . . . . . . . . . . . . . . . . . . . . . . . . . . . . . . . . . . . . . . . . . . . . . . . . . . . . . . . . . . . . . . . . . . . . . . . . . . . . . . . . . . . KU363309.1_China_Pig**

**8401 . . . . . . . . . . . . . . . . . . . . . . . . . . . . . . . . . . . . . . . . . . . . . . . . . . . . . . . . I . . . . . . . . . . . . . . . . . . . . . . . . . . . . . . . . . . . . . . . . . . . KX965684.1_China_Pig**

**8401 . . . . . . R . . . . . . R . . . . . . . . . . . . . . . . . . . . . . . . . . . . . . . . . . . . . . . . . . . . . . . . . . . . . . . . . . . . . . . . . . . . . . . . . . . . . . . . . . . . V . KY927818.1_Cambodia_Pig**

**8401 . . . . . . . . . . . . . . . . . . . . . . . . . . . . . . . . . . . . . . . . . . . . . . . . . . . . . . . . . . . . . . . . . . . . . . . . . . . . . . . . . . . . . . . . . . . . . . . . . . . . LC461960.1_Japan_Pig**

**8401 . . . . . . R . . . . . . . . . . . . . . . . . . . . . . . . . . . . . . . . . . . . . . . . . . . . . . . . . . . . . . . . . . . . . . . . . . . . . . . . . . . . . . . . . . . . . . . . . . . V . LC708276._Japan_Pig**

**8401 . D . K . . R . . . . . . R . . . . . . . . . . . . . . . . . . . . . . . . . . . . . . . . . . . . . . . . . . . . . . . . . . . . . . . . . . . . . . . . . . . . . . . . . . . . . . . . . . . . V . MN544780.1_China_Pig**

**8401 . . . . . . R . . . . . . R . . . . . . . . . . . . . . . . . . . . . . . . . . . . . . . . . . . . . . . . . . . . . . . . . . . . . . . . . . . . . . . . . . . . . . . . . . . . . . . . . . . . V . MT232844.1_India_Pig**

**8401 . D . G . . . . . . . . . . . . . . . . . . . . . . . . . . . . . . . . . . . . . . . . . . . . . . . . . . . . . . . . . . . . . . . . . . . . . . . . . . . . . . . . . . . . . . . . . . . . . . V . MT253737.1_Australia_Pig**

**8401 . . . . . . R . . . . . . R . . . . . . . . . . . . . . . . . . . . . . . . . . . . . . . . . . . . . . . . . . . . . . . . . . . . . . . . . . . . . . . . . . . . . . . . . . . . . . . . . . . . V . MZ702743.1_India_Assam_Pig**

**8401 . . . . . . . . . . . . . . . . . . . . . . . . . . . . . . . . . . . . . . . . . . . . . . . . . . . . . . . . . . . . . . . . . . . . . . . . . . . . . . . . . . . . . . . . . . . . . . . . . . . . U15763.1_USA_Pig**

**E V L N E T T N W L W A H L S R E K R P R L C T K E E F I K K V N S N A A L G A V F A E Q N Q W S T A R E A V D D P R F W E M V D E E R E N H L R G E C H T C I Y N M M G K R E K K P G E F G K A K G S Majority**

**------------------+-------------------+-------------------+-------------------+-------------------+-------------------+-------------------+-------------------+-------------------+-------------------+-**

**2910 2920 2930 2940 2950 2960 2970 2980 2990 3000**

**------------------+-------------------+-------------------+-------------------+-------------------+-------------------+-------------------+-------------------+-------------------+-------------------+-**

**8701 . . . . . . . . . . . . . . . . . . . . . . . . . . . . . . . . . . . . . . . . . . . . . . . . . . . . . . . . . . . . . . . . . . . . . . . . . . . . . . . . . . . . . . . . . . . . . . . . . . . . AB551990.1_Japan_Pig**

**8701 . . . . . . . . . . . . . . . . . . . . . . . . . . . . . . . . . . . . S . . . . . . . . . . . . . . . . . . G . . L . . . . . N . . . . . . . . . . . . . . . . . . . . . . . . . . . . . . . . . . . AY316157.1_Korea_Pig**

**8701 . . . . . . . . . . . . Y . . . . . . . . . . . . . . . . . . . . . . . . . . . . . . . . . . . . . . . . . . . . . . . . . . . . . . . . . . . . . . . . . . . . . . . . . . . . . . . . . . . . . . . AY849939.1_China_Pig**

**8701 . . . . . . . . . . . . Y . . . . . . . . . . . . . . . . . . . . . . . . . . . . . . . . . . . . . . . . . . . . . . . . . . . . . . . . . . . . . . . . . . . . . . . . . . . . . . . . . . . . . . . EF107523.1_China_Pig**

**8701 . . . . . . . . . . . . . . . . . . . . . . . . . . . . . . . . . . . . . . . . . . . . . . . . . . . . . . . G . . L . . . . . . . . . . . . . . . . . . . . . . . . . . . . . . . . . . . . . . . . . GQ902058.1_Thailand_Pig**

**8701 . . . . . . . . . . . . . . . . . . . . . . . . . . . . . . . . . . . . . . . . . . . . . . . . . . . . . . . G . . L . . . . . . . . . . . . . . . . . . . . . . . . . . . . . . . . . . . . . . . . . GQ902061.1_Thailand_Pig**

**8701 . . . . . . . . . . . . . . . . . . . . . . . . . . . . . . . . . . . . . . . . . . . . . . . . . . . . . . . . . . . . . . . . . . . . . . . . . . . . . . . . . . . . . . . . . . . . . . . . . . . . JN381872.1_China_Pig**

**8701 . . . . . . . . . . . . . . . . . . . . . . . . . . . . . . . . . . . . . . . . . . . . . . . . . . . . . . . . . . . . . . . . . . . . . . . . . . . . . . . . . . . . . . . . . . . . . . . . . . . . KC915016.1_China_Pig**

**8701 . . . . . . . . . . . . . . . . . . . . . . . . . . . . . . . . . . . . . . . . . . . . . . . . . . . . . . . . . . . . . . . . . . . . . . . . . . . . . . . . . . . . . . . . . . . . . . . . . . . . KF297916.1_China_Pig**

**8701 . . . . . . . . . . . . . . . . . . . . . . . . . . . . . . . . . . . . . . . . . . . . . . . . . . . . . . . . . . . . . . . . . . . . . . . . . . . . . . . . . . . . . . . . . . . . . . . . . . . . KF711994.1_South Korea_Pig**

**8701 . . . . . . . . . . . . . . . . . . . . . . . . . . . . . . . . . . . . . . . . . . . . . . . . . . . . . . . . . . . . . . . . . . . . . . . . . . . . . . . . . . . . . . . . . . . . . . . . . . . . KP164498.2_India_Pig**

**8701 . . . . . . . . . . . . Y . . . . . . . . . . . . . . . . . . . . . . . . . . . . . . . . . . . . . . . . . . . . . . . . . . . . . . . . . . . . . . . . . . . . . . . . . . . . . . . . . . . . . . . KT447437.1_South Korea_Pig**

**8701 . . . . . . . . . . . . Y . . . . . . . . . . . . . . . . . . . . . . . . . . . . . . . . . . . . . . . . . . . . . . . . . . . . . . . . . . . . . . . . . . . . . . . . . . . . . . . . . . . . . . . KU363309.1_China_Pig**

**8701 . . . . . . . . . . . . Y . . . . . . . . . . . . . . . . . . . . . . . . . . . . . . . . . . . . . . . . . . . . . . . . . . . . . . . . . . . . . . . . . . . . . . . . . . . . . . . . . . . . . . . KX965684.1_China_Pig**

**8701 . . . . . . . . . . . . . . . . . . . . . . . . . . . . . . . . . . . . . . . . . . . . . . . . . . . . . . . S . . L . . . . . N . . . . . . . . . . . . . . . . . . . . . . . . . . . . . . . . . . . KY927818.1_Cambodia_Pig**

**8701 . . . . . . . . . . . . . . . . . . . . . . . . . . . . . . . . . . . . . . . . . . . . . . . . . . . . . . . . . . . . . . . . . . . . . . . . . . . . . S . V . . . . . . . . . . . . . . . . . . . . LC461960.1_Japan_Pig**

**8701 . . . . . . . . . . . . . . . . . . . . . . . . . . . . . . . . . . . . . . . . . . . . . . . . . . . . . . . G . . L . . . . . N . . . . . . . . . . . . . . . . . . . . . . . . . . . . . . . . . . . LC708276._Japan_Pig**

**8701 . . . . . . . . . . . . . . . . . . . . . . . . . . . . . . . . . . . . . . . . . . . . . . . . . . . . . . . G . . L . . . . . N . . . . . . . . . . . . . . V . . . . . . . . . . . . . . . . . . . . MN544780.1_China_Pig**

**8701 . . . . . . . . . . . . . . . . . . . . . . . . . . . . . . . . . . . . . . . . . . . . . . . . . . . . . . . G . . L . . . . . N . . . . . . . . . . . . . . . . . . . . . . . . . . . . . . . . . . . MT232844.1_India_Pig**

**8701 . . . . . . . . . . . . . . . . . . . . . . . . . . . . . . . . . . . . . . . . . . . . . . . . . . . . . . . . . . . . . . . . . . . . . . . . . . . . . . . V . . . . . . . . . . . . . . . . . . . . MT253737.1_Australia_Pig**

**8701 . . . . . . . . . . . . . . . . . . . . . . . . . . . . . . . . . . . . . . . . . . . . . . . . . . . . . . . G . . L . . . . . N . . . . . . . . . . . . . . . . . . . . . . . . . . . . . . . . . . . MZ702743.1_India_Assam_Pig**

**8701 . . . . . . . . . . . . . . . . . . . . . . . . . . . . . . . . . . . . . . . . . . . . . . . . . . . . . . . . . . . . . . . . . . . . . . . . . . . . . . . . . . . . . . . . . . . . . . . . . . . . U15763.1_USA_Pig**

**R A I W F M W L G A R Y L E F E A L G F L N E D H W L S R E N S G G G V E G S G V Q K L G Y I L R D I A G K Q G G K M Y A D D T A G W D T R I T R T D L E N E A K V L E L L D G E H R M L A R A I I E L Majority**

**------------------+-------------------+-------------------+-------------------+-------------------+-------------------+-------------------+-------------------+-------------------+-------------------+-**

**3010 3020 3030 3040 3050 3060 3070 3080 3090 3100**

**------------------+-------------------+-------------------+-------------------+-------------------+-------------------+-------------------+-------------------+-------------------+-------------------+-**

**9001 . . . . . . . . . . . . . . . . . . . . . . . . . . . . . . . . . . . . . . . . . . . . . . . . . . . . . . . . . . . . . . . . . . . . . . . . . . . . . . . . . . . . . . . . . . . . . . . . . . . . AB551990.1_Japan_Pig**

**9001 . . . . . . . . . . . . . . . . . . . . . . . . . . . . . . . . . . . . . . . S . . . . . . . . . . . . . . . . . . . . . . . P . . . . . . . . . . . . . . . . . . . . . . . . . . . . . . . . . . . . AY316157.1_Korea_Pig**

**9001 . . . . . . . . . . . . . . . . . . . . . . . . . . . . . . . . . . . . . . . . . . . . . . . . . . . . . . . . . . . . . . . . . . . . . . . . . . . . . . . . . . . . . . . . . . . . . . . . . . . . AY849939.1_China_Pig**

**9001 . . . . . . . . . . . . . . . . . . . . . . . . . . . . . . . . . . . . . . . . . . . . . . . . . . . . . . . . . . . . . . . . . . . . . . . . . . . . . . . . . . . . . . . . . . . . . . . . . . . . EF107523.1_China_Pig**

**9001 . . . . . . . . . . . . . . . . . . . . . . . . . . . . . . . . . . . . . . . . . . . . . . . . . . . . . . . . . . . . . . . . . . . . . . . . . . . . . . . . . . . . . . . . . . . . . . . . . . . . GQ902058.1_Thailand_Pig**

**9001 . . . . . . . . . . . . . . . . . . . . . . . . . . . . . . . . . . . . . . . . . . . . . . . . . . . . . . . . . . . . . . . . . . . . . . . . . . . . . . . . . . . . . . . . . . . . . . . . . . . . GQ902061.1_Thailand_Pig**

**9001 . . . . . . . . . . . . . . . . . . . . . . . . . . . . . . . . . . . . . . . . . . . . . . . . . . . . . . . . . . . . . . . . . . . . . . . . . . . . . . . . . . . . . . . . . . . . . . . . . . . . JN381872.1_China_Pig**

**9001 . . . . . . . . . . . . . . . . . . . . . . . . . . . . . . . . . . . . . . . . . . . . . . . . . . . . . . . . . . . . . . . . . . . . . . . . . . . . . . . . . . . . . . . . . . . . . . . . . . . . KC915016.1_China_Pig**

**9001 . . . . . . . . . . . . . . . . . . . . . . . . . . . . . . . . . . . . . . . . . . . . . . . . . . . . . . . . . . . . . . . . . . . . . . . . . . . . . . . . . . . . . . . . . . . . . . . . . . . . KF297916.1_China_Pig**

**9001 . . . . . . . . . . . . . . . . . . . . . . . . . . . . . . . . . . . . . . . . . . . . . . . . . . . . . . . . . . . . . . . . . . . . . . . . . . . . . . . . . . . . . . . . . . . . . . . . . . . . KF711994.1_South Korea_Pig**

**9001 . . . . . . . . . . . . . . . . . . . . . . . . . . . . . . . . . . . . . . . . . . . . . . . . . . . . . . . . . . . . . . . . . . . . . . . . . . . . . . . . . . . . . . . . . . . . . . . . . . . . KP164498.2_India_Pig**

**9001 . . . . . . . . . . . . . . . . . . . . . . . . . . . . . . . . . . . . . . . . . . . . . . . . . . . . . . . . . . . . . . . . . . . . . . . . . . . . . . . . . . . . . . . . . . . K . . . . . . . . KT447437.1_South Korea_Pig**

**9001 . . . . . . . . . . . . . . . . . . . . . . . . . . . . . . . . . . . . . . . . . . . . . . . . . . . . . . . . . . . . . . . . . . . . . . . . . . . . . . . . . . . . . . . . . . . . . . . . . . . . KU363309.1_China_Pig**

**9001 . . . . . . . . . . . . . . . . . . . . . . . . . . . . . . . . . . . . . . . . . . . . . . . . . . . . . . . . . . . . . . . . . . . . . . . . . . . . . . . . . . . . . . . . . . . . . . . . . . . . KX965684.1_China_Pig**

**9001 . . . . . . . . . . . . . . . . . . . . . . . . . . . . . . . . . . . . . . . . . . . . . . . . . . . . . . . . . . . . . . . . . . . . . . . . . . . . . . . . . . . . . . . . . . . . . . . . . . . . KY927818.1_Cambodia_Pig**

**9001 . . . . . . . . . . . . . . . . . . . . . . . . . . . . . . . . . . . . . . . . . . . . . . . . . . . . . . . . . . . . . . . . . . . . . . . . . . . . . . . . . . . . . . . . . . . . . . . . . . . . LC461960.1_Japan_Pig**

**9001 . . . . . . . . . . . . . . . . . . . . . . . . . . . . . . . . . . . . . . . . . . . . . . . . . . . . . . . . . . . . . . . . . . . . . . . . . . . . . . . . . . . . . . . . . . . . . . . . . . . . LC708276._Japan_Pig**

**9001 . . . . . . . . . . . . . . . . . . . . . . . . . . . . . . . . . . . . . . . . . . . . . . . . . . . . . . . . . . . . . . . . . . . . . . . . . . . . . . . . . . . . . . . . . . . . . . . . . . . . MN544780.1_China_Pig**

**9001 . . . . . . . . . . . . . . . . . . . . . . . . . . . . . . . . . . . . . . . . . . . . . . . . . . . . . . . . . . . . . . . . . . . . . . . . . . . . . . . . . . . . . . . . . . . . . . . . . . . . MT232844.1_India_Pig**

**9001 . . . . . . . . . . . . . . . . . . . . . . . . . . . . . . . . . . . . . . . . . . . . . . . . . . . . . . . . . . . . . . . . . . . . . . . . . . . . . . . . . . . . . . . . . . . . . . . . . . . . MT253737.1_Australia_Pig**

**9001 . . . . . . . . . . . . . . . . . . . . . . . . . . . . . . . . . . . . . . . . . . . . . . . . . . . . . . . . . . . . . . . . . . . . . . . . . . . . . . . . . . . . . . . . . D . . . . . . . . . . MZ702743.1_India_Assam_Pig**

**9001 . . . . . . . . . . . . . . . . . . . . . . . . . . . . . . . . . . . . . . . . . . . . . . . . . . . . . . . . . . . . . . . . . . . . . . . . . . . . . . . . . . . . . . . . . . . . . . . . . . . . U15763.1_USA_Pig**

**T Y R H K V V K V M R P A A E G K T V M D V I S R E D Q R G S G Q V V T Y A L N T F T N I A V Q L V R L M E A E G V I G P Q H L E Q L P R K N K I A V R T W L F E N G E E R V T R M A I S G D D C V V K Majority**

**------------------+-------------------+-------------------+-------------------+-------------------+-------------------+-------------------+-------------------+-------------------+-------------------+-**

**3110 3120 3130 3140 3150 3160 3170 3180 3190 3200**

**------------------+-------------------+-------------------+-------------------+-------------------+-------------------+-------------------+-------------------+-------------------+-------------------+-**

**9301 . . . . . . . . . . . . . . . . . . . . . . . . . . . . . . . . . . . . . . . . . . . . . . . . . . . . . . . . . . . . . . . . . . . . . . T . . . . . . . . . . . . . . . . . . . . . . . . . . . . . AB551990.1_Japan_Pig**

**9301 . . . . Q . F . . . . . . . G . . . . . . . . . . . . . . . . . . . . . . . . . . . . . . . . . F . . . . K V . . . . . . . . . . . . . . . . . . . . . . . . . . . . . . . . . . . . . . . . . . . . . AY316157.1_Korea_Pig**

**9301 . . . . . . . . . . . . . . . . . . . . . . . . . . . . . . . . . . . . . . . . . . . . . . . . . . . . . . . . . . . . . . . . . . . . . . . . . . . . . . . . . . . . . . . . . . . . . . . . . . . . AY849939.1_China_Pig**

**9301 . . . . . . . . . . . . . . . . . . . . . . . . . . . . . . . . . . . . . . . . . . . . . . . . . . . . . . . . . . . . . . . . . . . . . . . . . . . . . . . . . . . . . . . . . . . . . . . . . . . . EF107523.1_China_Pig**

**9301 . . . . . . . . . . . . . . G . . . . . . . . . . . . . . . . . . . . . . . . . . . . . . . . . . . . . . . . . . . . . . . . . . . . . . . . . . . . . . . . . . . . . . . . . . . . . . . . . . . . . GQ902058.1_Thailand_Pig**

**9301 . . . . . . . . . . . . . . G . . . . . . . . . . . . . . . . . . . . . . . . . . . . . . . . . . . . . . . . . . . . . . . . . . . . . . . . . . . . . . . . . . . . . . . . . . . . . . . . . . . . . GQ902061.1_Thailand_Pig**

**9301 . . . . . . . . . . . . . . . . . . . . . . . . . . . . . . . . . . . . . . . . . . . . . . . . . . . . . . . . . . . . . . . . . . . . . . T . . . . . . . . . . . . . . . . . . . . . . . . . . . . . JN381872.1_China_Pig**

**9301 . . . . . . . . . . . . . . . . . . . . . . . . . . . . . . . . . . . . . . . . . . . . . . . . . . . . . . . . . . . . . . . . . . . . . . T . . . . . . . . . . . . . . . . . . . . . . . . . . . . . KC915016.1_China_Pig**

**9301 . . . . . . . . . . . . . . . . . . . . . . . . . . . . . . . . . . . . . . . . . . . . . . . . . . . . . . . . . . . . . . . . . . . . . . T . . . . . . . . . . . . . . . . . . . . . . . . . . . . . KF297916.1_China_Pig**

**9301 . . . . . . . . . . . . . . . . . . . . . . . . . . . . . . . . . . . . . . . . . . . . . . . . . . . . . . . . . . . . . . . . . . . . . . T . . . . . . . . . . . . . . . . . . . . . . . . . . . . . KF711994.1_South Korea_Pig**

**9301 . . . . . . . . . . . . . . . . . . . . . . . . . . . . . . . . . . . . . . . . . . . . . . . . . . . . . . . . . . . . . . . . . . . . . . T . . . . . . . . . . . . . . . . . . . . . . . . . . . . . KP164498.2_India_Pig**

**9301 . . . . . . . . . . . . . . . . . . . . . . . . . . . . . . . . . . . . . . . . . . . . . . . . . . . . . . . . . . . . . . . . . . . . . . . . . . . . . . . . . . . . . . . . . . . . . . . . . . . . KT447437.1_South Korea_Pig**

**9301 . . . . . . . . . . . . . . . . . . . . . . . . . . . . . . . . . . . . . . . . . . . . . . . . . . . . . . . . . . . . . . . . . . . . . . . . . . . . . . . . . . . . . . . . . . . . . . . . . . . . KU363309.1_China_Pig**

**9301 . . . . . . . . . . . . . . . . . . . . . . . . . . . . . . . . . . . . . . . . . . . . . . . . . . . . . . . . . . . . . . . . . H . . . . . . . . . . . . . . . . . . . . . . . . . . . . . . . A . . KX965684.1_China_Pig**

**9301 . . . . . . . . . . . . . . G . . . . . . . . . . . . . . . . . . . . . . . . . . . . . . . . . . . . . . . . . . . . . . . . . . . . . . . S . . . . . . . . . . . . . . . . . . . . . . . . . . . . . KY927818.1_Cambodia_Pig**

**9301 . . . . . . . . . . . . . . . . . . . . . . . . . . . . . . . . . . . . . . . . . . . . . . . . . . . . . . . . . . . . . . . . . . . . . . T . . . . . . . . . . . . . . . . . . . . . . . . . . . . . LC461960.1_Japan_Pig**

**9301 . . . . . . . . . . . . . . G . . . . . . . . . . . . . . . . . . . . . . . . . . . . . . . . . . . . . . . . . . . . . . . . . . . . . . . . . . . . . . . . . . . . . . . . . . . . . . . . . . . . . LC708276._Japan_Pig**

**9301 . . . . . . . . . . . . . . G . . . . . . . . . . . . . . . . . . . . . . . . . . . . . . . . . . . . . . . . . . . . . . . . . . . . . . . . . . . . . . . . . . . . . . . . . . . . . . . . . . . . . MN544780.1_China_Pig**

**9301 . . . . . . . . . . . . . . G . . . . . . . . . . . . . . . . . . . . . . . . . . . . . . . . . . . . . . . . . . . . . . . . . . . . . . . T . . . . . . . . . . . . . . . . . . . . . . . . . . . . . MT232844.1_India_Pig**

**9301 . . . . . . . . . . . . . S G . . . . . . . . . . . . . . . . . . . . . . . . . . . . . . . . . . . . . . . . . . . . . . . . . . . . . . . T R . . . . . . . . . . . . . . . A . . . . . . . . . . . . MT253737.1_Australia_Pig**

**9301 . . . . . . . . . . . . . . G . . . . . . . . . . . . . . . . . . . . . . . . . . . . . . . . . . . . . . . . . . . . . . . . . . . . . . . D . . . . . . . . . . . . . . . . . . . . . . . . . . . . . MZ702743.1_India_Assam_Pig**

**9301 . . . . . . . . . . . . . . . . . . . . . . . . . . . . . . . . . . . . . . . . . . . . . . . . . . . . . . . . . . . . . . . . . . . . . E . . . . . . . . . . . . . . . . . . . . . . . . . . . . . . U15763.1_USA_Pig**

**P L D D R F A T A L H F L N A M S K V R K D I Q E W K P S H G W H D W Q Q V P F C S N H F Q E I V M K D G R S I V V P C R G Q D E L I G R A R I S P G A G W N V K D T A C L A K A Y A Q M W L L L Y F H Majority**

**------------------+-------------------+-------------------+-------------------+-------------------+-------------------+-------------------+-------------------+-------------------+-------------------+-**

**3210 3220 3230 3240 3250 3260 3270 3280 3290 3300**

**------------------+-------------------+-------------------+-------------------+-------------------+-------------------+-------------------+-------------------+-------------------+-------------------+-**

**9601 . . . . . . . . . . . . . . . . . . . . . . . . . . . . . . . . . . . . . . . . . . . . . . . . . . . . . . . . . . . . . . . . . . . . . . . . . . . . . . . . . . . . . . . . . . . . . . . . . . . . AB551990.1_Japan_Pig**

**9601 . . . . . . . . . . . . . . . . . . . . . . . . . . . . . . . . . . . . . . . . . . . . . . . . . . . . . . . . . . . . . . . . . . . . . . . . . . . . . . . . . . . . . . . . . . . . . . . . . . . . AY316157.1_Korea_Pig**

**9601 . . . . . . . . . . . . . . . . . . . . . . . L . . . . . . . . . . . . . . . . . . . . . . . . . . . . . . . . . . . . . . . . . . . . . . . . . . . . . . . . . . . . . . . . . . . . . R . . . . . . AY849939.1_China_Pig**

**9601 . . . . . . . . . . . . . . . . . . . . . . . . . . . . . . . . . . . . . . . . . . . . . . . . . . . . . . . . . . . . . . . . . . . . . . . . . . . . . . . . . . . . . . . . . . . . . . . . . . . . EF107523.1_China_Pig**

**9601 . . . . . . . . . . . . . . . . . . . . . . . . . . . . . . . . . . . . . . . . . . . . . . . . . L . . . . . . . . . . . . . . . . . . . . . . . . . . . . . . . . . . . . . . . . . . . . . . . . . . GQ902058.1_Thailand_Pig**

**9601 . . . . . . . . . . . . . . . . . . . . . . . . . . . . . . . . . . . . . . . . . . . . . . . . . . . . . . . . . . . . . . . . . . . . . . . . . . . . . . . . . . . . . . . . . . . . . . . . . . . . GQ902061.1_Thailand_Pig**

**9601 . . . . . . . . . . . . . . . . . . . . . . . . . . . . . . . . . . . . . . . . . . . . . . . . . . . . . . . . . . . . . . . . . . . . . . . . . . . . . . . . . . . . . . . . . . . . . . . . . . . . JN381872.1_China_Pig**

**9601 . . . . . . . . . . . . . . . . . . . . . . . . . . . . . . . . . . . . . . . . . . . . . . . . . . . . . . . . . . . . . . . . . . . . . . . . . . . . . . . . . . . . . . . . . . . . . . . . . . . . KC915016.1_China_Pig**

**9601 . . . . . . . . . . . . . . . . . . . . . . . . . . . . . . . . . . . . . . . . . . . . . . . . . . . . . . . . . . . . . . . . . . . . . . . . . . . . . . . . . . . . . . . . . . . . . . . . . . . . KF297916.1_China_Pig**

**9601 . . . . . . . . . . . . . . . . . . . . . . . . . . . . . . . . . . . . . . . . . . . . . . . . . . . . . . . . . . . . . . . . . . . . . . . . . . . . . . . . . . . . . . . . . . . . . . . . . . . . KF711994.1_South Korea_Pig**

**9601 . . . . . . . . . . . . . . . . . . . . . . . . . . . . . . . . . . . . . . . . . . . . . . . . . . . . . . . . . . . . . . . . . . . . . . . . . . . . . . . . . . . . . . . . . . . . . . . V . . . . KP164498.2_India_Pig**

**9601 . . . . . . . . . . . . . . . . . . . . . . . . . . . . . . . . . . . . . . . . . . . . . . . . . . . . . . . . . . . . . . . . . . . . . . . . . . . . . . . . . . . . . . . . . . . . . . . . . . . . KT447437.1_South Korea_Pig**

**9601 . . . . . . . . . . . . . . . . . . . . . . . . . . . . . . . . . . . . . . . . . . . . . . . . . . . . . . . . . . . . . . . . . . . . . . . . . . . . . . . . . . . . . . . . . . . . . . . . . . . . KU363309.1_China_Pig**

**9601 . . . . . . . . . . . . . . . . . . . . . . . . . . . . . . . . . . . . . . . . . . . . . . . . . . . . . . . . . . . . . . . . . . . . . . . . . . . . . . . . . . . . . . . . . . . . . . . . . . . . KX965684.1_China_Pig**

**9601 . . . . . . . . . . . . . . . . . . . . . . . . . . . . . . . . . . . . . . . . . . . . . . . . . . . . . . . . . . . . . . . . . . . . . . . . . . . . . . . . . . . . . . . . . . . . . . . . . . . . KY927818.1_Cambodia_Pig**

**9601 . . . . . . . . . . . . . . . . . . . . . . . . . . . . . . . . . . . . . . . . . . . . . . . . . . . . . . . . . . . . . . . . . . . . . . . . . . . . . . . . . . . . . . . . . . . . . . . . . . . . LC461960.1_Japan_Pig**

**9601 . . . . . . . . . . . . . . . . . . . . . . . . . . . . . . . . . . . . . . . . . . . . . . . . . . . . . . . . . . . . . . . . . . . . . . . . . . . . . . . . . . . . . . . . . . . . . . . . . . . . LC708276._Japan_Pig**

**9601 . . . . . . . . . . . . . . . . . . . . . . . . . . . . . . . . . . . . . . . . . . . . . . . . . . . . . . . . . . . . . . . . . . . . . . . . . . . . . . . . . . . . . . . . . . . . . . . . . . . . MN544780.1_China_Pig**

**9601 . . . . . . . . . . . . . . . . . . . . . . . . . . . . . . . . . . . . . . . . . . . . . . . . . . . . . . . . . . . . . . . . . . . . . . . . . . . . . . . . R . . . . . . . . . . . . . . . . . . . MT232844.1_India_Pig**

**9601 . . . . . . . . S . . . . . . . . . . . . . . . . . . . . . . . . . . . . . . . . . . . . . . . . . . . . . . . . . . . . . . . . . . . . . . . . . . . . . . . . . . . . . . . . . . . . . . . . . . . MT253737.1_Australia_Pig**

**9601 . . . . . . . . . . . . . . . . . . . . . . . . . . . . . . . . . . . . . . . . . . . . . . . . . . . . . . . . . . . . . . . . . . . . . . . . . . . . . . . . R . . . . . . . . . . . . . . . . . . . MZ702743.1_India_Assam_Pig**

**9601 . . . . . . . . . . . . . . . . . . . . . . . . . . . . . . . . . . . . . . . . . . . . . . . . . . . . . . . . . . . . . . . . . . . . . . . . . . . . . . . . . . . . . . . . . . . . . . . . . . . . U15763.1_USA_Pig**

**R R D L R L M A N A I C S A V P V D W V P T G R T S W S I H S K G E W M T T E D M L Q V W N R V W I E E N E W M M D K T P I T S W T D V P Y V G K R E D I W C G S L I G T R S R A T W A E N I Y A A I N Majority**

**------------------+-------------------+-------------------+-------------------+-------------------+-------------------+-------------------+-------------------+-------------------+-------------------+-**

**3310 3320 3330 3340 3350 3360 3370 3380 3390 3400**

**------------------+-------------------+-------------------+-------------------+-------------------+-------------------+-------------------+-------------------+-------------------+-------------------+-**

**9901 . . . . . . . . . . . . . . . . . . . . . . . . . . . . . . . . . . . . . . . . . . . . . . . . . . . . . . . . . . . . . . . . . . . . . . . . . . . . . . . . . . . . . . . . . . . . . . . . . . . . AB551990.1_Japan_Pig**

**9901 . . . . . . . . . . . . . . . . . . . . . . . . . . . . . . . . . . . . . . . . . . . . . . . . . . . . . . . . . . . . . . . . . . . . . . . . . . . . . . . . . . . . . . . . . . . . . . . . . . . . AY316157.1_Korea_Pig**

**9901 . . . . C . . . . . . . . . . . . . . . . . . . . . . . . . . . . . . . . . . . . . . . . . . . . . . . . . . . . . . . . . . . . . . . . . . . . . . . . . . . . . . . . . . . . . . . . . . . . . . . AY849939.1_China_Pig**

**9901 . . . . . . . . . . . . . . . . . . . . . . . . . . . . . . . . . . . . . . . . . . . . . . . . . . . . . . . . . . . . . . . . . . . . . . . . . . . . . . . . . . . . . . . . . . . . . . . . . . . . EF107523.1_China_Pig**

**9901 . . . . . . . . . . . . . . . . . . . . . . . . . . . . . . . . . . . . . . . . . . . . . . . . . . . . . . . . . . . . . . . . . . . . . . . . . . . . . . . . . . . . . . . . . . . . . . . . . . . . GQ902058.1_Thailand_Pig**

**9901 . . . . . . . . . . . . . . . . . . . . . . . . . . . . . . . . . . . . . . . . . . . . . . . . . . . . . . . . T . . . . . . . . . . . . . . . . . . . . . . . . . . . . . . . . . . . . . . . . . . . GQ902061.1_Thailand_Pig**

**9901 . . . . . . . . . . . . . . . . . . . . . . . . . . . . . . . . . . . . . . . . . . . . . . . . . . . . . . . . . . . . . . . . . . . . . . . . . . . . . . . . . . . . . . . . . . . . . . . . . . . . JN381872.1_China_Pig**

**9901 . . . . . . . . . . . . . . . . . . . . . . . . . . . . . . . . . . . . . . . . . . . . . . . . . . . . . . . . . . . . . . . . . . . . . . . . . . . . . . . . . . . . . . . . . . . . . . . . . . . . KC915016.1_China_Pig**

**9901 . . . . . . . . . . . . . . . . . . . . . . . . . . . . . . . . . . . . . . . . . . . . . . . . . . . . . . . . . . . . . . . . . . . . . . . . . . . . . . . . . . . . . . . . . . . . . . . . . . . . KF297916.1_China_Pig**

**9901 . . . . . . . . . . . . . . . . . . . . . . . . . . . . . . . . . . . . . . . . . . . . . . . . . . . . . . . . . . . . . . . . . . . . . . . . . . . . . . . . . . . . . . . . . . . . . . . . . . . . KF711994.1_South Korea_Pig**

**9901 . . . . . . . . . . . . . . . . . . . . . . . . . . . . . . . . . . . . . . . . . . . . . . . . . . . . . . . . . . . . . . . . . . . . . . . . . . . . . . . . . . . . . . . . . . . . . . . . . . . . KP164498.2_India_Pig**

**9901 . . . . . . . . . . . . . . . . . . . . . . . . . . . . . . . . . . . . . . . . . . . . . . . . . . . . . . . . . . . . . . . . . . . . . . . . . . . . . . . . . . . . . . . . . . . . . . . . . . . . KT447437.1_South Korea_Pig**

**9901 . . . . . . . . . . . . . . . . . . . . . . . . . . . . . . . . . . . . . . . . . . . . . . . . . . . . . . . . . . . . . . . . . . . . . . . . . . . . . . . . . . . . . . . . . . . . . . . . . . . . KU363309.1_China_Pig**

**9901 . . . . . . . . . . . . . . . . . . . . . . . . . . . . . . . . . . . . . . . . . . . . . . . . . . . . . . . . . . . . . . . . . . . . . . . . . . . . . . . . . . . . . . . . . . . . . . . . . . . . KX965684.1_China_Pig**

**9901 . . . . . . . . . . . . . . . . . . . . . . . . . . . . . . . . . . . . . . . . . . . . . . . . . . . . . . . . . . . . . . . . . . . . . . . . . . . . . . . . . . . . . . . . . . . . . . . H . . . . KY927818.1_Cambodia_Pig**

**9901 . . . . . . . . . . . . . . . . . . . . . . . . . . . . . . . . . . . . . . . . . . . . . . . . . . . . . . . . T . . . . . . . . . . . . . . . . . . . . . . . . . . . . . . . . . . . . . . . . . . . LC461960.1_Japan_Pig**

**9901 . . . . . . . . . . . . . . . . . . . . . . . . . . . . . . . . . . . . . . . . . . . . . . . . . . . . . . . . . . . . . . . . . . . . . . . . . . . . . . . . . . . . . . . . . . . . . . V . . . . . LC708276._Japan_Pig**

**9901 . . . . . . . . . . . . . . . . . . . . . . . . . . . . . . . . . . . . . . . . . . . . . . . . . . . . . . . . . . . . . . . . . . . . . . . . . . . . . . . . . . . . . . . . . . . . . . . . . . . . MN544780.1_China_Pig**

**9901 . . . . . . . . . . . . . . . . . . . . . . . . . . . . . . . . . . . . . . . . . . . . . . . . . . . . . . . . . . . . . . . . . . . . . . . . . . . . . . . . . . . . . . . . . . . . . . . . . . . . MT232844.1_India_Pig**

**9901 . . . . . . . . . . . . . . . . . N . . . . . . . . . . . . . . . . . . . . . . . . . . . . . . . . . . . . . . T . . . . . . . . . . . . . . . . . . . . . . . . . . . . . . . . . . . . . . . . . . . MT253737.1_Australia_Pig**

**9901 . . . . . . . . . . . . . . . . . . . . . . . . . . . . . . . . . . . . . . . . . . . . . . . . . . . . . . . . . . . . . . . . . . . . . . . . . . . . . . . . . . . . . . . . . . . . . . . . . . . . MZ702743.1_India_Assam_Pig**

**9901 . . . . . . . . . . . . . . . . . . . . . . . . . . . . . . . . . . . . . . . . . . . . . . . . . . . . . . . . . . . . . . . . . . . . . . . . . . . . . . . . . . . . . . . . . . . . . . . . . . . . U15763.1_USA_Pig**

**Q V R A V I G K E N Y V D Y M T S L R R Y E D V L I Q E D R V I - Majority**

**------------------+-------------------+-------------------+-------**

**3410 3420 3430**

**------------------+-------------------+-------------------+-------**

**10201 . . . . . . . . . . . . . . . . . . . . . . . . . . . . . . . . . AB551990.1_Japan_Pig**

**10201 . . . . I . . . . . . . . . . . . . . . . . . . . . . . . . . . . AY316157.1_Korea_Pig**

**10201 . . . . . . . . . . . . . . . . . . . . . . . . . . . . . . . . . AY849939.1_China_Pig**

**10201 . . . . . . . . . . . . . . . . . . . . . . . . . . . . . . . . . EF107523.1_China_Pig**

**10201 . . . . I . . . . . . . . . . . . . . . . . . . . . . . . . . . . GQ902058.1_Thailand_Pig**

**10201 . . . . . . . . . . . . . . . . . . . . . . . . . . . . . . . . . GQ902061.1_Thailand_Pig**

**10201 . . . . . . . . . . . . . . . . . . . . . . . . . . . . . . . . . JN381872.1_China_Pig**

**10201 . . . . . . . . . . . . . . . . . . . . . . . . . . . . . . . . . KC915016.1_China_Pig**

**10201 . . . . . . . . . . . . . . . . . . . . . . . . . . . . . . . . . KF297916.1_China_Pig**

**10201 . . . . . . . . . . . . . . . . . . . . . . . . F . . . . . . . . KF711994.1_South Korea_Pig**

**10201 . . . . . . . . . . . . . . . . . . . . . . . . . . . . . . . . . KP164498.2_India_Pig**

**10201 . . . . . . . . . . . . . . . . . . . . . . . . . . . . . . . . . KT447437.1_South Korea_Pig**

**10201 . . . . . . . . . . . . . . . . . . . . . . . . . . . . . . . . . KU363309.1_China_Pig**

**10201 . . . . . . . . . . . . . . . . . . . . . . . . . . . . . . . . . KX965684.1_China_Pig**

**10201 . . . . I . . . . . . . . . . . . . . . . . . . . . . . . . . . . KY927818.1_Cambodia_Pig**

**10201 . . . . . . . . . . . . . . . . . . . . . . . . . . . . . . . . . LC461960.1_Japan_Pig**

**10201 . . . . I . . . . . . . . . . . . . . . . . . . . . . . . . . . . LC708276._Japan_Pig**

**10201 . . . . I . . . . . . . . . . . . . . . . . . . . . . . . . . . . MN544780.1_China_Pig**

**10201 . . . . I . . . . . . . . . . . . . . . . . . . . . . . . . . . . MT232844.1_India_Pig**

**10201 . . . . I . . . . . . . . . . L . . . . . . . . . . . . . . . . . MT253737.1_Australia_Pig**

**10201 . . . . I . . . . . . . . . . . . . . . . . . . . . . . . . . . . MZ702743.1_India_Assam_Pig**

**10201 . . . . . . . . . . . . . . . . . . . . . . . . . . . . . . . . . U15763.1_USA_Pig**
