# Supplementary material for: Japanese Encephalitis Virus Genotype III Strains Detection and Genome Sequencing from Indian Pig and Mosquito Vector
Source: Vaccines (Basel). 2023 Jan 10;11(1):150. doi: 10.3390/vaccines11010150 (PMC9862938; doi:10.3390/vaccines11010150)
Supplement: Supplementary file 1 [file vaccines-11-00150-s001.zip › vaccines-2082975-supplementary/Supplementary file 3.docx]

**Supplementary file 3: Alignments of amino acid sequences of JEV polyprotein gene (Isolated from mosquito)**

**M T K K P G G P G K N R A I N M L K R G L P R V F P L V G V K R V V M S L L D G R G P V R F V L A L I T F F K F T A L A P T K A L L G R W R A V E K S V A M K H L T S F K R E L G T L I D A V N K R G K Majority**

**------------------+-------------------+-------------------+-------------------+-------------------+-------------------+-------------------+-------------------+-------------------+-------------------+-**

**10 20 30 40 50 60 70 80 90 100**

**------------------+-------------------+-------------------+-------------------+-------------------+-------------------+-------------------+-------------------+-------------------+-------------------+-**

**1 . . . . . . . . . . . . . . . . . . . . . . . . . . . . . . . . . . . . . . . . . . . . . . . . . . . . . . . . . . . . . . . . . . . . . K . . . . . . . . . . . . . . . . . . . . . . . . . . . . . R AF098735.1_Taiwan_Mosquito**

**1 . . . . . . . . . . . . . . . . . . . . . . . . . . . . . . . . . . . . . . . . . . . . . . . . . . . . . . . . . . . . . . . . . . . . . . . . . . . . . . . . . . . . . . . . . . . . . . . . . . . . GQ902060.1_Thailand_Mosquito**

**1 . . . . . . . . . . . . . . . . . . . . . . . . . . . . . . . . . . . . . . . . . . . . . . . . . . . . . . . . . . . . . . . . . . . . . K . . . . . . . . . . . . . . . . . . . . . . . . . . . . . . GQ902063.1_Thailand_Mosquito**

**1 . . . . . . . . . . . . . . . . . . . . . . . . . . . . . . . . . . . . . . . . . . . . . . . . . . . . . . . . . . . . . . . . . . . . . . . . . . . . . . . . . . . . . . . . . . . . . . . . . . . . HQ652538.1_China_Mosquito**

**1 . . . . . . . . . R . . . . . . . . . . . . . . . . . . . . . . . . . . . . . . . . . . . . . . . . . . . . . . . . . . . . . . . . . . . . . . . . . . . . . . . . . . . . . . . . . . . . . . . . . . JF499790.1_Taiwan_Mosquito**

**1 . . . . . . . . . . . . . . . . . . . . . . . . . . . . . . . . . . . . . . . . . . . . . . . . . . T . . . . . . . . . . . . . . S . . . K . . . . . . . . . . . . . . . . . . . . . . . . . . . . . R JN864064.1_China_Mosquito**

**1 . . . . . . . . . R . . . . . . . . . . . . . . . . . . . . . . . . . . . . . . . . . . . . . . . . . . . . . . . . . . . . . . . . . . . . . . . . . . . . . . . . . . . . . . . . . . . . . . . . . . JQ031753.1_Taiwan_Mosquito**

**1 . . . . . . . . . . . . . . . . . . . . . . . . . . . . . . . . . . . . . . . . . . . . . . . . . . T . . . . . . . . . . . . . . S . . . K . . . . . . . . . . . . . . . . . . . . . . . . . . . . . R JQ086762.1_China_Mosquito**

**1 . . . . . . . . . . . . . . . . . . . . . . . . . . . . . . . . . . . . . . . . . . . . . . . . . . . . . . . . . . . . . . . . . . . . . K . . . . . . . . . . . . . . . . . . . . . . . . . . . . . . KT229574.1_China_Mosquito**

**1 . . . . . . . . . . . . . . . . . . . . . . . . . . . . . . . . . . . . . . . . . . . . . . . . . . . . . . . . . . . . . . . . . . . . . K . . . . . . . . . . . . . . . . . . . . . . . . . . . . . . KT229575.1_China_Mosquito**

**1 . . . . . . . . . . . . . . . . . . . . . . . . . . . . . . . . . . . . . . . . . . . . . . . . . . . . . . . . . . . . . . . . . . . . . . . . . . . . . . . . . . . . . . . . . . . . . . . . . . . . LC461957.1_Japan_Mosquito**

**1 . . . . . . . . . . . . . . . . . . . . . . . . . . . . . . . . . . . . . . . . . . . . . . . . . . . . . . . . . . . . . . . . . . . . . . . . . . . . . . . . . . . . . . . . . . . . . . . . . . . . LC513838.1_Japan_Mosquito**

**1 . . . . . . . . . . . . . . . . . . . . . . . . . . . . . . . . . . . . . . . . . . . . . . . . . . V A . . . . . . . . . . . . . . . . . K . . . . G . . . . . . . . . . . . . . . . . . . . . . . . R LC579814.1_Indonesia_Mosquito**

**1 . . . . . . . . . . . . . . . . . . . . . . . . . . . . . . . . . . . . . . . . . . . . . . . . . . . . . . . . . . . . . . . . . . . . . . . . . . . . . . . . . . . . . . . . . . . . . . . . . . . . LC623822.1_Japan_Mosquito**

**1 . . . . . . . . . . . . . . . . . . . . . . . . . . . . . . . . . . . . . . . . . . . . . . . . . . . . . . . . . . . . . . . . . . . . . . . . . . . . . . . . . . . . . . . . . . . . . . . . . . . . MH385014.1_China_Mosquito**

**1 . . . . . . . . . . . . . . . . . . . . . . . . . . . . . . . . . . . . . . . . . . . . . . . . . . . . . . . . . . . . . . . . . . . . . . . . . . . . . . . . . . . . . . . . . . . . . . . . . . . . MK558811.1_China_Mosquito**

**1 . . . . . . . . . . . . . . . . . . . . . . . . . . . . . . . . . . . . . . . . . . . . . . . . . . . . . . . . . . . . . . . . . . . . . . . . . . . . . . . . . . . . . . . . . . . . . . . . . . . . MT254426.1_China_Mosquito**

**1 . . . . . . . . . . . . . . . . . . . . . . . . . . . . . . . . . . . . . . . . . . . . . . . . . . . . . . . . . . . . . . . . . . . . . . . . . . . . . . . . . . . . . . . . . . . . . . . . . . . . MT560941.1_China_Mosquito**

**1 . . . . . . . . . . . . . . . . . . . . . . . . . . . . . . . . . . . . . . . . . . . . . . . . . . . . . . . . . . . . . . . . . . . . . . . . . . . . . . . . . . . . . . . . . . . . . . . . . . . . ON875960_India_Assam_Mosquito**

**K Q N K R G G N E S S I M W L A S L A I V T A C A G A M K L S N F Q G K L L M T I N N T D I A D V I V I P T S K G E N R C W V R A I D V G Y M C E D T I T Y E C P K L A V G N D P E D V D C W C D N Q E Majority**

**------------------+-------------------+-------------------+-------------------+-------------------+-------------------+-------------------+-------------------+-------------------+-------------------+-**

**110 120 130 140 150 160 170 180 190 200**

**------------------+-------------------+-------------------+-------------------+-------------------+-------------------+-------------------+-------------------+-------------------+-------------------+-**

**301 . . . . . . . . . G . . . . . . . . . V . I . . . . . . . . . . . . . . . . . . . . . . . . . . . . . . . . . . . . . . . . . . . . . . . . . . . . . . . . . . . . . T M . . . . . . . . . . . . . . . AF098735.1_Taiwan_Mosquito**

**301 . . . . . . . . . . . . . . . . . . . . . . . . . . . . . . . . . . . . . . . . . . . . . . . . . . . . . . . . . . . . . . . . . . . . . . . . . . . . . . . . . . . . . . . . . . . . . . . . . . . . GQ902060.1_Thailand_Mosquito**

**301 . . . . . . . . . G . . . . . T . . . V . I . . . . . . . . . . . . . . . . . . . . . . . . . . . . . . . . . . . . . . . . . . . . . . . . . . . . . . . . . . . . . T A . . . . . . . . . . . . . . . GQ902063.1_Thailand_Mosquito**

**301 . . . . . . . . . . . . . . . . . . . . I . . . . . . . . . . . . . . . . . . . . . . . . . . . . . . . . . . . . . . . . . . . . . . . . . . . . . . . . . . . . . . . . . . . . . . . . . . . . . . . HQ652538.1_China_Mosquito**

**301 . . . . . . . . . . . . . . . . . . . . . . . . . . . . . . . . . . . . . . . . . . . . . . . . . . . . . . . . . . . . . . . . . . . . . . . . . . . . . . . . . . . . M . . . . . . . . . . . . . . . JF499790.1_Taiwan_Mosquito**

**301 . . . . . . . . . G . . . . . . . . . V . I . . . . . . . . . . . . . . . . . . . . . . . . . . . . . . . . . . . . . . . . . . . . . . . . . . . . . . . . . . . . . T M . . . . . . . . . . . . . . . JN864064.1_China_Mosquito**

**301 . . . . . . . . . . . . . . . . . . . . . . . . . . . . . . . . . . . . . . . . . . . . . . . . . . . . . . . . . . . . . . . . . . . . . . . . . . . . . . . . . . . . . . . . . . . . . . . . . . . . JQ031753.1_Taiwan_Mosquito**

**301 . . . . . . . . . G . . . . . . . . . V . I . . . . . . . . . . . . . . . . . . . . . . . . . . . . . . . . . . . . . . . . . . . . . . . . . . . . . . . . . . . . . T M . . . . . . . . . . . . . . . JQ086762.1_China_Mosquito**

**301 . . . . . . . . . . . . . . . . . . . . . . . . . . T . . . . . . . . . . . . . . . . . . . . . . . L . . . . . . . . . . . . . T . H . . . . . . . P . . . . . . . . . . . . . . . . . . . . . . . . . KT229574.1_China_Mosquito**

**301 . . . . . . . . . . . . . . . . . . . . . . . . . . T . . . . . . . . . . . . . . . . . . . . . . . L . . . . . . . . . . . . . T . . . . . . . . . P . . . . . . . . . . . . . . . . . . . . . . . . . KT229575.1_China_Mosquito**

**301 . . . . . . . . . . . . . . . . . . . . . . . . . . . . . . . . . . . . . . . . . . . . . . S . . . . . . . . . . . . . . . . . . . . . . . . . . . . . . . . . . . . . . . . . . . . . . . . . . . . . LC461957.1_Japan_Mosquito**

**301 . . . . . . . . . . . . . . . . . . . . . . . . . . . . . . . . . . . . . . . . . . . . . . . . . . . . . . . . . . . . . . . . . . . . . . . . . . . . . . . . . . . . . . . . . . . . . . . . . . . . LC513838.1_Japan_Mosquito**

**301 . . . . . . . . G G T V L . . M . . T . A G V S V . . L . . . . . . . . . . . . . . . . . . . . . . . . . . . . . . . . . . . . . . . . . . . . . . . . . . . . . R . . P . . . . . . . . . . . . . . D LC579814.1_Indonesia_Mosquito**

**301 . . . . . . . . . . . . . . . . . . . . . . . . . . . . . . . . . . . . . . . . . . . . . . . . . . . . . . . . . . . . . . . . . . . . . . . . . . . . . . . . . . . . . . . . . . . . . . . . . . . . LC623822.1_Japan_Mosquito**

**301 . . . . . . . . . . . . . . . . . . . . . I . . . . . . . . . . . . . . . . . . . . . . . . . . . . . . . . . . . . . . . . . . . . . . . . . . . . . . . . . . . . . . . . . . . . . . . . . . . . . . MH385014.1_China_Mosquito**

**301 . . . . . . . . . . . . . . . . . . . . . . . . . . . . . . . . . . . . . . . . . . . . . . . . . . . . . . . . . . . . . . . . . . . . . . . . . . . . . . . . . . . . . . . . . . . . . . . . . . . . MK558811.1_China_Mosquito**

**301 . . . . . . . . . . . . . . . . . . . . . I . . . . . I . . . . . . . . . . . . . . . . . . . . . . . . . . . . . . . . . . . . . . . . . . . . . . . . . . . . . . . . . . . . . . . . . . . . . . . . MT254426.1_China_Mosquito**

**301 . . . . . . . . . . . . I . . . . . . . . . . . . . . . . . . . . . . . . . . . . . . . . . . . I . . . . . . . . . . . . . . . . . . . . . . . . . . . . . . . . . . . . . . . . . . . . . . . . . . . MT560941.1_China_Mosquito**

**301 . . . . . . . . . . . . . . . . . . . . . I . . . . . . . . . . . . . . . . . . . . . . . . . . . . . . . . . . . . . . . . . . . . . . . . . . . . . . . . . . . . . . . . . . . . . . . . . . . . . . ON875960_India_Assam_Mosquito**

**V Y V Q Y G R C T R T R H S K R S R R S V S V Q T H G E S S L V N K K E A W L D S T K A T R Y L M K T E N W I I R N P G Y A F L A A A L G W M L G S N S G Q R V V F T I L L L L V A P A Y S F N C L G M Majority**

**------------------+-------------------+-------------------+-------------------+-------------------+-------------------+-------------------+-------------------+-------------------+-------------------+-**

**210 220 230 240 250 260 270 280 290 300**

**------------------+-------------------+-------------------+-------------------+-------------------+-------------------+-------------------+-------------------+-------------------+-------------------+-**

**601 . . . . . . . . . . . . . . . . . . . . . . . . . . . . . . . . . . . . . . . . . . . . . . . . . . . . . . . . . . . . . . . . . . V . . . . . . . . N . . . . . . . . . . . . . . . . . . . . . . . . AF098735.1_Taiwan_Mosquito**

**601 . F . . . . . . . . . . . . . . . . . . . . . . . . . . . . . . . . . . . . . . . . . . . . . . . . . . . . . . . . . . . . . . . . . . . . . . . . . . . . . . . . . . . . . . . . . . . . . . . . . . GQ902060.1_Thailand_Mosquito**

**601 . . . . . . . . . . . . . . . . . . . . . . . . . . . . . . . . . . . . . . . . . . . . . . . . . . . . . . . . . . . . . . . . . . V . . . . . . . . N . . . . . . A . . . . . . . . . . . . . . . . . GQ902063.1_Thailand_Mosquito**

**601 . . . . . . . . . . . . . . . . . . . . . . . . . . . . . . . . . . . . . . . . . . . . . . . . . . . . . . . . . . . . . . . . . . . . . . . . . . . . . . . . . . . . . . . . . . . . . . . . . . . . HQ652538.1_China_Mosquito**

**601 . . . . . . . . . . . . . . . . . . . . . . . . . . . . . . . . . . . . . . . . . . . . . . . . . . . . . . . . . . . . . . . . . . . . . . . . . . . . . . . . . . . . . . . . . . . . . . . . . . . . JF499790.1_Taiwan_Mosquito**

**601 . . . . . . . . . . . . . . . . . . . . . . . . . . . . . . . . . . . . . . . . . . . . . . . . . . . . . . . . . . . . . . . . . . V . . . . . . . . N . . . . . . . . . . . . . . . . . . . . . . . . JN864064.1_China_Mosquito**

**601 . . . . . . . . . . . . . . . . . . . . . . . . . . . . . . . . . . . . . . . . . . . . . . . . . . . . . . . . . . . . . . . . . . . . . . . . . . . . . . . . . . . . . . . . . . . . . . . . . . . . JQ031753.1_Taiwan_Mosquito**

**601 . . . . . . . . . . . . . . . . . . . . . . . . . . . . . . . . . . . . . . . . . . . . . . . . . . . . . . . . . . . . . . . . . . V . . . . . . . . N . . . . . . . . . . . . . . . . . . . . . . . . JQ086762.1_China_Mosquito**

**601 . . . . . . . . . . . . . . . . . . K . . . . . . . . . . . . M . . . . . . . . . . . . . . . . . . . . . . . . . . . . . . . . . . . . . . . . . . . . . . . . . . . . . . . . . . . . . . . . . . . . KT229574.1_China_Mosquito**

**601 . . L . . . . . . . . . . . . . . . K . . . . . . . . . . . . M . . . . . . . . . . . . . . . . . . . . . . . . . . . . . . . . . . . . . . . . . . . . . . . . . . . . . . . . . . . . . . . . . . . . KT229575.1_China_Mosquito**

**601 . . . . . . . . . . . . . . . . . . . . . . . . . . . . . . . . . . . . . . . . . . . . . . . . . . . . . . . . . . . . . . . . . . . . . . . . . . . . . . . . . . . . . . . . . . . . . . . . . . . . LC461957.1_Japan_Mosquito**

**601 . . . . . . . . . . . . . . . . . . . . . . . . . . . . . . . . . . . . . . . . . . . . . . . . . . . . . . . . . . . . . . . . . . . . . . . . . . . . . . . . . . . . . . . . . . . . . . . . . . . . LC513838.1_Japan_Mosquito**

**601 . . . . . . . . . . . . . . . . . . . . . . . . . . . . . . . . . R . . . . . . . . . . . . . . . . . . . . . . . . . . . . . . . V . . . . . . . . . . . . . . . . . . . . . . . . . . . . . . . . . . LC579814.1_Indonesia_Mosquito**

**601 . . . . . . . . . . . . . . . . . . . . . . . . . . . . . . . . . . . . . . . . . . . . . . . . . . . . . . . . . . . . . . . . . . . . . . . . . . . . . . . . . . . . . . . . . . . . . . . . . . . . LC623822.1_Japan_Mosquito**

**601 . . . . . . . . . . . . . . . . . . . . . . . . . . . . . . . . . . . . . . . . . . . . . . . . . . . . . . . . . . . . . . . . . . . . . . . . . . . N . . . . . . . . . . . . . . . . . . . . . . . . MH385014.1_China_Mosquito**

**601 . . . . . . . . . . . . . . . . . . . . . . . . . . . . . . . . . . . . . . . . . . . . . . . . . . . . . . . . . . . . . . . . . . . . . . . . . . . . . . . . . . . . . . . . . . . . . . . . . . . . MK558811.1_China_Mosquito**

**601 . . . . . . . . . . . . . . . . . . . . . . . . . . . . . . . . . . . . . . . . . . . . . . . . . . . . . . . . . . . . . . . . . . . . . . . . . . . . . . . . . . . . . . . . . . . . . . . . . . . . MT254426.1_China_Mosquito**

**601 . . . . . . . . . . . . . . . . . . . . . . . . . . . . . . . . . . . . . . . . . . . . . . . . . . . . . . . . . . . . . . . . . . . . . . . . . . . . . . . . . . . . . . . . . . . . . . . . . . . . MT560941.1_China_Mosquito**

**601 . . . . . . . . . . . . . . . . . . . . . . . . . . . . . . . . . . . . . . . . . . . . . . . . . . . . . . . . . . . . . . . . . . . . . . . . . . . N . . . . . . . . . . . . . . . . . . . . . . . . ON875960_India_Assam_Mosquito**

**G N R D F I E G A S G A T W V D L V L E G D S C L T I M A N D K P T L D V R M I N I E A S Q L A E V R S Y C Y H A S V T D I S T V A R C P T T G E A H N E K R A D S S Y V C K Q G F T D R G W G N G C G Majority**

**------------------+-------------------+-------------------+-------------------+-------------------+-------------------+-------------------+-------------------+-------------------+-------------------+-**

**310 320 330 340 350 360 370 380 390 400**

**------------------+-------------------+-------------------+-------------------+-------------------+-------------------+-------------------+-------------------+-------------------+-------------------+-**

**901 . . . . . . . . . . . . . . . . . . . . . . . . . . . . . . . . . . . . . . . . . . . . . . . . . . . . . . . . . . . . . . . . . . . . . . . . . . . . . . . . . . . . . . . . . . . . . . . . . . . . AF098735.1_Taiwan_Mosquito**

**901 . . . . . . . . . . . . . . . . . . . . . . . . . . . . . . . . . . . . . . . . . . . . . . . . . . . . . . . . . . . . . . . . . . . . . . . . . . . . . . . . . . . . . . . . . . . . . . . . . . . . GQ902060.1_Thailand_Mosquito**

**901 . . . . . . . . . . . . . . . . . . . . . . . . . . . . . . . . . . . . . . . . . . . . . . . . . . . . . . . . . . . . . . . . . . . . . . . . . . . . . . . . . . . . . . . . . . . . . . . . . . . . GQ902063.1_Thailand_Mosquito**

**901 . . . . . . . . . . . . . . . . . . . . . . . . . . . . . . . . . . . . . . . . . . . . . . . . . . . . . . . . . . . . . . . . . . . . . . . . . . . . . . . . . . . . . . . . . . . . . . . . . . . . HQ652538.1_China_Mosquito**

**901 . . . . . . . . . . . . . . . . . . . . . . . . . . . . . . . . . . . . . . . . . . . . . . . . . . . . . . . . . . . . . . . . . . . . . . . . . . . . . . . . . . . . . . . . . . . . . . . . . . . . JF499790.1_Taiwan_Mosquito**

**901 . . . . . . . . . . . . . . . . . . . . . . . . . . . . . . . . . . . . . . . . . . . . . . . . . . . . . . . . . . . . . . . . . . . . . . . . . . . . . . . . . . . . . . . . . . . . . . . . . . . . JN864064.1_China_Mosquito**

**901 . . . . . . . . . . . . . . . . . . . . . . . . . . . . . . . . . . . . . . . . . . . . . . . . . . . . . . . . . . . . . . . . . . . . . . . . . . . . . . . . . . . . . . . . . . . . . . . . . . . . JQ031753.1_Taiwan_Mosquito**

**901 . . . . . . . . . . . . . . . . . . . . . . . . . . . . . . . . . . . . . . . . . . . . . . . . . . . . . . . . . . . . . . . . . . . . . . . . . . . . . . . . . . . . . . . . . . . . . . . . . . . . JQ086762.1_China_Mosquito**

**901 . . . . . . . . . . . . . . . . . . . . . . . . . . . . . . . . . . . . . . . . . . . . . . . . . . . . . . . . . . . . . . . . . . . . . . . . . . . . . . . . . . . . . . . . . . . . . . . . . . . . KT229574.1_China_Mosquito**

**901 . . . . . . . . . . . . . . . . . . . . . . . . . . . . . . . . . . . . . . . . . . . . . . . . . . . . . . . . . . . . . . . . . . . . . . . . . . . . . . . . . . . . . . . . . . . . . . . . . . . . KT229575.1_China_Mosquito**

**901 . . . . . . . . . . . . . . . . . . . . . . . . . . . . . . . . . . . . . . . . . . . . . . . . . . . . . . . . . . . . . . . . . . . . . . . . . . . . . . . . . . . . . . . . . . . . . . . . . . . . LC461957.1_Japan_Mosquito**

**901 . . . . . . . . . . . . . . . . . . . . . . . . . . . . . . . . . . . . . . . . . . . . . . . . . . . . . . . . . . . . . . . . . . . . . . . . . . . . . . . . . . . . . . . . . . . . . . . . . . . . LC513838.1_Japan_Mosquito**

**901 . . . . . . . . V . . . . . . . . . . . . . . . . . . . . H . R . . . . . . . V . . . . . . . . . . . . . . . . . . . . . . . . . . . . . . . . . . . . D . . . . . N . . . . . . . . . . . . . . . . . LC579814.1_Indonesia_Mosquito**

**901 . . . . . . . . . . . . . . . . . . . . . . . . . . . . . S . . . . . . . . . . . . . . . . . . . . . . . . . . . . . . . . . . . . . . . . . . . . . . . . . . . . . . . . . . . . . . . . . . . . . . LC623822.1_Japan_Mosquito**

**901 . . . . . . . . . . . . . . . . . . . . . . . . . . . . . . . . . . . . . . . . . . . . . . . . . . . . . . . . . . . . . . . . . . . . . . . . . . . . . . . . . . . . . . . . . . . . . . . . . . . . MH385014.1_China_Mosquito**

**901 . . . . . . . . . . . . . . . . . . . . . . . . . . . . . . . . . . . . . . . . . . . . . . . . . . . . . . . . . . . . . . . . . . . . . . . . . . . . . . . . . . . . . . . . . . . . . . . . . . . . MK558811.1_China_Mosquito**

**901 . . . . . . . . . . . . . . . . . . . . . . . . . . . . . . . . . . . . . . . . . . . . . . . . . . . . . . . . . . . . . . . . . . . . . . . . . . . . . . . . . . . . . . . . . . . . . . . . . . . . MT254426.1_China_Mosquito**

**901 . . . . . . . . . . . . . . . . . . . . . . . . . . . . . . . . . . . . . . . . . . . . . . . . . . . . . . . . . . . . . . . . . . . . . . . . . . . . . . . . . . . . . . . . . . . . . . . . . . . . MT560941.1_China_Mosquito**

**901 . . . . . . . . . . . . . . . . . . . . . . . . . . . . . . . . . . . . . . . . . . . . . . . . . . . . . . . . . . . . . . . . . . . . . . . . . . . . . . . . . . . . . . . . . . . . . . . . . . . . ON875960_India_Assam_Mosquito**

**L F G K G S I D T C A K F S C T S K A I G R M I Q P E N I K Y E V G I F V H G T T T S E N H G N Y S A Q V G A S Q A A K F T V T P N A P S I T L K L G D Y G E V T L D C E P R S G L N T E A F Y V M T V Majority**

**------------------+-------------------+-------------------+-------------------+-------------------+-------------------+-------------------+-------------------+-------------------+-------------------+-**

**410 420 430 440 450 460 470 480 490 500**

**------------------+-------------------+-------------------+-------------------+-------------------+-------------------+-------------------+-------------------+-------------------+-------------------+-**

**1201 . . . . . . . . . . . . . . . . . . . . . . T . . . . . . . . . . . . . . . . . . . . . . . . . . . . . . . . . . . . . . . . . . . . . . . . . . . . . . . . . . . . . . . . . . . . . . . . . . . . . AF098735.1_Taiwan_Mosquito**

**1201 . . . . . . . . . . . . . . . . . . . . . . T . . . . . . . . . . . V . . . . . . . . . . . . . . . . . . . . . . . . . . . . . . . . . . . . . . . . . . . . . . . . . . . . . . . . . . . . . . . . . GQ902060.1_Thailand_Mosquito**

**1201 . . . . . . . . . . . . . . . . . . . . . . T . . . . . . . . . . . . . . . . . . . . . . . . . . . . . . . . . . . . . . . . . . . . . . . . . . . . . . . . . . . . . . . . . . . . . . . . . . . . . GQ902063.1_Thailand_Mosquito**

**1201 . . . . . . . . . . . . . . . . . . . . . . . . . . . . . . . . . . . . . . . . . . . . . . . . . . . . . . . . . . . . . . . . . . . . . . . . . . . . . . . . . . . . . . . . . . . . . . . . . . . . HQ652538.1_China_Mosquito**

**1201 . . . . . . . . . . . . . . . . N . . . . . . . . . . . . . . . . . . . . . . . . . . . . . . . . . . . . . . . . . . . . . . . . . . . . . . . . . . . . . . . . . . . . . . . . . . . . . . . . . . . JF499790.1_Taiwan_Mosquito**

**1201 F . . . . . . . . . . . . . . . . . . . . . T . . . . . . . . K . . . . . . . . . . . . . . . . . . . . . . . . . . V . . . . . . . . . . V A . . . . . . . . D . . . . . . . . . . . . . . . . . . . . JN864064.1_China_Mosquito**

**1201 . . . . . . . . . . . . . . . . N . . . . . . . . . . . . . . . . . . . . . . . . . . . . . . . . . . . . . . . . . . . . . . . . . . . . . . . . . . . . . . . . . . . . . . . . . . . . . . . . . . . JQ031753.1_Taiwan_Mosquito**

**1201 F . . . . . . . . . . . . . . . . . . . . . T . . . . . . . . K . . . . . . . . . . . . . . . . . . . . . . . . . . V . . . . . . . . . . V A . . . . . . . . D . . . . . . . . . . . . . . . . . . . . JQ086762.1_China_Mosquito**

**1201 . . . . . . . . . . . . . . . . . . . . . . . . . . . . . . . . . . . . . . . . . . . . . . . . . . . . . . . . . . . . . . . . . . . . . . . . . . . . . . . . . . . . . . . . . . . . . . . . . . . . KT229574.1_China_Mosquito**

**1201 . . . . . . . . . . . . . . . . . . . . . . . . . . . . . . . . . . . . . . . . . . . . . . . . . . . . . . . . . . . . . . . . . . . . . . . . . . . . . . . . . . . . . . . . . . . . . . . . . . . . KT229575.1_China_Mosquito**

**1201 . . . . . . . . . . . . . . . . N . . . . . . . . . . . . . . . . . . . . . . . . . . . . . . . . . . . . . . . . . . . . . . . . . . . . . . . . . . . . . . . . . . . . . . . . . . . . . . . . . . . LC461957.1_Japan_Mosquito**

**1201 . . . . . . . . . . . . . . . . N . . . . . . . . . . . . . . . . . . . . . . . . . . . . . . . . . . . . . . . . . . . . . . . . . . . . . . . . . . . . . . . . . . . . . . . . . . . . . . . . . . . LC513838.1_Japan_Mosquito**

**1201 . . . . . . . . . . . . . . . . . . . T . K T . . . . . . . . . . . . . . . . . . . . . . . . . . T . . I . . . . . . . . . I . . . . . . . . . . . . . . . . . . . . . . . . . . . . . . . . . . . . . LC579814.1_Indonesia_Mosquito**

**1201 . . . . . . . . . . . . . . . . . . . . . . . . . . . . . . . . . . . . . . . . . . . . . . . . . . . . . . . . . . . . . . . . . . . . . . . . . . . . . . . . . . . . . . . . . . . . . . . . . . . . LC623822.1_Japan_Mosquito**

**1201 . . . . . . . . . . . . . . . . N . . . . . . . . . . . . . . . . . . . . . . . . . . . . . . . . . . . . . . . . . . . . . . . . . . . . . . . . . . . . . . . . . . . . . . . . . . . . . . . . . . . MH385014.1_China_Mosquito**

**1201 . . . . . . . . . . . . . . . . . . . . . . . . . . . . . . . . . . . . . . . . . . . . . . . . . . . . . . . . . . . . . . . . . . . . . . . . . . . . . . . . . . . . . . . . . . . . . . . . . . . . MK558811.1_China_Mosquito**

**1201 . . . . . . . . . . . . . . . . N . . . . . . . . . . . . . . . . . . . . . . . . . . . . . . . . . . . . . . . . . . . . . . . . . . . . . . . . . . . . . . . . . . . . . . . . . . . . . . . . . . . MT254426.1_China_Mosquito**

**1201 . . . . . . . . . . . . . . . . . . . . . . . . . . . . . . . . . . . . . . . . . . . . . . . . . . . . . . . . . . . . . . . . . . . . . . . . . . . . . . . . . . . . . . . . . . . . . . . . . . . . MT560941.1_China_Mosquito**

**1201 . . . . . . . . . . . . . . . . N . . . . . . . . . . . . . . . . . . . . . . . . . . . . . . . . . . . . . . . . . . . . . . . . . . . . . . . . . . . . . . . . . . . . . . . . . . . . . . . . . . . ON875960_India_Assam_Mosquito**

**G S K S F L V H R E W F H D L S L P W T S P S S T A W R N R E L L M E F E E A H A T K Q S V V A L G S Q E G G L H Q A L A G A I V V E Y S S S V K L T S G H L K C R L K M D K L A L K G T T Y G M C T E Majority**

**------------------+-------------------+-------------------+-------------------+-------------------+-------------------+-------------------+-------------------+-------------------+-------------------+-**

**510 520 530 540 550 560 570 580 590 600**

**------------------+-------------------+-------------------+-------------------+-------------------+-------------------+-------------------+-------------------+-------------------+-------------------+-**

**1501 . . . . . . . . . . . . . . . A . . . . . . . . . . . . . . . . . . . . . . . . . . . . . . . . . . . . . . . . . . . . . . . . . . . . . . . . . . . . . . . . . . . . . . . . . . . . . . . . . . . . AF098735.1_Taiwan_Mosquito**

**1501 . . . . . . . . . . . . . . . . . . . . . . . . . . . . . . . . . . . . . . . . . . . . . . . . . . . . . . . . . . . . . . . . . . . . . . . . . . . . . . . . . . . . . . . . . . . . . . . . . . . . GQ902060.1_Thailand_Mosquito**

**1501 . . . . . . . . . . . . . . . A . . . . . . . . . . . . . . . . . . . . . . . . . . . . . . . . . . . . . . . . . . . . . . . . . . . . . . . . . . . . . . . . . . . . . . . . . . . . . . . . . . . . GQ902063.1_Thailand_Mosquito**

**1501 . . . . . . . . . . . . . . . . . . . . . . . . . . . . . . . . . . . . . . . . . . . . . . . . . . . . . . . . . . . . . . . . . . . . . . . . . . . . . . . . . . . . . . . . . . . . . . . . . . . . HQ652538.1_China_Mosquito**

**1501 . . . . . . . . . . . . . . . . . . . . . . . . . . . . . . . . . . . . . . . . . . . . . . . . . . . . . . . . . . . . . . . . . . . . . . . . . . . . . . . . . . . . . . . . . . . . . . . . . . . . JF499790.1_Taiwan_Mosquito**

**1501 . . . . . . . . . . . . . . . A . . . . . . . . . . . . . . . . . . . . . G . . . . . . . . . . . . . . . . . . . H . . . . . . . . . . . . . . . . . . . . . . . . . . . . . . . . . . . . . . . . . . JN864064.1_China_Mosquito**

**1501 . . . . . . . . . . . . . . . . . . . . . . . . . . . . . . . . . . . . . . . . . . . . . . . . . . . . . . . . . . . . . . . . . . . . . . . . . . . . . . . . . . . . . . . . . . . . . . . . . . . . JQ031753.1_Taiwan_Mosquito**

**1501 . . . . . . . . . . . . . . . A . . . . . . . . . . . . . . . . . . . . . G . . . . . . . . . . . . . . . . . . . H . . . . . . . . . . . . . . . . . . . . . . . . . . . . . . . . . . . . . . . . . . JQ086762.1_China_Mosquito**

**1501 . . . . . . . . . . . . . . . . . . . . . . . . . . . . . . . . . . . . . . . . . . . . . . . . . . . . . . . . . . . . . . . . . . . . . . . . . . . . . . . . . . . . . . . . . . . . . . . . . . . . KT229574.1_China_Mosquito**

**1501 . . . . . . . . . . . . . . . . . . . . . . . . . . . . . . . . . . . . . . . . . . . . . . . . . . . . . . . . . . . . . . . . . . . . . . . . . . . . . . . . . . . . . . . . . . . . . . . . . . . . KT229575.1_China_Mosquito**

**1501 . . . . . . . . . . . . . . . . . . . . . . . . . . . . . . . . . . . . . . . . . . . . . . . . . . . . . . . . . . . . . . . . . . . . . . . . . . . . . . . . . . . . . . . . . . . . . . . . . . . . LC461957.1_Japan_Mosquito**

**1501 . . . . . . . . . . . . . . . . . . . . . . . . . . . . . . . . . . . . . . . . . . . . . . . . . . . . . . . . . . . . . . . . . . . . . . . . . . . . . . . . . . . . . . . . . . . . . . . . . . . . LC513838.1_Japan_Mosquito**

**1501 . . . . . . . . . . . . . . . A . . . . . A . N . . . . . . . . . . . . . . . . . . . . . . . . . . . . . . A . . . . . . . . . . . . . . . . . . . . . . . . . . . . . . . . . T . . . . . . . . . . . LC579814.1_Indonesia_Mosquito**

**1501 . . . . . . . . . . . . . . . . . . . . . . . . . . . . . . . . . . . . . . . . . . . . . . . . . . . . . . . . . . . . . . . . . . . . . . . . . . . . . . . . . . . . . . . . . . . . . . . . . . . . LC623822.1_Japan_Mosquito**

**1501 . . . . . . . . . . . . . . . . . . . . . . . . . . . . . . . . . . . . . . . . . . . . . . . . . . . . . . . . . . . . . . . . . . . . . . . . . . . . . . . . . . . . . . . . . . . . . . . . . . . . MH385014.1_China_Mosquito**

**1501 . . . . . . . . . . . . . . . . . . . . . . . . . . . . . . . . . . . . . . . . . . . . . . . . . . . . . . . . . . . . . . . . . . . . . . . . . . . . . . . . . . . . . . . . . . . . . . . . . . . . MK558811.1_China_Mosquito**

**1501 . . . . . . . . . . . . . . . . . . . . . . . . . . . . . . . . . . . . . . . . . . . . . . . . . . . . . . . . . . . . . . . . . . . . . . . . . . . . . . . . . . . . . . . . . . . . . . . . . . . . MT254426.1_China_Mosquito**

**1501 . . . . . . . . . . . . . . . . . . . . . . . . . . . . . . . . . . . . . . . . . . . . . . . . . . . . . . . . . . . . . . . . . . . . . . . . . . . . . . . . . . . . . . . . . . . . . . . . . . . . MT560941.1_China_Mosquito**

**1501 . . . . . . . . . . . . . . . . . . . . . . . . . . . . . . . . . . . . . . . . . . . . . . . . . . . . . . . . . . . . . . . . . . . . . . . . . . . . . . . . . . . . . . . . . . . . . . . . . . . . ON875960_India_Assam_Mosquito**

**K F S F A K N P A D T G H G T V V I E L T Y S G S D G P C K I P I V S V A S L N D M T P V G R L V T V N P F V A T S S S N S K V L V E M E P P F G D S Y I V V G R G D K Q I N H H W H K A G S T L G K A Majority**

**------------------+-------------------+-------------------+-------------------+-------------------+-------------------+-------------------+-------------------+-------------------+-------------------+-**

**610 620 630 640 650 660 670 680 690 700**

**------------------+-------------------+-------------------+-------------------+-------------------+-------------------+-------------------+-------------------+-------------------+-------------------+-**

**1801 . . . . . . . . . . . . . . . . . . . . S . . . . . . . . . . . . . . . . . . . . . . . . . . . . . . . . . . . . . . A . . . . . . . . . . . . . . . . . . . . . . H . . . . . . . . . . . . . . . . . AF098735.1_Taiwan_Mosquito**

**1801 . . . . . . . . . . . . . . . . . . . . . . . . . . . . . . . . . . . . . . . . . . . . . . . . . . . . . . . . . . . . . . . . . . . . . . . . . . . . . . . . . . . . . . . . . . . . . . . . . . . . GQ902060.1_Thailand_Mosquito**

**1801 . . . . . . . . . . . . . . . . . . . . S . . . . . . . . . . . . . . . . . . . . . . . . . . . . . . . . . . . A . . A . . . . . . . . . . . . . . . . . . . . . . . . . . . . . . . . . . . . . . . . GQ902063.1_Thailand_Mosquito**

**1801 . . . . . . . . . . . . . . . . . . . . . . . . . . . . . . . . . . . . . . . . . . . . . . . . . . . . . . . . . . . . . . . . . . . . . . . . . . . . . . . . . . . . . . . . . . . . . . . . . . . . HQ652538.1_China_Mosquito**

**1801 . . . . . . . . . . . . . . . . . . . . . . . . . . . . . . . . . . . . . . . . . . . . . . . . . . . . . . . . . . . . . . . . . . . . . . . . . . . . . . . . . . . . . . . . . . . . . . . . . . . . JF499790.1_Taiwan_Mosquito**

**1801 . . . . . . . . V . . . . . . . . . . . S . . . . . . . . . . . . . . . . . . . . . . . . . . . . . . . . . . . . . . A . . . . . . . . . . . . . . . . . . . . . . . . . . . . . . . . . . . . . . . . JN864064.1_China_Mosquito**

**1801 . . . . . . . . . . . . . . . . . . . . . . . . . . . . . . . . . . . . . . . . . . . . . . . . . . . . . . . . . . . . . . . . . . . . . . . . . . . . . . . . . . . . . . . . . . . . . . . . . . . . JQ031753.1_Taiwan_Mosquito**

**1801 . . . . . . . . V . . . . . . . . . . . S . . . . . . . . . . . . . . . . . . . . . . . . . . . . . . . . . . . . . . A . . . . . . . . . . . . . . . . . . . . . . . . . . . . . . . . . . . . . . . . JQ086762.1_China_Mosquito**

**1801 . . . . . . . . . . . . . . . . . . . . . . . . . . . . . . . . . . . . . . . . . . . . . . . . . . . . . . . . . . . . . . . . . . . . . . . . . . . . . . . . . . . . . . . . . . . . . . . . . . . . KT229574.1_China_Mosquito**

**1801 . . . . . . . . . . . . . . . . . . . . . . . . . . . . . . . . . . . . . . . . . . . . . . . . . . . . . . . . . . . . . . . . . . . . . . . . . . . . . . . . . . . . . . . . . . . . . . . . . . . . KT229575.1_China_Mosquito**

**1801 . . . . . . . . . . . . . . . . . . . . . . . . . . . . . . . . . . . . . . . . . . . . . . . . . . . . . . . . . . . T . . . . . . . . . . . . . . . . . . . . . . . . . . . . . . . . . . . . . . . . LC461957.1_Japan_Mosquito**

**1801 . . . . . . . . . . . . . . . . . . . . . . . . . . . . . . . . . . . . . . . . . . . . . . . . . . . . . . . . . . . . . . . . . . . . . . . . . . . . . . . . . . . . . . . . . . . . . . . . . . . . LC513838.1_Japan_Mosquito**

**1801 . . . . . . . . . . . . . . . . . . . . Q . . . . . . . . . . . . . . . . . . . . . . . . . . . . . . . . . . . . . . . . . . . . . . . . . . . . . . . . . . . . . . . . . . . . . . . P . . . . . . . LC579814.1_Indonesia_Mosquito**

**1801 . . . . . . . . . . . . . . . . . . . . . . . . . . . . . . . . . I . . . . . . . . . . . . . . . . . . . . . . . . . . . . . . . . . . . . . . . . . . . . . . . . . . . . . . . . . . . . . . . . . . LC623822.1_Japan_Mosquito**

**1801 . . . . . . . . . . . . . . . . . . . . . . . . . . . . . . . . . A . . . . . . . . . . . . . . . . . . . . . . . . . . . . . . . . . . . . . . . . . . . . . . . . . . . . . . . . . . . . . . . . . . MH385014.1_China_Mosquito**

**1801 . . . . . . . . . . . . . . . . . . . . . . . . . . . . . . . . . . . . . . . . . . . . . . . . . . . . . . . . . . . . . . . . . . . . . . . . . . . . . . . . . . . . . . . . . . . . . . . . . . . . MK558811.1_China_Mosquito**

**1801 . . . . . . . . . . . . . . . . . . . . . . . . . . . . . . . . . . . . . . . . . . . . . . . . . . . . . . . . . . . . . . . . . . . . . . . . . . . . . . . . . . . . . . . . . . . . . . . . . . . . MT254426.1_China_Mosquito**

**1801 . . . . . . . . . . . . . . . . . . . . . . . . . . . . . . . . . . . . . . . . . . . . . . . . . . . . . . . . . . . . . . . . . . . . . . . . . . . . . . . . . . . . . . . . . . . . . . . . . . . . MT560941.1_China_Mosquito**

**1801 . . . . . . . . . . . . . . . . . . . . . . . . . . . . . . . . . . . . . . . . . . . . . . . . . . . . . . . . . . . . . . . . . . . . . . . . . . . . . . . . . . . . . . . . . . . . . . . . . . . . ON875960_India_Assam_Mosquito**

**F S T T L K G A Q R L A A L G D T A W D F G S I G G V F N S I G K A V H Q V F G G A F R T L F G G M S W I T Q G L M G A L L L W M G V N A R D R S I A L A F L A T G G V L V F L A T N V H A D T G C A I Majority**

**------------------+-------------------+-------------------+-------------------+-------------------+-------------------+-------------------+-------------------+-------------------+-------------------+-**

**710 720 730 740 750 760 770 780 790 800**

**------------------+-------------------+-------------------+-------------------+-------------------+-------------------+-------------------+-------------------+-------------------+-------------------+-**

**2101 . . . . . . . . . . . . . . . . . . . . . . . . . . . . . . . . . . . . . . . . . . . . . . . . . . . . . . . . . . . . . . . . . . . . . . . . . . . . . . . . . . . . . . . . . . . . . . . . . . . . AF098735.1_Taiwan_Mosquito**

**2101 . . . . . . . . . . . . . . . . . . . . . . . . . . . . . . . . . . . . . . . . . . . . . . . . . . . . . . . . . . . . . . . . . . . . . . . . . . . . . . . . . . . . . . . . . . . . . . . . . . . . GQ902060.1_Thailand_Mosquito**

**2101 . . . . . . . . . . . . . . . . . . . . . . . . . . . . . . . . . . . . . . . . . . . . S . . . . . . . . . . . . . . . . . . . . . . . . . . . . . . . . . . . . . . . . . . . . . . . . . . . . . . . GQ902063.1_Thailand_Mosquito**

**2101 . . . . . . . . . . . . . . . . . . . . . . . . . . . . . . . . . . . . . . . . . . . . . . . . . . . . . . . . . . . . . . . . . . . . . . . . . . . . . . . . . . . . . . . . . . . . . . . . . . . . HQ652538.1_China_Mosquito**

**2101 . . . . . . . . . . . . . . . . . . . . . . . . . . . . . . . . . . . . . . . . . . . . . . . . . . . . . . . . . . . . . . . . . . . . . . . . . . . . . . . . . . . . . . . . . . . . . . . . . . . . JF499790.1_Taiwan_Mosquito**

**2101 . . . . . . . . . . . . . . . . . . . . . . . . . . . . . . . . R . . . . . . . . . . . . . . . . . . . . . . . . . . . . . . . . . . . . . . . . . . . . . . . . . . . . . . . . . . . . . . . . . . . JN864064.1_China_Mosquito**

**2101 . . . . . . . . . . . . . . . . . . . . . . . . . . . . . . . . . . . . . . . . . . . . . . . . . . . . . . . . . . . . . . . . . . . . . . . . . . . . . . . . . . . . . . . . . . . . . . . . . . . . JQ031753.1_Taiwan_Mosquito**

**2101 . . . . . . . . . . . . . . . . . . . . . . . . . . . . . . . . R . . . . . . . . . . . . . . . . . . . . . . . . . . . . . . . . . . . . . . . . . . . . . . . . . . . . . . . . . . . . . . . . . . . JQ086762.1_China_Mosquito**

**2101 . . . . . . . . . . . . . . . . . . . . . . . . . . . . . . . . . . . . . . . . . . . . . . . . . . . . . . . . . . . . . . . . . . . . . . . . . . . . . . . . . . . . . . . . . . . . . . . . . . . . KT229574.1_China_Mosquito**

**2101 . . . . . . . . . . . . . . . . . . . . . . . . . . . . . . . . . . . . . . . . . . . . . . . . . . . . . . . . . . . . . . . . . . . . . . . . . . . . . . . . . . . . . . . . . . . . . . . . . . . . KT229575.1_China_Mosquito**

**2101 . . . . . . . . . . . . . . . . . . . . . . . . . . . . . . . . . . . . . . . . . . . . . . . . . . . . . . . . . . . . . . . . . . . . . . . . . . . . V . . . . . . . . . . . . . . . . . . . . . . . LC461957.1_Japan_Mosquito**

**2101 . . . . . . . . . . . . . . . . . . . . . . . . . . . . . . . . . . . . . . . . . . . . . . . . . . . . . . . . . . . . . . . . . . . . . . . . . . . . . . . . . . . . . . . . . . . . . . . . . . . . LC513838.1_Japan_Mosquito**

**2101 . . . . . . . . . . . . . . . . . . . . . . . . . . . . . . . . . . . . . . . . . . . . . . . . . . . . . . . . . . . . . . . . . . . . . . . . . . . M . . . V . . . T . L . . . . . . . . . . . . . . LC579814.1_Indonesia_Mosquito**

**2101 . . . . . . . . . . . . . . . . . . . . . . . . . . . . . . . . . . . . . . . . . . . . . . . . . . . . . . . . . . . . . . . . . . . . . . . . . . . . . . . S . . . . . . . . . . . . . . . . . . . . LC623822.1_Japan_Mosquito**

**2101 . . . . . . . . . . . . . . . . . . . . . . . . . . . . . . . . . . . . . . . . . . . . . . . . . . . . . . . . . . . . . . . . . . . . . . . . . . . . . . . . . . . . . . . . . . . . . . . . . . . . MH385014.1_China_Mosquito**

**2101 . . . . . . . . . . . . . . . . . . . . . . . . . . . . . . . . . . . . . . . . . . . . . . . . . . . . . . . . . . . . . . . . . . . . . . . . . . . . . . . . . . . . . . . . . . . . . . . . . . . . MK558811.1_China_Mosquito**

**2101 . . . . . . . . . . . . . . . . . . . . . . . . . . L . . . . . . . . . . . . . . . . . . . . . . . . . . . . . . . . . . . . . . . . . . . . . . . . . . . . . . . . . . . . . . . . . . . . . . . . . MT254426.1_China_Mosquito**

**2101 . . . . . . . . . . . . . . . . . . . . . . . . . . . . . . . . . . . . . . . . . . . . . . . . . . . . . . . . . . . . . . . . . . . . . . . . . . . . . . . . . . . . . . . . . . . . . . . . . . . . MT560941.1_China_Mosquito**

**2101 . . . . . . . . . . . . . . . . . . . . . . . . . . . . . . . . . . . . . . . . . . . . . . . . . . . . . . . . . . . . . . . . . . . . . . . . . . . . . . . . . . . . . . . . . . . . . . . . . . . . ON875960_India_Assam_Mosquito**

**D I T R K E M R C G S G I F V H N D V E A W V D R Y K Y L P E T P R S L A K I V H K A H Q E G V C G V R S V T R L E H Q M W E S V R D E L N V L L K E N A V D L S V V V N K P V G R Y R S A P K R L S M Majority**

**------------------+-------------------+-------------------+-------------------+-------------------+-------------------+-------------------+-------------------+-------------------+-------------------+-**

**810 820 830 840 850 860 870 880 890 900**

**------------------+-------------------+-------------------+-------------------+-------------------+-------------------+-------------------+-------------------+-------------------+-------------------+-**

**2401 . . . . . . . . . . . . . . . . . . . . . . . . . . . . . . . . . . . . . . . . . . . . K . . . . . . . . . . . . . . . . . . A . . . . . . . . . . . . . . . . . . . . . . . . . . . . . . . . . . . . AF098735.1_Taiwan_Mosquito**

**2401 . . . . . . . . . . . . . . . . . . . . . . . . . . . . . . . . . . . . . . . . . . . . . . . . . . . . . . . . . . . . . . . . . . . . . . . . . . . . . . . . . . . . . . . . . . . . . . . . . . . . GQ902060.1_Thailand_Mosquito**

**2401 . . . . . . . . . . . . . . . . . . . . . . . . . . . . . . . . . . . . . . . . . . . . K . . . . . . . . . . . . . . . . . . A . . . . . . . . . . . . . . . . . . . . . . . . . . . . . . . . . . . . GQ902063.1_Thailand_Mosquito**

**2401 . . . . . . . . . . . . . . . . . . . . . . . . . . . . . . . . . . . . . . . . . . . . . . . . . . . . . . . . . . . . . . . . . . . . . . . . . . . . . . . . . . . . . . . . . . . . . . . . . . . . HQ652538.1_China_Mosquito**

**2401 . . . . . . . . . . . . . . . . . . . . . . . . . . . . . . . . . . . . . . . . . . . . . . . . . . . . . . . . . . . . . . . . . . . . . . . . . . . . . . . . . . . . . . . . . . . . . . . . . . . . JF499790.1_Taiwan_Mosquito**

**2401 . . . . . . . . . . . . . . . . . . . . . . . . . . . . . . . . . . . . . . . . . . . . K . . . . . . . . . . . . . . . . . . A . . . . . . . . . . . . . . . . . . . . . . . . . . . . . . . . . . . . JN864064.1_China_Mosquito**

**2401 . . . . . . . . . . . . . . . . . . . . . . . . . . . . . . . . . . . . . . . . . . . . . . . . . . . . . . . . . . . . . . . . . . . . . . . . . . . . . . . . . . . . . . . . . . . . . . . . . . . . JQ031753.1_Taiwan_Mosquito**

**2401 . . . . . . . . . . . . . . . . . . . . . . . . . . . . . . . . . . . . . . . . . . . . K . . . . . . . . . . . . . . . . . . A . . . . . . . . . . . . . . . . . . . . . . . . . . . . . . . . . . . . JQ086762.1_China_Mosquito**

**2401 . . . . . . . . . . . . . . . . . . . . . . . . . . . . . . . . . . . . . . . . . . . . . . . . . . . . . . . . . . . . . . . . . . . . . . . . . . . . . . . . . . . . . . . . . . . . . . . . . . . . KT229574.1_China_Mosquito**

**2401 . . . . . . . . . . . . . . . . . . . . . . . . . . . . . . . . . . . . . . . . . . . . . . . . . . . . . . . . . . . . . . . . . . . . . . . . . . . . . . . . . . . . . . . . . . . . . . . . . . . . KT229575.1_China_Mosquito**

**2401 . . . . . . . . . . . . . . . . . . . . . . . . . . . . . . . . . . . . . . . . . . . . . . . . . . . . . . . . . . . . . . . . . . . . . . . . . . . . . . . . . . . . . . . . . . . . . . . . . . . . LC461957.1_Japan_Mosquito**

**2401 . . . . . . . . . . . . . . . . . . . . . . . . . . . . . . . . . . . . . . . . . . . . . . . . . . . . . . . . . . . . . . . . . . . . . . . . . . . . . . . . . . . . . . . . . . . . . . . . . . . . LC513838.1_Japan_Mosquito**

**2401 . . . . . . . . . . . . . . . . . . . . . . . . . . . . . . . . . . . . . . . . . . . . M . . I . . . . . . . . . . . . . . . A . . . . . . . . . . . . . . . . . . . . . . . . . . . . . . . . . . . . LC579814.1_Indonesia_Mosquito**

**2401 . . . . . . . . . . . . . . . . . . . . . . . . . . . . . . . . . . . . . . . . . . . . . . . . . . . . . . . . . . . . . . . . . . . . . . . . . . . . . . . . . . . . . . . . . . . . . . . . . . . . LC623822.1_Japan_Mosquito**

**2401 . . . . . . . . . . . . . . . . . . . . . . . . . . . . . . . . . . . . . . . . . . . . . . . . . . . . . . . . . . . . . . . . . . . . . . . . . . . . . . . . . . . . . . . . . . . . . . . . . . . . MH385014.1_China_Mosquito**

**2401 . . . . . . . . . . . . . . . . . . . . . . . . . . . . . . . . . . . . . . . . . . . . . . . . . . . . . . . . . . . . . . . . . . . . . . . . . . . . . . . . . . . . . . . . . . . . . . . . . . . . MK558811.1_China_Mosquito**

**2401 . . . . . . . . . . . . . . . . . . . . . C . . . . . . . . . . . . . . . . . . . . . . . . . . . . . . . . . . . . . . . . . . . . . . . . . . . . . . . . . . . . . . . . . . . . . . . . . . . . . . MT254426.1_China_Mosquito**

**2401 . . . . . . . . . . . . . . . . . . . . . . . . . . . . . . . . . . . . . . . . . . . . . . . . . . . . . . . . . . . . . . . . . . . . . . . . . . . . . . . . . . . . . . . . . . . . . . . . . . . . MT560941.1_China_Mosquito**

**2401 . . . . . . . . . . . . . . . . . . . . . . . . . . . . . . . . . . . . . . . . . . . . . . . . . . . . . . . . . . . . . . . . . . . . . . . . . . . . . . . . . . . . . . . . . . . . . . . . . . . . ON875960_India_Assam_Mosquito**

**T Q E K F E M G W K A W G K S I L F A P E L A N S T F V V D G P E T K E C P D E R R A W N S M Q I E D F G F G I T S T R V W L K I R E E N T D E C D G A I I G T A V K G H V A V H S D L S Y W I E S R L Majority**

**------------------+-------------------+-------------------+-------------------+-------------------+-------------------+-------------------+-------------------+-------------------+-------------------+-**

**910 920 930 940 950 960 970 980 990 1000**

**------------------+-------------------+-------------------+-------------------+-------------------+-------------------+-------------------+-------------------+-------------------+-------------------+-**

**2701 . . . . . . . . . . . . . . . . . . . . . . . . . . . . . . . . . . . . . . . . H . . . . . . . . . . . . . . . . . . . . . . . . . . . S . . . . . . . . . . . . . . . . . . . . . . . . . . . . . . Y AF098735.1_Taiwan_Mosquito**

**2701 . . . . . . . . . . . . . . . . . . . . . . . . . . . . . . . . . . . . . . . . . . . . . . . . . . . . . . . . . . . . . . . . . . . . D . . . . . . . . . . . . . . . . . . . . . . . . . . . . . . . GQ902060.1_Thailand_Mosquito**

**2701 . . . . . . . . . . . . . . . . . . . . . . . . . . . . . . . . . . . . . . . . H . . . . . . . . . . . . . . . . . . . . . . . . . . . S . . . . . . . . . . . . . . . . . . . . . . . . . . . . . . Y GQ902063.1_Thailand_Mosquito**

**2701 . . . . . . . . . . . . . . . . . . . . . . . . . . . . . . . . . . . . . . . . . . . . . . . . . . . . . . . . . . . . . . . . . . . . . . . . . . . . . . . . . . . . . . . . . . . . . . . . . . . . HQ652538.1_China_Mosquito**

**2701 . . . . . . . . . . . . . . . . . . . . . . . . . . . . . . . . . . . . . . . . . . . . . . . . . . . . . . . . . . . . . . . . . . . . . . . . . . . . . . . . . . . . . . . . . . . . . . . . . . . . JF499790.1_Taiwan_Mosquito**

**2701 . . . . . . . . . . . . . . . . . . . . . . . . . . . . . . . . . . . . . . . . H . . . . . . . . . . . . . . . . . . . . . . . . . . . S . . . . . . . N . . . . . . . . . . . . . . . . . . . . . . Y JN864064.1_China_Mosquito**

**2701 . . . . . . . . . . . . . . . . . . . . . . . . . . . . . . . . . . . . . . . . . . . . . . . . . . . . . . . . . . . . . . . . . . . . . . . . . . . . . . . . . . . . . . . . . . . . . . . . . . . . JQ031753.1_Taiwan_Mosquito**

**2701 . . . . . . . . . . . . . . . . . . . . . . . . . . . . . . . . . . . . . . . . H . . . . . . . . . . . . . . . . . . . . . . . . . . . S . . . . . . . N . . . . . . . . . . . . . . . . . . . . . . Y JQ086762.1_China_Mosquito**

**2701 . . . . . . . . . . . . . . . . . . . . . . . . . . . . . . . . . . . . . . . . . . . . . . . . . . . . . . . . . . . . . . . . . . . . . . . . . . . . . . . . . . . . . . . . . . . . . . . . . . . . KT229574.1_China_Mosquito**

**2701 . . . . . . . . . . . . . . . . . . . . . . . . . . . . . . . . . . . . . . . . . . . . . . . . . . . . . . . . . . . . . . . . . . . . . . . . . . . . . . . . . . . . . . . . . . . . . . . . . . . . KT229575.1_China_Mosquito**

**2701 . . . . . . . . . . . . . . . . . . . . . . . . . . . . . . . . . . . . . . . . . . . . . . . . . . . . . . . . . . . . . . . . . . . . . . . . . . . . . . . . . . . . . . . . . . . . . . . . . . . . LC461957.1_Japan_Mosquito**

**2701 . . . . . . . . . . . . . . . . . . . . . . . . . . . . . . . . . . . . . . . . . . . . . . . . . . . . . . . . . . . . . . . . . . . . . . . . . . . . . . . . . . . . . . . . . . . . . . . . . . . . LC513838.1_Japan_Mosquito**

**2701 . . . . . . . . . . . . . . . . . . . . . . . . . . . . . . . . . . . . . . . . H . . . . . . . . . . . . . . . . . . . . . . . . . . . . . . . . . . T . . . . . I . . . . . . . . . . . . . . . . H . LC579814.1_Indonesia_Mosquito**

**2701 . . . . . . . . . . . . . . . . . . . . . . . . . . . . . . . . . . . . . . . . . . . . . . . . . . . . . . . . . . . . . . . . . . . . D . . . . . . . . . . . . . . . . . . . . . . . . . . . . . . . LC623822.1_Japan_Mosquito**

**2701 . . . . . . . . . . . . . . . . . . . . . . . . . . . . . . . . . . . . . . . . . . . . . . . . . . . . . . . . . . . . . . . . . . . . . . . . . . . . . . . . . . . . . . . . . . . . . . . . . . . . MH385014.1_China_Mosquito**

**2701 . . . . . . . . . . . . . . . . . . . . . . . . . . . . . . . . . . . . . . . . . . . . . . . . . . . . . . . . . . . . . . . . . . . . D . . . . . . . . . . . . . . . . . . . . . . . . . . . . . . . MK558811.1_China_Mosquito**

**2701 . . . . . . . . . . . . . . . . . . . . . . . . . . . . . . . . . . . . . . . . . . . . . . . . . . . . . . . . . . . . . . . . . . . . . . . . . . . . . . . . . . . . . . . . . . . . . . . . . . . . MT254426.1_China_Mosquito**

**2701 . . . . . . . . . . . . . . . . . . . . . . . . . . . . . . . . . . . . . . . . . . . . . . . . . . . . . . . . . . . . . . . . . . . . D . . . . . . . . . . . . . . . . . . . . . . . . . . . . . . . MT560941.1_China_Mosquito**

**2701 . . . . . . . . . . . . . . . . . . S . . . . . . . . . . . . . . . . . . . . . . . . . . . . . . . . . . . . . . . . . . . . . . . . . . . . . . . . . . . . . . . . . . . . . . . . . . . . . . . . . ON875960_India_Assam_Mosquito**

**N D T W K L E R A V F G E V K S C T W P E T H T L W G D G V E E S E L I I P H T I A G P R S K H N R R E G Y K T Q N Q G P W D E N G I V L D F D Y C P G T K V T I T E D C G K R G P S I R T T T D S G K Majority**

**------------------+-------------------+-------------------+-------------------+-------------------+-------------------+-------------------+-------------------+-------------------+-------------------+-**

**1010 1020 1030 1040 1050 1060 1070 1080 1090 1100**

**------------------+-------------------+-------------------+-------------------+-------------------+-------------------+-------------------+-------------------+-------------------+-------------------+-**

**3001 . . . . . . . . . . . . . . . . . . . . . . . . . . . . . . . . . . . . . . . . . . . . K . . . . . . . . . . . . . . . . . . . . . . . . . . . . . . . . . . . . . . . . . . . . . . V . . . . . . . . AF098735.1_Taiwan_Mosquito**

**3001 . . . . . . . . . . . . . . . . . . . . . . . . . . . . . . . . . . . . . . . . . . . . K . . . . . . . . . . . . . . . . . . . . . . . . . . . . . . . . . . . . . . . . . . . . . . . . . . . . . . . GQ902060.1_Thailand_Mosquito**

**3001 . . . . . . . . . . . . . . . . . . . . . . . . . . . . . . . . . . . . . . . . L . . . K . . . . . . . . . . . . . . . . . . . . . . . . . . . . . . . . . . . . . . . . . . . . . . V . . . . . . . . GQ902063.1_Thailand_Mosquito**

**3001 . . . . . . . . . . . . . . . . . . . . . . . . . . . . . . . . . . . . . . . . . . . . . . . . . . . . . . . . . . . . . . . . . . . . . . . . . . . . . . . . . . . . . . . . . . . . . . . . . . . . HQ652538.1_China_Mosquito**

**3001 . . . . . . . . . . . . . . . . . . . . . . . . . . . . . . . . . . . . . . . . . . . . . . . . . . . . . . . . . . . . . . . . . . . . . . . . . . . . . . . . . . . . . . . . . . . . . . . . . . . . JF499790.1_Taiwan_Mosquito**

**3001 . . . . . . . . . . . . . . . . . . . . . . . . . . . . D . . . . . . . . . . . . . . . K . . . . . . . . . . . . . . . . . . . . . . . . . . . . . . . . . . . . . . . . S . . . . . V . . . . . . . . JN864064.1_China_Mosquito**

**3001 . . . . . . . . . . . . . . . . . . . . . . . . . . . . . . . . . . . . . . . . . . . . . . . . . . . . . . . . . . . . . . . . . . . . . . . . . . . . . . . . . . . . . . . . . . . . . . . . . . . . JQ031753.1_Taiwan_Mosquito**

**3001 . . . . . . . . . . . . . . . . . . . . . . . . . . . . D . . . . . . . . . . . . . . . K . . . . . . . . . . . . . . . . . . . . . . . . . . . . . . . . . . . . . . . . S . . . . . V . . . . . . . . JQ086762.1_China_Mosquito**

**3001 . . . . . . . . . . . . . . . . . . . . . . . . . . . . . . . . . . . . . . . . . . . . . . . . . . . . . . . . . . . . . . . . . . . . . . . . . . . . . . . . . . . . . . . . . . . . . . . . . . . . KT229574.1_China_Mosquito**

**3001 . . . . . . . . . . . . . . . . . . . . . . . . . . . . . . . . . . . . . . . . . . . . . . . . . . . . . . . . . . . . . . . . . . . . . . . . . . . . . . . . . . . . . . . . . . . . . . . . . . . . KT229575.1_China_Mosquito**

**3001 . . . . . . . . . . . . . . . . . . . . . . . . . . . . . . . . . . . . . . . . . . . . . . . . . . . . . . . . . . . . . . . . . . . . . . . . . . . . . . . . . . . . . . . . . . . . . . . . . . . . LC461957.1_Japan_Mosquito**

**3001 . . . . . . . . . . . . . . . . . . . . . . . . . . . . . . . . . . . . . . . . . . . . . . . . . . . . . . . . . . . . . . . . . . . . . . . . . . . . . . . . . . . . . . . . . . . . . . . . . . . . LC513838.1_Japan_Mosquito**

**3001 . . . . . . . . . . . . . I . . . . . . . . . . . . . . . . . . . . . . . . . . . . . . K . . . . . . . . . . . . . . . . . . . D . . . . . . . . . . . . . . . . . . . . . . . . . . V . . . . . . . . LC579814.1_Indonesia_Mosquito**

**3001 . . . . . . . . . A . . . . . . . . . . . . . . . . . . . . . . . . . . . . . . . . . . . . . . . . . . . . . . . . . . . . . . . . . . . . . . . . . . . . . . . . . . . . . . . . . . . . . . . . . . LC623822.1_Japan_Mosquito**

**3001 . . . . . . . . . . . . . . . . . . . . . . . . . . . . . . . . . . . . . . . . . . . . . . . . . . . . . . . . . . . . . . . . . . . . . . . . . . . . . . . . . . . . . . . . . . . . . . . . . . . . MH385014.1_China_Mosquito**

**3001 . . . . . . . . . . . . . . . . . . . . . . . . . . . . . . . . . . . . . . . . . . . . . . . . . . . . . . . . . . . . . . . . . . . . . . . . . . . . . . . . . . . . . . . . . . . . . . . . . . . . MK558811.1_China_Mosquito**

**3001 . . . . . . . . . . . . . . . . . . . . . . . . . . . . . . . . . . . . . . . . . . . . . . . . . . . . . . . . . . . . . . . . . . . . . . . . . . . . . . . . . . . . . . . . . . . . . . . . . . . . MT254426.1_China_Mosquito**

**3001 . . . . . . . . . . . . . . . . . . . . . . . . . . . . . . . . . . . . . . . . . . . . . . . . . . . . . . . . . . . . . . . . . . . . . . . . . . . . . . . . . . . . . . . . . . . . . . . . . . . . MT560941.1_China_Mosquito**

**3001 . . . . . . . . . . . . . . . . . . . . . . . . . . . . . . . . . . . . . . . . . . . . . . . . . . . . . . . . . . . . . . . . . . . . . . . . . . . . . . . . . . . . . . . . . . . . . . . . . . . . ON875960_India_Assam_Mosquito**

**L I T D W C C R S C S L P P L R F R T E N G C W Y G M E I R P V R H D E T T L V R S Q V D A F N G E M I D P F Q L G L L V M F L A T Q E V L R K R W T A R L T I P A V L G A L L V L M L G G I T Y T D L Majority**

**------------------+-------------------+-------------------+-------------------+-------------------+-------------------+-------------------+-------------------+-------------------+-------------------+-**

**1110 1120 1130 1140 1150 1160 1170 1180 1190 1200**

**------------------+-------------------+-------------------+-------------------+-------------------+-------------------+-------------------+-------------------+-------------------+-------------------+-**

**3301 . . . . . . . . . . . . . . . G . . . . . . . . . . . . . . . . . . . . . . . . . . . . . . . . . G . V . . . . . . . . . . . . . . . . . . . . . . . . . . . . . . . . . . . . . . . . . . . . . . . . AF098735.1_Taiwan_Mosquito**

**3301 . . . . . . . . . . . . . . . . . . . . . . . . . . . . . . . . . . . . . . . . . . . . . . . . . . . . . . . . . . . . . . . . . . . . . . . . . . . . . . . . . . . . . . . . . . . . . . . . . . . . GQ902060.1_Thailand_Mosquito**

**3301 . . . . . . . . . . . . . . . . . . . . . . . . . . . . . . . . . . . . . . . . . . . . . . . . . . . V . . . . . . . . . . . . . . . . . . . . . . . . . . . . . . . . . . . . . . . . . . . . . . . . GQ902063.1_Thailand_Mosquito**

**3301 . . . . . . . . . . . . . . . . . . . . . . . . . . . . . . . . . . . . . . . . . . . . . . . . . . . . . . . . . . . . . . . . . . . . . . . . . . . . . . . . . . . . . . . . . . . . . . . . . . . . HQ652538.1_China_Mosquito**

**3301 . . . . . . . . . . . . . . . . . . . . . . . . . . . . . . . . . . . . . . . . . . . . . . . . . . . . . . . . . . . . . . . . . . . . . . . . . . . . . . . . . . . . . . . . . . . . . . . . . . . . JF499790.1_Taiwan_Mosquito**

**3301 . . . . . . . . . . . . . . . . . . . . . . . . . . . . . . . . M . . . . . . . . . . . H . . K . . . V . . . . . . . . . . . . . . . . . . . . . . . . . . . . . . . . . V . . . . . . . . . . . . . . JN864064.1_China_Mosquito**

**3301 . . . . . . . . . . . . . . . . . . . . . . . . . . . . . . . . . . . . . . . . . . . . . . . . . . . . . . . . . . . . . . . . . . . . . . . . . . . . . . . . . . . . . . . . . . . . . . . . . . . . JQ031753.1_Taiwan_Mosquito**

**3301 . . . . . . . . . . . . . . . . . . . . . . . . . . . . . . . . M . . . . . . . . . . . H . . K . . . V . . . . . . . . . . . . . . . . . . . . . . . . . . . . . . . . . V . . . . . . . . . . . . . . JQ086762.1_China_Mosquito**

**3301 . . . . . . . . . . . . . . . . . . . . . . . . . . . . . . . . . . . . . . . . . . . . . . . . . . . . . . . . . . . . . . . . . . . . . . . . . . . . . . . . . . . . . . . . . . . . . . . . . . . . KT229574.1_China_Mosquito**

**3301 . . . . . . . . . . . . . . . . . . . . . . . . . . . . . . . . . . . . . . . . . . . . . . . . . . . . . . . . . . . . . . . . . . . . . . . . . . . . . . . . . . . . . . . . . . . . . . . . . . . . KT229575.1_China_Mosquito**

**3301 . . . . . . . . . . . . . . . . . . . . . . . . . . . . . . . . . . . . . . . . . . . . . . . . . . . . . . . . . . . . . . . . . . . . . . . . . . . . . . . . . . . . . . . . . . . . . . . . . . . . LC461957.1_Japan_Mosquito**

**3301 . . . . . . . . . . . . . . . . . . . . . . . . . . . . . . . . . . . . . . . . . . . . . . . . . . . . . . . . . . . . . . . . . . . . . . . . . . . . . . . . . . . . . . . . . . . . . . . . . . . M LC513838.1_Japan_Mosquito**

**3301 . . . . . . . . . . T . . . . . . . . G S . . . . . . . V . . . K . . . A . . . . . . . . . . S . . . . . . . . . . . . . . . . . . . . . . . . . . . . . . . V . . . . . . . . . . . . . . . . . . . . LC579814.1_Indonesia_Mosquito**

**3301 . . . . . . . . . . . . . . . . . . . . . . . . . . . . . . . . . . . . . . . . . . . . . . . . . . . . . . . . . . . . . . . . . . . . . . . . . . . . . . . . . . . . . . . . . . . . . . . . . . . . LC623822.1_Japan_Mosquito**

**3301 . V . . . . . . . . . . . . . . . . . . . . . . . . . . . . . . . . . . . . . . . . . . . . . . . . . . . . . . . . . . . . . . . . . . . . . . . . . . . . . . . . . . . . . . . . . . . . . . . . . . MH385014.1_China_Mosquito**

**3301 . . . . . . . . . . . . . . . . . . . . . . . . . . . . . . . . . . . . . . . . . . . . . . . . . . . . . . . . . . . . . . . . . . . . . . . . . . . . . . . . . . . . . . . . . . . . . . . . . . . . MK558811.1_China_Mosquito**

**3301 . . . . . . . . . . . . . . . . . . . . . . . . . . . . . . . . . . . . . . . . . . . . . . . . . . . . . . . . . . . . . . . . . . . . . . . . . . . . . . . . . . . . . . . . . . . . . . . . . . . . MT254426.1_China_Mosquito**

**3301 . . . . . . . . . . . . . . . . . . . . . . . . . . . . . . . . . . . . . . . . . . . . . . . . . . . . . . . . . . . . . . . . . . . . . . . . . . . . . . . . . . . . . . . . . . . . . . . . . . . . MT560941.1_China_Mosquito**

**3301 . . . . . . . . . . . . . . . . . . . . . . . . . . . . . . . . . . . . . . . . . . . . . . . . . . . . . . . . . . . . . . . . . . . . . . . . . . . . . . . . . . . . . . . . . . . . . . . . . . . . ON875960_India_Assam_Mosquito**

**A R Y V V L V A A A F A E A N S G G D V L H L A L I A V F K I Q P A F L V M N M L S A R W T N Q E N V V L V L G A A F F Q L A S V D L Q I G V H G I L N A A A I A W M I V R A I T F P T T S T V A M P V Majority**

**------------------+-------------------+-------------------+-------------------+-------------------+-------------------+-------------------+-------------------+-------------------+-------------------+-**

**1210 1220 1230 1240 1250 1260 1270 1280 1290 1300**

**------------------+-------------------+-------------------+-------------------+-------------------+-------------------+-------------------+-------------------+-------------------+-------------------+-**

**3601 . . . . . . . . . . . . . . . . . . . . . . . . . . . . . . . . . . . . . . . . . . T . . . . . . . . . . . . . . . . . . . . . . . . . . . . . . . . . . . . M . . . . . . . . . . . . . . S . T . . . AF098735.1_Taiwan_Mosquito**

**3601 . . . . . . . . . . . . . . . . . . . . . . . . . . . . . . . . . . . . . . . . . . . . . . . . . . M . . . . . . . . . . . . . . . . . . . . . . . . . . . . . . . . . . . . . . . . . . . . . T . . . GQ902060.1_Thailand_Mosquito**

**3601 . . . . . . . . . . . . . . . . . . . . . . . . . . . . . . . . . . . . . . . . . . T . . . . . . . . . . . . . . . . . . . . . . . . . . . . . . . . . . . . . . . . . . . . . . . . . . . S . T . . . GQ902063.1_Thailand_Mosquito**

**3601 . . . . . . . . . . . . . . . . . . . . . . . . . . . . . . . . . . . . . . . . . . . . . . . . . . . . . . . . . . . . . . . . . . . . . . . . . . . . . . . . . . . . . . . . . . . . . . . . . . . . HQ652538.1_China_Mosquito**

**3601 . . . . . . . . . . . . . . . N . . . . . . . . . . . . . . . . . . . . . . . . . . . . . . . . . . . . . . . . . . . . . . . . . . . . . . . . . . . . . . . . . . . . . . . . . . . . . . . . . . . I JF499790.1_Taiwan_Mosquito**

**3601 . . . . . . . . . . . . . . . . . . . . . . . . . . . . . . . . . . . . . . . . . . T . . . . . . . . . . . . . . . . . . . . . . . . . . . . . . . . . . . . . . . . . . . . . . . . . . . S . T . . . JN864064.1_China_Mosquito**

**3601 . . . . . . . . . . . . . . . N . . . . . . . . . . . . . . . . . . . . . . . . . . . . . . . . . . . . . . . . . . . . . . . . . . . . . . . . . . . . . . . . . . . . . . . . . . . . . . . . . . . I JQ031753.1_Taiwan_Mosquito**

**3601 . . . . . . . . . . . . . . . . . . . . . . . . . . . . . . . . . . . . . . . . . . T . . . . . . . . . . . . . . . . . . . . . . . . . . . . . . . . . . . . . . . . . . . . . . . . . . . S . T . . . JQ086762.1_China_Mosquito**

**3601 . . . . . . . . . . . . . . . . . . . . . . . . . . . . . . . . . . . . . . . . . . . . . . . . . . . . . . . . . . . . . . . . . . . . . . . . . . . . . . . . . . . . . . . . . . . . . . . . . . . . KT229574.1_China_Mosquito**

**3601 . . . . . . . . . . . . . . . . . . . . . . . . . . . . . . . . . . . . . . . . . . . . . . . . . . . . . . . . . . . . . . . . . . . . . . . . . . . . . . . . . . . . . . . . . . . . . . . . . . . . KT229575.1_China_Mosquito**

**3601 . . . . . . . . . . . . . . . . . . . . . . . . . . . . . . . . . S . . . . . . . . . . . . . . . . . . . . . . . . . . . . . . . . . . . . . . . . . . . . . . . . . . . . . . . . . . . . . . . . . . LC461957.1_Japan_Mosquito**

**3601 . . . . . . . . . . . . . . . . . . . . . . . . . . . . . R . . . S . . . . . . . . . . . . . . . . . . . . . . . . . . . . . . . . . . . . . . . . . . . . . . . . . . . . . . . . . . . . . . . . . . LC513838.1_Japan_Mosquito**

**3601 . . . . . . . . . . . . . . . N . . . . . . . . . . . . . . . . . . . . . A . . . . T . . . . . . . . . . . . . . . . . . . . . A . . . . . . . . M . . . . . . . . . . I K . . . . . . . . . I T . . L LC579814.1_Indonesia_Mosquito**

**3601 . . . . . . . . . . . . . . . . . . . . . . . . . . . . . . . . . . . . . . . . . . . . . . . . . . . . . . . . . . . . . . . . . . . . . . . . . . . . . . . . . . . . . . . . . . . . . . . . V T . . LC623822.1_Japan_Mosquito**

**3601 . . . . . . . . . . . . . . . . . . . . . . . . . . . . . . . . . . . . . . . . . . . . . . . . . . . . . . . . . . . . . . . . . . . . . . . . . . . . . . . . . . . . . . . . . . . . . . . . . . . I MH385014.1_China_Mosquito**

**3601 . . . . . . . . . . . . . . . . . . . . . . . . . . . . . . . . . . . . . . . . . . . . . . . . . . . . . . . . . . . . . . . . . . . . . . . . . . . . . . . . . . . . . . . . . . . . . . . . . . . . MK558811.1_China_Mosquito**

**3601 . . . . . . . . . . . . . . . . . . . . . . . . . . . . . . . . . . . . . . . . . . . . . . . . . . . . . . . . . . . . . . . . . . . . . . . . . . . . . . . . . . . . . . . . . . . . . . . . . . . I MT254426.1_China_Mosquito**

**3601 . . . . . . . . . . . . . . . . . . . . . . . . . . . . . . . . . . . . . . . . . . . . . . . . . . . . . . . . . . . . . . . . . . . . . . . . . . . . . . . . . . . . . . . . . . . . . . . . . . . . MT560941.1_China_Mosquito**

**3601 . . . . . . . . . . . . . . . . . . . . . . . . . . . . . . . . . . . . . . . . . . . . . . . . . . . . . . . . . . . . . . . . . . . . . . . . . . . . . . . . . . . . . . . . . . . . . . . . . . . I ON875960_India_Assam_Mosquito**

**L A L L T P G M R A L Y L D T Y R I I L L V I G I C S L L Q E R R K T M A K K K G A V L L G L A L T S T G W F S P T T I A A G L M V C N P N K K R G W P A T E F L S A V G L M F A I V G G L A E L D I E Majority**

**------------------+-------------------+-------------------+-------------------+-------------------+-------------------+-------------------+-------------------+-------------------+-------------------+-**

**1310 1320 1330 1340 1350 1360 1370 1380 1390 1400**

**------------------+-------------------+-------------------+-------------------+-------------------+-------------------+-------------------+-------------------+-------------------+-------------------+-**

**3901 . . . . . . . . . . . . . . . . . . . . . . . . . . . . . . . . E . . . . . . . . . . . . . . . . . . . . . . . . . . . . . . . . . . . . . . . . . . . . . . . . . . . . . . . . . . . . . . . . . . . AF098735.1_Taiwan_Mosquito**

**3901 . . . . . . . . . . . . . . . . . . . . . . . . T . . . . . . . . . . . . . . . . . . . . . . . . . . . . . . . . . . . . . . . . . . . . . . . . . . . . . . . . . . . . . . . . . . . . . . . . . . . GQ902060.1_Thailand_Mosquito**

**3901 . . . . . . . . . . . . . . . . . . . . . . . . . . . . . . . . K . . . . . . . . . . . . . . . . . . . . . . . . . . . . . . . . . . . . . . . . . . . . . . . . . . . . . . . . . . . . . . . . . . . GQ902063.1_Thailand_Mosquito**

**3901 . . . . A . . . . . . . . . . . . . . . . . . . . . . . . . . . . . . . . . . . . . . . . . . . . . . . . . . . . . . . . . . . . . . . . . . . . . . . . . . . . . . . . . . . . . . . . . . . . . . . HQ652538.1_China_Mosquito**

**3901 . . . . . . . . . . . . . . . . . . . . . . . . . . . . . . . . . . . . . . . . . . . . . . . . . . . . . . . . . . . . . . . . . . . . . . . . . . . . . . . . . . . . . . . . . . . . . . . . . . . . JF499790.1_Taiwan_Mosquito**

**3901 . . . . . . . . . . . . . . . . . . . . . . . . . . . . . H . . K . . . . . . . . . . . . . . . . . . A . . . . . . . . . . . . . . . . . . . . . . . . . . . . . . . . . . . . . V . . . . . . . . . . JN864064.1_China_Mosquito**

**3901 . . . . . . . . . . . . . . . . . . . . . . . . . . . . . . . . . . . . . . . . . . . . . . . . . . . . . . . . . . . . . . . . . . . . . . . . . . . . . . . . . . . . . . . . . . . . . . . . . . . . JQ031753.1_Taiwan_Mosquito**

**3901 . . . . . . . . . . . . . . . . . . . . . . . . . . . . . H . . K . . . . . . . . . . . . . . . . . . A . . . . . . . . . . . . . . . . . . . . . . . . . . . . . . . . . . . . . V . . . . . . . . . . JQ086762.1_China_Mosquito**

**3901 . . . . . . . . . . . . . . . . . . . . . . . . . . . . . . . . . . . . . . . . . . . . . . . . . . . . . . . . . . . . . . . . . . . . . . . . . . . . . . . . . . . . . . . . . . . . . . . . . . . . KT229574.1_China_Mosquito**

**3901 . . . . . . . . . . . . . . . . . . . . . . . . . . . . . . . . . . . . . . . . . . . . . . . . . . . . . . . . . . . . . . . . . . . . . . . . . . . . . . . . . . . . . . . . . . . . . . . . . . . . KT229575.1_China_Mosquito**

**3901 . . . . . . . . . . . . . . . . . . . . . . . . . . . . . . . . . . . . . . . . . . . . . . . . . . . . . . . . . . . . . . . . . . . . . . . . . . . . . . . . . . . . . . . . . . . . . . . . . . . . LC461957.1_Japan_Mosquito**

**3901 . . . . . . . . . . . . . . . . . . . . . . . . . . . . . . . . . . . . . . . . . . . . . . . . . . . . . . . . . . . . . . . . . . . . . . . . . . . . . . . . . . . . . . . . . . . . . . . . . . . . LC513838.1_Japan_Mosquito**

**3901 . . . . V . . . . . . . . . . . . . . . . I . . V . . . . . . . . . . . . . . . . . . . . . . . . . . . . . . . . . . . . . . . . . . . . . . . . . . . . . . . . . . . . . . . . . . . . . . . . . . . LC579814.1_Indonesia_Mosquito**

**3901 . . . . . . . . . . . . . . . . . . . . . . . . . . . . . . . . . . . . . . . . . . . . . . . . . . . . . . . . . . . . . . . . . . . . . . . . . . . . . . . . . . . . . . . . . . . . . . . . . . . . LC623822.1_Japan_Mosquito**

**3901 . . . . . . . . . . . . . . . . . . . . . . . . . . . . . . . . . . . . . . . . . . . . . . . . . . . . . . . . . . . . . . . . . . . . . . . . . . . . . . . . . . . . . . . . . . . . . . . . . . . . MH385014.1_China_Mosquito**

**3901 . . . . . . . . . . . . . . . . . . . . . . . . . . . . . . . . . . . . . . . . . . . . . . . . . . . . . . . . . . . . . . . . . . . . . . . . . . . . . . . . . . . . . . . . . . . . . . . . . . . . MK558811.1_China_Mosquito**

**3901 . . . . . . . . . . . . . . . . . . . . . . . . . . . . . . . . . . . . . . . . . . . . . . . . . . . . . . . . . . . . . . . . . . . . . . . . . . . . . . . . . . . . . . . . . . . . . . . . . . . . MT254426.1_China_Mosquito**

**3901 . . . . . . . . . . . . . . . . . . . . . . . . . . . . . . . . . . . . . . . . . . . . . . . . . . . . . . . . . . . . . . . . . . . . . . . . . . . . . . . . . . . . . . . . . . . . . . . . . . . . MT560941.1_China_Mosquito**

**3901 . . . . . . . . . . . . . . . . . . . . . . . . . . . . . . . . . . . . . . . . . . . . . . . . . . . . . . . . . . . . . . . . . . . . . . . . . . . . . . . . . . . . . . . . . . . . . . . . . . . . ON875960_India_Assam_Mosquito**

**S M S I P F M L A G L M A V S Y V V S G K A T D M W L D R A A D I S W E M E A A I T G S S R R L D V K L D D D G D F H L I D D P G V P W K V W L L R M S C I G L A A L T P W A I V P A A F G Y W L T L K Majority**

**------------------+-------------------+-------------------+-------------------+-------------------+-------------------+-------------------+-------------------+-------------------+-------------------+-**

**1410 1420 1430 1440 1450 1460 1470 1480 1490 1500**

**------------------+-------------------+-------------------+-------------------+-------------------+-------------------+-------------------+-------------------+-------------------+-------------------+-**

**4201 . . . . . . . . . . . . . . . . . . . . . . . . . . . E . . . . . . . . . D . . . . . . . . . . . . . . . . . . . . . . . . . . . . . . . . . V . . . . . . . . . . . . . . . . . . . . . . . . . . . . AF098735.1_Taiwan_Mosquito**

**4201 . . . . . . . . . . . . . . . . . . . . . . . . . . . . . . . . . . . . . . . . . . . . . . . . . . . . . . . . . . . . . . . . . . . . . . . . . . . . . . . . . . . . . . . . . . . . . . . . . . . . GQ902060.1_Thailand_Mosquito**

**4201 . . . . . . . . . . . . . . . . . . . . . . . . . . . E . . . . . . . . . D . . . . . . . . . . . . . . . . . . . . . . . . . . . . . . . . . V . . . . . . . . . . . . . . . . . . . . . . . . . . . . GQ902063.1_Thailand_Mosquito**

**4201 . . . . . . . . . . . . . . . . . . . . . . . . . . . . . . . . . . . . . . . . . . . . . . . . . . . . . . . . . . . . . . . . . . . . . . . . . . . . . . . . . . . . . . . . . . . . L . . . . . . . HQ652538.1_China_Mosquito**

**4201 . . . . . . . . . . . . . . . . . . . . . . . . . . . . . . . . . . . . . . . . . . . . . . . . . . . . . . . . . . . . . . . . . . . . . . . . . . . . . . . . . . . . . . . . . . . . . . . . . . . . JF499790.1_Taiwan_Mosquito**

**4201 . . . . . . . . . . . . . . . . . . . . . . . . . . . E . . . . . . . D . G . . . . . . . . . . . . . . . . . . . . . . . . . . . . . . . . . V . . . . . . . . . . . . . . . . . . . . . . . . . . . . JN864064.1_China_Mosquito**

**4201 . . . . . . . . . . . . . . . . . . . . . . . . . . . . . . . . . . . . . . . . . . . . . . . . . . . . . . . . . . . . . . . . . . . . . . . . . . . . . . . . . . . . . . . . . . . . . . . . . . . . JQ031753.1_Taiwan_Mosquito**

**4201 . . . . . . . . . . . . . . . . . . . . . . . . . . . E . . . . . . . D . G . . . . . . . . . . . . . . . . . . . . . . . . . . . . . . . . . V . . . . . . . . . . . . . . . . . . . . . . . . . . . . JQ086762.1_China_Mosquito**

**4201 . . . . . . . . . . . . . . . . . . . . . . . . . . . . . . . . . . . . . . . . . . . . . . . . . . . . . . . . . . . . . . . . . . . . . . . . . . . . . . . . . . . . . . . . . . . . . . . . . . . . KT229574.1_China_Mosquito**

**4201 . . . . . . . . . . . . . . . . . . . . . . . . . . . . . . . . . . . . . . . . . . . . . . . . . . . . . . . . . . . . . . . . . . . . . . . . . . . . . . . . . . . . . . . . . . . . . . . . . . . . KT229575.1_China_Mosquito**

**4201 . . . . . . . . . . . . . . . . . . . . . . . . . . . . . . . . . . . . . . . . . . . . . . . . . . . . . . . . . . . . . . . . . . . . . . . . . . . . . . . . . . . . . . . . . . . . . . . . . . . . LC461957.1_Japan_Mosquito**

**4201 . . . . . . . . . . . . . . . . . . . . . . . . . . . . . . . . . . . . . . . . . . . . . . . . . . . . . . . . . . . . . . . . . . . . . . . . . . . . . . . . . . . . . . . . . . . . . . . . . . . . LC513838.1_Japan_Mosquito**

**4201 . . . . . . . . . . . . . . . . . . . . . . . . . . . E . . . . . . . . . D . . . . . . . . . . . . . . . . . . . . . . V . . . . . . . . . . V . . . . . . . . . . . . . . . . . . . . . . . . . . . . LC579814.1_Indonesia_Mosquito**

**4201 . . . . . . . . . . . . . . . . . . . . . . . . . . . . . . . . . . . . . . . . . . . . . . . . . . . . . . . . . . . . . . . . . . . . . . . . . . . . . . . . . . . . . . . . . . . . . . . . . . . . LC623822.1_Japan_Mosquito**

**4201 . . . . . . . . . . . . . . . . . . . . . . . . . . . . . . . . . . . . . . . . . . . . . . . . . . . . . . . . . . . . . . . . . . . . . . . . . . . . . . . . . . . . . . . . . . . . . . . . . . . . MH385014.1_China_Mosquito**

**4201 . . . . . . . . . . . . . . . . . . . . . . . . . . . . . . . . . . . . . . . . . . . . . . . . . . . . . . . . . . . . . . . . . . . . . . . . . . . . . . . . . . . . . . . . . . . . . . . . . . . . MK558811.1_China_Mosquito**

**4201 . . . . . . . . . . . . . . . . . . . . . . . . . . . . . . . . . . . . . . . . . . . . . . . . . . . . . . . . . . . . . . . . . . . . . . . . . . . . . . . . . . . . . . . . . . . . . . . . . . . . MT254426.1_China_Mosquito**

**4201 . . . . . . . . . . . . . . . . . . . . . . . . . . . . . . . . . . . . . . . . . . . . . . . . . . . . . . . . . . . . . . . . . . . . . . . . . . . . . . . . . . . . . . . . . . . . . . . . . . . . MT560941.1_China_Mosquito**

**4201 . . . . . . . . . . . . . . . . . . . . . . . . . . . . . . . . . . . . . . . . . . . . . . . . . . . . . . . . . . . . . . . . . . . . . . . . . . . . . . . . . . . . . . . . . . . . . . . . . . . . ON875960_India_Assam_Mosquito**

**T T K R G G V F W D T P S P K P C L K G D T T T G V Y R I M A R G I L G T Y Q A G V G V M Y E N V F H T L W H T T R G A A I M S G E G K L T P Y W G S V K E D R I S Y G G P W R F D R K W N G T D D V Q Majority**

**------------------+-------------------+-------------------+-------------------+-------------------+-------------------+-------------------+-------------------+-------------------+-------------------+-**

**1510 1520 1530 1540 1550 1560 1570 1580 1590 1600**

**------------------+-------------------+-------------------+-------------------+-------------------+-------------------+-------------------+-------------------+-------------------+-------------------+-**

**4501 . . . . . . . . . . . . . . . . . S . . . . . . . . . . . . . C . . . . . . . S . . . . I . . . . . . . . . . P . . . . . . . . . . . . . . . . . . . . . . . . . A . . . . . . . . . . . . . . . . . . AF098735.1_Taiwan_Mosquito**

**4501 . . . . . . . . . . . . . . . . . . . . . . . . . . . . . . . . . . . . . . . . . . . . . . . . . . . . . . . . . . . . . . . . . . . . . . . . . . . . . . . . . . . . . . . . . . . . . . . . . . . . GQ902060.1_Thailand_Mosquito**

**4501 . . . . . . . . . . . . . . . . . S . . . . . . . . . . . . . . . . . . . . . . . . . . . . . . . . . . . . . . . . . . . . . . . . . . . . . . . . . . . . . . . A . . . . . . . . . . . . . . . . . . GQ902063.1_Thailand_Mosquito**

**4501 . . . . . . . . . . . . . . . . . . . . . . . . . . . . . . . . . . . . . . . . . . . . . . . . . . . . . . . . . . . . . . . . . . . . . . . . . . . . . . . . . . . . . . . . . . . . . . . . . . . . HQ652538.1_China_Mosquito**

**4501 . . . . . . . . . . . . . . . . . . R . . . . . . . . . . . . . . . . . . . . . . . . . . . . . . . . . . . . . . . . . . . . . . . . . . . . . . . . . . . . . . . . . . . . . . . . . . . . . . . . . JF499790.1_Taiwan_Mosquito**

**4501 . . . . . . . . . . . . . . . . . S . . . . . . . . . . . . . . . . . . . . . . . . . . . . . . . . . . . . . . . . . . . . V . . . . . . . . . . . . . . . . . . A . . . . . . . . . . . . . . . . . . JN864064.1_China_Mosquito**

**4501 . . . . . . . . . . . . . . . . . . R . . . . . . . . . . . . . . . . . . . . . . . . . . . . . . . . . . . . . . . . . . . . . . . . . . . . . . . . . . . . . . . . . . . . . . . . . . . . . . . . . JQ031753.1_Taiwan_Mosquito**

**4501 . . . . . . . . . . . . . . . . . S . . . . . . . . . . . . . . . . . . . . . . . . . . . . . . . . . . . . . . . . . . . . V . . . . . . . . . . . . . . . . . . A . . . . . . . . . . . . . . . . . . JQ086762.1_China_Mosquito**

**4501 . . . . . . . . . . . . . . . . . . . . . . . . . . . . . . . . . . . . . . . . . . . . . . . . . . . . . . . . . . . . . . . . . . . . . . . . . . . . . . . . . . . . . . . . . . . . . . . . . . . . KT229574.1_China_Mosquito**

**4501 . . . . . . . . . . . . . . . . . . . . . . . . . . . . . . . . . . . . . . . . . . . . . . . . . . . . . . . . . . . . . . . . . . . . . . . . . . . . . . . . . . . . . . . . . . . . . . . . . . . . KT229575.1_China_Mosquito**

**4501 . . . . . . . . . . . . . . . . . . . . . . . . . . . . . . . . . . . . . . . . . . . . . . . . . . . . . . . . . . . . . . . . . . . . . . . . . . . . . . . . . . . . . . . . . . . . . . . . . . . . LC461957.1_Japan_Mosquito**

**4501 . . . . . . . . . . . . . . . . . . . . . . . . . . . . . . . . . . . . . . . . . . . . . . . . . . . . . . . . . . . . . . . . . . . . . . . . . . . . . . . . . . . . . . . . . . . . . . . . . . . . LC513838.1_Japan_Mosquito**

**4501 . . . . . . . . . . . . . . . . . T . . . . . . . . . . . . . . . . . . . . . . . . . . . . . G . . . . . . . . . . . . . . . . . . . . . . . . . . . . . . . . . A . . . . . . . . . . . . . K . . . . LC579814.1_Indonesia_Mosquito**

**4501 . . . . . . . . . . . . . . . . . . . . . . . . . . . . . . . . . . . . . . . . . . . . . . . . . . . . . . . . . . . . . . . . . . . . . . . . . . . . . . . . . . . . . . . . . . . . . . . . . . . . LC623822.1_Japan_Mosquito**

**4501 . . . . . . . . . . . . . . . . . . . . . . . . . . . . . . . . . . . . . . . . . . . . . . . . . . . . . . . . . . . . . . . . . . . . . . . . . . . . . . . . . . . . . . . . . . . . . . . . . . . . MH385014.1_China_Mosquito**

**4501 . . . . . . . . . . . . . . . . . . . . . . . . . . . . . . . . . . . . . . . . . . . . . . . . . . . . . . . . . . . . . . . . . . . . . . . . . . . . . . . . . . . . . . . . . . . . . . . . . . . . MK558811.1_China_Mosquito**

**4501 . . . . . . . . . . . . . . . . . . . . . . . . . . . . . . . . . . . . . . . . . . . . . . . . . . . . . . . . . . . . . . . . . . . . . . . . . . . . . . . . . . . . . . . . . . . . . . . . . . . . MT254426.1_China_Mosquito**

**4501 . . . . . . . . . . . . . . . . . . . . . . . . . . . . . . . . . . . . . . . . . . . . . . . . . . . . . . . . . . . . . . . . . . . . . . . . . . . . . . . . . . . . . . . . . . . . . . . . . . . . MT560941.1_China_Mosquito**

**4501 . . . . . . . . . . . . . . . . . . . . . . . . . . . . . . . . . . . . . . . . . . . . . . . . . . . . . . . . . . . . . . . . . . . . . . . . . . . . . . . . . . . . . . . . . . . . . . . . . . . . ON875960_India_Assam_Mosquito**

**V I V V E P G K P A V N I Q T K P G V F R T P F G E V G A V S L D Y P R G T S G S P I L D S N G D I I G L Y G N G V E L G D G S Y V S A I V Q G D R Q E E P V P D A Y T P S M L K K R Q M T V L D L H P Majority**

**------------------+-------------------+-------------------+-------------------+-------------------+-------------------+-------------------+-------------------+-------------------+-------------------+-**

**1610 1620 1630 1640 1650 1660 1670 1680 1690 1700**

**------------------+-------------------+-------------------+-------------------+-------------------+-------------------+-------------------+-------------------+-------------------+-------------------+-**

**4801 . . . . . . . . A . . . . . . . . . . . . . . . . . . . . . . . . . . . . . . . . . . . . . . . . . . . . . . . . . . . . . . . . . . . . . . . . . . . . . . . E . . . . N . . R . . . . . . . . . . . AF098735.1_Taiwan_Mosquito**

**4801 . . . . . . . . . . . . . . . . . . . . . . . . . . I . . . . . . . . . . . . . . . . . . . . . . . . . . . . . . . . . . . . . . . . . . . . . . . . . . . . . . . . . . . . . . . . . . . . . . . . . GQ902060.1_Thailand_Mosquito**

**4801 . . . . . . . . A . . . . . . . . . . . . . . . . . . . . . . . . . . . . . . . . . . . . . . . . . . . . . . . . . . . . . . . . . . . . . . . E . . . . . . . E . . . . N . . R . . . L . . . . . . . GQ902063.1_Thailand_Mosquito**

**4801 . . . . . . . . . . . . . . . . . . . . . . . . . . . . . . . . . . . . . . . . . . . . . . . . . . . . . . . . . . . . . . . . . . . . . . . . . . . . . . . . . . . . . . . . . . . . . . . . . . . . HQ652538.1_China_Mosquito**

**4801 . . . . . . . . . . . . . . . . . . . . . . . . . . . . . . . . . . . . . . . . . . . . . . . . . . . . . . . . . . . . . . . . . . . . . . . . . . . . . . . . . . . . . . . . . . . R . . . . . . . . JF499790.1_Taiwan_Mosquito**

**4801 . . . . . . . . G . . . . . . . . . . . . . . . . . . . . . . . . . . . . . . . . . . . . . . . . . . . . . . . . . . . . . . . . . . . . . . . . . . . . . . . E . . . . N . . R . . . . . . . . . . . JN864064.1_China_Mosquito**

**4801 . . . . . . . . . . . . . . . . . . . . . . . . . . . . . . . . . . . . . . . . . . . . . . . . . . . . . . . . . . . . . . . . . . . . . . . . . . . . . . . . . . . . . . . . . . . . . . . . . . . . JQ031753.1_Taiwan_Mosquito**

**4801 . . . . . . . . G . . . . . . . . . . . . . . . . . . . . . . . . . . . . . . . . . . . . . . . . . . . . . . . . . . . . . . . . . . . . . . . . . . . . . . . E . . . . N . . R . . . . . . . . . . . JQ086762.1_China_Mosquito**

**4801 . . . . . . . . . . . . . . . . . . . . . . . . . . . . . . . . . . . . . . . . . . . . . . . . . . . . . . . . . . . . . . . . . . . . . . . . . . . . . . . . . . . . S . . . . . . . . . . . . . . . KT229574.1_China_Mosquito**

**4801 . . . . . . . . . . . . . . . . . . . . . . . . . . . . . . . . . . . . . . . . . . . . . . . . . . . . . . . . . . . . . . . . . . . . . . . . . . . . . . . . . . . . S . . . . . . . . . . . . . . . KT229575.1_China_Mosquito**

**4801 . . . . . . . . . . . . . . . . . . . . . . . . . . I . . . . . . . . . . . . . . . . . . . . . . . . . . . . . . . . . . . . . . . . . . . . . . . . . . . . . . . . . . . . . . . . . . . . . . . . . LC461957.1_Japan_Mosquito**

**4801 . . . . . . . . . . . . . . . . . . . . . . . . . . I . . . . . . . . . . . . . . . . . . . . . . . . . . . . . . . . . . . . . . . . . . . . . . . . . . . . . . . . . . . . . . . . . . . . . . . . . LC513838.1_Japan_Mosquito**

**4801 . . . . . . . . A . I . . . . . . . . . C . . . . . . . . . . . . . . . . . . . . . . . . . . . . . . . . . . . . . . . . . . . . . . . . . . . E . . . . . I . E . . N . . . . . . . . . . . . . . . . LC579814.1_Indonesia_Mosquito**

**4801 . . . . . . . . . . . . . . . . . . . . . . . . . . I . . . . . . . . . . . . . . . . . . . . . . . . . . . . . . . . . . . . . . . . . . . . . . . . . . . . . . . . . . . . . . . . . . . . . . . . . LC623822.1_Japan_Mosquito**

**4801 . . . . . . . . . . . . . . . . . . . . . . . . . . . . . . . . . . . . . . . . . . . . . . . . . . . . . . . . . . . . . . . . . . . . . . . . . . . . . . . . . . . . . . . . . . . . . . . . . . . . MH385014.1_China_Mosquito**

**4801 . . . . . . . . . . . . . . . . . . . . . . . . . . I . . . . . . . . . . . . . . . . . . . . . . . . . . . . . . . . . . . . . . . . . . . . . . . . . . . . . . . . . . . . . . . . . . . . . . . . . MK558811.1_China_Mosquito**

**4801 . . . . . . . . . . . . . . . . . . . . . . . . . . . . . . . . . . . . . . . . . . . . . . . . . . . . . . . . . . . . . . . . . . . . . . . . . . . . . . . . . . . . . . . . . . . . . . . . . . . . MT254426.1_China_Mosquito**

**4801 . . . . . . . . . . . . . . . . . . . . . . . . . . I . . . . . . . . . . . . . . . . . . . . . . . . . . . . . . . . . . . . . . . . . . . . . . . . . . . . . . . . . . . . . . . . . . . . . . . . . MT560941.1_China_Mosquito**

**4801 . . . . . . . . . . . . . . . . . . . . . . . . . . . . . . . . . . . . . . . . . . . . . . . . . V . . . . . . . . . . . . . . . . . . . . . . . . . . . . . . . . . . . . . . . . . . . . . . . . . . ON875960_India_Assam_Mosquito**

**G S G K T R K I L P Q I I K D A I Q Q R L R T A V L A P T R V V A A E M A E A L R G L P V R Y Q T S A V Q R E H Q G N E I V D V M C H A T L T H R L M S P N R V P N Y N L F V M D E A H F T D P A S I A Majority**

**------------------+-------------------+-------------------+-------------------+-------------------+-------------------+-------------------+-------------------+-------------------+-------------------+-**

**1710 1720 1730 1740 1750 1760 1770 1780 1790 1800**

**------------------+-------------------+-------------------+-------------------+-------------------+-------------------+-------------------+-------------------+-------------------+-------------------+-**

**5101 . . . . . . . . . . . . . . . . . . . . . . . . . . . . . . . . . . . . . . . . . . . . . . . . . . . . . . . . . . . . . . . . . . . . . . . . . . . . . . . . . . . . . . . . . . . . . . . . . . . . AF098735.1_Taiwan_Mosquito**

**5101 . . . . . . . . . . . . . . . . . . . . . . . . . . . . . . . . . . . . . . . . . . . . . . . . . . . . . . . . . . . . . . . . . . . . . . . . . . . . . . . . . . . . . . . . . . . . . . . . . . . . GQ902060.1_Thailand_Mosquito**

**5101 . . . . . . . . . . . . . . . . . . . . . . . . . . . . . . . . . . . . . . . . . . . . . . . . . . . . . . . . . . . . . . . . . . . . . . . . . . . . . . . . . . . . . . . . . . . . . . . . . . . . GQ902063.1_Thailand_Mosquito**

**5101 . . . . . . . . . . . . . . . . . . . . . . . . . . . . . . . . . . . . . . . . . . . . . . . . . . . . . . . . . . . . . . . . . . . . . . . . . . . . . . . . . . . . . . . . . . . . . . . . . . . . HQ652538.1_China_Mosquito**

**5101 . . . . . . . . . . . . . . . . . . . . . . . . . . . . . . . . . . . . . . . . . . . . . . . . . . . . . . . . . . . . . . . . . . . . . . . . . . . . . . . . . . . . . . . . . . . . . . . . . . . . JF499790.1_Taiwan_Mosquito**

**5101 . . . . . . . . . . . . . . . . . . . . . . . . . . . . . . . . . . . . . . V . . . . . . . . . . . . . . . . . . . . . . . . . . . . . . . . . . . . . . . . . . . . . . . . . . . . . . . . . . . . . JN864064.1_China_Mosquito**

**5101 . . . . . . . . . . . . . . . . . . . . . . . . . . . . . . . . . . . . . . . . . . . . . . . . . . . . . . . . . . . . . . . . . . . . . . . . . . . . . . . . . . . . . . . . . . . . . . . . . . . . JQ031753.1_Taiwan_Mosquito**

**5101 . . . . . . . . . . . . . . . . . . . . . . . . . . . . . . . . . . . . . . V . . . . . . . . . . . . . . . . . . . . . . . . . . . . . . . . . . . . . . . . . . . . . . . . . . . . . . . . . . . . . JQ086762.1_China_Mosquito**

**5101 . . . . . . . . . . . . . . . . . . . . . . . . . . . . . . . . . . . . . . . . . . . . . . . . . . . . . . . . . . . . . . . . . . . . . . . . . . . . . . . . . . . . . . . . . . . . . . . . . . . . KT229574.1_China_Mosquito**

**5101 . . . . . . . . . . . . . . . . . . . . . . . . . . . . . . . . . . . . . . . . . . . . . . . . . . . . . . . . . . . . . . . . . . . . . . . . . . . . . . . . . . . . . . . . . . . . . . . . . . . . KT229575.1_China_Mosquito**

**5101 . . . . . . . . . . . . . . . . . . . . . . . . . . . . . . . . . . . . . . . . K . . . . . . . . . . . . . . . . . . . . . . . . . . . . . . . . . . . . . . . . . . . . . . . . . . . . . . . . . . . LC461957.1_Japan_Mosquito**

**5101 . . . . . . . . . . . . . . . . . . . . . . . . . . . . . . . . . . . . . . . . K . . . . . . . . . . . . . . . . . . . . . . . . . . . . . . . . . . . . . . . . . . . . . . . . . . . . . . . . . . . LC513838.1_Japan_Mosquito**

**5101 . . . . . . . . . . . . . R . . . . . . . . . . . . . . . . . . . . . . . . . . . . . . . . . . . . . . . . . . . . . . . . . . . . . . . . . . . . . . . . . . . . . . . . . . . . . . . . . . . . . . LC579814.1_Indonesia_Mosquito**

**5101 . . . . . . . . . . . . . . . . . . . . . . . . . . . . . . . . . . . . . . . . K . . . . . . . . . . . . . . . . . . . . . . L . . . . . . . . . . . . . . . . . . . . . . . . . . . . . . . . . . . . LC623822.1_Japan_Mosquito**

**5101 . . . . . . . . . . . . . . . . . . . . . . . . . . . . . . . . . . . . . . . . . . . . . . . . . . . . . . . . . . . . . . . . . . . . . . . . . . . . . . . . . . . . . . . . . . . . . . . . . . . . MH385014.1_China_Mosquito**

**5101 . . . . . . . . . . . . . . . . . . . . . . . . . . . . . . . . . . . . . . . . K . . . . . . . . . . . . . . . . . . . . . . . . . . . . . . . . . . . . . . . . . . . . . . . . . . . . . . . . . . . MK558811.1_China_Mosquito**

**5101 . . . . . . . . . . . . . . . . . . . . . . . . . . . . . . . . . . . . . . . . . . . . . . . . . . . . . . . . . . . . . . . . . . . . . . . . . . . . . . . . . . . . . . . . . . . . . . . . . . . . MT254426.1_China_Mosquito**

**5101 . . . . . . . . . . . . . . . . . . . . . . . . . . . . . . . . . . . . . . . . K . . . . . . . . . . . . . . . . . . . . . . . . . . . . . . . . . . . . . . . . . . . . . . . . . . . . . . . . . . . MT560941.1_China_Mosquito**

**5101 . . . . . . . . . . . . . . . . . . . . . . . . . . . . . . . . . . . . . . . . . . . . . . . . . . . . . . . . . . . . . . . . . . . . . . . . . . . . . . . . . . . . . . . . . . . . . . . . . . . . ON875960_India_Assam_Mosquito**

**A R G Y I A T K V E L G E A A A I F M T A T P P G T T D P F P D S N A P I H D L Q D E I P D R A W S S G Y E W I T D Y A G K T V W F V A S V K M G N E I A M C L Q R A G K K V I Q L N R K S Y D T E Y P Majority**

**------------------+-------------------+-------------------+-------------------+-------------------+-------------------+-------------------+-------------------+-------------------+-------------------+-**

**1810 1820 1830 1840 1850 1860 1870 1880 1890 1900**

**------------------+-------------------+-------------------+-------------------+-------------------+-------------------+-------------------+-------------------+-------------------+-------------------+-**

**5401 . . . . . . . . . . . . . . . . . . . . . . . . . . . . . . . . . . . . . . . . . . . . . . . . . . . . . . . . . E . . . . . . . . . . . . . . . . . . . . . . . . . . . . . . . . . . . . . . . . . . AF098735.1_Taiwan_Mosquito**

**5401 . . . . . . . . . . . . . . . . . . . . . . . . . . . . . . . . . . . . . . . . . . . . . . . . . . . . . . . . . E . . . . . . . . . . . . . . . . . . . . . . . . . . . . . . . . . . . . . . . . . . GQ902060.1_Thailand_Mosquito**

**5401 . . . . . . . . . . . . . . . . . . . . . . . . . . . . . . . . . . . . . . . . . . . . . . . . . . . . . . . . . E . . . . . . . . . . . . . . . . . . . . . . . . . . . . . . . . . . . . . . . . . . GQ902063.1_Thailand_Mosquito**

**5401 . . . . . . . . . . . . . . . . . . . . . . . . . . . . . . . . . . . . . . . . . . . . . . . . . . . . . . . . . . . . . . . . . . . . . . . . . . . . . . . . . . . . . . . . . . . . . . . . . . . . HQ652538.1_China_Mosquito**

**5401 . . . . . . . . . . . . . . . . . . . . . . . . . . . . . . . . . . . . . . . . . . . . . . . . . . . . . . . . . . . . . . . . . . . . . . . . . . . . . . . . . . . . . . . . . . . . . . . . . . . . JF499790.1_Taiwan_Mosquito**

**5401 . . . . . . . . . . . . . . . . . . . . . . . . . . . . . . . . . . . . . . . . . . . . . . . . . . . . . . . . . E . . . . . . . . . . . . . . . . . . . . . . . . . . . . . . . . . . . . . . . . . . JN864064.1_China_Mosquito**

**5401 . . . . . . . . . . . . . . . . . . . . . . . . . . . . . . . . . . . . . . . . . . . . . . . . . . . . . . . . . . . . . . . . . . . . . . . . . . . . . . . . . . . . . . . . . . . . . . . . . . . . JQ031753.1_Taiwan_Mosquito**

**5401 . . . . . . . . . . . . . . . . . . . . . . . . . . . . . . . . . . . . . . . . . . . . . . . . . . . . . . . . . E . . . . . . . . . . . . . . . . . . . . . . . . . . . . . . . . . . . . . . . . . . JQ086762.1_China_Mosquito**

**5401 . . . . . . . . . . . . . . . . . . . . . . . . . . . . . . . . . . . . . . . . . . . . . . . . . . . . . . . . . . . . . . . . . . . . . . . . . . . . . . . . . . . . . . . . . . . . . . . . . . . . KT229574.1_China_Mosquito**

**5401 . . . . . . . . . . . . . . . . . . . . . . . . . . . . . . . . . . . . . . . . . . . . . . . . . . . . . . . . . . . . . . . . . . . . . . . . . . . . . . . . . . . . . . . . . . . . . . . . . . . . KT229575.1_China_Mosquito**

**5401 . . . . . . . . . . . . . . . . . . . . . . . . . . . . . . . . . . . L . . . . . . . . . . . . . . . . . . . . . . . . . . . . . . . . . . . . . . . . . . . . . . . . . . . . . . . . . . . . . . . . LC461957.1_Japan_Mosquito**

**5401 . . . . . . . . . . . . . . . . . . . . . . . . . . . . . . . . . . . . . . . . . . . . . . . . . . . . . . . . . . . . . . . . . . . . . . . . . . . . . . . . . . . . . . . . . . . . . . . . . . . . LC513838.1_Japan_Mosquito**

**5401 . . . . . . . . . . . . . . . . . . . . . . . . . . . . . . . . . . . . . . . . . . . . . . . . . . . . . . . . . E . . . . . . . . . . . . . . . . . . . V . . . . . . . R . . . . . . . . . . . . . . LC579814.1_Indonesia_Mosquito**

**5401 . . . . . . . . . . . . . . . . . . . . . . . . . . . . . . . . . . . . . . . . . . . . . . . . . . . . . . . . . . . . . . . . . . . . . . . . . . . . . . . . . . . . . . . . . . . . . . . . . . . . LC623822.1_Japan_Mosquito**

**5401 . . . . . . . . . . . . . . . . . . . . . . . . . . . . . . . . . . . . . . . . . . . . . . . . . . . . . . . . . . . . . . . . . . . . . . . . . . . . . . . . . . . . . . . . . . . . . . . . . . . . MH385014.1_China_Mosquito**

**5401 . . . . . . . . . . . . . . . . . . . . . . . . . . . . . . . . . . . . . . . . . . . . . . . . . . . . . . . . . . . . . . . . . . . . . . . . . . . . . . . . . . . . . . . . . . . . . . . . . . . . MK558811.1_China_Mosquito**

**5401 . . . . . . . . . . . . . . . . . . . . . . . . . . . . . . . . . . . . . . . . . . . . . . . . . . . . . . . . . . . . . . . . . . . . . . . . . . . . . . . . . . . . . . . . . . . . . . . . . . . . MT254426.1_China_Mosquito**

**5401 . . . . . . . . . . . . . . . . . . . . . . . . . . . . . . . . . . . . . . . . . . . . . . . . . . . . . . . . . . . . . . . . . . . . . . . . . . . . . . . . . . . . . . . . . . . . . . . . . . . . MT560941.1_China_Mosquito**

**5401 . . . . . . . . . . . . . . . . . . . . . . . . . . . . . . . . . . . . . . . . . . . . . . . . . . . . . . . . . . . . . . . . . . . . . . . . . . . . . . . . . . . . . . . . . . . . . . . . . . . . ON875960_India_Assam_Mosquito**

**K C K N G D W D F V I T T D I S E M G A N F G A S R V I D C R K S V K P T I L E E G E G R V I L G N P S P I T S A S A A Q R R G R V G R N P N Q V G D E Y H Y G G A T S E D D S N L A H W T E A K I M L Majority**

**------------------+-------------------+-------------------+-------------------+-------------------+-------------------+-------------------+-------------------+-------------------+-------------------+-**

**1910 1920 1930 1940 1950 1960 1970 1980 1990 2000**

**------------------+-------------------+-------------------+-------------------+-------------------+-------------------+-------------------+-------------------+-------------------+-------------------+-**

**5701 . . . . . . . . . . . . . . . . . . . . . . . . . . . . . . . . . . . . . . . . . . . . . . . . . . . . . . . . . . . . . . . . . . . . . . . . . . . . . . . . . P . . . . . . . . . . . . . . . . . . AF098735.1_Taiwan_Mosquito**

**5701 . . . . . . . . . . . . . . . . . . . . . . . . . . . . . . . . . . . . . . . . . . . . . . . . . . . . . . . . . . . . . . . . . . . . . . . . . . . . . . . . . . . . . . . . . . . . . . . . . . . . GQ902060.1_Thailand_Mosquito**

**5701 . . . . . . . . . . . . . . . . . . . . . . . . . . . . . . . . . . . . . . . . . . . . . . . . . . . . . . . . . . . . . . . . . . . . . . . . . . . . . . . . . . . . . . . . . . . . . . . . . . . . GQ902063.1_Thailand_Mosquito**

**5701 . . . . . . . . . . . . . . . . . . . . . . . . . . . . . . . . . . . . . . . . . . . . . . . . . . . . . . . . . . . . . . . . . . . . . . . . . . . . . . . . . . . . . . . . . . . . . . . . . . . . HQ652538.1_China_Mosquito**

**5701 . . . . . . . . . . . . . . . . . . . . . . . . . . . . . . . . . . . . . . . . . . . . . . . . . . . . . . . . . . . . . . . . . . . . . . . . . . . . . . . . . . . . . . . . . . . . . . . . . . . . JF499790.1_Taiwan_Mosquito**

**5701 . . . . . . . . . . . . . . . . . . . . . . . . . . . . . . . . . . . . . . . . . . . . . . . . . . . . . . . . . . . . . . . . . . . . . . . . . . . . . . . . . . . . . . . . . . . . . . . . . . . . JN864064.1_China_Mosquito**

**5701 . . . . . . . . . . . . . . . . . . . . . . . . . . . . . . . . . . . . . . . . . . . . . . . . . . . . . . . . . . . . . . . . . . . . . . . . . . . . . . . . . . . . . . . . . . . . . . . . . . . . JQ031753.1_Taiwan_Mosquito**

**5701 . . . . . . . . . . . . . . . . . . . . . . . . . . . . . . . . . . . . . . . . . . . . . . . . . . . . . . . . . . . . . . . . . . . . . . . . . . . . . . . . . . . . . . . . . . . . . . . . . . . . JQ086762.1_China_Mosquito**

**5701 . . . . . . . . . . . . . . . . . . . . . . . . . . . . . . . . . . . . . . . . . . . . . . . . . . . . . . . . . . . . . . . . . . . . . . . . . . . . . . . . . . . . . . . . . . . . . . . . . . . . KT229574.1_China_Mosquito**

**5701 . . . . . . . . . . . . . . . . . . . . . . . . . . . . . . . . . . . . . . . . . . . . . . . . . . . . . . . . . . . . . . . . . . . . . . . . . . . . . . . . . . . . . . . . . . . . . . . . . . . . KT229575.1_China_Mosquito**

**5701 . . . . . . . . . . . . . . . . . . . . . . . . . . . . . . . . . . . . . . . . . . . . . . . . . . . . . . . . . . . . . . . . . . . . . . . . . . . . . . . . . . . . . . . . . . . . . . . . . . . . LC461957.1_Japan_Mosquito**

**5701 . . . . . . . . . . . . . . . . . . . . . . . . . . . . . . . . . . . . . . . . . . . . . . . . . . . . . . . . . . . . . . . . . . . . . . . . . . . . . . . . . . . . . . . . . . . . . . . . . . . . LC513838.1_Japan_Mosquito**

**5701 . . . . . . . . . . . . . . . . . . . . . . . . . . . . . . . . . . . . . . . . . . . . . . . . . . . . . . . . . . . . . . . . . . . . . . . . . . . . . . . . . . . . . . . . . . . . . . . . . . L . LC579814.1_Indonesia_Mosquito**

**5701 . . . . . . . . . . . . . . . . . . . . . . . . . . . . . . . . . . . . . . . . . . . . . . . . . . . . . . . . . . . . . . . . . . . . . . . . . . . . . . . . . . . . . . . . . . . . . . . . . . . . LC623822.1_Japan_Mosquito**

**5701 . . . . . . . . . . . . . . . . . . . . . . . . . . . . . . . . . . . . . . . . . . . . . . . . . . . . . . . . . . . . . . . . . . . . . . . . . . . . . . . . . . . . . . . . . . . . . . . . . . . . MH385014.1_China_Mosquito**

**5701 . . . . . . . . . . . . . . . . . . . . . . . . . . . . . . . . . . . . . . . . . . . . . . . . . . . . . . . . . . . . . . . . . . . . . . . . . . . . . . . . . . . . . . . . . . . . . . . . . . . . MK558811.1_China_Mosquito**

**5701 . . . . . . . . . . . . . . . . . . . . . . . . . . . . . . . . . . . . . . . . . . . . . . . . . . . . . . . . . . . . . . . . . . . . . . . . . . . . . . . . . . . . . . . . . . . . . . . . . . . . MT254426.1_China_Mosquito**

**5701 . . . . . . . . . . . . . . . . . . . . . . . . . . . . . . . . . . . . . . . . . . . . . . . . . . . . . . . . . . . . . . . . . . . . . . . . . . . . . . . . . . . . . . . . S . . . . . . . . . . . MT560941.1_China_Mosquito**

**5701 . . . . . . . . . . . . . . . . . . . . . . . . . . . . . . . . . . . . . . . . . . . . . . . . . . . . . . . . . . . . . . . . . . . . . . . . . . . . . . . . . . . . . . . . . . . . . . . . . . . . ON875960_India_Assam_Mosquito**

**D N I H M P N G L V A Q L Y G P E R E K A F T M D G E Y R L R G E E K K N F L E L L R T A D L P V W L A Y K V A S N G I Q Y T D R K W C F D G P R T N A I L E D N T E V E I V T R M G E R K I L K P R W Majority**

**------------------+-------------------+-------------------+-------------------+-------------------+-------------------+-------------------+-------------------+-------------------+-------------------+-**

**2010 2020 2030 2040 2050 2060 2070 2080 2090 2100**

**------------------+-------------------+-------------------+-------------------+-------------------+-------------------+-------------------+-------------------+-------------------+-------------------+-**

**6001 . . . . . . . . . . . . . . . . . . . . . . . . . . . . . . . . . . . . . . . . . . . . . . . . . . . . . R . . . . . . . . . . . . . . . . . . . . . . . . . . . . . . . . . . . . . . . . . . . . . . AF098735.1_Taiwan_Mosquito**

**6001 . . . . . . . . . . . . . . . . . . . . . . . . . . . . . . . . . . . . . . . . . . . . . . . . . . . . . . . . . . . . . . . . . . . . . . . . . . . . . . . . . . . . . . . . . . . . . . . . . . . . GQ902060.1_Thailand_Mosquito**

**6001 . . . . . . . . . . . . . . . . . . . . . . . . . . . . . . . . . . . . . . . . . . . . . . . . . . . . . . . . . . . . . . . . . . . . . . . . . . . . . . . . . . . . . . . . . . . . . . . . . . . . GQ902063.1_Thailand_Mosquito**

**6001 . . . . . . . . . . . . . . . . . . . . . . . . . . . . . . . . . . . . . . . . . . . . . . . . . . . . . . . . . . . F . . . . . . . . . . . . . . . . . . . . . . . . . . . . . . . . . . . . . . . . HQ652538.1_China_Mosquito**

**6001 . . . . . . . . . . . . . . . . . . . . . . . . . . . . . . . . . . . . . . . . . . . . . . . . . . . . . . . . . . . . . . . . . . . . . . . . . . . . . . . . . . . . . . . . . . . . . . . . . . . . JF499790.1_Taiwan_Mosquito**

**6001 . . . . . . . . . . . . . . . . . . . . . . . . . . . . . . . . . . . . . . . . . . . . . . . . . . . . . . . . . . . . . . . . . . . . . . . . . . . . . . . . . . . . . . . . . . . . . . . . . . . . JN864064.1_China_Mosquito**

**6001 . . . . . . . . . . . . . . . . . . . . . . . . . . . . . . . . . . . . . . . . . . . . . . . . . . . . . . . . . . . . . . . . . . . . . . . . . . . . . . . . . . . . . . . . . . . . . . . . . . . . JQ031753.1_Taiwan_Mosquito**

**6001 . . . . . . . . . . . . . . . . . . . . . . . . . . . . . . . . . . . . . . . . . . . . . . . . . . . . . . . . . . . . . . . . . . . . . . . . . . . . . . . . . . . . . . . . . . . . . . . . . . . . JQ086762.1_China_Mosquito**

**6001 . . . . . . . . . . . . . . . . . . . . . . . . . . . . . . . . . . . . . . . . . . . . . . . . . . . . . . . . . . . . . . . . . . . . . . . . . . . . . . . . . . . . . . . . . . . . . . . . . . . . KT229574.1_China_Mosquito**

**6001 . . . . . . . . . . . . . . . . . . . . . . . . . . . . . . . . . . . . . . . . . . . . . . . . . . . . . . . . . . . . . . . . . . . . . . . . . . . . . . . . . . . . . . . . . . . . . . . . . . . . KT229575.1_China_Mosquito**

**6001 . . . . . . . . . . . . . . . . . . . . . . . . . . . . . . . . . . . . . . . . . . . . . . . . . . . . . . . . . . . . . . . . . . . . . . . . . . . . . . . . . . . . . . . . . . . . . . . . . . . . LC461957.1_Japan_Mosquito**

**6001 . . . . . . . . . . . . . . . . . . . . . . . . . . . . . . . . . . . . . . . . . . . . . . . . . . . . . . . . . . . . . . . . . . . . . . . . . . . . . . . . . . . . . . . . . . . . . . . . . . . . LC513838.1_Japan_Mosquito**

**6001 . . . . . . . . . . . . . . . . . . . . . . . . . . . . . . . . . . . . . . . . . . . . . . . . . . . . . . . . . . . . . . . . . . . . . . . . . . . . . . . . . . . . . . . . . . . . . . . . . . . . LC579814.1_Indonesia_Mosquito**

**6001 . . . . . . . . . . . . . . . . . . . . . . . . . . . . . . . . . . . . . . . . . . . . . . . . . . . . . . . . . . . . . . . . . . . . . . . . . . . . . . . . . . . . . . . . . . . . . . . . . . . . LC623822.1_Japan_Mosquito**

**6001 . . . . . . . . . . . . . . . . . . . . . . . . . . . . . . . . . . . . . . . . . . . . . . . . . . . . . . . . . . . . . . . . . . . . . . . . . . . . . . . . . . . . . . . . . . . . . . . . . . . . MH385014.1_China_Mosquito**

**6001 . . . . . . . . . . . . . . . . . . . . . . . . . . . . . . . . . . . . . . . . . . . . . . . . . . . . . . . . . . . . . . . . . . . . . . . . . . . . . . . . . . . . . . . . . . . . . . . . . . . . MK558811.1_China_Mosquito**

**6001 . . . . . . . . . . . . . . . . . . . . . . . . . . . . . . . . . . . . . . . . . . . . . . . . . . . . . . . . . . . . . . . . . . . . . . . . . . . . . . . . . . . . . . . . . . . . . . . . . . . . MT254426.1_China_Mosquito**

**6001 . . . . . . . . . . . . . . . . . . . . . . . . . . . . . . . . . . . . . . . . . . . . . . . . . . . . . . . . . . . . . . . . . . . . . . . . . . . . . . . . . . . . . . . . . . . . . . . . . . . . MT560941.1_China_Mosquito**

**6001 . . . . . . . . . . . . . . . . . . . . . . . . . . . . . . . . . . . . . . . . . . . . . . . . . . . . . . . . . . . . . . . . . . . . . . . . . . . . . . . . . . . . . . . . . . . . . . . . . . . . ON875960_India_Assam_Mosquito**

**L D A R V Y A D H Q A L K W F K D F A A G K R S A V S F I E V L G R M P E H F M G K T R E A L D T M Y L V A T A E K G G K A H R M A L E E L P D A L E T I T L I V A I T V M T G G F F L L M M Q R K G I Majority**

**------------------+-------------------+-------------------+-------------------+-------------------+-------------------+-------------------+-------------------+-------------------+-------------------+-**

**2110 2120 2130 2140 2150 2160 2170 2180 2190 2200**

**------------------+-------------------+-------------------+-------------------+-------------------+-------------------+-------------------+-------------------+-------------------+-------------------+-**

**6301 . . . . . . . . . . . . . . . . . . . . . . . . . . . . . . E P . . . . . . . . . . . . . . . . . . . . . . . . . . . . . . . . . . . . . . . . . . . . . . . . . . . . . . . . . . . . . . . . . . . . AF098735.1_Taiwan_Mosquito**

**6301 . . . . . . . . . . . . . . . . . . . . . . . . . . . . . . . . . . . . . . . . . . . . . . . . . . . . . . . . . . . . . . . . . . . . . . . . . . . . . . . . . . . . . . . . . . . . . . . . . . . . GQ902060.1_Thailand_Mosquito**

**6301 . . . . . . . . . . . . . . . . . . . . . . . . . . . . . . . . . . . . . . . . . . . . . . . . . . . . . . . . . . . . . . . . . . . . . . . . . . . . . . . . . . . . . . . . . . . . . . . . . . . . GQ902063.1_Thailand_Mosquito**

**6301 . . . . . . . . . . . . . . . . . . . . . . . . . . . . . . . . . . . . . . . . . . . . . . . . . . . . . . . . . . . . . . . . . . . . . . . . . . . . . . . . . . . . . . . . . . . . . . . . . . . . HQ652538.1_China_Mosquito**

**6301 . . . . . . . . . . . . . . . . . . . . . . . . . . . . . . . . . . . . . . . . . . . . . . . . . . . . . . . . . . . . . . . . . . . . . . . . . . . . . . . . . . . . . . . . . . . . . . . . . . . . JF499790.1_Taiwan_Mosquito**

**6301 . . . . . . . . . . . . . . . . . . . . . . . . . . . . . . . . . . . . . . . . . . . . . . . . . . . . . . . . . . . . . . . . . . . . . . . . . . . . . . . . . . . . . . . . . . . . . . . . . . . . JN864064.1_China_Mosquito**

**6301 . . . . . . . . . . . . . . . . . . . . . . . . . . . . . . . . . . . . . . . . . . . . . . . . . . . . . . . . . . . . . . . . . . . . . . . . . . . . . . . . . . . . . . . . . . . . . . . . . . . . JQ031753.1_Taiwan_Mosquito**

**6301 . . . . . . . . . . . . . . . . . . . . . . . . . . . . . . . . . . . . . . . . . . . . . . . . . . . . . . . . . . . . . . . . . . . . . . . . . . . . . . . . . . . . . . . . . . . . . . . . . . . . JQ086762.1_China_Mosquito**

**6301 . . . . . . . . . . . . . . . . . . . . . . . . . . . . . . . . . . . . . . . . . . . . . . . . . . . . . . . . . . . . . . . . . . . . . . . . . . . . . . . . . . . . . . . . . . . . . . . . . . . . KT229574.1_China_Mosquito**

**6301 . . . . . . . . . . . . . . . . . . . . . . . . . . . . . . . . . . . . . . . . . . . . . . . . . . . . . . . . . . . . . . . . . . . . . . . . . . . . . . . . . . . . . . . . . . . . . . . . . . . . KT229575.1_China_Mosquito**

**6301 . . . . . . . . . . . . . . . . . . . . . . . . . . . . . . . . . . . . . . . . . . . . . . . . . . . . . . . . . . . . . . . . . . . . . . . . . . . . . . . . . . . . . . . . . . . . . . . . . . . . LC461957.1_Japan_Mosquito**

**6301 . . . . . . . . . . . . . . . . . . . . . . . . . . . . . . . . . . . . . . . . . . . . . . . . . . . . . . . . . . . . . . . . . . . . . . . . . . . . . . . . . . . . . . . . . . . . . . . . . . . . LC513838.1_Japan_Mosquito**

**6301 . . . . . . . . . . . . . . . . . . . . . . . . . I . . . . . . . . . . . . . A . . . . . . . . . . . . . . . . . R . . . . . . . . . . . . . . . . . . . . . . . . . A . . . . . . . . . . . . . . . . LC579814.1_Indonesia_Mosquito**

**6301 . . . . . . . . . . . . . . . . . . . . . . . . . . . . . . . . . . . . . . . . . . . . . . . . . . . . . . . . . . . . . . . . . . . . . . . . . . . . . . . . . . . . . . . . . . . . . . . . . . . . LC623822.1_Japan_Mosquito**

**6301 . . . . . . . . . . . . . . . . . . . . . . . . . . . . . . . . . . . . . . . . . . . . . . . . . . . . . . . . . . . . . . . . . . . . . . . . . . . . . . . . . . . . . T . . . . . . . . . . . . . . MH385014.1_China_Mosquito**

**6301 . . . . . . . . . . . . . . . . . . . . . . . . . . . . . . . . . . . . . . . . . . . . . . . . . . . . . . . . . . . . . . . . . . . . . . . . . . . . . . . . . . . . . . . . . . . . . . . . . . . . MK558811.1_China_Mosquito**

**6301 . . . . . . . . . . . . . . . . . . . . . . . . . . . . . . . . . . . . . . . . . . . . . . . . . . . . . . . . . . . . . . . . . . . . . . . . . . . . . . . . . . . . . . . . . . . . . . . . . . . . MT254426.1_China_Mosquito**

**6301 . . . . . . . . . . . . . . . . . . . . . . . . . . . . . . . . . . . . . . . . . . . . . . . . . . . . . . . . . . . . . . . . . . . . . . . . . . . . . . . . . . . . . . . . . . . . . . . . . . . . MT560941.1_China_Mosquito**

**6301 . . . . . . . . . . . . . . . . . . . . . . . . . . . . . . . . . . . . . . . . . . . . . . . . . . . . . . . . . . . . . . . . . . . . . . . . . . . . . . . . . . . . . . . . . . . . . . . . . . . . ON875960_India_Assam_Mosquito**

**G K M G L G A L V L T L A T F F L W A A E V P G T K I A G T L L V A L L L M V V L I P E P E K Q R S Q T D N Q L A V F L I C V L T V V G V V A A N E Y G M L E K T K A D L K S M F G G R T Q A S G L T G Majority**

**------------------+-------------------+-------------------+-------------------+-------------------+-------------------+-------------------+-------------------+-------------------+-------------------+-**

**2210 2220 2230 2240 2250 2260 2270 2280 2290 2300**

**------------------+-------------------+-------------------+-------------------+-------------------+-------------------+-------------------+-------------------+-------------------+-------------------+-**

**6601 . . . . . . . . . . . . . . . . . . . . . . . . . . . . . . . . I . . . . . . . . . . . . . . . . . . . G . . . . . . . . . . . . . . . . . . . . . . . . . . . . . . . . . . . . . . K . . . . . . . . AF098735.1_Taiwan_Mosquito**

**6601 . . . . . . . . . . . . . . . . . . . . . . . . . . . . . . . . . . . . . . . . . . . . . . . . . . . . . . . . . . . . . . . . . . . . . . . . . . . . . . . . . . . . . . . . . . . . . . . P . . . . GQ902060.1_Thailand_Mosquito**

**6601 . . . . . . . . . . . . . . . . . . . . . . . . . . . . . . . . I . . . . . . . . . . . . . . . . . . . . . . . . . . . . . . . . . . . . . . . . . . . . . . . . . . . . . . . . . . K . . . . . . . . GQ902063.1_Thailand_Mosquito**

**6601 . . . . . . . . . . . . . . . . . . . . . . . . . . . . . . . . . . . . . . . . . . . . . . . . . . . . . . . . . . . . . . . . . . . . . . . . . . . . . . . . . . . . . . . . . . . . . . . P . . . . HQ652538.1_China_Mosquito**

**6601 . . . . . . . . . . . . . . . . . . . . . . . . . . . . . . . . . . . . . . . . . . . . . . . . . . . . . . . . . . . . . . . . . . . . . . . . . . . . . . . . . . . . . . . . . . . . . . . P . . . . JF499790.1_Taiwan_Mosquito**

**6601 . . . . . . . . . . . . . . . . . . . . . . . . . . . . . . . . I . . . . . . . . . . . . . . . . . . . . . . . . . . . . . . . . . . . . . . . . . . . . . . . . . . . . . . . . . . K . . . . . . . . JN864064.1_China_Mosquito**

**6601 . . . . . . . . . . . . . . . . . . . . . . . . . . . . . . . . . . . . . . . . . . . . . . . . . . . . . . . . . . . . . . . . . . . . . . . . . . . . . . . . . . . . . . . . . . . . . . . P . . . . JQ031753.1_Taiwan_Mosquito**

**6601 . . . . . . . . . . . . . . . . . . . . . . . . . . . . . . . . I . . . . . . . . . . . . . . . . . . . . . . . . . . . . . . . . . . . . . . . . . . . . . . . . . . . . . . . . . . K . . . . . . . . JQ086762.1_China_Mosquito**

**6601 . . . . . . . . . . . . . . . . . . . . . . . . . . . . . . . . . . . . . . . . . . . . . . . . . . . . . . . . . . . . . . . . . . . . . . . . . . . . . . . . . . . . . . . . . . . . . . . P . . . . KT229574.1_China_Mosquito**

**6601 . . . . . . . . . . . . . . . . . . . . . . . . . . . . . . . . . . . . . . . . . . . . . . . . . . . . . . . . . . . . . . . . . . . . . . . . . . . . . . . . . . . . . . . . . . . . . . . P . . . . KT229575.1_China_Mosquito**

**6601 . . . . . . . . . . . . . . . . . . . . . . . . . . . . . . . . . . . . . . . . . . . . . . . . . . . . . . . . . . . . . . . . . . . . . . . . . . . . . . . . . . . . . . . . . . . . . . . . . . . . LC461957.1_Japan_Mosquito**

**6601 . . . . . . . . . . . . . . . . . . . . . . . . . . . . . . . . . . . . . . . . . . . . . . . . . . . . . . . . . . . . . . . . . . . . . . . . . . . . . . . . . . . . . . . . . . . . . . . . . . . . LC513838.1_Japan_Mosquito**

**6601 . . . . . . . . . . V . . . . . . . . . . . S . . . . . . . . . I . . . . . . . . . . . . . . . . . . . . . . . . . . . . . . . . . . . . . . . . . . . . . . . . . . . . . . . . . . K A P V . . M . . LC579814.1_Indonesia_Mosquito**

**6601 . . . . . . . . . . . . . . . . . . . . . . . . . . . . . . . . . . . . . . . . . . . . . . . . . . . . . . . . . . . . . . . . . . . . . . . . . . . . . . . . . . . . . . . . . . . K . . . . . . . . LC623822.1_Japan_Mosquito**

**6601 . . . . . . . . . . . . . . . . . . . . . . . . . . . . . . . . . . . . . . . . . . . . . . . . . . . . . . . . . . . . . . . . . . . . M . . . . . . . . . . . . . . . . . . . . S . . . . . P . . . . MH385014.1_China_Mosquito**

**6601 . . . . . . . . . . . . . . . . . . . . . . . . . . . . . . . . . . . . . . . . . . . . . . . . . . . . . . . . . . . . . . . . . . . . . . . . . . . . . . . . . . . . . . . . . . . K . . . . . . . . MK558811.1_China_Mosquito**

**6601 . . . . . . . . . . . . . . . . . . . . . . . . . . . . . . . . . . . . . . . . . . . . . . . . . . . . . . . . . . . . . . . . . . . . . . . . . . . . . . . . . . . . . . . . . . . . . . . P . . . . MT254426.1_China_Mosquito**

**6601 . . . . . . . . . . . . . . . . . . . . . . . . . . . . . . . . . . . . . . . . . . . . . . . . . . . . . . . . . . . . . . . . . . . . M . . . . . . . . . . . . . . . . . . . . . . K . . . . . . I . MT560941.1_China_Mosquito**

**6601 . . . . . . . . . . . . . . . . . . . . . . . . . . . . . . . . . . . . . . . . . . . . . . . . . . . . . . . . . . . . . . . . . . . . . . . . . . . . . . . R . . . . . . . . . . . . . . . P . . . . ON875960_India_Assam_Mosquito**

**L P S M A L D L R P A T A W A L Y G G S T V V L T P L L K H L I T S E Y V T T S L A S I S S Q A G S L F V L P R G V P F T D L D L T V G L V F L G C W G Q I T L T T F L T A M V L V T L H Y G Y M L P G Majority**

**------------------+-------------------+-------------------+-------------------+-------------------+-------------------+-------------------+-------------------+-------------------+-------------------+-**

**2310 2320 2330 2340 2350 2360 2370 2380 2390 2400**

**------------------+-------------------+-------------------+-------------------+-------------------+-------------------+-------------------+-------------------+-------------------+-------------------+-**

**6901 . . . . . . . . . . . . . . . . . . . . . . . . . . . . . . . . . . . . . . . . . . . . N . . . . . . . . . . . . . . . . . . . . . . . . . . . . . . . . . . . . . . . . . . . . A . . . . . . . . . . AF098735.1_Taiwan_Mosquito**

**6901 . . . . . . . . . . . . . . . . . . . . . . . . . . . . . . . . . . . . . . . . . . . . . . . . . . . . . . . . . . . . . . . . . . . . . . . . . . . . . . . . . . . . . . . . . . . . . . . . . . . . GQ902060.1_Thailand_Mosquito**

**6901 . . . . . . . . . . . . . . . . . . . . . . . . . . . . . . . . . . . . . . . . . . . . N . . . . . . . . . . . . . . . . . . . . . . . . . . . . . . . . . . . . . . . . . . . . . . . . . . . . . . . GQ902063.1_Thailand_Mosquito**

**6901 . . . . . . . . . . . . . . . . . . . . . . . . . . . . . . . . . . . . . . . . . . . . . . . . . . . . . . . . . . . . . . . . . . . . . . . . . . . . . . . . . . . . . . . . . . . . . . . . . . . . HQ652538.1_China_Mosquito**

**6901 . . . . . . . . . . . . . . . . . . . . . . . . . . . . . . . . . . . . . . . . . . . . . . . . . . . . . . . . . . . . . . . . . . . . . . . . . . . . . . . . . . . . . . . . . . . . . . . . . . . . JF499790.1_Taiwan_Mosquito**

**6901 . . . . . . . . . . . . . . . . . . . . . . . . . . . . . . . . . . . . . . . . . . . . N . . . . . . . . . . . . . . . . . . . . . . . . . . . . . . . . V . . . . . . . . . . . A . . . . . . . . . . JN864064.1_China_Mosquito**

**6901 . . . . . . . . . . . . . . . . . . . . . . . . . . . . . . . . . . . . . . . . . . . . . . . . . . . . . . . . . . . . . . . . . . . . . . . . . . . . . . . . . . . . . . . . . . . . . . . . . . . . JQ031753.1_Taiwan_Mosquito**

**6901 . . . . . . . . . . . . . . . . . . . . . . . . . . . . . . . . . . . . . . . . . . . . N . . . . . . . . . . . . . . . . . . . . . . . . . . . . . . . . V . . . . . . . . . . . A . . . . . . . . . . JQ086762.1_China_Mosquito**

**6901 . . . . . . . . . . . . . . . . . . . . . . . . . . . . . . . . . . . . . . . . . . . . . . . . . . . . . . . . . . . . . . . . . . . . . . . . . . . . . . . . . . . . . . . . . . . . . . . . . . . . KT229574.1_China_Mosquito**

**6901 . . . . . . . . . . . . . . . . . . . . . . . . . . . . . . . . . . . . . . . . . . . . . . . . . . . . . . . . . . . . . . . . . . . . . . . . . . . . . . . . . . . . . . . . . . . . . . . . . . . . KT229575.1_China_Mosquito**

**6901 . . . . . . . . . . . . . . . . . . . . . . . . . . . . . . . . . . . . . . . . . . . . . . . . . . . . . . . . . . . . . . . . . . . . . . . . . . . . . . . . . . . . . . . . . . . . . . . . . . . . LC461957.1_Japan_Mosquito**

**6901 . . . . . . . . . . . . . . . . . . . . . . . . . . . . . . . . . . . . . . . . . . . . . . . . . . . . . . . . . . . . . . . . . . . . . . . . . . . . . . . . . . . . . . . . . . . . . . . . . . . . LC513838.1_Japan_Mosquito**

**6901 . . G . . . . . . . . . . . . . . . . . . . . . . . . . . . I . . . . . . . . . . . . . N . . . . . . . . . . . . . . . . . . . . . . . . . . . . . . . . . . . . . . . . . V . . T . . . . . . . . . . LC579814.1_Indonesia_Mosquito**

**6901 . . . . . . . . . . . . . . . . . . . . . . . . . . . . . . . . . . . . . . . . . . . . . . . . . . . . . . . . . . . . . . . . . . . . . . . . . . . . . . . . . . . . . . . . . . . . . . . . . . . . LC623822.1_Japan_Mosquito**

**6901 . . . . . . . . . . . . . . . . . . . . . . . . . . . . . . . . . . . . . . . . . . . . . . . . . . . . . . . . . . . . . . . . . . . . . . . . . . . . . . . . . . . . . . . . . . . . . . . . . . . . MH385014.1_China_Mosquito**

**6901 . . . . . . . . . . . . . . . . . . . . . . . . . . . . . . . . . . . . . . . . . . . . . . . . . . . . . . . . . . . . . . . . . . . . . . . . . . . . . . . . . . . . . . . . . . . . . . . . . . . . MK558811.1_China_Mosquito**

**6901 . . . . . . . . . . . . . . . . . . . . . . . . . . . . . . . . . . . . . . . . . . . . . . . . . . . . . . . . . . . . . . . . . . . . . . . . . . . . . . . . . . . . . . . . . . . . . . . . . . . . MT254426.1_China_Mosquito**

**6901 . . . . . . . . . . . . . . . . . . . . . . . . . . . . . . . . . . . . . . . . . . . . . . . . . . . . . . . . . . . . . . . . . . . . . . . . . . . . . . . . . . . . . . . . . . . . . . . . . . . . MT560941.1_China_Mosquito**

**6901 . . . . . . . . . . . . . . . . . . . . . . . . . . . . . . . . . . . . . . . . . . . . . . . . . . . . . . . . . . . . . . . . . . . . . . . . . . . . . . . . . . . . . . . . . . . . . . . . . . . . ON875960_India_Assam_Mosquito**

**W Q A E A L R A A Q R R T A A G I M K N A V V D G M V A T D V P E L E R T T P L M Q K K V G Q V L L I G V S V A A F L V N P N V T T V R E A G V L V T A A T L T L W D N G A S A V W N S T T A T G L C H Majority**

**------------------+-------------------+-------------------+-------------------+-------------------+-------------------+-------------------+-------------------+-------------------+-------------------+-**

**2410 2420 2430 2440 2450 2460 2470 2480 2490 2500**

**------------------+-------------------+-------------------+-------------------+-------------------+-------------------+-------------------+-------------------+-------------------+-------------------+-**

**7201 . . . . . . . . . . . . . . . . . . . . . . . . . . . . . . . . . . . . . . . . . . . . . . . . . . . . . . . . . . . . . . . . . . . . . . . . . . . . . . . . . . . . . . . . . . . . . . . . . . . . AF098735.1_Taiwan_Mosquito**

**7201 . . . . . . . . . . . . . . . . . . . . . . . . . . . . . . . . . . . . . . . . . . . . . . . . . . . . . . . . . . . . . . . . . . . . . . . . . . . . . . . . . . . . . . . . . . . . . . . . . . . . GQ902060.1_Thailand_Mosquito**

**7201 . . . . . . . . . . . . . . . . . . . . . . . . . . . . . . . . . . . . . . . . . . . . . . . . . . . . . . . . . . . . . . . . . . . . . . . . . . . . . . . . . . . . . . . . . . . . . . . . . . . . GQ902063.1_Thailand_Mosquito**

**7201 . . . . . . . . . . . . . . . . . . . . . . . . . . . . . . . . . . . . . . . . . . . . . . . . . . . . . . . . . . . . . . . . . . . . . . . . . . . . . . . . . . . . . . . . . . . . . . . . . . . . HQ652538.1_China_Mosquito**

**7201 . . . . . . . . . . . . . . . . . . . . . . . . . . . . . . . . . . . . . . . . . . . . . . . . . . . . . . . . . . . . . . . . . . . . . . . . . . . . . . . . . . . . . . . . . . . . . . . . . . . . JF499790.1_Taiwan_Mosquito**

**7201 . . . . . . . . . . . . . . . . . . . . . . . . . . . . . . . . . . . . . . . . . . . . . . . . . . . . . . . . . . . . . . . . . . A . . . . A . . . . . . . . . . . . . . . . . . . . . . . . . . . . JN864064.1_China_Mosquito**

**7201 . . . . . . . . . . . . . . . . . . . . . . . . . . . . . . . . . . . . . . . . . . . . . . . . . . . . . . . . . . . . . . . . . . . . . . . . . . . . . . . . . . . . . . . . . . . . . . . . . . . . JQ031753.1_Taiwan_Mosquito**

**7201 . . . . . . . . . . . . . . . . . . . . . . . . . . . . . . . . . . . . . . . . . . . . . . . . . . . . . . . . . . . . . . . . . . A . . . . A . . . . . . . . . . . . . . . . . . . . . . . . . . . . JQ086762.1_China_Mosquito**

**7201 . . . . . . . . . . . . . . . . . . . . . . . . . . . . . . . . . . . . . . . . . . . . . . . . . . . . . . . . . . . . . . . . . . . . . . . . . . . . . . . . . . . . . . . . . . . . . . . . . . . . KT229574.1_China_Mosquito**

**7201 . . . . . . . . . . . . . . . . . . . . . . . . . . . . . . . . . . . . . . . . . . . . . . . . . . . . . . . . . . . . . . . . . . . . . . . . . . . . . . . . . . . . . . . . . . . . . . . . . . . . KT229575.1_China_Mosquito**

**7201 . . . . . . . . . . . . . . . . . . . . . . . . . . . . . . . . . . . . . . . . . . . . . . . . . . . . . . . . . . . . . . . . . . . . . . . . . . . . . . . . . . . . . . . . . . . . . . . . . . . . LC461957.1_Japan_Mosquito**

**7201 . . . . . . . . . . . . . . . . . . . . . . . . . . . . . . . . . . . . . . . . . . . . . . . . . . . . . . . . . . . . . . . . . . . . . . . . . . . . . . . . . . . . . . . . . . . . . . . . . . . . LC513838.1_Japan_Mosquito**

**7201 . . . . . . . . . . . . . . . . . . . . . . . . . . . . . . . . . . . . . . . . . . . . . . . . . . . . . . . . . . . . . . . . . . . . . . . . . . . . . . . . . . . . . . . . . . . . . . . . . . . . LC579814.1_Indonesia_Mosquito**

**7201 . . . . . . . . . . . . . . . . . . . . . . . . . . . . . . . . . . . . . . . . . . . . . . . . . . . . . . . . . . . . . . . . . . . . . . . . . . . . . . . . . . . . . . . . . . . . . . . . . . . . LC623822.1_Japan_Mosquito**

**7201 . . . . . . . . . . . . . . . . . . . . . . . . . . . . . . . . . . . . . . . . . . . . . . . . . . . . . . . . . . . . . . . . . . . . . . . . . . . . . . . . . . . . . . . . . . . . . . . . . . . . MH385014.1_China_Mosquito**

**7201 . . . . . . . . . . . . . . . . . . . . . . . . . . . . . . . . . . . . . . . . . . . . . . . . . . . . . . . . . . . . . . . . . . . . . . . . . . . . . . . . . . . . . . . . . . . . . . . . . . . . MK558811.1_China_Mosquito**

**7201 . . . . . . . . . . . . . . . . . . . . . . . . . . . . . . . . . . . . . . . . . . . . . . . . . . . . . . . . . . . . . . . . . . . . . . . . . . . . . . . . . . . . . . . . . . . . . . . . . . . . MT254426.1_China_Mosquito**

**7201 . . . . . . . . . . . . . . . . . . . . . . . . . . . . . . . . . . . . . . . . . . . . . . . . . . . . . . . . . . . . . . . . . . . . . . . . . . . . . . . . . . . . . . . . . . . . . . . . . . . . MT560941.1_China_Mosquito**

**7201 . . . . . . . . . . . . . . . . . . . . . . . . . . . . . . . . . . . . . . . . . . . . . . . . . . . . . . . . . . . . . . . . . . . . . . . . . . . . . . . . . . . . . . . . . . . . . . . . . . . . ON875960_India_Assam_Mosquito**

**V M R G S Y L A G G S I A W T L I K N A D K P S L K R G R P G G R T L G E Q W K E K L N A X E Q G - V L Q I Q K R G H N - G G P H - S T Q G - A R E Q H S G R T S S L A R V S K A P L A R G K R I C L A Majority**

**------------------+-------------------+-------------------+-------------------+-------------------+-------------------+-------------------+-------------------+-------------------+-------------------+-**

**2510 2520 2530 2540 2550 2560 2570 2580 2590 2600**

**------------------+-------------------+-------------------+-------------------+-------------------+-------------------+-------------------+-------------------+-------------------+-------------------+-**

**7501 . . . . . . . . . . . . . . . . . . . . . . . . . . . . . . . . . . . . . . . . . . . . . X . . R R . F . . P E . . . . R . . . . . . . . . . T . K . . . . . . . G F . . L . . T . . . . . E . . . . . AF098735.1_Taiwan_Mosquito**

**7501 . . . . . . . . . . . . . . . . . . . . . . . . . . . . . . . . . . . . . . . . . . . . . X . . . . . . . . . E . S . . . . . . . . . . . . . . . . . . . . . . P . F . . . . . . . . . . . . G . . . . GQ902060.1_Thailand_Mosquito**

**7501 . . . . . . . . . . . . . . . . . . . . . . . . . . . . . . . . . . . . . . . . . . . . . X . . R R . F . . P E . . . . R . . . . . . . . S . T . K . R . . . . . G F . G L . . T . . . . . E . . . . . GQ902063.1_Thailand_Mosquito**

**7501 . . . . . . . . . . . . . . . . . . . . . . . . . . . . . . . . . . . . . . . . . R . . . X . . . . . . . . . . . . . . . . . . . . . . . . . . . . . . . . . . . . . . . . . . . . . . . . . . . R . . HQ652538.1_China_Mosquito**

**7501 . . . . . . . . . . . . . . . . . . . . . . . . . . . . . . . . . . . . . . . . . . . . . X . . . . . F . . . . . . . . . S . . . . . . . . . . . . . . . . . P . . . . . . . . . . . . . . . . . . . . JF499790.1_Taiwan_Mosquito**

**7501 . . . . . . . . . . . . . . . . . . . . . . . . . . . . X . . . . . P R . . . . . . . . . H . . R R . F . . P E . . . . R . . . . . . . . . Q T . K . . . . . . . G F . . L . . T . . . . . E . . . . . JN864064.1_China_Mosquito**

**7501 . . . . . . . . . . . . . . . . . . . . . . . . . . . . . . . . . . . . . . . . . . . . . X . . . . . . . . . . . . . . . S . . . . . . . . . . . . . . . . . P . . . . . . . . . . . . . . . . . . . . JQ031753.1_Taiwan_Mosquito**

**7501 . . . . . . . . . . . . . . . . . . . . . . . . . . . . . . . . . . P R . . . . . . . . . X . . R R . F . . P E . . . . R . . . . . . . . . Q T . K . . . . . . . G F . . L . . T . . . . . E . . . . . JQ086762.1_China_Mosquito**

**7501 . . . . . . . . . . . . . . . . . . . . . . . . . . . . . . . . . . . . . . . . . . . . . X . . . . . . . . . . . . . . . . . . . . . . . . . . . K . . . . . . . . . . . . . . . . . . . . . . . . . . KT229574.1_China_Mosquito**

**7501 . . . . . . . . . . . . . . . . . . . . . . . . . . . . . . . . . . . . . . . . . . . . . X . . . . . . . . . . . . . . . . . . . . . . . . . . . K . . . . . . . . . . . . . . . . . . . . . . . . . . KT229575.1_China_Mosquito**

**7501 . . . . . . . . . . . . . . . . . . . . . . . . . . . . . . . . . . . . . . . . . . . . . X . . . . . . . . . . . . . . . . . . . . . . . . . . . . . . . . . . . . . . . . . . . . . . . . . . . . . . LC461957.1_Japan_Mosquito**

**7501 . . . . . . . . . . . . . . . . . . . . . . . . . . . . . . . . . . . . . . . . . . . . . X . . . . . . . . . . . . . . . . . . . . . . . . . . . . . . . . . . . . . . . . . . . . . . . . . . . . . . LC513838.1_Japan_Mosquito**

**7501 . . . . . . . . . . . . . . . . . . . . . . . . . . . . . . . . . . . . . . . . . R . . . X . . R R I F . . . E G S . . . . . . . . G . . S Q . . . . Q . . . A . R F . . F . . T S . . C . . . . R F T LC579814.1_Indonesia_Mosquito**

**7501 . . . . . . . . . . . . . . . . . . . . . . . . . . . . . . . . . . . . . . . . . . . . . X . . . . I . . . . . . . . . . . . . . . . . . . . . . . . . . . . . . . . . . . . . . . . . . . . . . . . . LC623822.1_Japan_Mosquito**

**7501 . . . . . . . . . . . . . . . . . . . . . . . . . . . . . . . . . . . . . . . . . . . . . X . . . . . . . . . . . . . . . . . . . . . . . . . . . . . . . . . . . . . . . . . . . . . . . . . . . . . . MH385014.1_China_Mosquito**

**7501 . . . . . . . . . . . . . . . . . . . . . . . . . . . . . . . . . . . . . . . . . . . . . X . . R . . . . . . . . . . . . . . S . . . . . . . . . . . . . . . . . . . . . . . . . . . . . . . . . . . . MK558811.1_China_Mosquito**

**7501 . . . . . . . . . . . . . . . . . . . . . . . . . . . . . . . . . . . . . . . . . . . . . X . . . . . . . . . . . . . . . . . . . . . . . . . . . K . . . . . . . . . . . . . . . . . . . . . . V . . . MT254426.1_China_Mosquito**

**7501 . . . . . . . . . . . . . . . . . . . . . . . . . . . . . . . . . . . . . . . . . . . . . X . . . . . . . . . . . . . . . . . . . . . . . . . . . . . . T . . . . . . . . . . . . . . . . . . . . . . . MT560941.1_China_Mosquito**

**7501 . . . . . . . . . . . . . . . . . . . . . . . . . . . . . . . . . . . . . . . . . . . . . X . . . . . . . . . . . . . . . . . . . . . . . . . . . . . . . . . . . . . . . . . . . . . . . . . . . R . . ON875960_India_Assam_Mosquito**

**N R K S H R S G M R A R R L E L L R S N S E K S S G S Q R V H E R W G G T R R T D A H A E L R L E P G L A K E W G G R I L Q T L G A - - H P V L - H R R I F P K S R G G G T T H A A R P R N D I - L V T Majority**

**------------------+-------------------+-------------------+-------------------+-------------------+-------------------+-------------------+-------------------+-------------------+-------------------+-**

**2610 2620 2630 2640 2650 2660 2670 2680 2690 2700**

**------------------+-------------------+-------------------+-------------------+-------------------+-------------------+-------------------+-------------------+-------------------+-------------------+-**

**7798 . . . . . . . R V W . W . M . . . . . . P . E G P . . . . I . . . . . . . . . . . . . . . . . . . . . . P E . . S . . V . . . F R . Q . . S . . R . . G . L . . . . S R R . . . T T . . . D . . . . . A AF098735.1_Taiwan_Mosquito**

**7798 . . . . . . . . . W . W . . . . . . . . . . . . . . . . . . . . . . . . . . . . . . . . . . . . . . . . . . . . . . . V . . . . . . Q . . . . . . . . . . . . . . . S . . . . . . . . L . . . . . . A . GQ902060.1_Thailand_Mosquito**

**7798 . . . . . . . R V W . W . M . . . . . . P . E G P . G . . I . . . . . . . . . . . . . . . . . . . L . . P E . . S . . V . . . F R . Q . . . . . . . . G . L . . . . . R . . . . T T . . . D . V . . . A GQ902063.1_Thailand_Mosquito**

**7798 . . . . . . . . . . . . . . . . . . . . . . . . . . . . . . . . . . . . . . . . . . . . . . . . . . . . . . . . S . . . . . . . . . . . . . . . . . . . . . . . . . . . . . . . . . . L . . . . . . . . HQ652538.1_China_Mosquito**

**7798 . . . . . . . . . . T . . . . . . . . . . . . . . . . . . . . . . . . . . . . . . . . . . . . . . . . . V . . . . . . . . . . . . . . . . . . . . . . . . . . . . . . . . . . . . . . . . . . . . . . . JF499790.1_Taiwan_Mosquito**

**7798 . . . . . . . R V W . W . M . . . . . . P . E G P . . . . I . . . . . . . . . . . . . . . . . . . . . . P E . . S . . V . . . F R . Q . Y . . . . . . G . L . . . . S R R . . . T T . . . D . . . . . A JN864064.1_China_Mosquito**

**7798 . . . . . . . . . . T . . . . . . . . . . . . . . . . . . I . . . . . . . . . . . . . . . . . . . . . F V . . . . . . V . . . . . . . . . . . . . . . . . . . . . . . . . . . . . . . . . . . . . . . . JQ031753.1_Taiwan_Mosquito**

**7798 . . . . . . . R V W . W . M . . . . . . P . E G P . . . . I . . . . . . . . . . . . . . . . . . . . . . P E . . S . . V . . . F R . Q . Y . . . . . . G . L . . . . S R R . . . T T . . . D . . . . . A JQ086762.1_China_Mosquito**

**7798 . . . . . . . . . . . . . . . . . . . . . . . . . . . . . . . . . . . . . . . . . . . . . . . . . . . . V . . . . . . V . . . . . . . . . . . . . . . G . . . . . . . . . . . . . . . . . . . . . . . . KT229574.1_China_Mosquito**

**7798 . . . . . . . . . . . . . . . . . . . . . . . . . . . . . . . . . . . . . . . . . . . . . . . . . . . . V . . . . . . V . . . . . . . . . . . . . . . G . . . . . . . . . . . . . . . . . . . . . . . . KT229575.1_China_Mosquito**

**7798 . . E . . . . . . . . . . . . . . . . . . . . . . . . . . . . . . . . . . . . . . . . . . . . . . . . . . E K . . . . . . . . . . . . . Y . . . . . . . . I . . . G . . . . . . . . . L . . . . . . . . LC461957.1_Japan_Mosquito**

**7798 . . . . . . . . . . . . . . . . . . . . P . . . . . . . . . . . . . . . . . . . . . . . . . . . . . . . . . K . . . . . . . . . . . . . Y . . . . . . . . I . . . G . . . . . . . . . L . . . . . . . . LC513838.1_Japan_Mosquito**

**7798 . . . . . . P R V W T W . . . . . . G . . . E . . . G E . I . . . R . R A . G . H . . . . . W V . . . . T . . . S R . V . . A . . T . . . L A . . . . G . . . . . . S . R . . N P . S . G . . F . . . A LC579814.1_Indonesia_Mosquito**

**7798 . . . . . . . . . . . . . . . . . . . . . . . . . . . . . . . . G . . . . . . . . T . . . . . . . . . . . . K . . . . . . . . . . . . . . . . . . . . . . . . . . G . . . . . . . . . L . . . . . . . . LC623822.1_Japan_Mosquito**

**7798 . . . . . . . . . . . . . . . . . . . . . . . . . . . . . . . . . . . . . . . . . . . . . . . . . . . . V . . . . . . . . . . . . . . . . S . . . . . . . L . . . . . . . . . . . . . . . . . . . . . . MH385014.1_China_Mosquito**

**7798 . . . . . . . . . . . . . . . . . . . . . . . . . . . . . . . . . . . . . . . . N . . . . . . . . . . . . . K . . . . . . . . . . . . . . . . . . . . . . . S . . G . . . . . . . . . L . . . . . . . . MK558811.1_China_Mosquito**

**7798 . . . . . . . . . . . . . . . . . . . . . . . . . . . . . . . . . . . . . . . . . . . . . . . . . . . . V . . . . . . . . . . . . . . . . . . . . . . . . . . . . . . . . . . . . . . . . . . . . . . . MT254426.1_China_Mosquito**

**7798 . . . . . . . . . W . . . . . . . . . . . . . . . . . . . . . . . . . . . . . . N . . . . . . . . . . . . . K . . . . . . . . . . . . . . . . . . . . . . . . . . G . . . . . . . . . L . . . . . . . . MT560941.1_China_Mosquito**

**7798 . . . . . . . . . . . . . . . . . . . . . . . . . . . . . . . . . . . . . . . . . . . . . . . . . . . . V . . . . . C . . . . . . . . . . S . . . . . . . L . . . . . . . . . . . . . . . . . . . . . . ON875960_India_Assam_Mosquito**

**S G T - R V L H K S A L P L H A - S Y R K D G S P A T S F R W R A G A P P P V S K L Q P - D V L G E W S C W Q C G A C G Q H D Q P S A A R A N G S H S V E R A K V - R G C Q P G Q R D E S C G E G R G P Majority**

**------------------+-------------------+-------------------+-------------------+-------------------+-------------------+-------------------+-------------------+-------------------+-------------------+-**

**2710 2720 2730 2740 2750 2760 2770 2780 2790 2800**

**------------------+-------------------+-------------------+-------------------+-------------------+-------------------+-------------------+-------------------+-------------------+-------------------+-**

**8098 P R . . . . . . . . S . . . . . Q G . . . N . . S . A P L . R W . S . S . . . P Q . . S R . . . . . . . R . . . . . R C E . . . . G T T G . . . . . . . . . . . . . G R . . . R E . N K . R . K . . S . AF098735.1_Taiwan_Mosquito**

**8098 . . . . . . . . . . . . . . . . Q . . . . . . . . . . . L . G . . . . . . L . P . . . . . . . . . . . G . R . . . . . S . . . . . . . . . . . . . . . . . . . . . . . . . . . . . . . K . . . . . . . . GQ902060.1_Thailand_Mosquito**

**8098 P R . . . . . . . . S . . . . . Q G . . . N . . S . A P . . R W . S . S . L . P . . . S R . . . . . . . . . . . . . R C E . . . . G T T G . . . P . . . . . . . . . G R . . . R E . N K . . . K . . N . GQ902063.1_Thailand_Mosquito**

**8098 . . . . . . . . . . . . . . . . . . . . . . . . L . . . L . . . . . . . . . . . . . . . . . . . . . . . . . . . . . . . . . . . . . . . . . . . . . . . . . . . . . . . . . . . . . . . . . . . . . . . HQ652538.1_China_Mosquito**

**8098 . . . . . . . . . . . . . . . . . . . . . . . . . . . . . . . . . . . . S . . . . . . . . . . . . . . . . . . . . . . S . . . . . . . . . . . . . . . . . . . . . . . . . . . . . . . . . . . . . . . . JF499790.1_Taiwan_Mosquito**

**8098 P R . . . . . . . . S . . . . . Q G . . . N . . S . A . L . R W . S . S . . . P . . . S R . . . . . . . R . . . . . R C E . . . . G I T G . . . . . . . . . . . . . G R . . . R E . N K . R . K . . S . JN864064.1_China_Mosquito**

**8098 . . . . . . . . . . . . . . . . . . . . . . . . . . . . . . . . . . . . S . . . . . . . . . . . . . . . . . . . . . . S . . . . . . . . . . . . . R . . . . . . . . . . . . . . . . . . . . . . . . . . JQ031753.1_Taiwan_Mosquito**

**8098 P R . . . . . . . . S . . . . . Q G . . . N . . S . A . L . R W . S . S . . . P . . . S R . . . . . . . R . . . . . R C E . . . . G I T G . . . . . . . . . . . . . G R . . . R E . N K . R . K . . S . JQ086762.1_China_Mosquito**

**8098 . . . . . . . . . . . . . . . . . . . . . . . . . . . . . . . . . . . . S . . . . . . . . . . . . . . . . . . . . . . . . . . . . . . . . . D . . . . . . . . . . . . . . . . . . . . . . . . . . . . . KT229574.1_China_Mosquito**

**8098 . . . . . . . . . . . . . . . . . . . . . . . . . . . . . . . . . . . . S . . . . . . . . . . . . . . . . . . . . . . . . . . . . . . . . . D . . . . . . . . . . . . . . . . . . . . . . . . . . . . . KT229575.1_China_Mosquito**

**8098 . . . . . . . . . . . . . I . . . . . . . . . . . . . . . . . . . . . . S . . . . . . . . . . . . . R . R . . R . . . . . . . . S . . . . . . . . . . . . . . . . . . . . . . . . . . . . R . . . . . . LC461957.1_Japan_Mosquito**

**8098 . . . . . . . . . . . . . . . . . . . . . . . . . . . . . . . . . . . . S L . . . . . . . . . . . . R . R . . R . . . . . . . . S . . . . . . . . . . . . . . . . . . . . . . . . . . . . . . . . . . . LC513838.1_Japan_Mosquito**

**8098 Q R P . . I . . . G . . . . . . . . H . E . . . A . A P L W R . . C . S . F . P E . K S . . . . . . R . S . . . . . R . E . . . . G . . . . . . P . C . . . T . . R . . . . L . E W N Q . R R . . . S . LC579814.1_Indonesia_Mosquito**

**8098 . . . . . . . . . . . . . . . . . . . . . . . . . . . . . . . . . . . . . . . . . . . . . . . . . . R . . . . R . . . . . . . . S . . . . . . . . . N . . . . . . . . . . . . . . . N . . . . . . . D . LC623822.1_Japan_Mosquito**

**8098 . . . . . . . . . . . . . . . . . . . . . . . . . . . . . . . . . . . . S . . P . . . . . . . . . . . . . . . . . . . . . . . . . . . . . . . . . . . . . . . E . . . . . . . . . . . . . . . . . . . . MH385014.1_China_Mosquito**

**8098 . . . . . . . . . . . . . . . . . . . . . . . . . . . . . . . . . . . . . . . . . . . . . . . . . . R . . . . . . . . . . . . . S . . T . . . . . . . . . G . . . . . . . . . . . . . . . . . . . . . . MK558811.1_China_Mosquito**

**8098 . . . . . . . . . . . . . . . . . . . . . . . . . . A . . . . . . . . . S S . P . . . . . . . . . . . . . . . . . . . . . Y . . . . T . . . . . . . . . . . . . . . . . . . . . . . . . . . . . . . . . MT254426.1_China_Mosquito**

**8098 . . . . . . . . . . . . . . . . . . . . . . . . . . A . . . . . . . . . . . . . . . . . . . . . . . R . . . . R . . . . . . . . S . . . . E . . . . . . . . . . . . . . . . . . . . . . . . . . . . . . MT560941.1_China_Mosquito**

**8098 . . . . . . . . . . . . . . . . . . . . . . . . . . . . . . . . . . . . S . . P . . . . . . . . . . . . . . . . . . . . . . . . . G . . . . . . . . . . . . . E . . . . . . . . . . . . . . . . R . . . ON875960_India_Assam_Mosquito**

**- Q P G K N - E E N P E A - R G I R H N L A Q R P R A S I P N L D L S R K L R S E G H W L S K L S R Q W G G K A H E Q T L G R H R Q C H H N G H D R H H P L W P A E G L - R E G - H E G S R A T S R S Q Majority**

**------------------+-------------------+-------------------+-------------------+-------------------+-------------------+-------------------+-------------------+-------------------+-------------------+-**

**2810 2820 2830 2840 2850 2860 2870 2880 2890 2900**

**------------------+-------------------+-------------------+-------------------+-------------------+-------------------+-------------------+-------------------+-------------------+-------------------+-**

**8398 . . S . E . Q . . . . . . . . R . . . . V . . . . . . . . . H . . I P . . . . . . . Y . . . . . . . . R S . E . . . . . . . . . C . R . . H . . . . . . . F . T . K S F Q G . S . . . . . . . . . W . . AF098735.1_Taiwan_Mosquito**

**8398 . . . . . . . . . . . . . . . . . C . . . . . . . . . . . . . . . . . . . . . . . . . . . R E . . . . . . . . . . . . . . . C . . . . . . . . . . . . Y . . . . . K . . . . . . R . . . . . . . . . . . GQ902060.1_Thailand_Mosquito**

**8398 . . S . E . Q . . . . K . . . R . . . . V . . . . . . . . . H . . I P . . . . . . . Y . . . Q . . C . R S . E . . . . . . . . . C . R . . H . . . . . . . F . T . K S F Q G . S . . . . . . . . . W . . GQ902063.1_Thailand_Mosquito**

**8398 Q . . . . . . . . . . . . . G . . H . . . . . . . . . P . . . . . . . . . . . . . . . . . . . . P . . . . . . . . . . . . . . . . . . . . . . . . . . . . . . . . . S . . . . . . . . . . . . . . . . . HQ652538.1_China_Mosquito**

**8398 . . . . . . . . . . . . T . . . . . . . . . . G . . . . . . . . . . . . . . . . . . . . . . . . . . . . . . . . . . . . . . . . . . . . . . . . . . . . . . . . . . . . . . . . . . . . . . . . . . . . JF499790.1_Taiwan_Mosquito**

**8398 . . S . E . Q . . . . . . . . R . . . . V . . . . . . . . . H . . I P . . . . . . . Y . . . Q . . . . R S . E . . . . . . . . . C . R . . H . . . . . . . F . T . K S F Q G . S . . . . . . . . . W . . JN864064.1_China_Mosquito**

**8398 . . . . . . . . . . . . T . . . . . . . . . . . . . . . . . . . . . . . . . . . . . . . . . . . . . . . . . . . . . . . . . . . . . . . . . . . . . . . . . . . . . . . . . . . . . . . . . . . . . . . JQ031753.1_Taiwan_Mosquito**

**8398 . . S . E . Q . . . . . . . . R . . . . V . . . . . . . . . H . . I P . . . . . . . Y . . . Q . . . . R S . E . . . . . . . . . C . R . . H . . . . . . . F . T . K S F Q G . S . . . . . . . . . W . . JQ086762.1_China_Mosquito**

**8398 . . . . . . . . . . . . T . . . . . . . . . . . . . T . . . . . . . . . . . . . . . . . . . . . . . . . . . . . . . . . . . . . . . . . . D . . . . . . . . . . . . . . . . . . . . . . . . . . . . . . KT229574.1_China_Mosquito**

**8398 . . . . . . . . . . . . T . . . . . . . . . . . . . T . . . . . . . . . . . . . . . . . . . . . . . . . . . . . . . . . . . . . . . . . . D . . . . . . . . . . . . . . . . . . . . . . . . . . . . . . KT229575.1_China_Mosquito**

**8398 . . . . . . . . . . . . . . . . . . . . . V . . . . . . . . . . . . . . . . . . . . . . . . . F . . . . . . . . . . . . . . . . . . . . . . . . . . . . . . . S . . . . . . . . . . . . . . T . . . . . LC461957.1_Japan_Mosquito**

**8398 . . . . . . . . . . . . . . . . . . . . . . . . . . . . . . . . . . . . . . . . . . . . . . E F . . . . . . . . . . . . . . . . . . . . . . . . . . . . . . . S . . . . . . . . . . . . . . T . . . . . LC513838.1_Japan_Mosquito**

**8398 Q . . R . . . . T . . K . E . . . . R D M . . G . . . P V S H . . I P . . . . . . S . . . . . . . C . . S . E . N . . A . . . . S . . . . . . . . . Y N S . R T . . S . Q . K S . . K S . . . . I W . . LC579814.1_Indonesia_Mosquito**

**8398 . . . . . . . . . . . . . . . . . . . . . . . . . . . . . . . . . . . . . . . . . . . . . . . F . . . . . . . . . . . . . . . . . . . . . . . . . . . . . . . . . . . . . . . . . . . . . . T . . . . . LC623822.1_Japan_Mosquito**

**8398 . . . . . . . . . . . . T . . . . . Y . . . . . . . . . . . . . . . . . . . . . . . . . . . . . . . . . . . . . . . . . . . . . . . . . . . . . . . . . S . . . . . . . . . . . . . . S . . . . . . . . MH385014.1_China_Mosquito**

**8398 . . . . . . . . . . . . . . . . . . . . . . . . . . . . . . . . . . . . . . . . . . . . . . . F . . . . . S . . . . . . . . . . . . . . . . . . . . . . . . . . . . . . . . . . . . . . . . T . . . . . MK558811.1_China_Mosquito**

**8398 . . . . . . . . . . . . T . . . . . Y . . . . . . . . . . S . . . . . W . . . . . . . . . . E . . . . . . S E . . . . . . . . . . . . . . . . . . . . . . . . . . . . . . . . . . . . S . . . . . . . . MT254426.1_China_Mosquito**

**8398 . . . . . . . . . . . . . . . . . . . . . . . . . . . . . . . . . . . . . . . . . . . . . . . F . . . . . . . . . . . . . . . . . . . . . . . . . . . . . . . . . . . . . . . . . . . . . . T . . . . . MT560941.1_China_Mosquito**

**8398 . . . . . . . . . D . . T . . . . . Y . . . . . . . . . . . . . . . . . . . . . . . . . . . . . . . . . . . . . . . . . . . . . . . . . . . . . . . . . . . . . . . . . . . . . . . . S P . . . . . . . ON875960_India_Assam_Mosquito**

**G S A Q R D H Q L A V G P L V T G E T T P L V H - G R I H K E S Q Q Q R S S R S S V R - T E S M E H G A G S R G - P S V L G D G Q - R K G K P F A R G V P H V H L Q H D G K K R E K T R R V R K G - R E Majority**

**------------------+-------------------+-------------------+-------------------+-------------------+-------------------+-------------------+-------------------+-------------------+-------------------+-**

**2910 2920 2930 2940 2950 2960 2970 2980 2990 3000**

**------------------+-------------------+-------------------+-------------------+-------------------+-------------------+-------------------+-------------------+-------------------+-------------------+-**

**8698 . . . . . . . . . . . . L . . . . K K . . . . . Q . . . . . . . . . . . G . W . . . . . . . . . . . . . . G C . . . A . . . . . . . . E . . . S . . R . S . M Y . . . . . . . . . E A W . . W . S . . K AF098735.1_Taiwan_Mosquito**

**8698 . . . . . . Y . . . . . . . . . . . . . . . . Y Q . . . . . . . . . . C . . W . . . . . . . . . . . S . . G . . . . . . . . . . R . . . . . . . . . . . . . . . . . . . . . . . . . . W . . . Q . . . K GQ902060.1_Thailand_Mosquito**

**8698 . . . . . . . . . . . . . . . . . K K . . . . . . . . . . . . . . . . . G . W . G . . . . . . . . . . . . G C . . . . . . . . . . . . E R . . . . . R . S . M Y . . . . . . E . . E A W . . W E S Q . K GQ902063.1_Thailand_Mosquito**

**8698 . . . . . . Y . . . . . . . . . . . . . . F . . Q . . . . . . . . . . . . . . . . . . . . . . . . . S . . . C . . . . . . . . . . . . . . . . . . . . . . . . . . . . . . . . . . . . . . . . . . . G . HQ652538.1_China_Mosquito**

**8698 . . . . . . . . . . . . . . . . . . A . . . . . Q . . . . . . . . . . . . . . . . . C . . . . . . . . . . . . . R . . . . . . . . R . . . . . . . . . . . . . . . . . . . . E . . . . . . . . . . . . . JF499790.1_Taiwan_Mosquito**

**8698 . . . . . . . . . . . . L . . . . K K . . . . . Q . . . . . . . . . . . G . W . . . . . . . . . . . . . . G C . . . A . . . . . . . . E . . . S . . R . S . M Y . . . . . . . . . E A W . . W . S . . K JN864064.1_China_Mosquito**

**8698 . . . . . . . . . . . . . . . . . . A . . . . . . . . . . . . . . . . . . . . . . . . . . . . . . . . . . . . . R . . . . . . . . R . . . . . . . . . . . . . . . . . . . . E . . . . . . . . . . . . . JQ031753.1_Taiwan_Mosquito**

**8698 . . . . . . . . . . . . L . . . . K K . . . . . Q . . . . . . . . . . . G . W . . . . . . . . . . . . . . G C . . . A . . . . . . . . E . . . S . . R . S . M Y . . . . . . . . . E A W . . W . S . . K JQ086762.1_China_Mosquito**

**8698 . . . . . . . . . . . . . . . . . . . . . . . . . . . . . . . . . . . . . . . . . . . . . . . . . . . . . . . . R . . . . . . . . R . . . . . . . . . . . . . . . . . . . . . . . . . . . . . . . . . . KT229574.1_China_Mosquito**

**8698 . . . . . . . . . . . . . . . . . . . . . . . . . . . . . . . . . . . . . . . . . . . . . . . . . . . . . . . . R . . . . . . . . R . . . . . . . . . . . . . . . . . . . . . . . . . . . . . . . . . . KT229575.1_China_Mosquito**

**8698 . . . . . . Y . . V . . . P . . . . . . . . . . . . . . . . . . . . . . . . . . . . . . . . . . . . . . . G . . . . . . . . . . . . . . . . . . . . . . . . . . . . . . . . . . . . . . . . . . . . . . LC461957.1_Japan_Mosquito**

**8698 . . . . . . Y . . V . . . P . . . . . . . . . . . . . . . . . . . . . . . . . . . . . . . . . . . . . . . G . . . . . . . . . . . . . . . . . . . . . . . . . . . . . . . . . . . . . . . . . . . . . . LC513838.1_Japan_Mosquito**

**8698 . . . . . N . K . . . . L F . P . K K . S F . . E . . V Y . . . . . . . G P . . . I . . . . . V . Y S T . . . . . . A L . . . . R C G . . . . P . . R M . . M R . . . . . . . . . E . W . . . E S Q . K LC579814.1_Indonesia_Mosquito**

**8698 . . . . . . Y . . . . . . . . . . . . . . . . . . . . . . . . . . . . . . . . . . . . . . . . . . . . . . G . . . . . . . . . . . . . . . . . . . . R . . . . . . . . . . . . . . . . . . . . . . . . . LC623822.1_Japan_Mosquito**

**8698 . . . . . . . . . . . . . . . . . . . . . . . . . . . . . . . . . . . . . . . . . . . . . . . . . . . . . . . . R . . . . . . . . . . . . . . . . . . . . . . . . . . . . . . . . . . . . . . . . . . . MH385014.1_China_Mosquito**

**8698 . . . . . . Y . . . . . . . . . . . . . . . . Y . . . . . . . . . . . . . . . . . . . . . . . . . . . . . . . . . . . . . . . . . R . . . . . . . . . . . . . . . . . . . E . . . . . . . . . . . . . . MK558811.1_China_Mosquito**

**8698 . . . . . N . . . . . . . . . . . . . . . . . . . . . . . . . . . . . . . . . . . . . . . . . . . . . . . . . . R . . . . . . . . . . . . . . . . . . . . . . . . . . . . . . . . . . . . . . . . . . . MT254426.1_China_Mosquito**

**8698 . . . . . . Y . . . . . . . . . . . . . . . . . . . . . . . . . . . . . . . . . . . . . . . . . . . . . . G . . . . . . . . . . . . . . . . . . . . . . . . . . . . . . . . . . . . . . . . . . . . . . MT560941.1_China_Mosquito**

**8698 . . . . . . . . . . . . . . . . . . . . . . . . . . . . . . . . . . . . . . W . . . . . . . . . . . . . . . . . R . . . . . . . . . . . . . . . . . . . . . . . . . . . . . . . . . . . . . . . . . . . ON875960_India_Assam_Mosquito**

**- G Y L V H V A R S S V P R V R S P R I S K - R P L A E P R E F R R W G G R F R R P K A G I H S P - H S R E A R R - N V C R - H R R V G H Q N H - N R L G K - S Q S A G A F G W - T P H A R P S H N - T Majority**

**------------------+-------------------+-------------------+-------------------+-------------------+-------------------+-------------------+-------------------+-------------------+-------------------+-**

**3010 3020 3030 3040 3050 3060 3070 3080 3090 3100**

**------------------+-------------------+-------------------+-------------------+-------------------+-------------------+-------------------+-------------------+-------------------+-------------------+-**

**8998 Q . H . . . . . W . T . S . . . . F G V P E . . . . . . . . . . . . . . . . L . . . . . . . . P . . Y . . K . . . E . . R . . Y . . . . . . . Y Q S . S R . . . . G . . . S R R . . . . . . . . . . . . AF098735.1_Taiwan_Mosquito**

**8998 Q . . . . . . . . . T . . . . . . . . . P . . . . . . . . . . . . . R . . . . . . . . . . . . P . . . . . . T . . E . . . . . . . . . . . . . Y . . . . . . . . . . . . . . . R . . . . . . . . . . . . GQ902060.1_Thailand_Mosquito**

**8998 Q . H . . . . . W . T . S . . . . F G V P E . . . . V . . . . . . . . S . . L . . . . . . . . P . . . . . K . . . E . . R . . . . . . . . . D Y Q . . F R . . . . G . . . P R R . . . Y . . . . . . . . GQ902063.1_Thailand_Mosquito**

**8998 . . . . . . . . . . . . S . . . . . . . . . . . . . . . S . . . . . . . . . . . . . . . . . . . . . . . . . . . . Q . . . . . . . . . . . . . . . . . . . . . . . . . . . . . . R . . . . . . . . . . . HQ652538.1_China_Mosquito**

**8998 . . . . . . . . . . . . . . . . . . . . . . . . . . . . . . . L . . . . . . . . . . . V . . . . . . . . . . . . . . D . . . . . . . L . . . . . . . . . . . . . . . . . T . . . . . . . . . . . . . . . JF499790.1_Taiwan_Mosquito**

**8998 Q . H . . . . . W . T . S . . . . F G V P E . . . . . . . . . . . . . S . . L . . . . . . . . P . . . . . K . . . E . . R . . Y . . . . . . . Y Q . . F R . . . . G T . . P R R . . . . . . . . . . . . JN864064.1_China_Mosquito**

**8998 . . . . . . . . . . . . . . . . . . . . . . . . . . . . . . . L . . . . . . . . . . . V . . . . . . . . . . . . . . . . . . . . . . L . . . . . . . . . . . . . . . . . T . . . . . . . . . . . . . . . JQ031753.1_Taiwan_Mosquito**

**8998 Q . H . . . . . W . T . S . . . . F G V P E . . . . . . . . . . . . . S . . L . . . . . . . . P . . . . . K . . . E . . R . . Y . . . . . . . Y Q . . F R . . . . G T . . P R R . . . . . . . . . . . . JQ086762.1_China_Mosquito**

**8998 . S . . . . . . . . . . . . . . . . . . . . . . . . . . . . . . . . . . . . . . . . . . . . . P . . . . . . . . . . . . . . . . . . . . . . . . . . . . . . . . . . . . . . . . . . . . . . . . . . . . KT229574.1_China_Mosquito**

**8998 . S . . . . . . . . . . . . . . . . . . . . . . . . . . . . . . . . . . . . . . . . . . . . . P . . . . . . . . . . . . . . . . . . . . . . . . . . . . . . . . . . . . . . . . . . . . . . . . . . . . KT229575.1_China_Mosquito**

**8998 . . . . . . . . . . . . . . . . . . . . . . . . . . . . . . . . . . . . . . . . . . . . . . . . . . . . . . . . . Q . . . . . . . . . . . . . . . . . . . . . . . . . . . . . . . . . . . . . . . . . . LC461957.1_Japan_Mosquito**

**8998 . . . . . . . . . . . . . . . . . . . . . . . . . . . . . . . . . . . . . . . . . . . . . . . . . . . . . . . . . Q . . . . . . . . . . . . . . . . . . . . . . . . . . . S . . . . . . . . . . . . . . LC513838.1_Japan_Mosquito**

**8998 . S H . . . . . . . A I . . I . G S G V L . . . . . . K . E . L . W . S . . . . . T E . R . . P S . . . K K R . . E . . . . . . C W M . . . . N Q . . . . . . G . G S . . L . . R . S . . . S C . . R I LC579814.1_Indonesia_Mosquito**

**8998 . . . . . . . . . . . . . . . . . . . . . . . . . . . . . . . . . . . . . . . . . . . . . . . . . . . . . . . . . . . . . . . . . . . . . . . Y . . . . . . . . . . . . . . . . . . . . . . . . . . . . LC623822.1_Japan_Mosquito**

**8998 . . . . . . . . . . . . . . . . . . . . . . . . . . . . . . . . . . . . . . . . . . . . . . . . . . . . . . . . . . . . . . . . . . . . . . . . . . . . . . . . . . . . . . . . . A . . . . . . . . . I MH385014.1_China_Mosquito**

**8998 . . . . . . . . . . . . . . . . . . . . . . . . . . . . . . . . . . . . . . L . . . . . . . . . . . . . . . . . . . . . . . . . . . . . . . . Y . . . . . . . . . . . . . . . . . . . . . . . . . . . . MK558811.1_China_Mosquito**

**8998 . . . . . . . . . . . . . . . . . . . . . . . . . . . . . . . . . . . . . . . . . . . . . . . . . . . . . . . . . . . . . . . . . . . . . . . . . . . . . . . . . . . . . . . . R . . . . . . . . . . . MT254426.1_China_Mosquito**

**8998 . . . . . . . . . . . . . . . . . . . . . . . . . . . . . . . . . . . . . . . . . . . . . . . . . . . . . . . . . . . . . . . . . . . . . . . Y . . . . . . . . . . . . . . . . . . . . . . . . . . . . MT560941.1_China_Mosquito**

**8998 . . . . . . . . . . . . . . . . . . . . . . . . . . . . . . . . . . . . . . . . . . . . . . . . . . . . . . . . . . . . . . . . . . . . . . . . . . . . . . . . . . . . . . . . . . . . . . . . . . . . ON875960_India_Assam_Mosquito**

**N V Q A Q S G Q G H E T C S R R K D S D G R D I T R R P K G E W A G G D L R S Q H I H E H C R P A C P L D G G - G G H W T T T L G T A A Q E K Q D S C - D L A L - E W R G E S D - D G D Q W R R L C R Q Majority**

**------------------+-------------------+-------------------+-------------------+-------------------+-------------------+-------------------+-------------------+-------------------+-------------------+-**

**3110 3120 3130 3140 3150 3160 3170 3180 3190 3200**

**------------------+-------------------+-------------------+-------------------+-------------------+-------------------+-------------------+-------------------+-------------------+-------------------+-**

**9298 D L . . . . . . . . . . . . . . . . R . . . V F K . . S . . . . T . . H . C . . . F . . . R C . . R Q A . . . . . . . . . . . . . . . T . K . . . . . Q . . . . . . . . . . . . Q . . . . R . . . . . . AF098735.1_Taiwan_Mosquito**

**9298 . . . . . . . . . . . . . . . . E . . . . . . . . . . . . . . . . . . . . C . . . . . . . . . . . . . . . . . . . . . . . . . . . . V . . . . . . . . . . . . . . . . . . . . . . . . . . . . . . R . . GQ902060.1_Thailand_Mosquito**

**9298 D L . . . . . . . . . . . . . . . . R . . . . . K . . S T . . . T S . H . C . . . F . . . R C . . R Q A . . . . . . . . . . . . . A . T . K N . . . . Q . . . . . . . . . . . . Q . . . . R . . . . . . GQ902063.1_Thailand_Mosquito**

**9298 . . . . . . . . . Y . . . . . . . . . . . . . . . . . . . R . R . . . . . . . . . . . . . . . . . . . . . . . . R . . . . . . . . . . . . K . . . . . . . . . F . . . . . . . . E . . . . . . . . R . . HQ652538.1_China_Mosquito**

**9298 . . . . . . . . . Y . . . . . . . . . . . . . . . . . . . . . . . . . . . . . . . . . . . . . . . . . . . . . . . . . . . . . . . . . . . . . . N . . . . . . . . . . . . . . . . . . . . . . . . . . . JF499790.1_Taiwan_Mosquito**

**9298 D L . . . . . . . . . . . . . . . . R . . . . . K . . S . . . . T . . H . C . . . F . . . R C . . R Q A . . . . . . . . . . . . . . S T . . . . . . . Q . . . . . . . . . . . . Q . . . . R . . . . . . JN864064.1_China_Mosquito**

**9298 . . . . . . . . . Y . . . . . . . . . . . . . . . . . . . . . . . . . . . . P . . . . . . . . . . . . . . . . . R . . . . . . . . . . . . . . . N . . . . . . . R . . . . . . . . . . . . . . . . . . . JQ031753.1_Taiwan_Mosquito**

**9298 D L . . . . . . . . . . . . . . . . R . . . . . K . . S . . . . T . . H . C . . . F . . . R C . . R Q A . . . . . . . . . . . . . . S T . . . . . . . Q . . . . . . . . . . . . Q . . . . R . . . . . . JQ086762.1_China_Mosquito**

**9298 . . . . . . . . . Y . . . . . . . . . . . . . . . . . . . . . . . . . . . . . . . . . . . . . . . . . . . . . . . . . . . . . . . . . . . K . . N . . . . . . . . . . . . . . . . . . . . . . . . . . . KT229574.1_China_Mosquito**

**9298 . . . . . . . . . Y . . . . . . . . . . . . . . . . . . . . . . . . . . . . . . . . . . . . . . . . . . . . . . . . . . . . . . . . . . . K . . N . . . . . . . . . . . . . . . . . . . . . . . . . . . KT229575.1_China_Mosquito**

**9298 . . . . . . . . . . . . . . . . E . . . . . . . . . . . . . . R . . S . . . . . . . . . . . . . . . . . . . . . . . . . . . . . . . . . . K . . . . . . . . . . . . . . . . . . . . . . . . . . . R . . LC461957.1_Japan_Mosquito**

**9298 . . . . . . . . . . . . . . . . E . . . . . . . . . . . . . . R . . . . . . . . . . . . . . . . . . . . . . . . . . . . . . . . . . . . . . . . . . . . . . . . . . . . . . . . . . . . . . . . . R . . LC513838.1_Japan_Mosquito**

**9298 D I . T . . S . S . . A L D . . . . R . . . . . K . . . E R Q R . S . H . C . . . L . K . . C . . S . T . . . . R . . . . . . R . . I . . . . . . . S . N . V V . K . . . K . V Q N . . . R . . . . C . LC579814.1_Indonesia_Mosquito**

**9298 . . . . . . . . . . . A R . . . . . . . . . . . . . . . . . . R . . . . . . . . . . . . . . . . T . . . . . . . . . . . . . . . . . . . . K . . . . . . . . . . . . . . . . . . Q . . . . . . . . R . . LC623822.1_Japan_Mosquito**

**9298 . . . . . . . . . Y . . . . . . . . . . . . . . . . . . . . . . . . . . . . . . . . . . . . . . . R . . . . . . . . . . . . . . . . . . . . R . N . . . . . . . . . . . . . . . . . . . . . . . . R . . MH385014.1_China_Mosquito**

**9298 . . . . . . . . . . . A R . . . . . . . . . . . . . . . . . . R . . . . . . . . . . . . . . . . . . . . . . . . . . . . . . . . . . . . . K . . . . . . . . . . . . . . . . . . . . . . . . . . . . . . MK558811.1_China_Mosquito**

**9298 . . . . . . . . . Y . . . . . . . . . . . . . . . . . . . . K . . . . . . . . . . . . . . . . . . . . . . . . . . . . . . . . . . . . . . . . . N . . . . . . . . . . . . . . . . . . . . R . . . R . . MT254426.1_China_Mosquito**

**9298 . . . . . . . . . . . . R . . . . . . . . . . . . . . . . . . R . . . . . . . . . . . . . . . . . . . . . . . . . . . . . . . . . . . . . K . . . . . . . . . . . . . . . . . . . . . . . . . . . R . . MT560941.1_China_Mosquito**

**9298 . . . . . . . . . Y . . . . . . . . . . . . . . . . . . . . . . . . . . . C . . . . . . . . . . . . . . . . . . . . . . . . . . . . . . . . R . N . . . N . . . . . . . . . . . . . . . . . . . . R . . ON875960_India_Assam_Mosquito**

**A A G - Q I R H G S P F P Q R N V E G Q K R Y P R M E A F A W L A R L A A G S L L L Q S F S G D C D E R W K E H S R P V Q R A G - A D W Q G A H L P R S W M E C E G H S L P G Q S V C T D V A A S I L P Majority**

**------------------+-------------------+-------------------+-------------------+-------------------+-------------------+-------------------+-------------------+-------------------+-------------------+-**

**3210 3220 3230 3240 3250 3260 3270 3280 3290 3300**

**------------------+-------------------+-------------------+-------------------+-------------------+-------------------+-------------------+-------------------+-------------------+-------------------+-**

**9598 . . . R . . . . . P . L . . . . . K . . . . H . G . . . . . . . . . . . . S . . . . . P . . . . . . . . . . . Y . C . . . . T . . . . R . . S . . . . . . . . . . . . . . S . . . I . . . E . T P . . . AF098735.1_Taiwan_Mosquito**

**9598 . . . . . . . . . . . . . . . . . . . . . . . . G . . . . S . . . . . . . . P . . . . . . . . . . . . . . . . . . . . . . . . . . . . . . . . . . . . . . . . . . . . . . S . . . . . . . . . . P . . . GQ902060.1_Thailand_Mosquito**

**9598 . . . R . . . Y S P . L . . . . . K . . . . H . G . . . . S . . . . . . . S . . . . . P . . . . . . . . . . . Y . . . . . . T . . . . R . . S . . S . . . . . . . . . G . . . . . I . . . . . T P . . . GQ902063.1_Thailand_Mosquito**

**9598 . . . . . . . . . . . . . . . . . . . . . . . . . . . . . . . . . . . . . . . . . . . . . . . . . . . . . . . . . . . . . . . . . . . . . . . . . . . . . . . . . . . G . . S . . . . . . . . . . . . . HQ652538.1_China_Mosquito**

**9598 . . . . . . . . . P . . . . . . . . . . . . . . . . . . . . . . . . . . . . . . . . . . . . . . . . . . . . . . . . . . . . . . . . . . . . . . . . . . . . . . . . . . . . . . . . . . . . . . . . . . JF499790.1_Taiwan_Mosquito**

**9598 T . . R . . . . S P . L . . . . . K . . . . H . G . . . . . . . . . . . . S . . . F . P . . . . . . . . . . . Y . C . . . . T . . . . R . . S . . S . . . . . . . . . . . . . . . I . . . . . T P . . . JN864064.1_China_Mosquito**

**9598 . . . . . . . . . P . . . . . . . . . . . . . . . . . . . . . . . . . . . . . . . . . . . . . . . . . . . . . . . . . . . . . . . . . . . . . . . . . . . . . . . . . . . . . . . . R . N . . . . . . . JQ031753.1_Taiwan_Mosquito**

**9598 T . . R . . . . S P . L . . . . . K . . . . H . G . . . . . . . . . . . . S . . . F . P . . . . . . . . . . . Y . C . . . . T . . . . R . . S . . S . . . . . . . . . . . . . . . I . . . . . T P . . . JQ086762.1_China_Mosquito**

**9598 . . . . . . . . . . . . . . . . . . . . . . . . . . . . . . . . . . . . . . . . . . . . . . . . . . . . . . . . . . . . . . . . . . . . . . . . . . . . . . . . . . . . . . S . . . . . . . . . . . . . KT229574.1_China_Mosquito**

**9598 . . . . . . . . . . . . . . . . . . . . . . . . . . . . . . . . . . . . . . . . . . . . . . . . . . . . . . . . . . . . . . . . . . . . . . . . . . . . . . . . . . . . . . S . . . . . . . . . . . . . KT229575.1_China_Mosquito**

**9598 . . . . . . . . . . . . . . . . . . . . . . . . . . . . . . . . . . . . . . . . . . . . . . . . . . . . . . . . . . . . . . . . . . . . . . . . . . . . . . . . . . . . . . . . . . . . . . . . . . F . LC461957.1_Japan_Mosquito**

**9598 . . . . . . . . . . . . . . . . . . . . . . . . . . . . . . . . . . . . . . . . . . . . . . . . . . . . . . . . . . . . . . . . . . . . . . . . . . . . . . . . . . . . . . . . . . . . . . . . . . . . LC513838.1_Japan_Mosquito**

**9598 . . . . . . C . S P S L S . . D . . . . E . H . G . . T L P . . . . . . . S . F . . . P . P . . . N . G . E . . . C . M . G P . . . N R . . T . . S . G . V . . K . . . . L S . . L R A N . . . L V . . LC579814.1_Indonesia_Mosquito**

**9598 . . . . . . . . . . . . . . . . . . . . . . . . . . . . . . . . . . . . . . . . . . . . . . . . . . . . . . . . . . . . . . . . . . . . . . . . . . . . . V . . . . . . . . . . . . . . . . . . . . . . LC623822.1_Japan_Mosquito**

**9598 . . . . . . . . . . . . . . . . . . . . . . . . . . . . . . . . . . . . . S . . . . . P . . . . . . . . . . . . . C . . . . T . . . . R . . S . . S . . . . . . . . . . . S . . . I . . . . . T P . . . MH385014.1_China_Mosquito**

**9598 . . . . . . . . . . . . . . . . . . . . . . . . . . . . . . . . . . . . . . . . . . . . . . . . . . . . . . . . . . . . . . . . . . . . . . . . . . . . . . . . . . . . . . . . . . . . . . . T . . . . MK558811.1_China_Mosquito**

**9598 T . . . . . . . . . . . . . . . . . . . . . . . . . . . . . . . . . . . . . . . . . . . . . . . . . . . . . . . . . . . . . . . . . . . . . . . . . . . . . . . . . . . . . . . . . . . . . . . . . . . MT254426.1_China_Mosquito**

**9598 . . . . . . . . . . . . . . . . . . . . . . . . . . . . . . . . . . . . . . P . . . . . . . . . . . . . . . . . . . . . . . . . . . . . . . . . . . . . . . . . . . . . . . . . . . . . . . . . . . . . MT560941.1_China_Mosquito**

**9598 . . . . . . . . . . . . . . . . . . . . . . . . . . . . . . . . . . . . . . . . . . . . . . . . . . . . . . . . . . . . . . . . . . . . . . . . . . . . . . . . . . . . . . . . . . . . . . . . . . . . ON875960_India_Assam_Mosquito**

**S E G P T P H G K C N L L S S S S G L G A H R Q N I L V N T L K R R V D D H - R H A A S L E Q S M D - R K - M D D G Q D P N H K L D R R S V R G K A - G H L V W Q S H R N A I Q G N M G - E H L R G N K Majority**

**------------------+-------------------+-------------------+-------------------+-------------------+-------------------+-------------------+-------------------+-------------------+-------------------+-**

**3310 3320 3330 3340 3350 3360 3370 3380 3390 3400**

**------------------+-------------------+-------------------+-------------------+-------------------+-------------------+-------------------+-------------------+-------------------+-------------------+-**

**9898 . . . L A S . . . . D . . . . A . . . . . . G . D . . . D . . E . . . . . . G . . . . G . . . . L . . . . . . . . . . . S . . . . . . . . . . . . . . . . . . . . P . . . . . . S . L . R . . . . . D . AF098735.1_Taiwan_Mosquito**

**9898 . . . . . . . . E . . . . . . . . . . . . . . . . . . . . . . . . . . . . . . . . . . . . . . G V . . . . . . . . . . . . . . . . . . . P I . . . . . . . . . . . . . . . . . . . . . . . . . . . . . . GQ902060.1_Thailand_Mosquito**

**9898 P . . L A S . . . . . . . . . A . . . . . . . . D . . . . . . E . . . . . . G G . . . G . . . . L . . . . . . . . . . . S . . . . . . . . . C . . . . . . . . . . P . W . . . . S . L . . . . . C . . . GQ902063.1_Thailand_Mosquito**

**9898 . . . . . . . . . . . . . . . . . . . . . . . . . . . . . . . . G . . . . . . . . . . . . . . G . . . . . . . . . . . . . . . . . . . . . . . . . . . . . . . . . . . . . . . . . . . . . . . . . . . . HQ652538.1_China_Mosquito**

**9898 . . . . . . . . . . . . . . . . . . . . . . . . . . . . . . . . . . . . . . . . . . . . . . . G . . . . . . . . . . . . . . . . . . . . . . . . . . . . . . . . . . . . . . . . . . . . . . . . . . . . JF499790.1_Taiwan_Mosquito**

**9898 . Q . L A S . . . . D . . . . A . R . . . . . . D . . . . . . E . . . . . . G . . . . G . . . . L . . . . . . . . . . . S . . . . . . . . . C . . . R . . . . . . P . . . . . . S . L D . . . . C . D . JN864064.1_China_Mosquito**

**9898 . . . . . . . . . . . . . . . . . . . . . Y . . . . . . . . . . . . . . . . . . . . . . . . . . . . . . . . . . . . . . . . . . . . . . . . . . . . . . . . . . . . . . . . . . . . . . . . . . . . . . JQ031753.1_Taiwan_Mosquito**

**9898 . Q . L A S . . . . D . . . . A . R . . . . . . D . . . . . . E . . . . . . G . . . . G . . . . L . . . . . . . . . . . S . . . . . . . . . C . . . R . . . . . . P . . . . . . S . L D . . . . C . D . JQ086762.1_China_Mosquito**

**9898 . . . . . . . . . . . . . . . . . . . . . . . . . . . . . . . . . . . . . . . . . . . . . . . G . . . . . . . . . . . . . . . . . . . . . . . . . . . . . . . . . . . . . . . . . . . . . K . . . . . . KT229574.1_China_Mosquito**

**9898 . . . . . . . . . . . . . . . . . . . . . . . . . . . . . . . . . . . . . . . . . . . . . . . G . . . . . . . . . . . . . . . . . . . . . . . . . . . . . . . . . . . . . . . . . . . . . K . . . . . . KT229575.1_China_Mosquito**

**9898 . . . . . . . . . . . . . . . . . . . . . . . . . . . . . . . . . . . . . . . . . . . . . . . . . . . . . . . . . . . . . . . . . . . . . . . . . . . . . . . . . . . . . . . . . . . . . . R . . . . . LC461957.1_Japan_Mosquito**

**9898 . . . . . . . . . . . . . . . . . . . . . . . . . . . . . . . . . . . . . . . . Y . . . . . . . . . . . . . . . . . . . . . . . . . . . . . . . . . . . . . . . . . . . . . . . . . . . . . R . . . . . LC513838.1_Japan_Mosquito**

**9898 P Q R L A S . . . R . . . . . A . . . . . . . . . . . . D P . . G . M . . . . . . . . G . . . . L . R . . . . . G R . . . . . E . . . C P L . . . T . . . . . R . . . . . . H . S . L . . . . . C S . . LC579814.1_Indonesia_Mosquito**

**9898 . . . . . . . . . . . . . . . . . . . . . . . . . . . . . . . . . . . . . . . . . . T . . . . . . . . . . . . . . . . . . . . . . . . . . . . . . . . . . . . . . . . . . . . . . . . . . . . . . . . . LC623822.1_Japan_Mosquito**

**9898 . . . L A S . . . . D . . . . A . . . . . . . . D V . . . . . E . . . . . . G . . . . G . . . . L . . . . . . . . . . . S . . . . . . . . . C . . . . . . . . . . P . . . . . . S . L . . . . . C . D . MH385014.1_China_Mosquito**

**9898 . . . . . . . . . . . . . . . . . . . . . . . . . . . . . . . . . . . . . . . . . . T . . . . . . . . . . . . . . . . . . . . . . . . . . . . . . . . . . . . . . . . . . . . . . . . . . . . . . . . . MK558811.1_China_Mosquito**

**9898 . . . L . . . . . . . . . . . . . . . . . . . . . . . . . . . . . . . . . . . . . . . . . . . G . . . . . . . . . . . . . . . . . . . . . . . . . . . . . . . . . . Y . . . . . . . . . . . . . . S . . MT254426.1_China_Mosquito**

**9898 . . . . . . . . . . . . . . . . . . . . . . . . . . . . . . . . . . . . . . . . . . . . . . . . . . . . . . . . . . . . . . . . . . . H . . . . . . . . . . . . . . . . . . . . . . . . . . . . . . . . MT560941.1_China_Mosquito**

**9898 . . . . . . . . . . . . . . . . . . . . . . . . . . . . . . . . . . . . . . . . . . . . . . . G . . . . . . . . . . . . . . Y . . . . . . . . . . . . . . . . . . . . . . . . . . . . . . . . . . . . . ON875960_India_Assam_Mosquito**

**P S E G H H W K R K L C - L H D F P Q K I - G C I D S G G Q G H L - Majority**

**------------------+-------------------+-------------------+---------**

**3410 3420 3430**

**------------------+-------------------+-------------------+---------**

**10198 . G . S C . . E . . . . . . . . L T . E . R R R L . P . R . . . . AF098735.1_Taiwan_Mosquito**

**10198 . . . S R . . . . . . . . . . . . . . . . . . . F . . . . . . Y . GQ902060.1_Thailand_Mosquito**

**10198 . G . S C . R . . . . . . . . . L T . E . R R R F . P . R . . . . GQ902063.1_Thailand_Mosquito**

**10198 . . . . . . . . . . . . . . . . . . . . V . . . . . . . . . . . . HQ652538.1_China_Mosquito**

**10198 . . . . . . . . . . . . . . . . . . . . . . . . . . . . . . . . . JF499790.1_Taiwan_Mosquito**

**10198 . G . S C . . E . . . . . . . . L T . E . R R R L . P . R . . . . JN864064.1_China_Mosquito**

**10198 . . . . . . . . . . . . . . . . . . . . . . . . . . . . . . . . . JQ031753.1_Taiwan_Mosquito**

**10198 . G . S C . . E . . . . . . . . L T . E . R R R L . P . R . . . . JQ086762.1_China_Mosquito**

**10198 . . . . . . . . . . . . . . . . . . . . . . . . . . . . . . . . . KT229574.1_China_Mosquito**

**10198 . . . . . . . . . . . . . . . . . . . . . . . . . . . . . . . . . KT229575.1_China_Mosquito**

**10198 . . . . . . . . . . . . . . . . . . . . . . . . . . . . . . . . . LC461957.1_Japan_Mosquito**

**10198 . . . . . . . . . . . . . . . . . . . . . . . . . . . . . . . . . LC513838.1_Japan_Mosquito**

**10198 S . . S N . R E . D . . R . . . I T . E . R . . F . P . R . . . . LC579814.1_Indonesia_Mosquito**

**10198 . . . . . . . . . . . . . . . . . . . . . . . . . . . . . . . . . LC623822.1_Japan_Mosquito**

**10198 . G Q S C . R E . . . . . . . . L T . E . R R R P . P . R . . . . MH385014.1_China_Mosquito**

**10198 . . . . Y . . . . . . . . . . . . . . . . . . . . . . . . . . . . MK558811.1_China_Mosquito**

**10198 . . . . . . . . . . . . . . . . . . . . . . . . V . . . . . . . . MT254426.1_China_Mosquito**

**10198 . . . . . . . . . . . . . . . . . . . . . . . . . . . . . . . . . MT560941.1_China_Mosquito**

**10198 . . . . . . . . . . . . . . . . . . . . . . . . V . . . . . . . . ON875960_India_Assam_Mosquito**
