# Supplementary material for: Japanese Encephalitis Virus Genotype III Strains Detection and Genome Sequencing from Indian Pig and Mosquito Vector
Source: Vaccines (Basel). 2023 Jan 10;11(1):150. doi: 10.3390/vaccines11010150 (PMC9862938; doi:10.3390/vaccines11010150)
Supplement: Supplementary file 1 [file vaccines-11-00150-s001.zip › vaccines-2082975-supplementary/Supplementary table 1.docx]

| **Supplementary Table 1: Reference sequences used in this study** | | | |
| --- | --- | --- | --- |
| *List of Polyprotein sequences of JEV isolated from Pig* | | | |
| **Country** | **Genotype** | **Source** | **Genome Accession No.** |
| China | - | Pig | KC915016.1 |
| Japan | - | Pig | LC708276.1 |
| Thailand | - | Pig | GQ902061.1 |
| Thailand | - | Pig | GQ902058.1 |
| South Korea | - | Pig | KT447437.1 |
| India | I | pig | MT232844.1 |
| China | I | - | JN381872.1 |
| South Korea | I | Pig | AY316157.1 |
| Australia | I | Pig | MT253733.1 |
| Cambodia | I | Pig | KY927818.1 |
| China | I | Pig | MN544780.1 |
| Australia | II | Pig | MT253737.1 |
| India | III | Pig | MZ702743.1 |
| Japan | III | Pig | LC461960.1 |
| USA | III | - | U15763.1 |
| South Korea | III | - | KF711994.1 |
| Japan | III | Pig Serum | AB551990.1 |
| China | III | Pig | AY849939.1 |
| China | III | Pig | EF107523.1 |
| India | III | Pig | KP164498.2 |
| China | III | Pig | KX965684.1 |
| China | III | Pig | KU363309.1 |
| China | III | Pig | KF297916.1 |
| *List of Polyprotein sequences of JEV isolated from Mosquito* | | | |
| Taiwan | - | *Aedes albopictus* | AF098735.1 |
| China | - | Mosquito | JN864064.1 |
| China | I | Mosquito | MH385014.1 |
| China | I | *Culex tritaeniorhynchus* | KT229575.1 |
| China | I | *Culex tritaeniorhynchus* | KT229574.1 |
| China | I | *Culex tritaeniorhynchus* | KT229573.1 |
| China | - | Mosquito | JQ086762.1 |
| Thailand | I | Mosquito | GQ902063.1 |
| Thailand | - | Mosquito | GQ902060.1 |
| Taiwan | - | Mosquito | JF499790.1 |
| China | - | Mosquito | HQ652538.1 |
| Taiwan | - | Mosquito | JQ031753.1 |
| China | - | *Culex tritaeniorhynchus* | MT254426.1 |
| China | - | *Culex quinquefasciatus* | MT560941.1 |
| Japan | I | *Culex tritaeniorhynchus* | LC623822.1 |
| Indonesia | IV | *Culex vishnui* | LC579814.1 |
| Japan | I-a | *Culex tritaeniorhynchus* | LC461957.1 |
| China | - | *Culex tritaeniorhynchus* | MK558811.1 |
| Japan | - | *Culex tritaeniorhynchus* | LC513838.1 |
