# Supplementary material for: Japanese Encephalitis Virus Genotype III Strains Detection and Genome Sequencing from Indian Pig and Mosquito Vector
Source: Vaccines (Basel). 2023 Jan 10;11(1):150. doi: 10.3390/vaccines11010150 (PMC9862938; doi:10.3390/vaccines11010150)
Supplement: Supplementary file 1 [file vaccines-11-00150-s001.zip › vaccines-2082975-supplementary/Supplementary Table 2.docx]

| **Supplementary Table 2: List of substitution of nucleotides in JEV isolated from Mosquito** | | | |
| --- | --- | --- | --- |
| Serial no. | Nucleotide Position in Polyprotein gene of JEV isolated from Mosquito | Nucleotide present (Origin) | Instead of |
|  | 147 | C | T |
|  | 777 | C | T |
|  | 1083 | T | C |
|  | 2755 | T | G |
|  | 3876 | G | A |
|  | 4128 | G | A/T |
|  | 4725 | C | T |
|  | 4948 | G | A |
|  | 4995 | T | C |
|  | 5043 | C | A/T |
|  | 5922 | G | A |
|  | 6168 | A | G |
|  | 6243 | T | C |
|  | 6839 | G | A |
|  | 7071 | T | A/C |
|  | 7975 | T | C |
|  | 8389 | A | G |
|  | 8420 | G | A |
|  | 8677 | C | T |
|  | 10087 | T | C |
