# Supplementary material for: Japanese Encephalitis Virus Genotype III Strains Detection and Genome Sequencing from Indian Pig and Mosquito Vector
Source: Vaccines (Basel). 2023 Jan 10;11(1):150. doi: 10.3390/vaccines11010150 (PMC9862938; doi:10.3390/vaccines11010150)
Supplement: Supplementary file 1 [file vaccines-11-00150-s001.zip › vaccines-2082975-supplementary/Supplementary Table 3.docx]

| **Supplementary Table 3: List of substitution of nucleotides in JEV isolated from pig** | | | |
| --- | --- | --- | --- |
| Serial no. | Nucleotide Position in Polyprotein gene of JEV isolated from pig | Nucleotide present (Origin) | Instead of |
|  | 1901 | C | T |
|  | 4252 | A | G |
|  | 6120 | A | G |
|  | 6486 | G | A |
|  | 9268 | G | C |
|  | 9528 | A | G |
|  | 9511 | G | A |
