# Supplementary material for: Japanese Encephalitis Virus Genotype III Strains Detection and Genome Sequencing from Indian Pig and Mosquito Vector
Source: Vaccines (Basel). 2023 Jan 10;11(1):150. doi: 10.3390/vaccines11010150 (PMC9862938; doi:10.3390/vaccines11010150)
Supplement: Supplementary file 1 [file vaccines-11-00150-s001.zip › vaccines-2082975-supplementary/Supplementary Table 4.docx]

| **Supplementary Table 4: List of substitution of nucleotides in two strains of JEV isolated from Indian pigs** | | | |
| --- | --- | --- | --- |
| Serial no. | Nucleotide Position in Polyprotein gene of JEV isolated from Indian Pig | Nucleotide present (Origin) | Instead of |
|  | 147 | C | T |
|  | 4926 | T | C |
|  | 5550 | T | C |
|  | 5556 | A | G |
|  | 6498 | C | T |
|  | 7422 | A | G |
|  | 7515 | T | C |
|  | 8337 | G | A |
|  | 8673 | A | G |
|  | 9099 | C | A |
|  | 9842 | G | A |
|  | 10086 | T | C |
|  | 4128 | G | A |
|  | 3690 | A | G |
|  | 2793 | G | A |
