# Supplementary material for: Japanese Encephalitis Virus Genotype III Strains Detection and Genome Sequencing from Indian Pig and Mosquito Vector
Source: Vaccines (Basel). 2023 Jan 10;11(1):150. doi: 10.3390/vaccines11010150 (PMC9862938; doi:10.3390/vaccines11010150)
Supplement: Supplementary file 1 [file vaccines-11-00150-s001.zip › vaccines-2082975-supplementary/Supplementary table 5.docx]

| **Supplementary Table 5: List of substitution of amino acid in JEV isolated from pig (MZ702743.1)** | | | |
| --- | --- | --- | --- |
| Serial no. | Amino acid Position in Polyprotein gene of JEV isolated from pig | Amino acid present (Origin) | Instead of |
|  | 1418 | V | I |
|  | 2383 | F | C |
|  | 3090 | H | D |
|  | 3171 | N | D |
