# Supplementary material for: Japanese Encephalitis Virus Genotype III Strains Detection and Genome Sequencing from Indian Pig and Mosquito Vector
Source: Vaccines (Basel). 2023 Jan 10;11(1):150. doi: 10.3390/vaccines11010150 (PMC9862938; doi:10.3390/vaccines11010150)
Supplement: Supplementary file 1 [file vaccines-11-00150-s001.zip › vaccines-2082975-supplementary/Supplementary table 6.docx]

| **Supplementary Table 6: List of substitution of amino acid in polyprotein gene of JEV isolated from mosquito (ON875960)** | | | |
| --- | --- | --- | --- |
| Serial no. | Amino acid Position in Polyprotein gene of JEV isolated from mosquito | Amino acid present (Origin) | Instead of |
|  | 919 | A | S |
|  | 1650 | I | V |
|  | 2280 | K | R |
|  | 2259 | R | C |
|  | 2797 | G | R |
|  | 2810 | N | D |
|  | 2893 | S | P |
|  | 3363 | H | Y |
